# N-Heterocyclic Carbene to Actinide d-Based π-bonding Correlates with Observed Metal–Carbene Bond Length Shortening Versus Lanthanide Congeners

**DOI:** 10.1021/jacs.3c12721

**Published:** 2024-04-03

**Authors:** Conrad A. P. Goodwin, Ralph W. Adams, Andrew J. Gaunt, Susan K. Hanson, Michael T. Janicke, Nikolas Kaltsoyannis, Stephen T. Liddle, Iain May, Jeffrey L. Miller, Brian L. Scott, John A. Seed, George F. S. Whitehead

**Affiliations:** † Chemistry Division, Los Alamos National Laboratory, Los Alamos, New Mexico 87545, United States; ‡ Centre for Radiochemistry Research, The University of Manchester, Oxford Road, Manchester M13 9PL, U.K.; § Department of Chemistry, The University of Manchester, Oxford Road, Manchester M13 9PL, U.K.; ∥ Materials Physics & Applications Division, Los Alamos National Laboratory, Los Alamos, New Mexico, 87545, United States

## Abstract

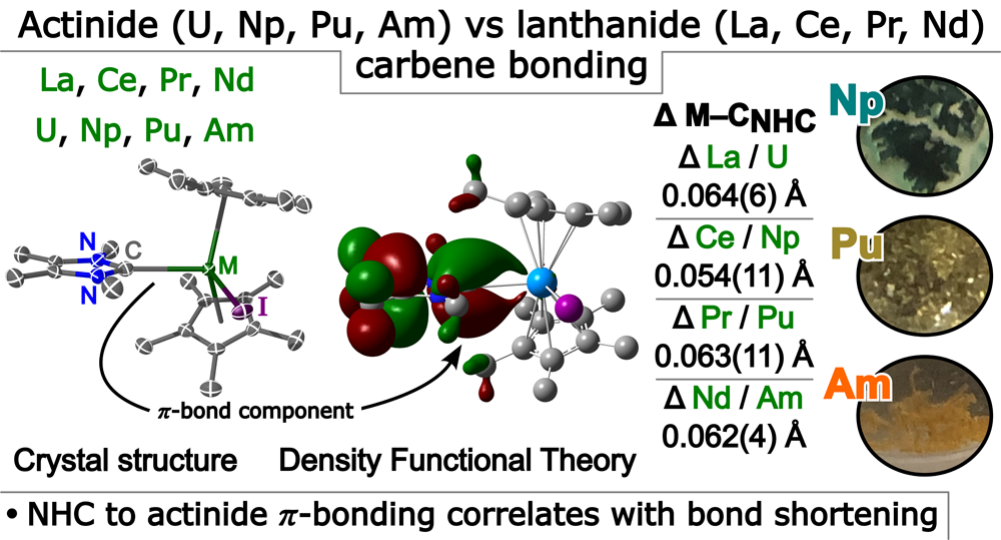

Comparison of bonding and electronic structural features between trivalent lanthanide (Ln) and actinide (An) complexes across homologous series’ of molecules can provide insights into subtle and overt periodic trends. Of keen interest and debate is the extent to which the valence f- and d-orbitals of trivalent Ln/An ions engage in covalent interactions with different ligand donor functionalities and, crucially, how bonding differences change as both the Ln and An series are traversed. Synthesis and characterization (SC-XRD, NMR, UV–vis–NIR, and computational modeling) of the homologous lanthanide and actinide N-heterocyclic carbene (NHC) complexes [M(C_5_Me_5_)_2_(X)(I^Me4^)] {X = I, M = La, Ce, Pr, Nd, U, Np, Pu; X = Cl, M = Nd; X = I/Cl, M = Nd, Am; and I^Me4^ = [C(NMeCMe)_2_]} reveals consistently shorter An–C vs Ln–C distances that do not substantially converge upon reaching Am^3+^/Nd^3+^ comparison. Specifically, the difference of 0.064(6) Å observed in the La/U pair is comparable to the 0.062(4) Å difference observed in the Nd/Am pair. Computational analyses suggest that the cause of this unusual observation is rooted in the presence of π-bonding with the valence d-orbital manifold in actinide complexes that is not present in the lanthanide congeners. This is in contrast to other documented cases of shorter An–ligand vs Ln–ligand distances, which are often attributed to increased 5f vs 4f radial diffusivity leading to differences in 4f and 5f orbital bonding involvement. Moreover, in these traditional observations, as the 5f series is traversed, the 5f manifold contracts such that by americium structural studies often find no statistically significant Am^3+^vs Nd^3+^ metal–ligand bond length differences.

## Introduction

Our understanding of the chemical bonding and coordination chemistry of the lanthanide (Ln) and actinide (An) elements has evolved substantially over the last century.^1−7^ Through this period, the postulate that Ln^3+^ cations operate in a highly ionic bonding regime, comparable to that of alkaline earth metals, has remained essentially unchanged.^3^ The core-like 4f manifold is too radially contracted to engage in substantial spatial-overlap-driven covalent interactions with bound ligands. In contrast to the lanthanides, many actinides have multiple readily accessible oxidation states, and the greater radial expansion of the 5f manifold (versus 4f) can result in appreciable interaction with ligand orbitals,^8^ though this decreases across the 5f row as *Z*_eff_ increases.^9−20^ Complexes in higher oxidation states (e.g., IV–VI), particularly those of uranium which are more studied than transuranium complexes, frequently show that the more radially diffuse 6d manifold (versus 5f) is capable of accepting ligand density and participates in multiple bonding interactions such as in actinyl or mono-oxo ({AnO_*x*_}^*n*+^) or bis-({An(NR)_2_}^*n*+^) linkages,^21−24^ and in other An=E combinations.^7,25−33^ In some instances, the covalency of An=element multiple bonds, featuring rich 5f, 6d, and 7p components, can approach or surpass that of transition metal complexes.^34^

Differences between lanthanide and actinide complexes, brought about, at least in part, by variations in metal–ligand interactions, are exploited in separations science applications.^35−41^ However, crystallographic studies comparing soft and hard σ-donor binding in metal pairs with similar ionic radii (e.g., 6-coordinate Ce^3+^ = 1.01 Å; U^3+^ = 1.025 Å) often show that for hard (e.g., N, O) σ donors, the two metals behave similarly and display minimal bond length differences. In studies of soft donors (e.g., Se, Te, and N-heterocycles) with f-block metals, bonding differences are often observed between the lanthanide and actinide series; however, to the best of our knowledge,^11,14−18,20,42−47^ in every homologous series studied thus far by single-crystal X-ray diffraction (SC-XRD), the magnitude of differences decreases to statistical insignificance as the f-block is traversed. This effect is such that by Am, an element challenging to separate/chemically distinguish from lanthanides, Am–ligand versus lanthanide–ligand bond length differences are usually statistically insignificant, at least with conventional statistical treatments.^48^ Nevertheless, these differences are exploited in Am^3+^, Cm^3+^, and Ln^3+^ separation schemes.^36−41^ Turning to π-bonding, where both metals are trivalent, Ln^3+^ ions are much less likely to have (energetically and spatially) accessible orbitals to engage in such interactions than An^3+^ ions.^49^ With π-acids (e.g., CO, {CN}^−^), actinide–ligand bonds are almost invariably shorter than lanthanide–ligand bonds,^50,51^ but much more frequently, the corresponding lanthanide complexes are simply not isolable.^52−54^ The prospect of U^3+^ 5f → L [or involving ligand symmetry-matched orbitals from the (U^3+^L_3_) fragment] π back-bonding has been advanced as a plausible mechanism to explain some of these differences.^52,55−61^ The opposite case, of L → M π-bonding, is rarely documented outside of multiply bonded species, which are challenging to isolate for trivalent lanthanides, hindering comparative studies. In the case of amido and alkoxide systems which may permit L → M π-donation, reports show that where present this involves donation to the 5f manifold.^62,63^ In some simple systems such as the [MCl_6_]^3−^ series, differences attributable to L → An π (L → 6d and 5f) contributions have been described.^64^

Furthermore, if π back-bonding from metal f orbitals drives an observable effect, then transuranium actinides might be expected to show a weaker effect than uranium due to radial contraction from larger *Z*_eff_; thus, a homologous series that spans several trivalent actinides is necessary to establish the nature of π-bonding and how it changes as the 5f row is traversed—few such homologous series are well studied.^11,14−18,20,43−47,50,65−71^

**Figure 1:**
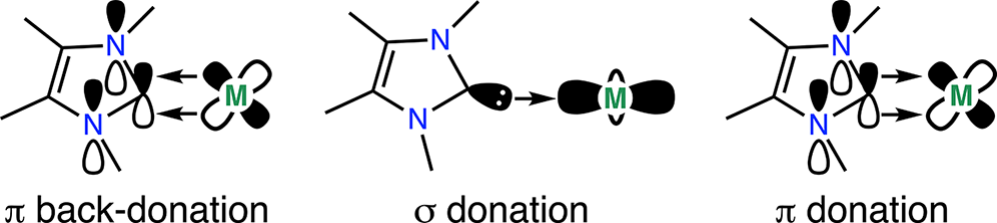
Various bonding interactions between an NHC ligand and d-orbitals.

NHCs (NHC = N-heterocyclic carbene) are classically regarded as strong C_NHC_ → M σ^2^ donors which means they will bind tightly and form isolable complexes offering a promising avenue to address some of the hindering factors above. Indeed, some transuranium NHC-complexes have recently been reported.^25^ Where orbital occupancy and overlap allow, synergic M → NHC π back-donation from low-valent transition metals is often observed (Figure 1 left). For Ln^3+^, the valence electrons, where present, reside in highly spatially contracted 4f-orbitals and so M → NHC back-donation is likely to be weak or absent. The greater radial extent of the 5f valence orbital set on An^3+^ (relative to 4f) may be significant enough to drive observable physical differences, such as bond length differences, derived from the differing f-orbital properties.^51,56^ Furthermore, many NHCs show nonzero 2p_z_ density at the carbenic carbon, which in principle can give rise to C_NHC_ → M π-bonding interactions – i.e., σ^2^π^x^ bonds, where x is small but nonzero (Figure 1 right).^72−80^ The somewhat (relative to 4f) radially expanded 5f and 6d (vacant) orbital sets on An^3+^ ions could both provide spatial overlap to form C_NHC_ → M π-bonding interactions. As such, f-block NHC complexes present a potential testbed for the investigation of lanthanide/actinide structural differences derived from both f-based M → L π back-bonding and L → M f- or d-based π-bonding.

Ephritikhine and co-workers have explored the binding of the simple NHC {C(NMeCMe)_2_}, denoted I^Me4^ henceforth, to both Ce^3+^ and U^3+^ within the [M(Cp^t^)_3_(I^Me4^)] (Cp^t^ = {C_5_H_4_-*t*Bu}) and also [M(Cp*)_2_(I)(I^Me4^)] (Cp* = {C_5_Me_5_}) frameworks.^51^ High-quality single-crystal X-ray diffraction data were obtained from reactions using just ∼25-35 mg of starting material, and the data showed that the U–C_NHC_ distance was 0.037 Å shorter than Ce–C_NHC_; note that (6-coordinate) Ce^3+^ is ca. 0.02 Å smaller than U^3+^, which hinders an ideal comparison. Here, we report the preparation of [M(C_5_Me_5_)_2_(X)(I^Me4^)] (X = I, M = La, Ce, Pr, Nd, U, Np, Pu; X = Cl, M = Nd; X = I/Cl, M = Nd, Am) complexes which show unusually large differences in the M–C_NHC_ bond length between members of the trivalent lanthanide and actinide series with similar ionic radii, and those differences do not significantly reduce as the f-block series is traversed to include Nd^3+^/Am^3+^ complexes.

## Results and Discussion

### Synthesis of [M(Cp*)_2_(I)(THF)] Complexes

**Scheme 1:**
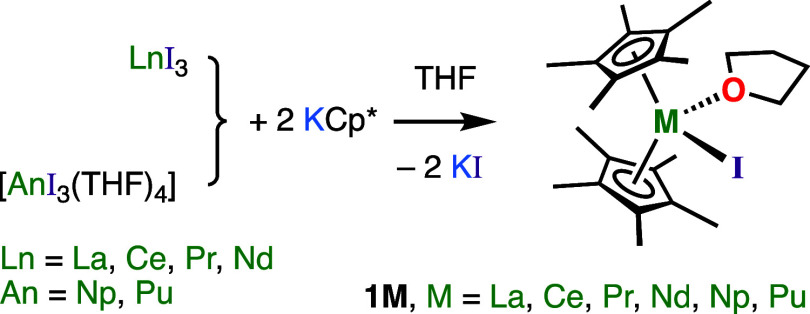
Synthesis of Isolated Crystalline [M(Cp*)_2_(I)(THF)] (**1M**, M = La, Ce, Pr, Nd, Np, and Pu) from Trivalent Iodide Precursors

Lanthanide [Ln(Cp*)_2_(I)(THF)] (**1M**, M = La, Ce, Pr, and Nd) complexes were synthesized on a ca. 150 μmol scale in an argon-filled inert-atmosphere glovebox at room temperature. THF was added to a solid mixture of binary MI_3_ and a slight excess (2.2 equiv) of KCp* in a glass scintillation vial with a Teflon-coated stirrer bar (Scheme 1). Workup and crystallization from warm toluene with a drop of THF (12 mg) gave free-flowing plank-shaped crystals of each complex in poor to fair yield (ca. 30–50%), though the exact % yields depend upon the amount of coordinated THF in each batch of “M(Cp*)_2_(I)(THF)_*m*_” (M = La, Ce, Pr, Nd; *m* = 0 to 1) which remained after drying (see Supporting Information). The corresponding actinide complexes **1Np** and **1Pu** were synthesized using the previously reported procedure, beginning with [AnI_3_(THF)_4_] (An = Np, Pu),^68^ and were used as crude products without recrystallization for subsequent reaction steps.

### Synthesis of [M(Cp*)_2_(I)(I^Me4^)] Complexes

**Scheme 2:**
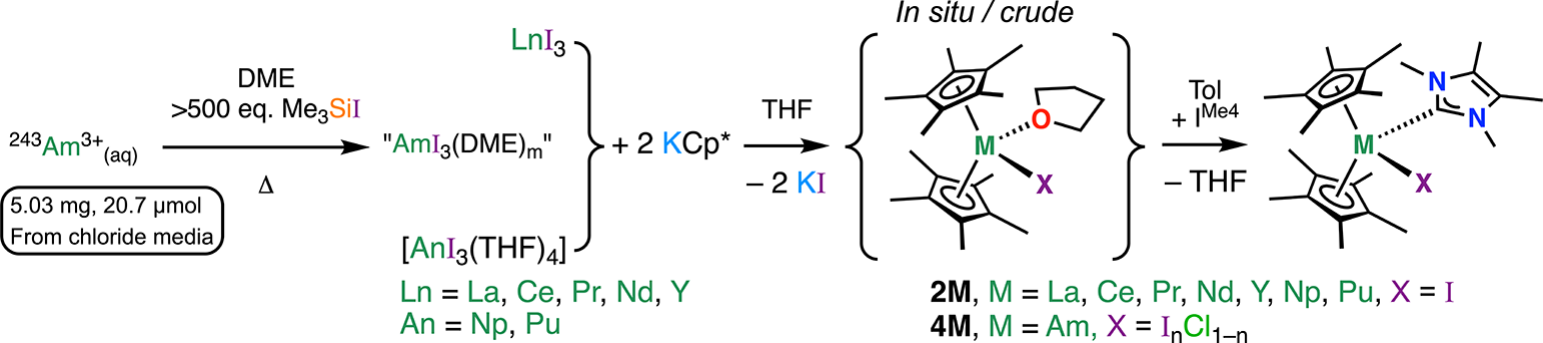
Synthesis of Isolated Crystalline [M(Cp*)_2_(I)(I^Me4^)] (**2M**, M = La, Ce, Pr, Nd, Y, Np, and Pu) from **1M**, [M(Cp*)_2_(I)(THF)], Prepared in Situ I^Me4^ = {C(NMeCMe)_2_}. Note that for M = Am, the X-atom depicted is a mixture of iodide and chloride.

Due to the poor solubility and stability of **1M** (M = La, Ce, Pr, Nd, Np, and Pu) in noncoordinating solvents such as toluene and hexane, we opted to synthesize all [M(Cp*)_2_(I)(I^Me4^)] complexes (**2M**, M = La, Ce, Pr, Nd, Y, Np, Pu, and Am) from **1M** prepared in situ/as crude material (Scheme 2). A slight excess of solid I^Me4^ (1.05 equiv) was added to the stirred solutions of **1M** in toluene. Workup and low-temperature (−35 °C) crystallization from the reaction solvent gave **2M** in modest yield (30–40%) as well-isolated large plank-shaped crystals in all cases. We have included the yttrium compound, **2Y**, here as a smaller member of the series for comparative purposes, but the isolation of both **1Y** and **1Am** was not attempted (vide infra).

Rare earth **2M** compounds (M = La, Ce, Pr, Nd, and Y) were assessed by elemental analysis and found to be analytically pure and in agreement with their [M(Cp*)_2_(I)(I^Me4^)] formulation. In the case of **2M** for Np and Pu, we can only judge their purity by ^1^H and ^13^C{^1^H} NMR spectroscopies (vide infra). For the synthesis of **2Am**, we attempted to generate putative “AmI_3_(DME)_*m*_” in analogy to our previous synthesis of the chloride congener (Scheme 2).^14,81^ The putative “AmI_3_(DME)_*m*_” was used as-synthesized for subsequent reaction steps–see Supporting Information for details. Instead of the anticipated Am^3+^ analogue of the other **2M** complexes, we isolated golden orange plank-shaped crystals of [Am(Cp*)_2_(I_*n*_Cl_1–*n*_)(I^Me4^)] (**4Am**), where *n* is approximately 0.65 as determined independently by both ^1^H NMR spectroscopy and single-crystal X-ray diffraction (vide infra). This suggests that the treatment of the putative “AmCl_3_(DME)_*m*_” precursor with Me_3_Si–I did not result in complete substitution of Cl for I. The corresponding Nd^3+^ complex [Nd(Cp*)_2_(I_*n*_Cl_1–*n*_)(I^Me4^)] (**4Nd**, where *n* is also approximately 0.65) was then synthesized analogously starting from Nd^3+^ in aqueous 6 M HCl solution. Finally, to aid the interpretation of the bonding data in these mixed I/Cl **4M** complexes, [Nd(Cp*)_2_(Cl)(I^Me4^)] (**5Nd**) was synthesized in low yield (24%) over two steps using binary NdCl_3_ as the metal source.

### Molecular Structures

**Figure 2:**
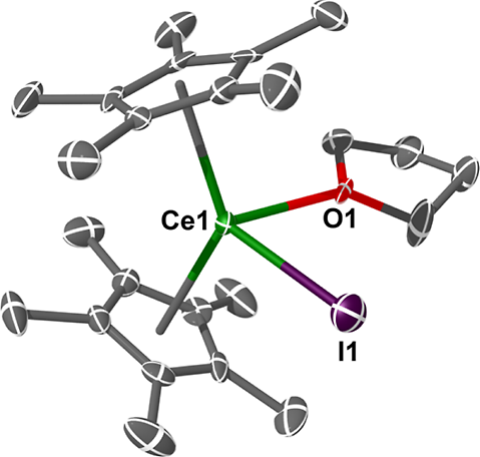
Molecular structure of **1Ce**. Ellipsoids set at 50% probability and H atoms, along with a second unit containing Ce(2), removed for clarity (operations: *X*, *Y*, *Z*). Ce(1)–I(1) = 3.1027(8) Å; Ce(1)–O(1) = 2.511(5) Å; Ce(1)–Cp_cent_ = 2.497(5) Å; Ce(1)–Cp_cent_ = 2.506(4) Å; Cp_cent_–Ce–Cp_cent_ = 136.04(11)°.

Single-crystal X-ray diffraction studies revealed all four new **1M** complexes to be isomorphous to the previously reported **1U**, **1Np**, and **1Pu** (except **1La** for which the *a* axis is doubled in length), and the Sm,^82^ Dy,^83^ and Yb analogues.^84^ All crystallize in the triclinic space group *P*1̅ with two molecules per asymmetric unit (*Z*′ = 2, except **1La** for which *Z*′ = 4)—the structure of **1Ce** is depicted in Figure 2. Detailed discussion of structural metrics for **1M** is provided in the Supporting Information and is summarized in Tables 1 & 2 and Figure 4 to facilitate ready comparison to **2M** complexes (vide infra).

**Table 1:** Bond Lengths (Å) and Angles (deg) for M(1) in **1M** (M = La, Ce, Pr, Nd, U, Np, and Pu)^a^

	**1La**^b^	**1U**(66)	**1Ce**	**1Np**(68)	**1Pr**	**1Pu**(68)	**1Nd**
M–I	3.1400(6)	3.0636(6)	3.1027(8)	3.0560(14)	3.0851(8)	3.0353(7)	3.0618(10)
M–O	2.536(4)	2.496(4)	2.511(5)	2.504(13)	2.491(5)	2.460(5)	2.461(8)
M–Cp_cent_	2.562(4)	2.476(4)	2.497(5)	2.530(5)	2.473(5)	2.455(6)	2.467(6)
	2.542(16)	2.494(4)	2.521(4)	2.561(5)	2.500(5)	2.463(5)	2.471(7)
Cp_cent_–M–Cp_cent_	138.08(17)	135.56(9)	136.04(11)	135.12(11)	135.92(12)	135.49(13)	135.48(16)

a Due to different numbering conventions across some examples, “M(1)” is denoted here as that with the shortest M–I distance, and numbering proceeds from there. Except for **1La**, all are isomorphous. However, **1La** is isostructural.

b Only the largest component of the disordered La–Cp rings is listed.

**Table 2:** Comparison of M–I and M–O Bond Lengths (Å) between **1M** (M = La, Ce, Pr, U, Np, and Pu) Complexes

	M–I		M–O	
	M(1)	M(2)	M(1)	M(2)
**1La**	3.1400(6)	3.1492(5)	2.536(4)	2.531(4)
**1U**	3.0636(6)	3.0955(6)	2.496(4)	2.486(4)
Δ(La–U)	*0.0764(8)*	*0.0537(8)*	*0.040(6)*	*0.045(6)*
**1Ce**	3.1027(8)	3.1272(7)	2.511(5)	2.507(4)
**1Np**	3.0560(14)	3.0832(12)	2.504(13)	2.472(12)
Δ(Ce–Np)	*0.0467(8)*	*0.0440(7)*	*0.007(6)*	*0.035(6)*
**1Pr**	3.0851(8)	3.1070(8)	2.491(5)	2.499(5)
**1Pu**	3.0353(7)	3.0594(6)	2.460(5)	2.463(6)
Δ(Pr–Pu)	*0.0498(1)*	*0.0476(1)*	*0.031(6)*	*0.036(6)*
Δ(Ce–Pu)	*0.0674(11)*	*0.0678(9)*	*0.051(7)*	*0.044(7)*

**Figure 3:**
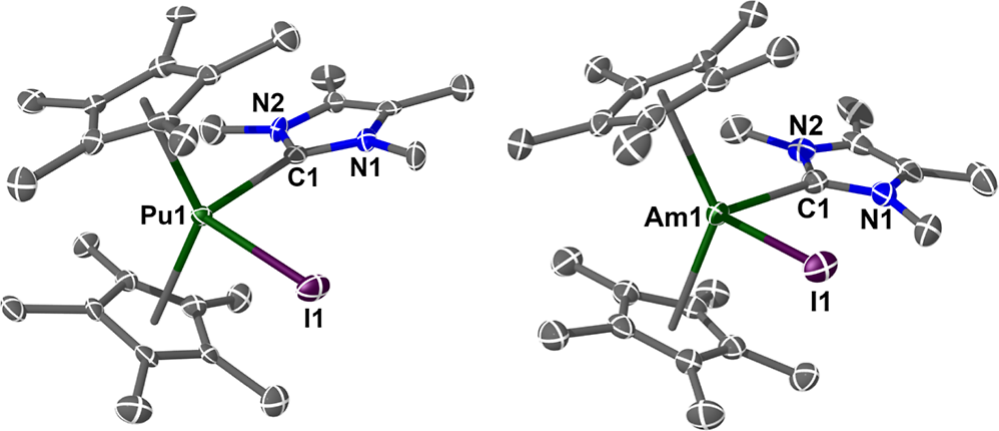
Molecular structure of **2Pu** and **4Am**. Ellipsoids set at 50% probability and H atoms removed for clarity (operations: *X*, *Y*, *Z*). In **4Am**, Cl(1) has been removed for clarity and the I(1) occupancy (0.65) has been refined competitively against Cl(1) (0.35). Pu(1)–I(1) = 3.0938(7) Å; Pu(1)–C(1) = 2.637(8) Å; Pu(1)–Cp_cent_ = 2.489(4) Å; Pu(1)–Cp_cent_ = 2.510(4) Å; Cp_cent_–Pu(1)–Cp_cent_ = 135.47(13)°. Am(1)–I(1) = 3.0666(11) Å; Am(1)–C(1) = 2.631(3) Å; Am(1)–Cp_cent_ = 2.4744(17) Å; Am(1)–Cp_cent_ = 2.4895(15) Å; Am(1)–Cl(1) = 2.682(8) Å; and Cp_cent_–Am–Cp_cent_ = 135.64(6)°.

The NHC-ligated complexes **2M** (M = La, Ce, Pr, Nd, Y, Np, and Pu) and [M(Cp*)_2_(I_*x*_Cl_1–*x*_)(I^Me4^)] (M = Nd, **4Nd**; Am, **4Am**) isolated herein are isomorphous with the previously reported **2U** and **2Ce** analogues,^51^ crystallizing in the monoclinic space group *P*2_1_/*c* with one molecule per asymmetric unit. In the case of **2La** and **2Ce** an additional polymorph was identified (see Supporting Information Tables S14 and S15 for more details). The structures of **2Pu** and **4Am** are shown in Figure 3. These complexes display a pseudo-tetrahedral bent-metallocene structure with a staggered Cp*···Cp* arrangement, like **1M**, as would be expected for a simple replacement of the THF moiety with the I^Me4^ NHC. Crystallographically characterized Pu–NHC complexes, and Pu–C σ-bonds in general, remain extremely scarce;^50,67,69,85^ also, to the best of our knowledge, **4Am** represents the first structural authentication of an Am–C σ-bonding interaction to any organic ligand.

To investigate the influence of the mixed Cl/I occupancy in **4Am** on the overall structure, the Nd-congener, **4Nd**, was structurally characterized as well as the pure chloride Nd-analogue, [Nd(Cp*)_2_(Cl)(I^Me4^)] (**5Nd**). The Cp_cent_–M–Cp_cent_ angles for all **2M** lie over an exceptionally narrow range of ca. 135.20(8)–135.89(6)° (135.61(9)° using the mean and a weighted standard deviation as the error).^48^ The M–Cp_cent_ ranges in **2M** [e.g., 2.5576(17)–2.5708(15) Å for **2La** and 2.4941(13)–2.5081(12) Å for **2Nd**] are nevertheless very close and on the order of only ca. 0.03 Å longer than **1M**, while statistically distinct from those in the corresponding **1M** complexes. The Cp_cent_–M–Cp_cent_ angles for **4Nd** (135.18(11)°) and **4Am** (135.64(6)°) are very close or actually overlap with the average for all others (including **2Y**) at 135.61(9)°.^48,51^ Similarly, both display M–C_Cp_ ranges (**4Nd**, 2.741(7)–2.816(7) Å; **4Am** 2.718(3)–2.802(3) Å), which follow smoothly from the previous members of their respective series, where the small decrease in length is commensurate with the reduction in ionic radius. Tables 3 and 4 summarize pertinent lengths and angles within the **2M** series and **4Am**/**4Nd**; see the Supporting Information for a comparison of **2La** and **2Ce** to their polymorphs (**2La**^**β**^ and **2Ce**^**β**^).

**Table 3:** Experimental and (in Italics) Computed (Vide Infra) Bond Lengths (Å) and Angles (deg) for **2M** (M = La, Ce, Pr, Nd, Y, U, Np, and Pu), and **4M** (M = Nd, Am)

	**2La**	**2U**(51)	**2Ce**^a^	**2Np**	**2Pr**	**2Pu**	**2Nd**	**4Nd**	**4Am**	**2Y**
M–I	3.1769(3)	3.1266(4)	3.1584(6)	3.1003(7)	3.1393(6)	3.0938(7)	3.1209(2)	3.103(2)	3.0666(11)	3.0409(4)
	*3.140*	*3.095*	*3.118*	*3.081*	*3.100*	*3.074*	*3.085*	–b	–b	
M–C_NHC_	2.751(4)	2.687(5)	2.724(7)	2.670(9)	2.700(7)	2.637(8)	2.693(3)	2.683(7)	2.631(3)	2.583(4)
	*2.754*	*2.667*	*2.720*	*2.665*	*2.698*	*2.643*	*2.690*	–b	–b	
M–Cp_cent_	2.5590(17)	2.514(3)	2.534(4)	2.495(4)	2.511(3)	2.489(4)	2.4941(13)	2.500(4)	2.4744(17)	2.3967(15)
	2.5723(15)	2.532(2)	2.547(3)	2.518(4)	2.529(3)	2.510(4)	2.5081(12)	2.511(3)	2.4895(15)	2.4126(15)
Cp_cent_–M–Cp_cent_	135.45(5)	135.20(8)	135.92(11)	134.82(14)	135.95(11)	135.47(13)	135.30(4)	135.18(11)	135.64(6)	135.89(6)

a **2Ce** has been reported previously;^51^ however, we have resynthesized it here and used values from our single-crystal X-ray diffraction study. Mean absolute deviation between the experiment and calculation = 0.032 Å for M–I and 0.006 Å for M–C.

b Geometry optimization was not performed on the **2Am** component of **4Am** due to the unavailability of dispersion corrections for Am,^86^ and the **2Nd** component of **4Nd** was not optimized as **2Nd** has been geometry-optimized separately starting from coordinates from crystals of the pure compound.

**Table 4:** Comparison of Experimental and Computed M–I and M–C_NHC_ Bond Lengths (Å) between **2M** (M = La, Ce, Pr, U, Np, and Pu) and 4M (M = Nd, Am) Complexes

	M–I		M–C_NHC_	
	Expt.	Comp.	Expt.	Comp.
**2La**	3.1769(3)	3.140	2.751(4)	2.754
**2U**	3.1266(4)	3.095	2.687(5)	2.667
Δ(La–U)	*0.0503(5)*	*0.045*	*0.064(6)*	*0.087*
**2Ce**	3.1584(6)	3.118	2.724(7)	2.720
**2Np**	3.1003(7)	3.081	2.670(9)	2.665
Δ(Ce–Np)	*0.0581(9)*	*0.037*	*0.054(11)*	*0.055*
**2Pr**	3.1393(6)	3.100	2.700(7)	2.698
**2Pu**	3.0938(7)	3.074	2.637(8)	2.643
Δ(Pr–Pu)	*0.0455(9)*	*0.026*	*0.063(11)*	*0.055*
Δ(Ce–Pu)	*0.0646(9)*	*0.044*	*0.087(11)*	*0.077*
**2Nd**	3.1209(2)	3.085	2.693(3)	2.690
**4Am**	3.0666(11)	–b	2.631(3)	–b
Δ(Nd–Am)^a^	*0.0543(11)*	–b	*0.062(4)*	–b
**4Nd**	3.103(2)	–b	2.683(7)	–b
Δ(Nd–Am)^a^	*0.0364(20)*	–b	*0.052(8)*	–b

a Entries compare **4Am** to **2Nd**, or **4Nd**, respectively.

b Geometry optimization was not performed on the **2Am** component of **4Am** due to the unavailability of dispersion corrections for Am,^86^ and the **2Nd** component of **4Nd** was not optimized as **2Nd** has been geometry-optimized separately starting from coordinates from crystals of the pure compound.

Complex **5Nd** crystallized in the triclinic space group *P*1̅ with *Z*′ = 2, unlike both **2M** (M = La, Ce, Pr, Nd, Y, U, Np, and Pu) and **4M** (M = Nd, Am), which suggests that beyond a certain chloride occupancy the bulk lattice changes. However, all pertinent bond lengths and angles for **5Nd** (e.g., Nd–C_Cp_ range = 2.723(3)–2.85(3) Å; Nd–C_NHC_ = 2.708(3) Å; Cp_cent_–Nd–Cp_cent_ = 134.3(4)° and 136.91(8)°) are very similar to both **2Nd** and **4Nd**. Between **2Nd**, **4Nd**, and **5Nd**, all metrical parameters overlap within the 3σ criterion with the exception of the Nd–I lengths (3.1209(2) Å in **2Nd**, and 3.103(2) Å in **4Nd**). This suggested that at least for Nd, within this molecular framework, there is a minimal perturbation to the rest of the molecular structure due to the influence of iodide (**2Nd**) vs chloride (**5Nd**)—though packing forces differ. Importantly, across all three structures with Nd, the Nd–C_NHC_ bond lengths are within 3σ of each other except for one of the Nd–C_NHC_ distances in **5Nd** (there are two independent molecules in the asymmetric unit); **2Nd**, 2.693(3) Å; **4Nd**, 2.683(7) Å; and **5Nd**, 2.667(3) Å and 2.708(3) Å. Thus, we suggest that it is possible to draw useful comparisons with other mixed-halide structures such as **4Am**—noting that M–X (X = I or Cl) bond lengths are likely to be somewhat less reliable than those from a data set which does not contain mixed occupancy atoms.

**Figure 4:**
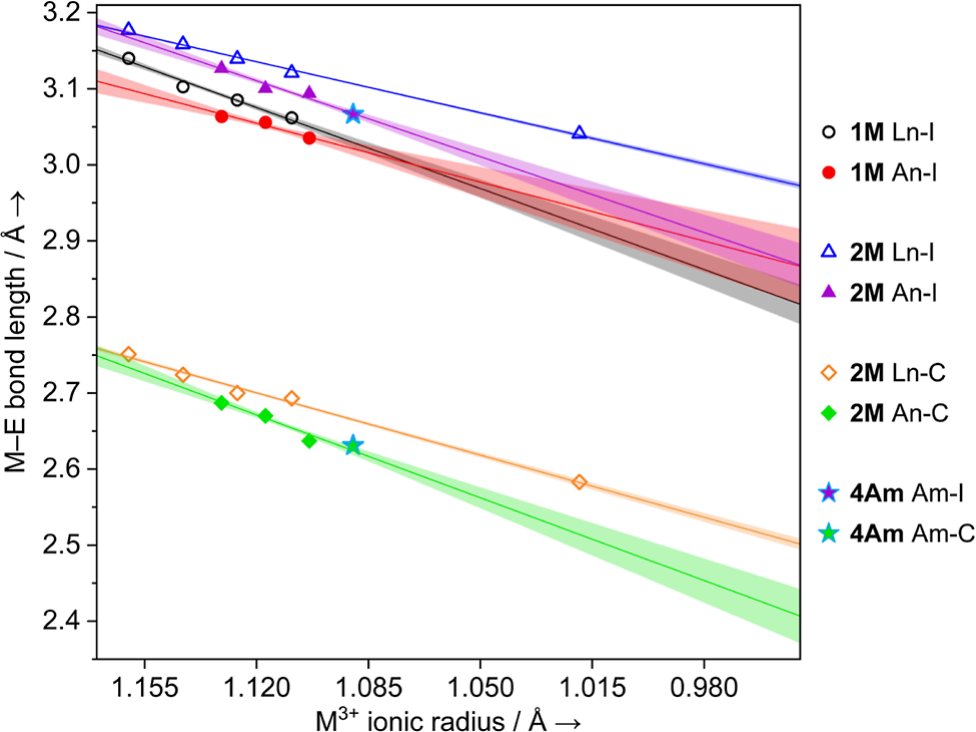
M^3+^ ionic radius (calculated 8-coordinate)^14^ vs the M–E bond length for M–I with both **1M** and **2M**, and M–C_NHC_ for **2M** and **4Am**. Open symbols are used for Ln complexes (La, Ce, Pr, Nd, and Y from left to right), and solid symbols are used for An complexes (U, Np, Pu, and Am from left to right). Solid lines denote a linear fit of the data points (see Supporting Information for statistical parameters), and shaded areas show the 50% confidence interval for extrapolated data. For the **1M** series, only metal site (1) is shown here, which corresponds in all cases to the shorter M–I length, to minimize the differences and not overstate any conclusions. See Supporting Information for additional data plots.

The M–C_NHC_ bond lengths within the **2M** series provide the most striking comparison. For example, with **2La** and **2U,** the difference in the M–C_NHC_ bond (Δ = 0.064(6) Å) is similar to the difference between **2Pu** (M–C_NHC_ = 2.637(8) Å) and either **2Ce** (M–C_NHC_ = 2.724(7) Å; Δ = 0.087(11) Å) or **2Pr** (M–C_NHC_ = 2.700(7) Å; Δ = 0.063(11) Å). Crucially, the difference in M–C_NHC_ lengths between the lanthanide and actinide series does not diminish significantly from U to Pu (La to Pr). By comparison, as Tables 2 and 3 show, the magnitude of the difference between lanthanide and actinide M–I bond lengths broadly decreases along both **1M** and **2M** series, which is the normal trend.^18,20,47,65,87−90^ In **4Am,** we see that the M–C_NHC_ length (2.631(3) Å) is shorter than all of the **2M** (M = La, Ce, Pr, Nd, U, Np, and Pu) complexes except that of **2Y**. When comparing **4Am** to all of **4Nd**, **2Nd**, and **5Nd** (8-coordinate ionic radii: Am^3+^ = 1.09 Å, Nd^3+^ = 1.109 Å; Δ = 0.019 Å), we find that none of the Nd-complexes has any overlap of their Nd–C_NHC_ lengths with the Am–C_NHC_ bond in **4Am**, even to a 5σ level of significance. For example, the difference in the M–C_NHC_ bond between **4Am** and **2Nd** is 0.062(3) Å (with **2Nd** bearing the longer bond length), and for **4Nd,** it is 0.082(8) Å, again where Nd bears the longer distance. The smallest difference between **4Am** and any of the Nd^3+^ complexes we observe is when comparing Nd(1) of **5Nd** with **4Am** (Δ = 0.036(4) Å)—again this difference is larger than the difference in their ionic radii even when accounting for the standard uncertainty in the measurements. To the best of our knowledge, this is unprecedented in studies of molecular systems with trivalent f-elements that span across La/U, Ce/Np, Pr/Pu, and Nd/Am comparisons. Figure 4 shows a plot of the calculated 8-coordinate ionic radius vs the M–E bond length for M–I in **1M**, and both M–I and M–C_NHC_ in **2M**.^14,91^ See Figure S44 in the Supporting Information for the same data plotted against the experimentally derived 6-coordinate ionic radii, which shows the same trend.

In assigning the coordination numbers of complexes herein, we have counted Cp as three sites;^92^ therefore, the metals are formally 8-coordinate, and so values for 8-coordinate ionic radii are used.^14,91^ This is to avoid overstating the significance of bond length differences, which can manifest when 6-coordinate radii are used with complexes which are not formally 6-coordinate. When comparing isomorphous Am and Nd complexes with formal coordination numbers (CN) greater than 6–which is every molecular Am complex to have been structurally characterized except [Am{N(O=PPh_2_)_2_}_3_] (6 CN),^14^ [AmBr_3_(OPCy_3_)_3_] (6 CN),^89^ and [AmCl_6_][PPh_4_]_3_ (6 CN)^64^ – one should account for the 0.019 Å difference between 8-coordinate Am^3+^ and Nd^3+^. When Am-ligand bond lengths are shorter than Nd-ligand bond lengths by >0.019 Å, they are more likely to be of significance beyond simple well-established ionic bonding trends as opposed to shortening, which lies between 0.008 and 0.019 Å.

It is important to place the metrical data in context with previous Am/Nd comparisons in the literature, which reflects the scarcity of clear, unambiguous cases where Am^3+^-ligand bond lengths are shorter than Nd^3+^-ligand bond lengths. By way of example, the M–Se bond lengths in a series of 9-coordinate complexes, [M{N(E=PPh_2_)_2_}_3_] (M = La, Ce, Nd, U, Np, Pu, and Am; E = Se),^14,68^ show that the An–Se length is shorter than the Ln–Se length for U/La and Pu/Ce pairs by 0.0360(5) and 0.0303(4) Å, but upon reaching Am/Nd, the difference dropped to 0.0176(9) Å which is on the edge of the difference in the 8-coordinate ionic radii of Am^3+^/Nd^3+^ (Δ = 0.019 Å), while much larger than the difference in 6-coordinate ionic radii for these metals (Δ = 0.008 Å)—noting of course that these complexes are 9-coordinate.^91^ In [M(Cp^tet^)_3_] [M = Am, Nd; Cp^tet^ = (C_5_Me_4_H)], the Am–Cp_centroid_ distance is 2.517(8) Å and 2.518(1) Å for the Nd–Cp_centroid_ distance, meaning that they are statistically identical.^81^ In [{M(Cp′)_3_}_2_(μ-4,4′-bipy)] (M = Am, Nd; Cp′ = {C_5_H_4_(SiMe_3_)}; bipy = bipyridine),^18^ the average Am and Nd M–Cp_centroid_ distances to the Cp′ ligand are 2.524(3) and 2.543(2) Å, respectively, (Δ = 0.019(4) Å)—again on the edge of significance when the differences in 8-coordinate ionic radius is considered.^92^ The difference in the metal–nitrogen distances is slightly more pronounced in the [{M(Cp′)_3_}_2_(μ-4,4′-bipy)] complexes, where the Am–N bond (2.618(3) Å) is shorter than the Nd–N bond (2.6482(16) Å) by 0.030(3) Å.^18^ In a study of f-block dithiocarbamate complexes, [M(S_2_CNEt_2_)_3_(N_2_C_12_H_8_)] (M = Nd, Sm, Eu, Gd, Dy, Am, Cm, and Cf), the average Am–N and Am–S distances were found to overlap within 3σ of each other once ionic radius differences were considered.^46^ Similarly, inconclusive signs of Am vs Nd bonding differences were found in a pair of dithiophosphinate complexes, [M{S_2_P(^*t*^Bu_2_C_12_H_4_)}_4_]^−^ (M = Am, Nd), which feature ligands directly relevant to S-donor extractant molecules that show selectivity for the minor actinide ions Am^3+^ and Cm^3+^, over Ln^3+^, in biphasic solvent extractions.^14,16,68,88^ Thus, the substantially shorter Am–C_NHC_ length in **4Am** compared with the isomorphous **4Nd** congener is unusual and warrants further spectroscopic/computational investigation to provide insight into the origin of the observed metrical differences.

### Quantum Chemical Calculations

To help elucidate the nature of the bonding and electronic structure differences in complexes **2M**, we turned to computational quantum chemistry in the form of scalar relativistic, hybrid density functional theory at the PBE0 level. Full details of the calculations are given in the Supporting Information. We began by optimizing the geometries of **2M** (M = La, Ce, Pr, Nd, U, Np, and Pu); M–I and M–C_NHC_ distances are collected in Tables 3 and 4. There is very good agreement in these metrics between experiment and calculation, especially for M–C_NHC_ lengths, for which the mean absolute deviation is only 0.006 Å. As with the single-crystal X-ray diffraction data, the difference between the M–I distances in corresponding pairs of lanthanides and actinides decreases systematically across the series, while the analogous difference in M–C_NHC_ distances does not, with that of the Pr/Pu pair being the same as that for Ce/Np, Δ = 0.055 Å. Note that due to the unavailability of dispersion corrections for Am,^86^ the structure of **2Am** was not optimized and was taken directly from the single-crystal X-ray diffraction geometry (that is, the iodo-component of **4Am**).

**Figure 5:**
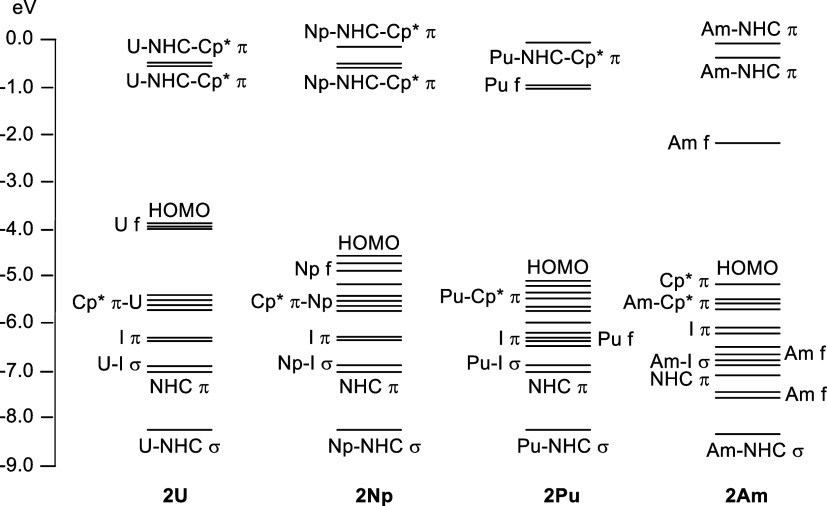
Molecular orbital energy level diagrams for **2An** (An = U, Np, Pu, and Am), obtained from the optimized geometries of **2U**, **2Np**, and **2Pu**, and from a single point calculation at the single-crystal X-ray diffraction geometry of the **2Am** component of **4Am** (due to unavailability of dispersion corrections for Am).^86^ Principal orbital character is indicated, though sometimes the indicated character is present in other, energetically close, orbitals. HOMO = highest occupied molecular orbital.

We initially anticipated that natural bond orbital (NBO) analysis would allow us to address the nature of the M–C_NHC_ interaction within a localized orbital framework, but NBO did not locate any M–C_NHC_ bonding orbitals. Therefore, we turned to an analysis of the Kohn–Sham orbitals, recognizing that these are typically rather delocalized in large, low-symmetry f-element organometallic compounds. Valence molecular orbital (MO) energy level diagrams are presented for **2An** (An = U, Np, Pu, Am) in Figure 5, with analogous diagrams for **2Ln** given in the Supporting Information (Figure S96). The energies of the An–Cp* and An–I orbitals change little across the series, in contrast to the metal 5f manifold, which shows the expected significant decrease in energy from U to Am. The NHC-based MOs–distinguished as σ and π according to their principal character about the M···C axis–are the most stable of those shown, except for **2Am**, for which two metal 5f-based orbitals lie in between the NHC-based levels (also the case for **2Nd**, see Figure S96).

**Figure 6:**
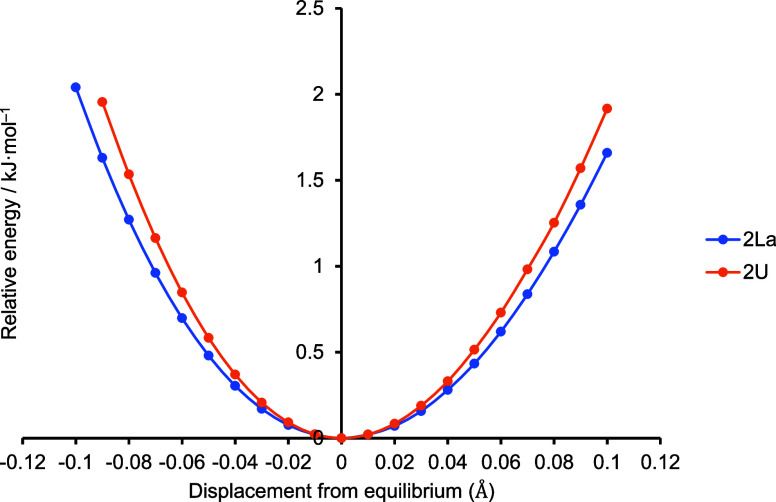
Energy relative to that at the fully optimized geometry for changing the M–C_NHC_ distance in **2La** and **2U**, while allowing the rest of the atomic positions to relax.

Before exploring the composition of the NHC-based σ and π MOs, it is instructive to examine the total energy surface for scanning the M–C_NHC_ distance. Figure 6 shows the change in the total (SCF) energy of **2La** and **2U** upon performing relaxed scans of this distance. Clearly, these energy surfaces are very flat; compressing or elongating the bonds by 0.05 Å, an amount similar to the Ln vs An bond length differences seen in the **2M** series, costs only ca. 0.5 kJ·mol^–1^, suggesting that quantum chemical differences in M–C_NHC_ bonding between Ln/An pairs are likely small. The energy penalties for the M–C_NHC_ bond length changes are within the range of crystal packing forces, but the consistent shortening of An–C_NHC_ relative to Ln–C_NHC_ within this series, to a degree which is not commensurate with the difference in ionic radius, suggests that the crystal packing forces are not the main cause. It is notable that the energy well for **2U** is slightly steeper than that for **2La**, suggesting that the U–C_NHC_ bond is the stronger. To further estimate this, we split both **2U** and **2La** into two fragments, the NHC ligand and the Cp_2_*MI component at their geometries in **2U** and **2La**, and calculated the energies of the separated fragments vs those of the full molecules. This yields fragmentation energies of 192.6 and 174.4 kJ·mol^–1^ for **2U** and **2La**, respectively, at the SCF level.

Table 5 presents Mulliken population analysis data for the NHC-based σ and π MOs, together with the M–C delocalization indices δ, calculated from the quantum theory of atoms in molecules (QTAIM). δ is often taken as a QTAIM measure of bond order. None of the δ values are large, indicating that the M–C_NHC_ interactions are not strong, in agreement with Figure 6. The δ values display a striking similarity among the lanthanides, and within the actinide series. The latter have slightly higher δ than the former, suggestive of a consistently larger M–C_NHC_ interaction in the 5f series, in agreement with the experimental and computational structural data.

**Table 5:** Kohn–Sham Molecular Orbital Compositions (Mulliken Analysis, 1% Threshold) and QTAIM Delocalization Indices (δ) for **2M** (M = La, Ce, Pr, Nd, U, Np, Pu, and Am)^a^

	**2La**	**2U**	**2Ce**	**2Np**	**2Pr**	**2Pu**	**2Nd**	**2Am**
metal content of M–NHC σ (%)	2.0s, 1.8p, 6.8d	1.9s, 1.0p, 7.7d, 1.4f (s + p + d = 10.6)	1.9s, 1.9p, 7.2d	1.8s, 1.1p, 7.4d, 2.4f (s + p + d = 10.3)	1.9s, 1.7p, 7.0d, 1.4f (s + p + d = 10.7)	1.8s, 1.2p, 7.4d, 3.3f (s + p + d = 10.6)	b	1.1s, 5.4d, 6.7f (s + p + d = 6.5)
total metal content (%)	10.6	12.0	11.0	12.7	12.1	13.8		13.2
metal content of M–NHC π (%)		1.3d		1.2d	b	1.2d, 3.4f		1.2d, 1.4f
M–C_NHC_ δ	0.30	0.38	0.31	0.38	0.31	0.38	0.31	0.37

a Data obtained from the optimized geometries of all **2M** bar **2Am**, for which a single point calculation at the single-crystal X-ray diffraction geometry was performed (due to non-availability of dispersion corrections for Am).

b Extensive mixing of ligand orbitals with metal 4f levels (see Supporting Information Figure S96) precludes clear population analysis.

Turning to the population analysis of the NHC-based σ and π MOs, we begin with the former. It is noteworthy that, within the first three Ln/An pairs, the sum of the metal s, p, and d contributions to these orbitals is very similar, all lying within the range 10.3–11.0%. For the actinide member of each pair, there is also a 5f contribution, which rises from 1.4% for **2U** to 3.3% for **2Pu** (noting also the 1.4% 4f contribution for **2Pr**). **2Am** differs from the other actinides in having reduced s + p + d and larger 5f (6.7%) composition, though it has a total metal contribution very similar to **2Np** and **2Pu**. It is tempting to ascribe the shorter An–C_NHC_ distances vs their Ln analogues to the extra f content of the σ MO, but while recognizing that we cannot rule this out as a contributory factor, we sound the following cautionary note: Bursten’s FEUDAL (f’s essentially unaffected, d’s accommodate ligands) model of the bonding in early actinide complexes tells us that, in general, it is the metals’ d-orbitals that are primarily responsible for metal–ligand binding, not the 5f. Furthermore, it is well known that periodic increases in metal f contributions to MOs featuring both metal and ligand content typically arise from atomic orbital energy matching, rather than reflecting overlap-driven covalency. We note that the M–C_NHC_ distances in **2Pu** and **2Am** are almost identical, despite the Am 5f contribution to the σ MO being twice that in **2Pu**.

**Figure 7:**
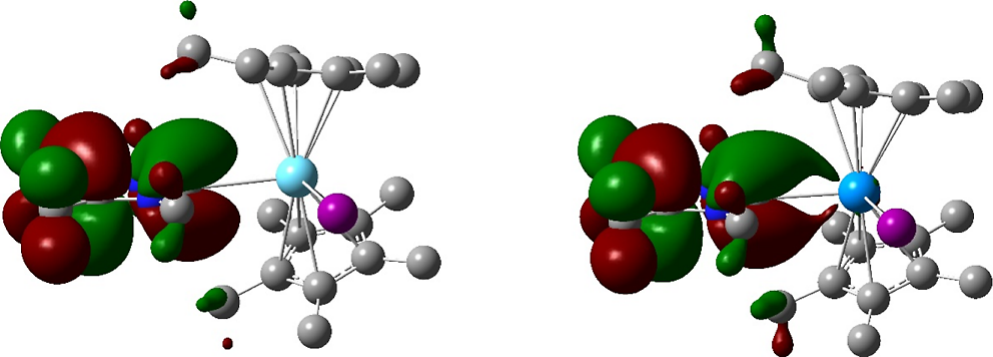
NHC-based π molecular orbital in **2La** (left) and **2U** (right), isovalue = 0.02. Hydrogen atoms omitted for clarity.

The structural data suggest that we are looking for a consistent difference in the An–C_NHC_ versus Ln–C_NHC_ bonding, a conclusion supported by the δ values in Table 5. Figure 6 tells us that such a difference is likely small, and the FEUDAL approach directs us to the 6d orbitals.^93−97^ It is therefore noticeable that all of the actinide complexes have a very small but consistent metal d contribution to the NHC-based π MOs (Table 5), which is absent in all of the lanthanide systems. Complexes **2Pu** and **2Am** also have small 5f contributions to this MO, but note the arguments above about the nature of 5f-based covalency in this part of the actinide series. Figure 7 presents images of the NHC-based π MOs in **2La** and **2U**; the metal contribution to the latter is clearly visible.

**Figure 8:**
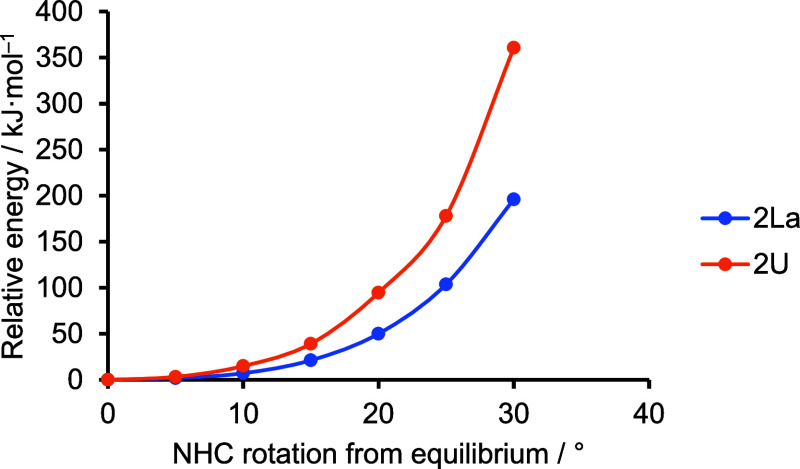
Energy relative to that at the fully optimized geometry for rotating the NHC ligand about the *M*–*C* axis in **2La** and **2U**, while keeping the rest of the atomic positions fixed.

If there is An–C_NHC_ bonding with a π-bonding component as described above, we would expect that rotating the NHC ligand about the M–C_NHC_ axis would be energetically more costly in the An vs Ln systems. We therefore attempted relaxed total energy surface scans for this distortion in **2La** and **2U**, but these calculations were not well-behaved, and indeed, the NMR spectroscopic data (vide infra) suggest that rotation about the M–NHC bond is a high-energy process, requiring significant structural rearrangement. Instead, Figure 8 presents the energies relative to that of the fully optimized geometry for rotating the NHC ligand while keeping all the other atomic positions fixed. Such an approach yields relative energies larger than in relaxed energy surface scans but typically provides an upper bound to the energetics. Clearly, from the ^1^H NMR data of all **2M** complexes, there is a penalty for rotating the NHC ligand about the metal as evidenced by the inequivalence of the Me-resonances at room temperature. This is likely to be predominantly steric in origin as it is seen even for **2La**. However, computationally, the energy penalty for rotating the NHC ligand in **2U** is significantly larger than in **2La**. This may, in part, reflect the ca. 0.1 Å shorter U–C_NHC_ distance vs the La equivalent but may also result in part from the differing M–NHC orbital character shown in Figure 7.

QTAIM bond critical point (BCP) ellipticities ε can give information on single vs multiple bonding; values close to zero indicate cylindrical symmetry about the BCP (single or triple bonds), while significant deviations from zero suggest (partial) double bonding. In our experience, f-element–ligand BCP electron densities are typically very low, which can lead to highly variable curvatures (and hence curvature ratios, which define ε). The BCP electron densities ρ in our target systems are indeed small; those in **2Ln** range from 0.042 electron·bohr^–3^ (for **2La**) to 0.045 for **2Pr** and **2Nd**, with those for **2An** being slightly larger, at 0.051 for **2U**, rising to 0.052 for **2Pu** and **2Am**. Such low values do indeed lead to some scatter in the ε values, but, in general, these are larger for the actinide–NHC bonds than for the lanthanide analogues, the clearest separation being 0.261/0.023 for **2Np** vs **2Ce** and 0.283/0.091 for **2Pu** vs **2Pr**. Lastly, we note that during the revision stage of this manuscript, a report was published that documented a plutonium complex with a Pu–C_NHC_ interaction, complementing earlier analogous neptunium chemistry.^25,98^ That research was focused on the characterization of Pu=C multiply bonded interactions through the coordination of diphosphonioalkylidene ({C(PPh_2_=NSiMe_3_)_2_}, BIPM) ligands to the actinide metal ion. One of those complexes also contained coordinated I^Me4^ NHC ligands, for which shorter Np–C_NHC_ vs Ce–C_NHC_ and Pu–C_NHC_ vs Pr–C_NHC_ bonds were observed with differences on the order of ∼0.045–0.060 Å. Consistent with this study, the bonding was found to be largely electrostatic in nature, but in those works, no differences in molecular orbital compositions related to the metal–NHC bonding were found to correlate with the bond metrics, nor were La/U and Nd/Am comparisons possible.

### NMR Spectroscopy

To further support the characterization of the complexes herein, NMR spectra were collected for **1M** (M = La, Ce, Pr, Nd) in d_6_-benzene with the addition of a weighed amount of H_8_-THF to prevent precipitation of the insoluble material. We were able to observe the C_5_Me_5_ singlet for all four molecules by ^1^H NMR (see Supporting Information for full details). Room temperature solution magnetic susceptibilities were determined for **1Ce**, **1Pr**, and **1Nd** by the Evans method, and they agreed well with the free-ion values: **1Ce** (^2^F_5/2_, measured: 2.47 μ_B_ vs 2.54 μ_B_ expected), **1Pr** (^3^H_4_, measured: 3.48 μ_B_ vs 3.58 μ_B_ expected), and **1Nd** (^4^I_9/2_, measured: 3.58 μ_B_ vs 3.62 μ_B_ expected).

Well-resolved ^1^H and ^13^C{^1^H} NMR spectra were also collected all **2M** complexes (except previously reported **2U**), as well as **4Am**, in neat d_6_-benzene (see Supporting Information for ^13^C{^1^H} NMR spectroscopy and all data). As previously observed with **2Ce** and **2U**,^51^ all **2M** complexes showed that rotation about the M–C_NHC_ bond is restricted. A variable temperature NMR (VT-NMR) of **2La** showed that even at 100 °C in d_8_-toluene, the La–C_NHC_ bond is restricted (Figures S57–S59). As La^3+^ is the largest ion studied herein and **2La** possesses the longest M–C_NHC_ bond length, the calculated barrier to rotation in this complex represents a lower bound for this series of complexes. Nevertheless, it is highly disfavored as Δ*S*^⧧^ = −11.6 J·K^–1^ mol^–1^ (−61.8 J·K^–1^ mol^–1^ to 38.5 J·K^–1^ mol^–1^) and Δ*H*^⧧^ = 92.3 kJ·mol^–1^ (74.6 kJ·mol^–1^ to 110.0 kJ·mol^–1^), where the values in brackets are the 95% confidence intervals. Presumably, the large barrier arises through a combination of predominantly steric effects, though, in the case of the actinide complexes, there may also be a contribution from the M–C_NHC_ π-bonding component which is supported by calculations (Figure 8). Table 6 shows the ^1^H NMR chemical shifts for the Cp* and I^Me4^ ligands of all **2M** complexes.

**Table 6:** ^1^H NMR Chemical Shifts (ppm vs d_6_-Benzene Residual) for **2M** (M = La, Ce, Pr, Nd, Y, U, Np, and Pu). Spectra Were Recorded at Ambient Temperature (295–298 K)

	**2La**	**2U**^a^(51)	**2Ce**	**2Np**	**2Pr**	**2Pu**	**2Nd**	**2Y**
C_5_Me_5_	2.17	0.68	6.10	0.77	13.61	1.65	11.82	2.09
I^Me4^ C(CH_3_)	1.21	–53.99	–23.68	0.18	–70.81	1.25	–33.77	1.21
	1.36	–46.82	–19.34	0.54	–47.13	1.25	–25.74	1.35
I^Me4^ N(CH_3_)	2.99	–11.19	–3.52	2.64	–13.10	3.94	–5.43	2.91
	3.54	–10.49	–3.23	4.27	–10.59	4.98	–4.70	3.66

a **2U** was reported previously in d_8_-THF.

The ^1^H NMR spectrum of **4Am** in d_6_-benzene shows features similar to all the **2M** complexes but with an apparent doubling of every signal (see Figures S64–S67). A variable temperature NMR spectroscopic (VT-NMR) study in d_6_-benzene (Figure S66), across a small temperature range due to radiological safety considerations, revealed that up to 50 °C, the peaks did not coalesce, nor did their relative ratios change. We attribute the doubling to the presence of a mixed halide species, [Am(Cp*)_2_(I_n_Cl_1–n_)(I^Me4^)], which informed and is in excellent agreement with our crystallographic study (where *n* is ca. 0.65 determined by competitive refinement of the halide sites).

### UV–Vis–NIR Spectroscopy

**Figure 9:**
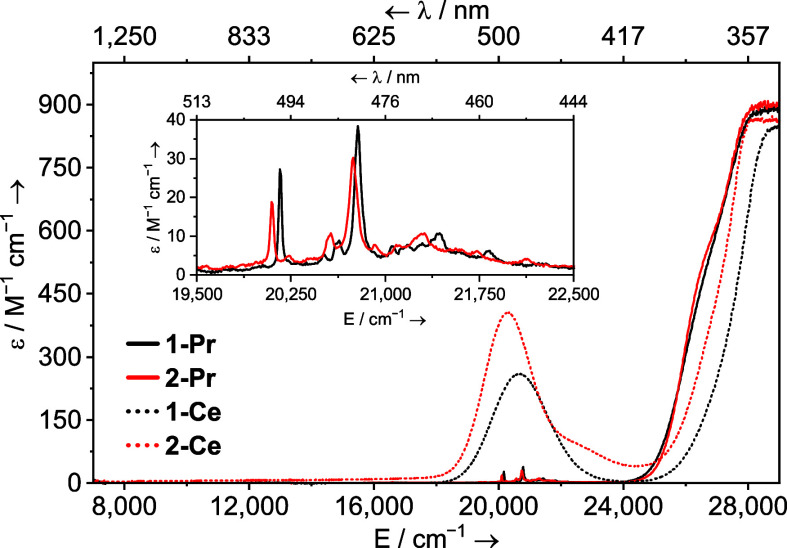
Solution UV–vis–NIR spectra of [M(Cp*)_2_(I)(THF)] (**1M**, M = Ce, dotted black; Pr, solid black) (3 mM, THF) and [M(Cp*)_2_(I)(I^Me4^)] (**2M**, M = Ce, dotted red; Pr, solid red) (3 mM, toluene) shown between 7000 and 29,000 cm^–1^ (1429–345 nm) at ambient temperature.

The UV–vis–NIR spectra of **1M** (M = La, Ce, Pr, and Nd) complexes were collected in THF at ambient temperature and can be compared with the spectra of their **2M** counterparts collected in toluene. Figure 9 shows the spectra of **1Pr**, **2Pr**, **1Ce**, and **2Ce** as examples. The influence of THF vs I^Me4^ bonding on the f → f and f → d transitions in **1M** and **2M** is instructive toward the potential origin of the structural difference between the 4f and 5f series.

The absorption spectra of both **1Ce** and **2Ce** are simple and characteristic of Ce^3+^ (4f^1^, ^2^F_5/2_) complexes. A broad, somewhat featureless transition tails in from the UV region down to ca. 24,000 cm^–1^ (417 nm), and a single additional broad peak is observed, which is the 5d ← 4f dipole allowed interconfigurational transition. In **1Ce,** this is seen at 20,675 cm^–1^ (484 nm, ε = 259 M^–1^ cm^–1^), and in **2Ce,** it lies at 20,308 cm^–1^ (492 nm, ε = 406 M^–1^ cm^–1^), a red shift of 367 cm^–1^. See the Supporting Information for a computational analysis of this transition, which supports this assignment. For Pr^3+^ (4f^2^, ^3^H_4_) ions, the 4f → 5d absorption energy is usually sufficiently large that it does not interfere with the vis–NIR absorption spectrum,^99−104^ and it is the first member of the series for which f → f (Laporté forbidden, intraconfigurational) transitions are observed and thus can directly report on the impact of THF vs I^Me4^ donor properties on the 4f manifold. The ^3^P_0_ ← ^3^H_4_ and ^3^P_1_ ← ^3^H_4_ transitions typically occur around 20,700 cm^–1^ (483 nm) and 21,400 cm^–1^ (467 nm), respectively.^99−101,103−105^ In **1Pr** these appear at 20,161 cm^–1^ (496 nm, ε = 27 M^–1^ cm^–1^ for ^3^P_0_) and 20,782 cm^–1^ (481 nm, ε = 38 M^–1^ cm^–1^ for ^3^P_1_). In **2Pr**, these transitions occur at 20,097 cm^–1^ (498 nm, ε = 19 M^–1^ cm^–1^) and 20,747 (482 nm, ε = 16 M^–1^ cm^–1^), and so, like with **1Ce** and **2Ce** above, both exhibit a modest redshift in **2Pr** vs **1Pr**, though less than the cerium complexes. When comparing **1Nd** and **2Nd**, we see a much smaller redshift between most of the features than what is seen for the Ce^3+^ and Pr^3+^ complexes, though the spectra are much more complex (Figure S82), which precludes assigning a redshift value between any set of peaks. However, when comparing **2Nd** [Nd(Cp*)_2_(I)(I^Me4^)] to **5Nd** [Nd(Cp*)_2_(Cl)(I^Me4^)], we see essentially no difference (Figure S93) reflecting that replacing chloride with iodide has little impact upon the observed electronic transitions.

**Figure 10:**
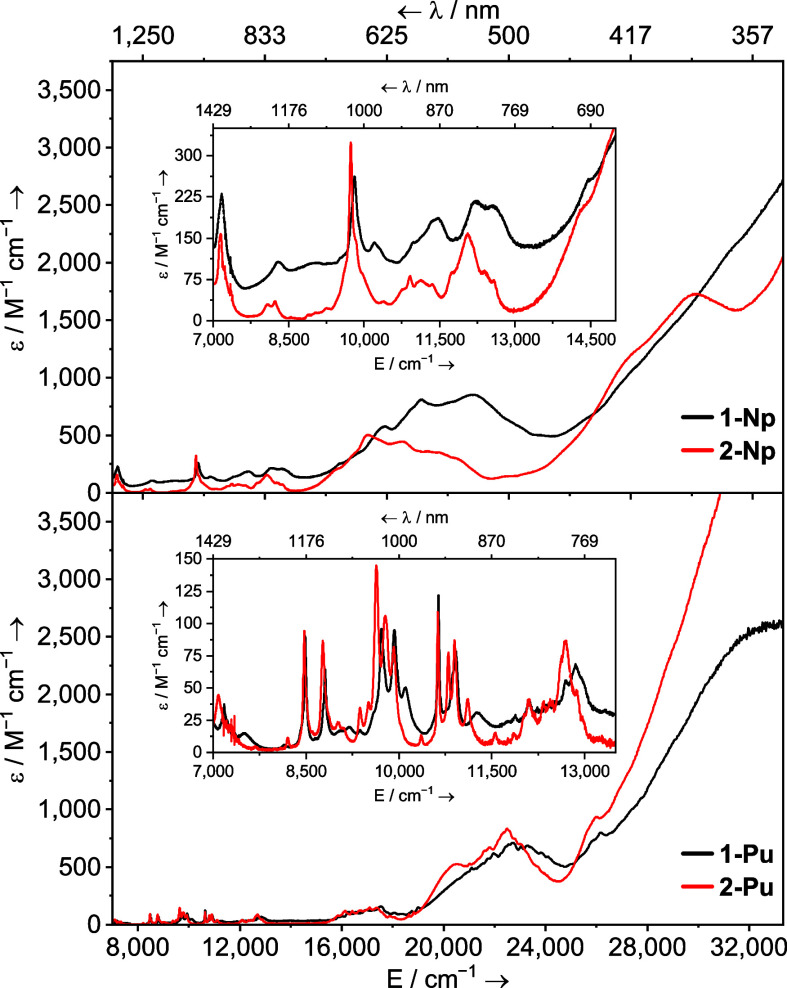
Top: solution UV–vis–NIR spectrum of [Np(Cp*)_2_(I)(THF)] (**1Np**, black line) and [Np(Cp*)_2_(I)(I^Me4^)] (**2Np**, red line) in toluene. Bottom: solution UV–vis–NIR spectra of [Pu(Cp*)_2_(I)(THF)] (**1Pu**, black line) and [Pu(Cp*)_2_(I)(I^Me4^)] (**2Pu**, red line). All spectra were collected in toluene at ambient temperature and are shown between 7000–33,333 cm^–1^ (1429–333 nm).

The UV–vis–NIR spectra of **2Np** and **2Pu** are remarkably similar to the previously reported spectra for **1Np** and **1Pu** (Figure 10).^68^ All four feature broad absorptions which tail in from the UV region down to ca. 24,000 cm^–1^ (417 nm), which lead into a series of poorly resolved features presumably derived from the 6d ← 5f transitions with fine structure arising from splitting of the 5f manifold or vibronic coupling of the excited 6d state to ligand modes. For **1Np**, we previously noted a main band which extends from ca. 14,000–22,000 cm^–1^ (ca. 714–455 nm, ε_max_ = 849 M^–1^ cm^–1^), which appears somewhat red-shifted in **2Np** and resides from ca. 13,000–20,000 cm^–1^ (769–500 nm, ε_max_ = 498 M^–1^ cm^–1^; Figure 10 top). Complex **1Pu** shows a similar broad peak from ca. 18,000–25,000 cm^–1^ (556–400 nm, ε_max_ = 704 M^–1^ cm^–1^), which appears much less red-shifted in **2Pu** (Figure 10 bottom) than between the Np^3+^ examples, such that the feature approximately overlaps the same range as in **1Pu** (i.e., 18,000–25,000 cm^–1^; 556–400 nm; ε_max_ = 831 M^–1^ cm^–1^). Fitting the peak groupings with a Gaussian curve (see Figures S94 and S95) shows the center of the main 5f → 6d grouping in **1Np** resides at 18,309(6) cm^–1^, while for **2Np,** it is at 16,467(9) cm^–1^ which is roughly 1800 cm^–1^ lower in energy. For **1Pu** (22,947(7) cm^–1^) and **2Pu** (22,211(13) cm^–1^), the shift is much smaller at ca. 730 cm^–1^. This trend of increasing energy in the 6d ← 5f transitions from Np to Pu is consistent with previous works.^68−70^

At the lower energy region of these spectra, characteristically weak and somewhat sharp Np^3+^ and Pu^3+^ f → f transitions can be seen^106−110^ and are remarkably similar within each of the two pairs. As with **2Pr** and **2Nd**, there is a small red shift for the I^Me4^ adduct vs the THF-adduct of ca. 70 to 200 cm^–1^ for **2Np**, depending on the pairs of peaks chosen, and at most ca. 150 cm^–1^ for **2Pu**.

**Figure 11:**
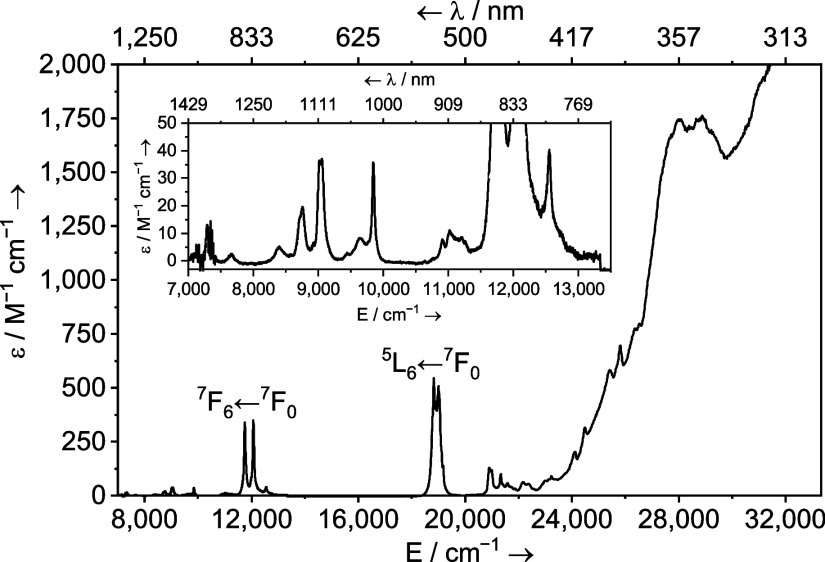
Solution UV–vis–NIR spectra of [Am(Cp*)_2_(I_*x*_Cl_1–*x*_)(I^Me4^)] (**4Am**, ca. 1 mM, toluene) shown between 7000–33,000 cm^–1^ (1429–303 nm) at ambient temperature. Molar absorptivity values were based on using the MW of the mixed I/Cl species, but individual bands are not assigned to the specific I vs Cl species.

Finally, the UV–vis–NIR spectrum of **4Am** (Figure 11) is broadly typical of Am^3+^ in solution, whereby we can identify features corresponding to the ^7^F_6_ ← ^7^F_0_ and ^5^L_6_ ← ^7^F_0_ transitions; however, both appear to be “doubled”. In **4Am**, the higher-energy ^5^L_6_ ← ^7^F_0_ transition appears as two sharp peaks, 18,832 cm^–1^ (531 nm, ε = 544 M^–1^ cm^–1^) and 19,004 cm^–1^ (526 nm, ε = 507 M^–1^ cm^–1^), while the ^7^F_6_ ← ^7^F_0_ transition appears as two sharp peaks at 11,751 cm^–1^ (851 nm, ε = 340 M^–1^ cm^–1^) and 12,071 cm^–1^ (828 nm, ε = 348 M^–1^ cm^–1^). A similar, but genuine, splitting of the high energy ^5^L_6_ ← ^7^F_0_ feature was seen in [Am(Cp^tet^)_3_],^81^ and also in [{Am(Cp′)_3_}_2_(μ-4,4′-bipy)].^18^ With **4Am**, given the NMR and structural data which strongly suggest that the bulk is a mixture of [Am(Cp*)_2_(I)(I^Me4^)] and the chloride congener, it is likely that the doubling is in fact due to differences in the f → f transitions of around 172 and 320 cm^–1^ for the major transitions in the iodide- and chloride-ligated complexes. This contrasts the little to no effect with Nd (vide supra).

The influence of THF vs I^Me4^ coordination on the f → f transitions in Np^3+^ and Pu^3+^ appears to be larger than seen with Pr^3+^ and Nd^3+^, which might be expected based upon the better spatial overlap of the 5f orbitals with ligands vs that of the 4f orbitals in the lanthanide counterparts. However, it is smaller than the changes seen in the 5f → 6d region of all of the spectra, which suggests that the key differences in the way THF and I^Me4^ bind to these ions involve the 5d (lanthanide) and 6d (actinide) orbitals. These data support conclusions derived from the calculations.

## Conclusions

Analysis across a series of NHC-ligated bent-metallocene complexes, [M(Cp*)_2_(X)(I^Me4^)] (X = I, M = La, Ce, Pr, Nd, U, Np, and Pu; X = Cl, M = Nd; X = I/Cl, M = Nd, and Am), reveals significant shortening of the metal–C_NHC_ ligand bond length with actinide metals vs lanthanide examples with closely matched ionic radii. This homologous series extends from La to Nd and from U to Am, including rare or unique examples of M–C σ-bonding to NHC ligands in the case of Pu and Am. Most remarkably, we observe no significant decrease in the extent of An vs Ln metal–C_NHC_ length shortening as the series are traversed. Structural and quantum chemical analyses between the NHC complexes and their THF-ligated precursors reveal that hard/soft arguments of the primarily electrostatic origin explain the anticipated lanthanide vs actinide differences in M–I and M–O bond lengths. However, the An–NHC π-type Kohn–Sham molecular orbitals consistently feature small (ca. 1–2%) 6d contributions, which are absent for all the lanthanide congeners. Quantum theory of atoms in molecules data suggest a consistently larger M–C_NHC_ interaction in the 5f series, and the computed barrier to rotation of the NHC ligand around the U–C_NHC_ axis is larger than that for the La analogue. Together, these results suggest larger M–C_NHC_ covalency in the actinide series and that ligand-π to vacant 6d interactions can differentiate lanthanide and actinide ions with these neutral donor ligands. This is a key distinction between this molecular framework and others and could more generally inform the design of future ligand systems to differentiate otherwise similar f-block ions. Typically, lanthanide and actinide differences (within homologous donor series) are driven by the modest differences between the 4f and 5f manifold, but for the NHC complexes here, it is 6d orbital participation in the form of a minor π-bonding contribution in these simple Lewis-base adducts which is present in the actinide complexes but absent in the lanthanide congeners which correlates with substantial structural and spectroscopic differences. This bonding mechanism was proposed over 30 years ago with simple π-basic ligands on uranium, and this work presents evidence that extends deeper into the transuranium series.^94^ In principle, strong σ-donor ligand systems capable of π-donation, such as amides or alkoxides, may show similar effects to those found herein. However, this may be restricted to metals in lower oxidation states (i.e., An^3+^), as previous works on Np^4+^ alkoxides show π-donation into the 5f manifold, rather than 6d.^62^ As organometallic chemistry of the transuranium elements is experiencing a renaissance as far across the actinide series as californium (the highest atomic number element which can be used for synthetic molecular chemistry on a mg scale), the results here suggest future studies into the role of d orbital-derived π-bonding beyond uranium, and indeed beyond americium, will be insightful.^71,111^

## Supporting Information Available

Experimental details, photographs taken during syntheses, crystallography details, molecular structure details and metrics, NMR and UV–vis–NIR spectra, and computational details (PDF)
