# Supplementary material for: N-Heterocyclic Carbene to Actinide d-Based π-bonding Correlates with Observed Metal–Carbene Bond Length Shortening Versus Lanthanide Congeners
Source: J Am Chem Soc. 2024 Apr 3;146(15):10367–80. doi: 10.1021/jacs.3c12721 (PMC11029940; doi:10.1021/jacs.3c12721)
Supplement: Supplementary file 1 — ja3c12721_si_001.pdf [file ja3c12721_si_001.pdf]

Supporting Information for:

N-heterocyclic carbene to actinide *d*-based π-bonding correlates with observed metal–carbene bond length shortening *versus* lanthanide congeners

Conrad A. P. Goodwin,[1,2,3] Ralph W. Adams,[3] Andrew J. Gaunt,*[1] Susan K. Hanson,[1] Michael T. Janicke,[1] Nikolas Kaltsoyannis,*[2,3] Stephen T. Liddle,[2,3] Iain May,[1] Jeffrey L. Miller,[1] Brian L. Scott,[4] John A. Seed,[2,3] and George F. S. Whitehead[3]

[1] Chemistry Division, Los Alamos National Laboratory, Los Alamos, New Mexico, 87545 (USA).

[2] Centre for Radiochemistry Research, The University of Manchester, Oxford Road, Manchester, M13 9PL (UK).

[3] Department of Chemistry, The University of Manchester, Oxford Road, Manchester, M13 9PL (UK).

[4] Materials Physics & Applications Division, Los Alamos National Laboratory, Los Alamos, New Mexico, 87545 (USA).

*Correspondence: gaunt@lanl.gov, nikolas.kaltsoyannis@manchester.ac.uk

Table of Contents

S1. Experimental Details 4
Radiological considerations 4
Equipment, materials, and solvents used with transuranium elements 4
Equipment, materials, and solvents used with lanthanide elements 6
A note on NMR spectroscopy of paramagnetic samples 7
Notes on the synthesis of I^{Me4}=S and I^{Me4} 10
Synthesis of crystals of [Ln(Cp*)$_2$(I)(THF)] (**1Ln**, Ln = La, Ce, Pr, Nd) 14
Synthesis of [Ln(Cp*)$_2$(I)(I^{Me4})] (**2Ln**, Ln = La, Ce, Pr, Nd, Y) 18
Synthesis of [Ce(Cp*)$_2$(I)(I^{Me4})] (**2Ce**) from I^{Me4} and "Ce(Cp*)$_2$(I) (**3**)" 23
Synthesis of [An(Cp*)$_2$(I)(I^{Me4})] (An = Np, **2Np**; Pu, **2Pu**) 25
Synthesis of [Nd(Cp*)$_2$(I_xCl_{1-x})(I^{Me4})] (**4Nd**) from $NdCl_3$ dissolved in $HCl_{(aq)}$ 27
Purification of Am^{3+} $_{(aq)}$ from aged oxide and stainless steel contamination 29
Synthesis of [Am(Cp*)$_2$(I_xCl_{1-x})(I^{Me4})] (**4Am**) from $^{243}Am^{3+}$ dissolved in $HCl_{(aq)}$ 31
Synthesis of [Nd(Cp*)$_2$(Cl)(I^{Me4})] (**5Nd**) from $NdCl_3$ 34
S2. Photographs taken during synthesis 36
Lanthanide syntheses 36
Neptunium and plutonium syntheses 38
Synthesis of **4Am** 43
S3. Crystallography 48
S4. Molecular structures 54
Complexes **1Ln** (Ln = La, Ce, Pr, Nd) 54
Bond metrics for **1La** 56
Bond metrics for **1Ln** (Ln = Ce, Pr, Nd) 57
Bond metrics for previously-reported **1An** (An = U, Np, Pu) 58
Complexes **2Ln** (Ln = La, Ce, Pr, Nd, Y) 59
Bond metrics for **2Ln** (Ln = La, Ce, Pr, Nd, Y) 61
Bond metric comparison for **2Ln** and **2Ln$^{\beta}$** (Ln = La, Ce) 62
Complexes **2Np** and **2Pu** 63
Bond metrics for **2An** (An = U, Np, Pu) 64
Comparison of **1M** to **2M** (M = La, Ce, Pr, Nd, U, Np, Pu) 65
Complexes **4Nd** and **4Am** 66
Complex **5Nd** 68
Bond metrics comparison for **2Nd**, **4Nd**, **4Am**, and **5Nd**. 69
Plots comparing M–C_{NHC} and M–I bond metrics for **1M**, **2M** and **4Am**. 70
S5. NMR spectroscopy plots 72
NMR spectra of [Ln(Cp*)$_2$(I)(THF)] (**1Ln**; Ln = La, Ce, Pr, Nd) 72
NMR spectra of [Ln(Cp*)$_2$(I)(I^{Me4})] (**2Ln**; Ln = La, Ce, Pr, Nd, Y) 75
NMR spectra of [An(Cp*)$_2$(I)(I^{Me4})] (**2An**; An = Np, Pu), and [Am(Cp*)$_2$(I_xCl_{1-x})(I^{Me4})] (**4Am**) 81
Comparison of ^1H NMR spectra between [Nd(Cp*)$_2$(X)(I^{Me4})] (X = I, **2Nd**; Cl, **5Nd**) 89
Magnetic moments determined by NMR spectroscopy (Evans method) 90
S6. UV-vis-NIR spectra 91
UV-vis-NIR spectra of [Ln(Cp*)$_2$(I)(THF)] (**1Ln**, Ln = La, Ce, Pr, Nd) 91
UV-vis-NIR spectra of [Ln(Cp*)$_2$(I)(I^{Me4})] (**2Ln**, Ln = La, Ce, Pr, Nd, Y) 93
Comparison of UV-vis-NIR spectra between **1Ln** and **2Ln** (Ln = La, Ce, Pr, Nd) 96
UV-vis-NIR spectra of [Np(Cp*)$_2$(I)(THF)] (**1Np**) and [Np(Cp*)$_2$(I)(I^{Me4})] (**2Np**) 98
UV-vis-NIR spectra of [Pu(Cp*)$_2$(I)(THF)] (**1Pu**) and [Pu(Cp*)$_2$(I)(I^{Me4})] (**2Pu**) 101

UV-vis-NIR spectrum of [Am(Cp*)$_2$(I$_x$Cl$_{1-x}$)(I^{Me4})] (**4Am**) ... 102
Comparison of UV-vis-NIR spectra between [Nd(Cp*)$_2$(X)(I^{Me4})] (X = I, **2Nd**; Cl = **5Nd**) ... 103
Gaussian fitting of 6d←5f peaks for **1Pu**, **2Pu**, **1Np**, and **2Np** ... 104
S7. Quantum chemical calculations ... 108
General considerations ... 108
TD-DFT calculations ... 110
Cartesian coordinates (Å) and SCF energies ... 111
S8. References ... 130

S1. Experimental Details

Radiological considerations

Caution! The ^{237}Np ($t_{½}$ = 2.144×10^{6} years), ^{239}Pu ($t_{½}$ = 2.411×10^{5} years), and ^{243}Am ($t_{1/2}$ = 7370 y) radionuclides – and their daughters – present serious health threats due to their α-, β-, and γ-emissions. ^{237}Np establishes a secular equilibrium (asymptotical concentration at 34.6 ppb) with the potent β-emitter ^{233}Pa ($t_{1/2}$ = 26.975(13) days, A = 777 TBq g^{-1}), which pertains also to an intense γ-ray emission. ^{243}Am is a high specific-activity α-particle (5.277 MeV)[1] and γ-ray (142 keV) emitting radionuclide and poses serious health risks if not properly handled and contained (as well as α/β/γ-radiation hazards from daughter products which notably include the short-lived ^{239}Np, a high energy 722 KeV β-particle emitter with $t_{1/2}$ = 2.35 d and 278 keV γ-rays). Accordingly, the primary occupational hazard of these systems is due to severe radiotoxic (α-particles) and heavy-metal toxicity effects, though for ^{237}Np cumulative tissue absorptive dose rates must be considered and protected against. Hence, all studies that involved manipulation of these isotopes were conducted in a radiation laboratory equipped with high-efficiency particulate air (HEPA) filtered hoods and in negative pressure gloveboxes. Additional safeguards included continuous air monitoring and use of hand-held radiation monitoring equipment. Entrance to the laboratory space was controlled with a hand and foot radiation monitoring instrumentation and a full body personal contamination monitoring station. The handling of free-flowing solids was restricted to be within negative-pressure gloveboxes equipped with HEPA filters. In addition to standard laboratory PPE, aqueous solutions were handled using multiple layers of latex gloves combined with DuPont™ Tyvek® 400 sleeves to provide overlapping coverage of the arms. Due to these radiological hazards, elemental analyses were not possible for ^{237}Np, ^{239}Pu, and ^{243}Am-containing materials.

Equipment, materials, and solvents used with transuranium elements

A negative-pressure, transuranium capable, helium atmosphere glovebox (UHP helium – AirGas) was used for all work involving the synthesis of transuranium compounds. The glovebox atmosphere

was maintained with a standalone Vacuum Atmosphere Genesis™ oxygen and moisture removal system, and atmosphere suitability was verified using a dilute toluene solution of $[Ti(Cp)_2(\mu\text{-}Cl)]_2$ (200 mg of commercial $[Ti(Cp)_2(Cl)_2]$ reduced over an excess of Zn powder in 20 mL of toluene, and filtered) prior to any manipulations such that the residue dried to a dark green color each time (a color change to yellow or orange indicates decomposition of the Ti test compound and atmospheric O_2/H_2O removal is required). Anhydrous THF (Sigma Aldrich), anhydrous *n*-hexane (Sigma Aldrich), and anhydrous toluene (Sigma Aldrich) were transferred onto activated 3 or 4 Å molecular sieves, stored for 1 week and degassed before use. D_6-benzene (Cambridge Isotopes) was stored over activated 4 Å molecular sieves and degassed before use – all solvents were tested with a dilute THF solution of Na_2Ph_2CO (150 mg Ph_2CO in 20 mL of THF with an excess of Na metal) such that THF required 1 drop / mL to retain purple coloration and hydrocarbon solvents required 1 drop / 2 mL.

All glassware, and glass-fibre filter discs, was stored in a vacuum oven (>150 °C) for 24 hours prior to being brought into the glovebox, and FEP (fluorinated ethylene propylene) NMR liners were brought into the box *via* overnight or multi-hour vacuum cycles. A vacuum oven was not used for reactions involving non-transuranium elements. Transuranium crystals for single-crystal X-ray diffraction were mounted in Paratone-N or NVH oil inside 0.5 mm quartz capillaries (Charles Supper). The quartz capillaries were inserted through silicone stoppers and placed inside test tubes to allow handling inside the transuranium glovebox while mounting crystals without contaminating the exterior surface of the capillary. The capillaries were then cut to appropriate size for later goniometer mounting with nail clippers. The ends of the cut capillaries were sealed with hot capillary wax before being removed from the glovebox for coating in clear nail varnish (Hard as Nails™) to provide shatter-resilience.[2] During the clipping and wax sealing steps, care must be taken to avoid the capillary touching any contaminated surfaces (this is achieved by the introduction of fresh petri dishes, forceps, clippers, and wax, as needed in conjunction with careful handling techniques to avoid contamination transfer). Solution phase UV-vis-NIR spectra were collected at ambient temperature using a Varian Cary 6000i UV-vis-NIR spectrometer. The solution was contained in a

low volume (1 mL) screw-capped quartz cuvette (1 cm path length) that was loaded in a transuranium glovebox (or HEPA filtered fume hood as appropriate) using Parafilm to protect the exterior surface of the cuvette and cap from radioactive contamination (parafilm removed in fume hood prior to data acquisition). Data was collected from 40,000 to 6,250 cm^{-1} (250 to 1,600 nm). Where ε values are reported for molecular complexes below there is a modest error due to the small quantities of weighed material, as is nearly always the case when these values are reported from synthetic chemistry (as opposed analytical determination methods). Nonetheless it is a useful metric to determine based on weight of crystal dissolved and solvent weight. For NMR spectroscopy, solution was loaded into a fresh FEP NMR liner that was protected from surface contamination with Parafilm while inside a transuranium glovebox. The liner was sealed with two PTFE plugs, brought out of the glovebox, and verified to be free of surface contamination before the parafilm was removed. The liner was then loaded into a J. Young tap appended 5 mm NMR tube, the headspace was then evacuated and refilled with He to provide an inert atmosphere headspace above the sample. NMR spectroscopic data collection was performed on a Bruker Avance II (400 MHz) at 295 K to 299 K.

Equipment, materials, and solvents used with lanthanide elements

Unless otherwise described, all syntheses and manipulations were conducted under UHP argon (AirGas) with rigorous exclusion of oxygen and water using Schlenk line and glove box techniques in an MBraun Labmaster Pro™. 4 Å molecular sieves were activated by heating for 36 hours at 200 °C, 10^{-4} mbar. Anhydrous THF (Sigma Aldrich) was transferred onto activated 4 Å molecular sieves, stored for 1 week and degassed before use. D_6-benzene (Cambridge Isotopes), anhydrous THF (Sigma Aldrich), anhydrous *n*-hexane (Sigma Aldrich), and anhydrous toluene (Sigma Aldrich) were transferred onto activated 3 or 4 Å molecular sieves, stored for 1 week and degassed before use. 1-hexanol (Sigma Aldrich, reagent grade), and Me_3SiCl (Sigma Aldrich, storred over copper) were used without purification. All solvents used for organometallic/coordination chemistry were tested with a dilute THF solution of Na_2Ph_2CO (150 mg Ph_2CO in 20 mL of THF with an excess of Na

metal) such that THF required 1 drop / mL to retain purple coloration and hydrocarbon solvents (including D_6-benzene) required 1 drop / 2 mL. [$AnI_3(THF)_4$] (An = Np, Pu) were prepared as described elsewhere.[3] Anhydrous LnI_3 (Ln = La, Ce, Pr, Nd) and anhydrous $NdCl_3$ were commercial samples (Alfa Aesar), used as received and stored under Ar. KCp* (KC_5Me_5) was prepared according to a literature procedure,[4] from HCp* and KN″. KN″ (N″ = $N(SiMe_3)_2$) (Strem) was used as received. 1,3-dihydro-1,3,4,5-tetramethyl-2*H*-imidazole-2-thione (I^{Me4}=S),[5] and 1,3-dihydro-1,3,4,5-tetramethyl-2*H*-imidazol-2-ylidene (I^{Me4}) were synthesized by modifications of literature procedures,[6] see below for notes on the synthesis of these two compounds. All glassware, and glass-fiber filter discs, were stored in an oven (>150 °C) for 24 hours prior to being brought into the glovebox. Solution phase UV-vis-NIR spectra were collected at ambient temperature using a Varian Cary 6000i UV-vis-NIR spectrometer. The solution was contained in a low volume (1 mL) screw-capped quartz cuvette (1 cm path length). ATR FT-IR spectra of microcrystalline samples were collected using a Bruker ALPHA II FT-IR spectrometer equipped with a Platinium ATR module with diamond window. NMR spectroscopic data collection was performed on a Bruker Avance II (400 MHz) at 295 K to 299 K.

A note on NMR spectroscopy of paramagnetic samples

All spectra were referenced to internal solvent residuals (^{1}H and ^{13}C) or externally to 10% TMS in D_3-chloroform (^{31}P) *via* Equation S1, which is the IUPAC recommended convention.

Equation S1.

$$\Delta\ (Hz) = \frac{SR^{1H}}{SF^{1H}} \times SF^{NUC}$$

Where SR^{1H} is the spectrum reference frequency (in Hz) of a reference ^{1}H NMR spectrum collected with TMS set to 0 ppm collected under the same experimental conditions; SF^{1H} is the spectrometer frequency (in MHz) for the ^{1}H nucleus; SF^{NUC} is the spectrometer frequency (in MHz) of the nucleus in question. The answer is given in Hz.

Paramagnetic samples become magnetized in the presence of an external magnetic field, such as that of an NMR spectrometer. The level of magnetisation will approximately follow Curie's law when saturation of magnetisation is not reached ($\mu_B \leq k_BT$). The magnetic response of a sample is proportional to: (i) sample temperature; (ii) external field strength; and (iii) sample concentration. This necessarily affects the reproducibility of the chemical shifts given for paramagnetic samples – a sample run at a different concentration, or a different field strength, or at a different temperature, will produce a different paramagnetic contribution to the observed chemical shift. Moreover, the direction that an individual chemical shift will change (upfield or downfield) cannot easily be predicted.[7] Finally, modern convention to reference chemical shift relative to solvent residual peaks further complicates the comparison of multiple samples as the factors listed above will also change the absolute shift of the solvent peak (relative to the spectrometer proton frequency) as the susceptibility of solvent molecules may differ from the ligand atoms surrounding a paramagnetic ion. Though, solvent effects even in diamagnetic NMR samples can vary chemical shift by several ppm for some nuclei.[8]

Considering these caveats, we report our data as it is output from experiment with rounding to two decimal places as this is convention. We defer to the expertise of the reader to interpret the data reported here in a way that is appropriate for their needs.

Table S1. Compound numbering, formula, reaction scale (by metal quantity used), yield, and then % yield where appropriate.

	Reaction scale (μmol metal)	Formula	Yield (mg)	% yield
**1La**	154	$[La(Cp^*)_2(I)(THF)_n]$ (n = 0–1)	40.4	43–49 †
**1Ce**	154	$[Ce(Cp^*)_2(I)(THF)_n]$ (n = 0–1)	30.5	32–37 †
**1Pr**	153	$[Pr(Cp^*)_2(I)(THF)_n]$ (n = 0–1)	44.9	48–54 †
**1Nd**	152	$[Nd(Cp^*)_2(I)(THF)_n]$ (n = 0–1)	39.8	43–48 †
**2La**	154	$[La(Cp^*)_2(I)(I^{Me4})]$	40.4	40
**2La$^{\beta}$**	§	$[La(Cp^*)_2(I)(I^{Me4})]\cdot(C_7H_8)$	–	–
**2Ce**	154	$[Ce(Cp^*)_2(I)(I^{Me4})]$	40.7	40
**2Ce$^{\beta}$**	§	$[Ce(Cp^*)_2(I)(I^{Me4})]\cdot(C_7H_8)$	–	–
**2Pr**	153	$[Pr(Cp^*)_2(I)(I^{Me4})]$	37.0	37
**2Nd**	152	$[Nd(Cp^*)_2(I)(I^{Me4})]$	40.0	39
**2Y**	162	$[Y(Cp^*)_2(I)(I^{Me4})]$	31.9	32
**2Np**	38	$[Np(Cp^*)_2(I)(I^{Me4})]$	8.4	31
**2Pu**	38	$[Pu(Cp^*)_2(I)(I^{Me4})]$	12.7	38
**4Nd**	20	$[Nd(Cp^*)_2(I_xCl_{1-x})(I^{Me4})]$	–	–
**4Am**	21	$[Am(Cp^*)_2(I_xCl_{1-x})(I^{Me4})]$	4.2	– ‡
**5Nd**	160	$[Nd(Cp^*)_2(Cl)(I^{Me4})]$	21.8	24

† For **1M** (M = La, Ce, Pr, Nd) the amount of coordinated THF varies between 0 and 1 as the THF is labile in vacuo. We have provided elemental analysis results and an assessment below of the yield for both limiting cases $[M(Cp^*)_2(I)(THF)_n]$ where n = 0 or 1. § Several crystals of **1La$^{\beta}$** and **1Ce$^{\beta}$** were isolated from the supernatents of the parent **1M** crystallizations. ‡ due to uncertainty in the precise iodide / chloride ratio, it would be inappropriate to assign a yield here.

Notes on the synthesis of I^{Me4}=S and I^{Me4}

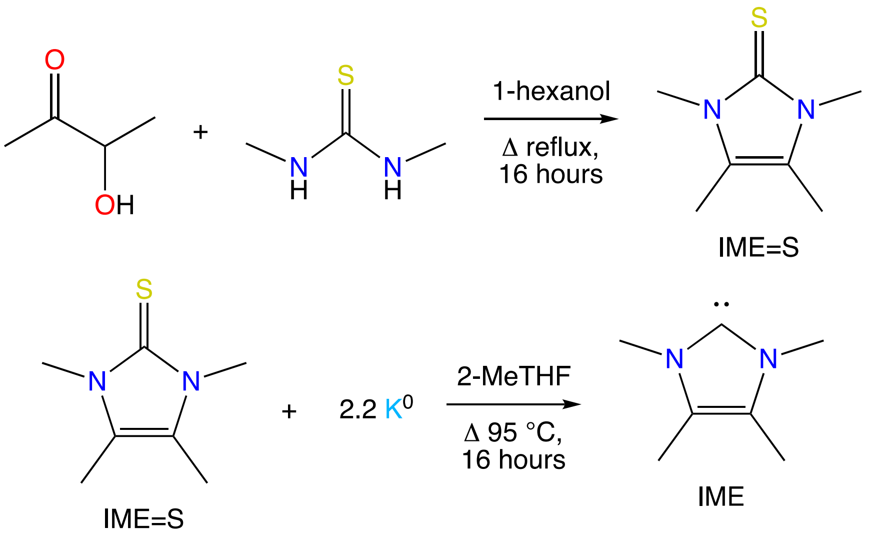

Scheme 1. The synthesis of I^{Me4}=S and I^{Me4} adapted from literature procedures.[5, 6]

Synthesis of I^{Me4}=S. The synthesis reported by Alcarazo was modified slightly.[5] We did not observe spontaneous precipitation of I^{Me4}=S once the reaction mixture was cooled to room temperature, instead the clear amber solution was stored in a freezer (–22 °C) for 3 hours. Upon removal from the freezer and stirring, fine needles promptly precipitated. The crystals were filtered cold, and washed with pre-cooled (–22 °C) Et_2O (8 × 10 mL) to afford I^{Me4}=S as a flowing white microcrystalline solid in comparable yield to previous reports. This material was then dried *in vacuo* (10^{-4} mbar) for 16 hours and stored in an inert atmosphere glovebox. For convenience, here we report the ^{1}H and ^{13}C{^{1}H} NMR spectra of I^{Me4}=S in anhydrous D_6-benzene so that its resonances can be easily discerned when this solvent is required for the collection of NMR spectra of metal complexes in the future (**Figure S1** and **Figure S2**).

^{1}H NMR (D_6-benzene, 400.13 MHz, 298 K): δ = 1.34 (s, 6 H, $C(C\underline{H}_3)_2$), 3.16 (s, 6 H, $N(C\underline{H}_3)_2$).

^{13}C{^{1}H} NMR (D_6-benzene, 100.62 MHz, 298 K): δ = 8.55 ($C(\underline{C}H_3)$), 31.46 ($N(\underline{C}H_3)$), 119.65 ($\underline{C}(CH_3)$), 163.53 ($\underline{C}$=S).

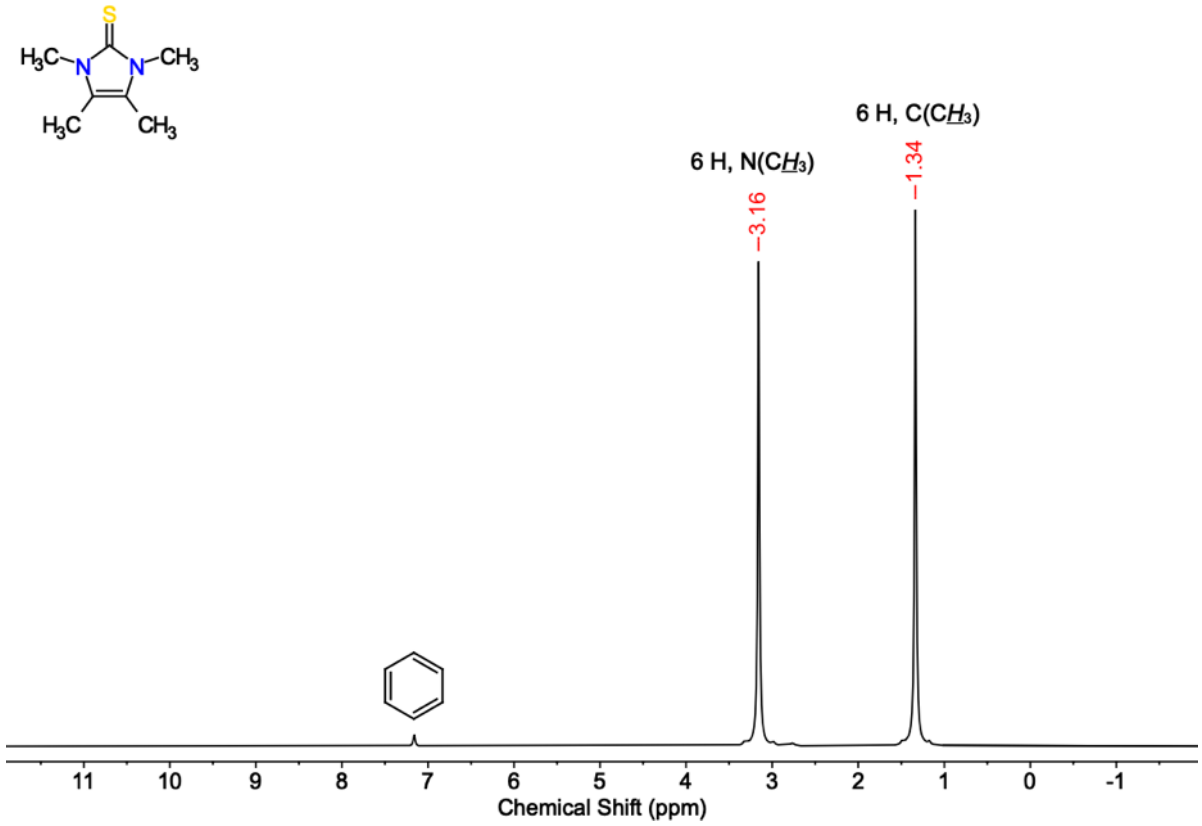

Figure S1. ^{1}H NMR spectrum of I^{Me4}=S in D$_6$-benzene.

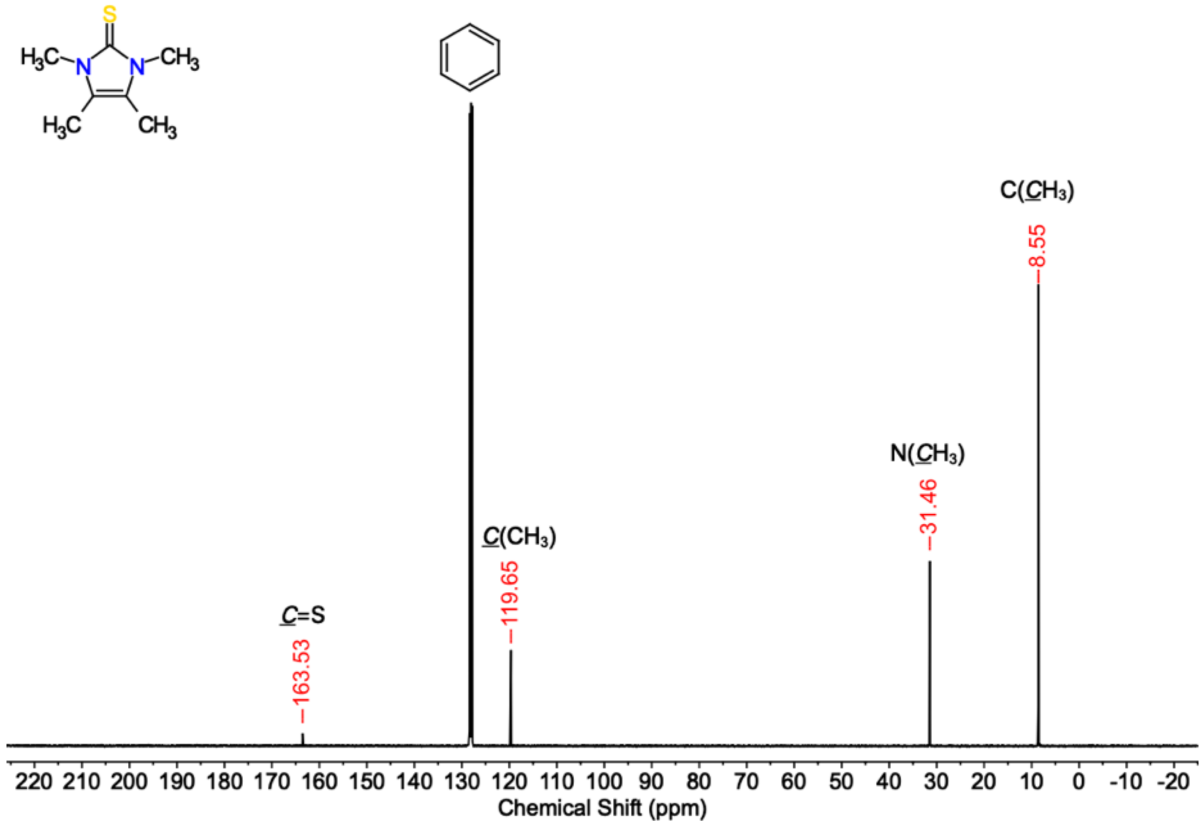

Figure S2. ^{13}C{^{1}H} NMR spectrum of I^{Me4}=S in D$_6$-benzene.

Synthesis of I^{Me4}. The synthesis reported by Spencer was modified slightly.[6] The reaction was performed in 2-MeTHF as the authors report, in a Schlenk tube appended with a Teflon stopper and evacuated to the solvent vapour pressure at room temperature. After stirring for 16 hours at 95 °C, the vessel was cooled to room temperature and brought into an inert atmosphere glovebox where workup was performed. The pale yellow supernatant was filtered from a pale blue powder, and residual K metal using a medium porosity glass fritt and a Büchner flask. Volatiles were removed *in vacuo* which afforded an off-white solid. This was dissolved in benzene, filtered into a 20 mL glass vial through 2 half-discs of glass microfiber mounted in a glass pipette, and dried *in vacuo* again which gave the product as a microcrystalline solid. The ^{1}H NMR spectrum is in agreement with the authors' report, and here we also provide the ^{13}C{^{1}H} NMR spectrum in D$_6$-benzene for completeness (**Figure S3** and **Figure S4**).

^{1}H NMR (D$_6$-benzene, 400.13 MHz, 298 K): δ = 1.60 (s, 6 H, C(C<u>*H*</u>$_3$)$_2$), 3.37 (s, 6 H, N(C<u>*H*</u>$_3$)$_2$).

^{13}C{^{1}H} NMR (D$_6$-benzene, 100.62 MHz, 298 K): δ = 8.83 (C(<u>*C*</u>H$_3$)), 35.19 (N(<u>*C*</u>H$_3$)), 122.58 (<u>*C*</u>(CH$_3$)), 213.29 (<u>*C*</u>=S).

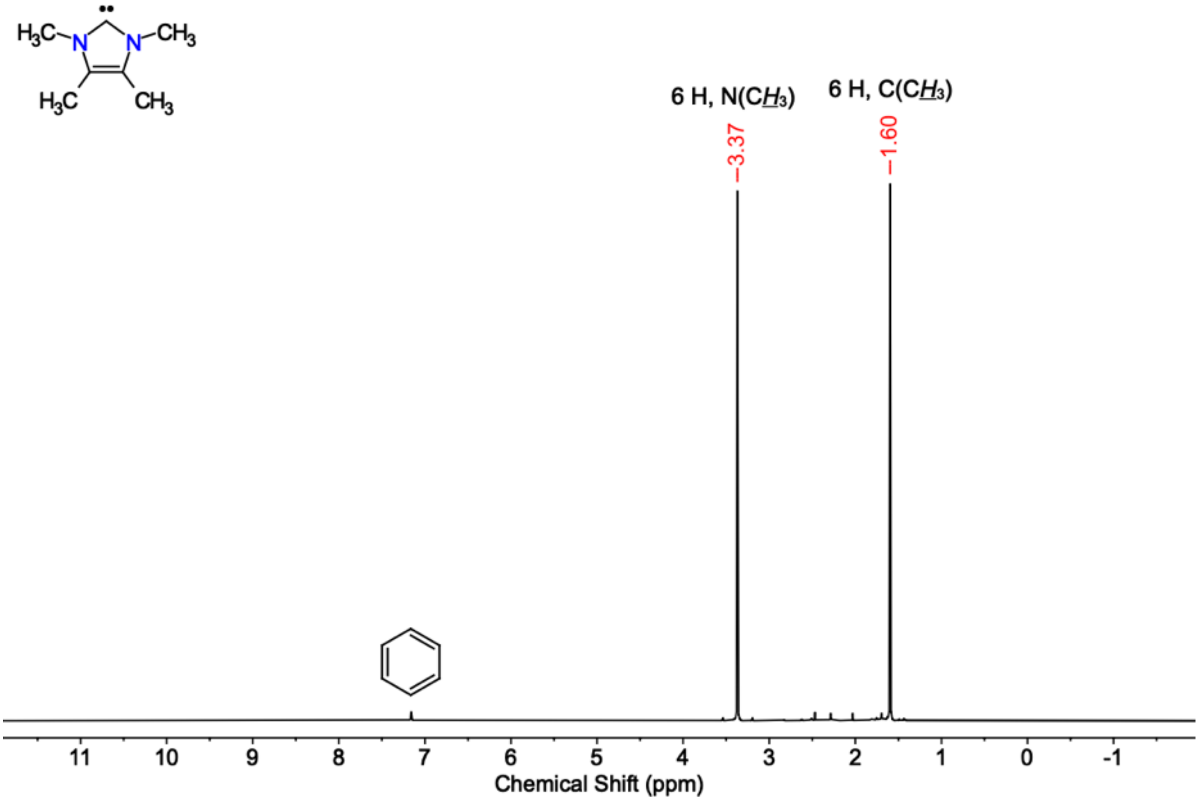

Figure S3. ^{1}H NMR spectrum of I^{Me4} in D$_6$-benzene.

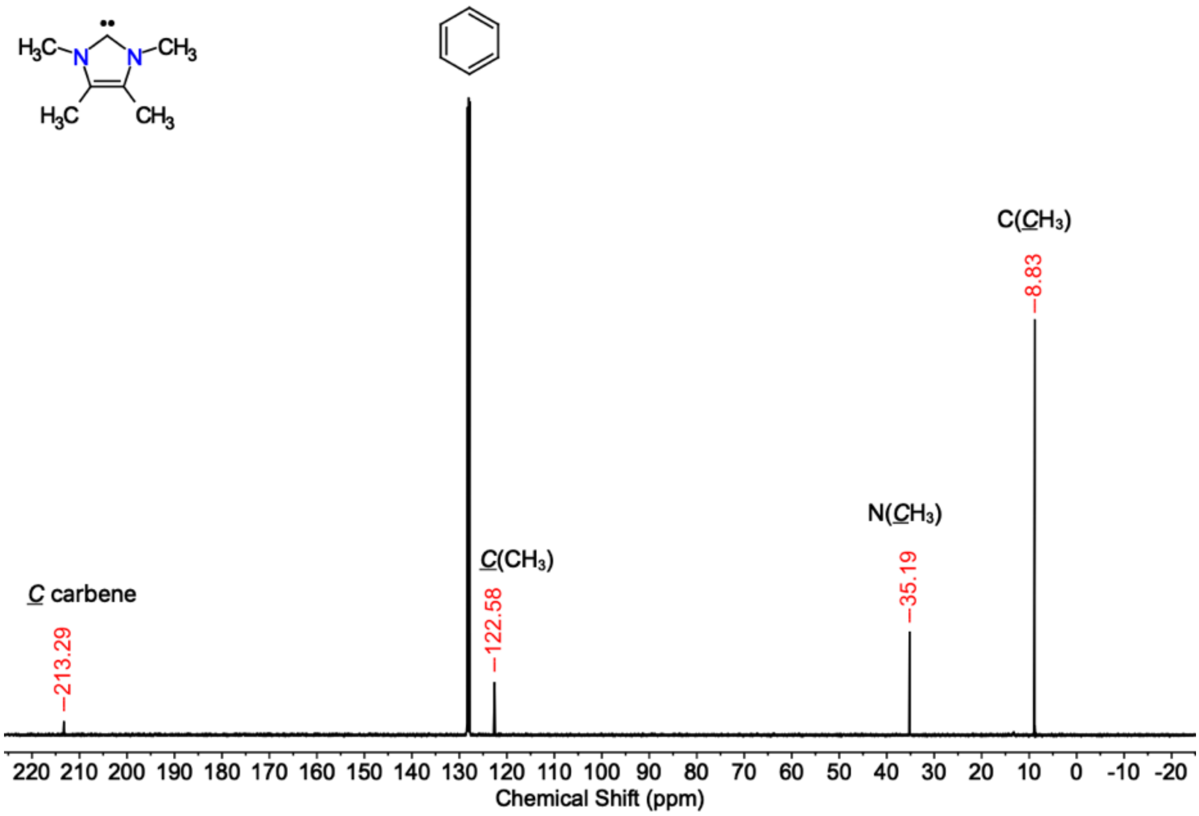

Figure S4. $^{13}C\{^{1}H\}$ NMR spectrum of I^{Me4} in D_6-benzene.

Synthesis of crystals of [Ln(Cp)$_2$(I)(THF)] (**1Ln**, Ln = La, Ce, Pr, Nd)*

All four [Ln(Cp*)$_2$(I)(THF)] (Ln = La, Ce, Pr, Nd) complexes were synthesized in an identical fashion and so a procedure is given for **1La**, along with characterization details for the others. Combustion analysis across three samples for each complex has shown agreement by C/H analysis, but no clear trend from La–Nd has emerged for example whether the larger metals desolvate more readily (*i.e.* form "Ln(Cp*)$_2$(I)" from **1Ln**) due to lowered charge-density. We provide yield values for both extremes of [Ln(Cp*)$_2$(I)(THF)$_n$] where n = 0 and 1. As lanthanide Cp* complexes routinely suffer from low C-combustion analysis results, the H-analysis is likely to be the only reliable metric of THF-content given the poor solubility of these complexes in hydrocarbon solvents and as such we suggest they be made *in-situ* for further chemistry provide characterization data from isolated crystalline samples for convenience. ^{1}H NMR spectroscopy was performed in D$_6$-benzene with a known quantity of [H]$_8$-THF added to aid solubility, this necessarily increases the error in the magnetic susceptibility determined by the Evans method due to the slight uncertainty in the solvent density, combined with the variable THF-content of the material. Figures of all spectra are presented below (**Figure S45** to **Figure S49**) to aid the reader.

Synthesis of [La(Cp*)$_2$(I)(THF)] (1La). THF (1.5 mL) was added to solid LaI$_3$ (80 mg, 154 μmol) in a 20 mL glass scintillation vial with a Teflon-coated stirrer bar, followed by solid KCp* (60 mg, 344 μmol, 2.2 equiv.). The colorless suspension was stirred and heated to a very gentle boil in the sealed vial for 45 minutes and then allowed to cool to room temperature, to afford a colorless solution with a white precipitate. The volatiles were removed *in vacuo*, and the flowing white powder was suspended in toluene (1.5 mL) and 1 drop (12 mg) of THF was added. The suspension was stirred for 45 minutes with modest heating (hot plate set to 50 °C), allowed to cool to room temperature, and then centrifuged (5,000 rpm, 10 minutes) which gave a pale straw-colored solution with a small amount of white powder. The solution was filtered into a 4 mL glass vial through 2 half-discs of glass microfiber mounted in a glass pipette and concentrated just past the point of incipient crystallization. It was important to not continue solvent removal for too long as the solids generally did not re-dissolve readily without the addition of more THF. The clear solution, with microcrystalline material

present on the vial walls was stored at –35 °C overnight (16 hours). A modest crop of colorless planks formed, from which the pale yellow supernatant was decanted and then the crystals were washed with hexane (2 × 2 mL) and dried *in vacuo* for 1 hour. A second crop of crystals formed over an additional overnight storage of the supernatant (separate from the hexane washings) at –35 °C, and these were washed and dried in the same manner (combined yield = 40.4 mg, 43% assuming $[La(Cp^*)_2(I)(THF)_n]$ $n = 1$; 49% if $n = 0$). Elemental analysis $n = 1$ calc. (%): C = 47.38, H = 6.30; $n = 0$ calc. (%): C = 44.79, H = 5.64; found (%): C = 44.31, H = 6.12.

^{1}H NMR (D_6-benzene + 1 drop (12 mg) $[H]_8$-THF, 400.13 MHz, 298 K): δ = 1.40 (s, THF 3,4-(C$\underline{H}_2$)), 2.07 (s, 30 H, Cp* C_5(C$\underline{H}_3$)$_5$), 3.57 (s, THF 2,5-(C$\underline{H}_2$)).

^{13}C{^{1}H} NMR (D_6-benzene + 1 drop (12 mg) $[H]_8$-THF, 100.62 MHz, 298 K): δ = 11.93 (Cp* C_5($\underline{C}H_3$)$_5$), 25.77 (s, THF 3,4-($\underline{C}H_2$)), 68.10 (s, THF 2,3-($\underline{C}H_2$)), 121.14 (Cp* $\underline{C}_5(CH_3)_5$).

UV-vis-NIR (THF): A featureless peak extends from ~390 nm (25,640 cm^{-1}) into the UV region, and beyond our spectral range.

FT-IR (ATR, microcrystalline): cm^{-1} = 354 (vw), 359 (vw), 374 (w), 379 (vw), 386 (vw), 402 (vw), 407 (vw), 410 (vw), 414 (vw), 425 (vw), 436 (vw), 440 (vw), 452 (vw), 460 (vw), 465 (vw), 477 (vw), 484 (vw), 490 (vw), 499 (vw), 506 (vw), 518 (vw), 525 (vw), 532 (vw), 536 (vw), 547 (vw), 551 (vw), 560 (vw), 564 (vw), 570 (vw), 577 (vw), 588 (vw), 608 (vw), 612 (vw), 624 (vw), 632 (vw), 639 (vw), 646 (vw), 669 (vw), 692 (vw), 702 (vw), 716 (vw), 726 (vw), 736 (vw), 745 (vw), 758 (vw), 771 (vw), 785 (vw), 799 (w), 834 (w), 856 (m), 910 (w), 919 (w), 948 (vw), 1,011 (m), 1,032 (w), 1,056 (vw), 1,117 (vw), 1,135 (vw), 1,159 (vw), 1,171 (w), 1,231 (vw), 1,242 (w), 1,290 (vw), 1,310 (w), 1,340 (w), 1,373 (m), 1,380 (w), 1,418 (m), 1,431 (m), 1,467 (w), 1,485 (w), 1,515 (vw), 1,528 (vw), 1,534 (vw), 1,544 (vw), 1,570 (vw), 1,584 (vw), 1,601 (vw), 2,717 (w), 2,845 (vs), 2,888 (vs), 2,940 (m), 2,956 (m), 2,964 (m).

Data for $[Ce(Cp^*)_2(I)(THF)]$ (1Ce). CeI_3 (80 mg, 154 μmol), KCp* (60 mg, 344 μmol, 2.2 equiv.). Orange, slightly fluorescent, solution and 1 crop of orange planks (yield = 30.5 mg, 32% assuming $[Ce(Cp^*)_2(I)(THF)_n]$ $n = 1$; 37% if $n = 0$). Elemental analysis $n = 1$ calc. (%): C = 47.29, H = 6.82; $n = 0$ calc. (%): C = 44.69, H = 5.63; found (%): C = 40.77, H = 6.09.

^{1}H NMR (D_6-benzene + 1 drop (12 mg) $[H]_8$-THF, 400.13 MHz, 298 K): δ = 1.34 (s, THF 3,4-(C*H*$_2$)), 3.52 (s, THF 2,5-(C*H*$_2$)), 5.35 (s, 30 H, Cp* C_5(C*H*$_3$)$_5$). Magnetic moment (Evans method, D_6-benzene + 1 drop (12 mg) $[H]_8$-THF, 298 K): μ_{eff} = 2.47 μB.

UV-vis-NIR (THF): λ_{max} (cm^{-1}; *ε*) = 485 (20,619, 247).FT-IR (ATR, microcrystalline): cm^{-1} = 351 (vw), 359 (vw), 362 (vw), 371 (vw), 376 (vw), 380 (vw), 385 (vw), 398 (vw), 401 (vw), 406 (vw), 414 (vw), 418 (vw), 424 (vw), 428 (vw), 433 (vw), 438 (vw), 442 (vw), 445 (vw), 451 (vw), 456 (vw), 466 (vw), 475 (vw), 481 (vw), 487 (vw), 493 (vw), 501 (vw), 509 (vw), 515 (vw), 526 (vw), 530 (vw), 539 (vw), 546 (vw), 558 (vw), 565 (vw), 575 (vw), 588 (vw), 613 (vw), 623 (vw), 668 (vw), 682 (vw), 692 (vw), 707 (vw), 732 (vw), 751 (vw), 755 (vw), 761 (vw), 773 (vw), 799 (vw), 833 (w), 856 (m), 910 (vw), 919 (w), 947 (vw), 951 (vw), 967 (vw), 1,012 (m), 1,031 (w), 1,056 (vw), 1,076 (vw), 1,106 (vw), 1,136 (vw), 1,158 (vw), 1,172 (vw), 1,193 (vw), 1,232 (vw), 1,242 (vw), 1,274 (vw), 1,290 (vw), 1,310 (vw), 1,340 (w), 1,373 (m), 1,431 (m), 1,485 (w), 1,515 (vw), 1,520 (vw), 1,539 (vw), 1,552 (vw), 1,559 (vw), 1,577 (vw), 1,636 (vw), 2,133 (w), 2,718 (w), 2,845 (vs), 2,886 (vs), 2,960 (m).

Data for [Pr(Cp*)$_2$(I)(THF)] (1Pr). PrI_3 (80 mg, 153 μmol), KCp* (60 mg, 344 μmol, 2.2 equiv.). Very pale green/yellow solution and 1 crop of very pale green/colorless planks (yield = 44.9 mg, 48% assuming [Pr(Cp*)$_2$(I)(THF)$_n$] *n* = 1; 54% if *n* = 0). Elemental analysis *n* = 1 calc. (%): C = 47.23, H = 6.28; *n* = 0 calc. (%): C = 44.63, H = 5.62; found (%): C = 43.08, H = 6.14.

^{1}H NMR (D_6-benzene + 1 drop (12 mg) $[H]_8$-THF, 400.13 MHz, 298 K): δ = 1.42 (s, THF 3,4-(C*H*$_2$)), 3.55 (s, THF 2,5-(C*H*$_2$)), 11.94 (s, 30 H, Cp* C_5(C*H*$_3$)$_5$). Magnetic moment (Evans method, D_6-benzene + 1 drop (12 mg) $[H]_8$-THF, 298 K): μ_{eff} = 3.48 μB.

UV-vis-NIR (THF): λ_{max} (cm^{-1}; *ε*) = 458 (21,834, 6), 463 (21,598, 6), 467 (21,413, 10), 470 (21,277, 8), 472 (21,186, 7), 475 (21,053, 7), 481 (20,790, 36), 485 (20,619, 8), 485 (20,619, 8), 487 (20,534, 5), 496 (20,161, 26), 601 (16,639, 2), 1,418 (7,052, 6), 1,480 (6,757, 10), 1,490 (6,711, 7), 1,502 (6,658, 4), 1,516 (6,596, 4), 1,531 (6,532, 3), 1,574 (6,353, 2).

FT-IR (ATR, microcrystalline): cm^{-1} = 359 (vw), 369 (vw), 373 (vw), 379 (vw), 387 (vw), 400 (vw), 407 (vw), 411 (vw), 414 (vw), 425 (vw), 440 (vw), 444 (vw), 452 (vw), 459 (vw), 465 (vw), 474 (vw), 486 (vw), 491 (vw), 504 (vw), 512 (vw), 519 (vw), 527 (vw), 532 (vw), 536 (vw), 547 (vw), 559 (vw),

588 (vw), 603 (vw), 612 (vw), 623 (vw), 633 (vw), 640 (vw), 646 (vw), 668 (w), 692 (vw), 702 (vw), 714 (vw), 726 (vw), 735 (vw), 742 (vw), 760 (vw), 782 (vw), 801 (w), 833 (m), 857 (m), 910 (w), 919 (w), 949 (w), 1,011 (s), 1,032 (w), 1,056 (vw), 1,091 (vw), 1,135 (vw), 1,158 (vw), 1,172 (w), 1,216 (vw), 1,231 (w), 1,242 (w), 1,276 (vw), 1,290 (w), 1,310 (w), 1,339 (w), 1,373 (m), 1,430 (m), 1,468 (w), 1,485 (w), 1,520 (vw), 1,527 (vw), 1,533 (vw), 2,716 (w), 2,845 (vs), 2,886 (vs), 2,961 (m).

Data for [Nd(Cp*)$_2$(I)(THF)] (1Nd). NdI_3 (80 mg, 152 μmol), KCp* (60 mg, 344 μmol, 2.2 equiv.). Sky-blue solution and 1 crop of blue planks (yield = 39.8 mg, 43% assuming [Nd(Cp*)$_2$(I)(THF)$_n$] n = 1; 48% if n = 0). Elemental analysis n = 1 calc. (%): C = 46.97, H = 6.24; n = 0 calc. (%): C = 44.35, H = 5.58; found (%): C = 40.53, H = 5.78.

^{1}H NMR (D_6-benzene + 1 drop (12 mg) [H]$_8$-THF, 400.13 MHz, 298 K): δ = 1.43 (s, THF 3,4-(C$\underline{H}_2$)), 3.56 (s, THF 2,5-(C$\underline{H}_2$)), 11.13 (s, 30 H, Cp* C_5(C$\underline{H}_3$)$_5$). Magnetic moment (Evans method, D_6-benzene + 1 drop (12 mg) [H]$_8$-THF, 298 K): μ_{eff} = 3.58 μB.

UV-vis-NIR (THF): λ_{max} (cm^{-1}; ε) = 436 (22,936, 2), 439 (22,779, 2), 443 (22,573, 1), 443 (22,573, 2), 461 (21,692, 2), 464 (21,552, 2), 468 (21,368, 2), 476 (21,008, 2), 483 (20,704, 3), 506 (19,763, 2), 514 (19,455, 6), 518 (19,305, 3), 522 (19,157, 5), 525 (19,048, 8), 529 (18,904, 16), 530 (18,868, 6), 533 (18,762, 10), 536 (18,657, 7), 538 (18,587, 6), 540 (18,519, 7), 543 (18,416, 5), 546 (18,315, 4), 550 (18,182, 5), 552 (18,116, 3), 553 (18,083, 3), 560 (17,857, 1), 566 (17,668, 1), 581 (17,212, 8), 586 (17,065, 29), 592 (16,892, 75), 596 (16,779, 171), 598 (16,722, 519), 602 (16,611, 102), 604 (16,556, 94), 609 (16,420, 36), 611 (16,367, 26), 617 (16,207, 20), 673 (14,859, 1), 681 (14,684, 4), 685 (14,599, 2), 689 (14,514, 1), 735 (13,605, 1), 740 (13,514, 4), 742 (13,477, 5), 744 (13,441, 6), 746 (13,405, 19), 750 (13,333, 7), 751 (13,316, 19), 760 (13,158, 5), 766 (13,055, 5), 769 (13,004, 8), 775 (12,903, 3), 780 (12,821, 6), 785 (12,739, 2), 792 (12,626, 3), 795 (12,579, 3), 800 (12,500, 24), 803 (12,453, 4), 806 (12,407, 6), 810 (12,346, 4), 814 (12,285, 6), 818 (12,225, 6), 823 (12,151, 3), 826 (12,107, 4), 840 (11,905, 3), 871 (11,481, 1), 884 (11,312, 4), 894 (11,186, 2), 898 (11,136, 1), 907 (11,025, 1), 919 (10,881, 1), 922 (10,846, 1).

FT-IR (ATR, microcrystalline): cm^{-1} = 359 (vw), 366 (vw), 370 (vw), 374 (vw), 381 (vw), 387 (vw), 402 (vw), 415 (vw), 421 (vw), 425 (vw), 435 (vw), 440 (vw), 445 (vw), 452 (vw), 460 (vw), 465 (vw),

471 (vw), 475 (vw), 479 (vw), 486 (vw), 497 (vw), 504 (vw), 510 (vw), 514 (vw), 521 (vw), 526 (vw), 532 (vw), 542 (vw), 547 (vw), 552 (vw), 560 (vw), 570 (vw), 579 (vw), 589 (vw), 612 (vw), 624 (vw), 634 (vw), 640 (vw), 646 (vw), 653 (vw), 670 (vw), 692 (vw), 702 (vw), 709 (vw), 716 (vw), 725 (vw), 728 (vw), 738 (vw), 745 (vw), 759 (vw), 762 (vw), 784 (vw), 800 (vw), 834 (m), 856 (m), 895 (vw), 909 (w), 920 (w), 949 (vw), 965 (vw), 1,011 (s), 1,032 (w), 1,056 (vw), 1,078 (vw), 1,135 (vw), 1,159 (vw), 1,171 (w), 1,206 (vw), 1,231 (vw), 1,241 (w), 1,276 (vw), 1,290 (vw), 1,310 (w), 1,339 (w), 1,373 (m), 1,431 (m), 1,468 (w), 1,485 (w), 1,517 (vw), 1,521 (vw), 1,528 (vw), 1,534 (vw), 1,553 (vw), 2,716 (w), 2,846 (vs), 2,887 (vs), 2,932 (m), 2,958 (m).

Synthesis of [Ln(Cp)$_2$(I)(I^{Me4})] (**2Ln**, Ln = La, Ce, Pr, Nd, Y)*

All [Ln(Cp*)$_2$(I)(I^{Me4})] (**2Ln**; Ln = La, Ce, Pr, Nd, Y) complexes were synthesized in an identical fashion and so a procedure is given for **2La**, along with characterization details for the others. Given the unreliability of obtaining a given THF-content in the **1Ln** (Ln = La, Ce, Pr, Nd) precursors we have prepared these materials *in situ* followed by reaction with solid I^{Me4}.

Synthesis of [La(Cp*)$_2$(I)(I^{Me4})] (2La). THF (1.5 mL) was added to solid LaI_3 (80 mg, 154 μmol) in a 20 mL glass scintillation vial with a Teflon-coated stirrer bar, followed by solid KCp* (60 mg, 344 μmol, 2.2 equiv.). The colorless suspension was heated and stirred to a very gentle boil in the sealed vial for 45 minutes and then allowed to cool to room temperature, to afford a colorless solution with a white precipitate. The volatiles were removed *in vacuo*, and the flowing white powder was suspended in toluene (1.5 mL) and 1 drop (12 mg) of THF was added. The suspension was stirred for 45 minutes with modest heating (hot plate set to 50 °C), allowed to cool to room temperature, and then centrifuged (5,000 rpm, 10 minutes) which gave a pale straw-colored solution with a small amount of white powder. The solution was filtered into a 20 mL glass scintillation vial through 2 half-discs of glass microfiber mounted in a glass pipette. A Teflon-coated stirrer bar was added, followed by solid I^{Me4} (20 mg, 161 μmol, 1.05 equiv.). The pale yellow solution was gently stirred for 10 minutes which gave a small quantity of microcrystalline material on the vial walls. The solution was

filtered into a 4 mL glass vial through 2 half-discs of glass microfiber mounted in a glass pipette and then concentrated slowly well beyond the point of incipient crystallization (*ca.* 0.5 mL end point). The vial was then heated strongly (hot plate set to 130 °C) until all the solids had redissolved and allowed to cool to room temperature and stand for 1 hour before storage at –35 °C overnight (16 hours). A modest crop of colorless planks formed, from which the pale yellow supernatant was decanted and then the crystals were washed with hexane (2 × 2 mL) and dried *in vacuo* for 1 hour (yield = 40.0 mg, 40%). Elemental analysis calc. (%): C = 49.10, H = 6.41, N = 4.24; found (%): C = 47.73, H = 6.77, N = 4.47.

^{1}H NMR (D_6-benzene, 400.13 MHz, 298 K): δ = 1.21 (s, 3 H, I^{Me4} C(C$\underline{\mathit{H}}_3$)), 1.36 (s, 3 H, I^{Me4} C(C$\underline{\mathit{H}}_3$)), 2.17 (s, 30 H, Cp* C_5(C$\underline{\mathit{H}}_3$)$_5$), 2.99 (s, 3 H, I^{Me4} N(C$\underline{\mathit{H}}_3$)), 3.54 (s, 3 H, I^{Me4} N(C$\underline{\mathit{H}}_3$)). ^{13}C{^{1}H} NMR (D_6-benzene, 100.62 MHz, 298 K): δ = 8.14 (I^{Me4} C($\underline{\mathit{C}}$H$_3$)), 8.17 (I^{Me4} C($\underline{\mathit{C}}$H$_3$)), 12.51 (Cp* C_5($\underline{\mathit{C}}$H$_3$)$_5$), 35.87 (I^{Me4} N($\underline{\mathit{C}}$H$_3$)), 37.17 (I^{Me4} N($\underline{\mathit{C}}$H$_3$)), 120.53 (Cp* $\underline{\mathit{C}}_5$(CH$_3$)$_5$), 124.78 (I^{Me4} C($\underline{\mathit{C}}$H$_3$)), 126.06 (I^{Me4} C($\underline{\mathit{C}}$H$_3$)) ppm. We could not locate the carbenic C, presumable due to quadrupolar broadening from the 99.9% abundant ^{139}La I = 7/2.

UV-vis-NIR (THF): A featureless peak extends from ~390 nm (25,640 cm^{-1}) into the UV region, and beyond our spectral range.

FT-IR (ATR, microcrystalline): cm^{-1} = 366 (w), 375 (w), 382 (w), 411 (vw), 420 (vw), 430 (vw), 443 (vw), 450 (vw), 462 (vw), 471 (vw), 481 (vw), 489 (vw), 501 (vw), 519 (vw), 527 (vw), 545 (vw), 567 (vw), 590 (vw), 610 (vw), 624 (vw), 636 (vw), 649 (vw), 671 (vw), 692 (vw), 722 (w), 742 (vw), 764 (vw), 773 (vw), 798 (vw), 810 (vw), 839 (w), 877 (vw), 887 (vw), 902 (vw), 911 (vw), 943 (vw), 961 (vw), 1,016 (w), 1,047 (w), 1,055 (w), 1,074 (w), 1,093 (vw), 1,100 (vw), 1,112 (vw), 1,134 (vw), 1,157 (vw), 1,168 (vw), 1,175 (vw), 1,203 (vw), 1,216 (w), 1,243 (vw), 1,289 (w), 1,362 (s), 1,395 (m), 1,420 (s), 1,430 (s), 1,470 (w), 1,490 (w), 1,515 (vw), 1,536 (vw), 1,555 (vw), 1,572 (vw), 1,599 (vw), 1,634 (w), 1,649 (w), 1,679 (vw), 2,716 (w), 2,748 (w), 2,846 (vs), 2,888 (vs), 2,950 (m), 2,962 (m), 2,997 (w).

Several crystals were obtained as a second crop from the toluene supernatent which contained a toluene solvate, [La(Cp*)$_2$(I)(I^{Me44})]·(C_7H_8) (**2La$^{\beta}$**) – no analysis was conducted on this material as all was consumed as part of the single-crystal X-ray diffraction study.

Data for [Ce(Cp*)$_2$(I)(I^{Me4})] (2Ce). CeI$_3$ (80 mg, 154 μmol), KCp* (60 mg, 344 μmol, 2.2 equiv.), I^{Me4} (22 mg, 177 μmol, 1.15 equiv.). Orange, slightly fluorescent, solution and 1 crop of orange planks. Yield = 40.7 mg, 40%). Elemental analysis calc. (%): C = 49.01, H = 6.40, N = 4.23; found (%): C = 48.79; H = 6.53, N = 4.23.

^{1}H NMR (D$_6$-benzene, 400.13 MHz, 298 K): δ = −23.68 (s, 3 H, I^{Me4} C(C$\underline{H}_3$)), −19.34 (s, 3 H, I^{Me4} C(C$\underline{H}_3$)), −3.52 (s, 3 H, I^{Me4} N(C$\underline{H}_3$)), −3.23 (s, 3 H, I^{Me4} N(C$\underline{H}_3$)), 6.10 (s, 30 H, Cp* C$_5$(C$\underline{H}_3$)$_5$).

Magnetic moment (Evans method, D$_6$-benzene, 298 K): μ_{eff} = 2.48 μB.

UV-vis-NIR (THF): λ_{max} (cm^{-1}; ε) = 439 (22,779, 78), 492 (20,325, 379).

FT-IR (ATR, microcrystalline): cm^{-1} = 364 (vw), 374 (w), 396 (vw), 408 (vw), 413 (vw), 420 (vw), 429 (vw), 438 (vw), 445 (vw), 463 (vw), 473 (vw), 478 (vw), 481 (vw), 489 (vw), 496 (vw), 500 (vw), 509 (vw), 519 (vw), 523 (vw), 526 (vw), 534 (vw), 545 (vw), 552 (vw), 561 (vw), 568 (vw), 574 (vw), 590 (vw), 597 (vw), 609 (vw), 613 (vw), 622 (vw), 631 (vw), 638 (vw), 651 (vw), 668 (vw), 676 (vw), 692 (vw), 722 (w), 744 (vw), 748 (vw), 755 (vw), 766 (vw), 779 (vw), 784 (vw), 799 (vw), 812 (vw), 840 (w), 856 (vw), 866 (vw), 869 (vw), 877 (vw), 884 (vw), 902 (vw), 910 (vw), 918 (vw), 944 (vw), 958 (vw), 962 (vw), 970 (vw), 976 (vw), 980 (vw), 1,017 (w), 1,047 (vw), 1,055 (vw), 1,074 (vw), 1,093 (vw), 1,101 (vw), 1,119 (vw), 1,128 (vw), 1,133 (vw), 1,159 (vw), 1,175 (vw), 1,181 (vw), 1,192 (vw), 1,198 (vw), 1,203 (vw), 1,216 (vw), 1,288 (w), 1,362 (s), 1,373 (m), 1,395 (m), 1,420 (s), 1,431 (s), 1,470 (w), 1,492 (w), 1,633 (s), 1,648 (w), 2,129 (w), 2,715 (w), 2,848 (vs), 2,886 (vs), 2,962 (m), 2,999 (w).

Several crystals were obtained as a second crop from the toluene supernatent which contained a toluene solvate, [Ce(Cp*)$_2$(I)(I^{Me44})]·(C$_7$H$_8$) (**2Ce$^{\beta}$**) – no analysis was conducted on this material as all was consumed as part of the single-crystal X-ray diffraction study.

Data for [Pr(Cp*)$_2$(I)(I^{Me4})] (2Pr). PrI$_3$ (80 mg, 153 μmol), KCp* (60 mg, 344 μmol, 2.2 equiv.), I^{Me4} (20 mg, 161 μmol, 1.05 equiv.). Very pale green/yellow solution and 1 crop of very pale green/colorless planks. Yield = 37.0 mg, 37%). Elemental analysis calc. (%): C = 48.95, H = 6.39, N = 4.23; found (%): C = 47.81, H = 6.46, N = 4.38.

^{1}H NMR (D_6-benzene, 400.13 MHz, 298 K): δ = −70.81 (s, 3 H, I^{Me4} C(C*H*$_3$)), −47.13 (s, 3 H, I^{Me4} C(C*H*$_3$)), −13.10 (s, 3 H, I^{Me4} N(C*H*$_3$)), −10.59 (s, 3 H, I^{Me4} N(C*H*$_3$)), 13.61 (s, 30 H, Cp* C_5(C*H*$_3$)$_5$).

Magnetic moment (Evans method, D_6-benzene, 298 K): μ_{eff} = 3.19 μB.

UV-vis-NIR (THF): λ_{max} (cm^{-1}; ε) = 452 (22,124, 4), 460 (21,739, 6), 469 (21,322, 11), 474 (21,097, 8), 478 (20,921, 8), 482 (20,747, 31), 486 (20,576, 11), 494 (20,243, 5), 498 (20,080, 19), 511 (19,569, 3), 516 (19,380, 3), 597 (16,750, 1), 603 (16,584, 2), 1,433 (6,978, 7), 1,482 (6,748, 7), 1,498 (6,676, 11), 1,509 (6,627, 7), 1,520 (6,579, 5), 1,540 (6,494, 6), 1,579 (6,333, 4).

FT-IR (ATR, microcrystalline): cm^{-1} = 355 (vw), 364 (vw), 367 (vw), 370 (vw), 376 (vw), 383 (vw), 398 (vw), 408 (vw), 413 (vw), 418 (vw), 424 (vw), 432 (vw), 439 (vw), 445 (vw), 452 (vw), 463 (vw), 476 (vw), 480 (vw), 488 (vw), 493 (vw), 499 (vw), 512 (vw), 522 (vw), 538 (vw), 544 (vw), 551 (vw), 568 (vw), 574 (vw), 589 (vw), 610 (vw), 624 (vw), 638 (vw), 656 (vw), 662 (vw), 674 (vw), 692 (vw), 704 (vw), 722 (w), 771 (vw), 785 (vw), 796 (vw), 801 (vw), 809 (vw), 840 (w), 883 (vw), 909 (vw), 920 (vw), 943 (vw), 962 (vw), 1,016 (w), 1,047 (vw), 1,056 (vw), 1,075 (w), 1,093 (vw), 1,103 (vw), 1,113 (vw), 1,133 (vw), 1,158 (vw), 1,174 (vw), 1,184 (vw), 1,203 (vw), 1,218 (vw), 1,288 (w), 1,362 (s), 1,395 (m), 1,420 (s), 1,431 (s), 1,471 (w), 1,490 (w), 1,523 (vw), 1,553 (vw), 1,560 (vw), 1,580 (vw), 1,599 (vw), 1,617 (vw), 1,635 (w), 1,649 (w), 1,663 (vw), 2,410 (w), 2,716 (w), 2,745 (w), 2,751 (w), 2,787 (w), 2,847 (vs), 2,885 (vs), 2,963 (m), 3,002 (w).

Data for [Nd(Cp*)$_2$(I)(I^{Me4})] (2Nd). NdI_3 (80 mg, 152 μmol), KCp* (60 mg, 344 μmol, 2.2 equiv.), I^{Me4} (20 mg, 161 μmol, 1.06 equiv.). Sky-blue solution and 1 crop of blue planks. Yield = 40.0 mg, 39%). Elemental analysis calc. (%): C = 48.71, H = 6.36, N = 4.21; found (%): C = 47.17, H = 6.86, N = 4.32.

^{1}H NMR (D_6-benzene, 400.13 MHz, 298 K): δ = −33.77 (s, 3 H, I^{Me4} C(C*H*$_3$)), −25.74 (s, 3 H, I^{Me4} C(C*H*$_3$)), −5.43 (s, 3 H, I^{Me4} N(C*H*$_3$)), −4.70 (s, 3 H, I^{Me4} N(C*H*$_3$)), 11.82 (s, 30 H, Cp* C_5(C*H*$_3$)$_5$).

Magnetic moment (Evans method, D_6-benzene, 298 K): μ_{eff} = 3.52 μB.

UV-vis-NIR (THF): λ_{max} (cm^{-1}; ε) = 439 (22,779, 3), 469 (21,322, 3), 483 (20,704, 3), 515 (19,417, 5), 519 (19,268, 5), 522 (19,157, 7), 525 (19,048, 8), 530 (18,868, 14), 532 (18,797, 5), 534 (18,727, 8), 536 (18,657, 8), 538 (18,587, 6), 540 (18,519, 11), 543 (18,416, 7), 545 (18,349, 7), 548 (18,248,

5), 552 (18,116, 4), 560 (17,857, 2), 583 (17,153, 8), 587 (17,036, 23), 590 (16,949, 14), 592 (16,892, 39), 593 (16,863, 55), 597 (16,750, 203), 599 (16,694, 371), 602 (16,611, 124), 604 (16,556, 123), 608 (16,447, 31), 613 (16,313, 14), 614 (16,287, 19), 616 (16,234, 15), 675 (14,815, 2), 683 (14,641, 5), 685 (14,599, 3), 689 (14,514, 2), 738 (13,550, 3), 742 (13,477, 5), 744 (13,441, 7), 747 (13,387, 15), 750 (13,333, 8), 752 (13,298, 20), 755 (13,245, 5), 759 (13,175, 7), 762 (13,123, 7), 767 (13,038, 9), 772 (12,953, 4), 778 (12,853, 7), 789 (12,674, 3), 794 (12,594, 3), 797 (12,547, 3), 802 (12,469, 19), 804 (12,438, 6), 806 (12,407, 5), 814 (12,285, 5), 819 (12,210, 7), 824 (12,136, 5), 831 (12,034, 4), 838 (11,933, 4), 846 (11,820, 2), 874 (11,442, 2), 885 (11,299, 5), 893 (11,198, 1), 898 (11,136, 2), 905 (11,050, 2), 917 (10,905, 1).

FT-IR (ATR, microcrystalline): cm^{-1} = 354 (vw), 360 (vw), 366 (vw), 377 (vw), 383 (vw), 402 (vw), 412 (vw), 418 (vw), 424 (vw), 437 (vw), 445 (vw), 452 (vw), 463 (vw), 475 (vw), 480 (vw), 485 (vw), 493 (vw), 500 (vw), 506 (vw), 518 (vw), 525 (vw), 538 (vw), 544 (vw), 567 (vw), 575 (vw), 590 (vw), 608 (vw), 624 (vw), 631 (vw), 640 (vw), 651 (vw), 656 (vw), 669 (vw), 692 (vw), 722 (w), 750 (vw), 755 (vw), 769 (vw), 784 (vw), 800 (vw), 840 (w), 858 (vw), 868 (vw), 888 (vw), 895 (vw), 900 (vw), 904 (vw), 945 (vw), 963 (vw), 989 (vw), 1,016 (w), 1,047 (vw), 1,055 (vw), 1,075 (w), 1,094 (vw), 1,101 (vw), 1,114 (vw), 1,133 (vw), 1,157 (vw), 1,173 (vw), 1,218 (w), 1,362 (s), 1,374 (m), 1,395 (m), 1,421 (s), 1,431 (s), 1,471 (w), 1,490 (w), 1,515 (vw), 1,534 (vw), 1,548 (vw), 1,553 (vw), 1,565 (vw), 1,571 (vw), 1,590 (vw), 1,599 (vw), 1,636 (w), 1,649 (w), 1,662 (vw), 1,675 (vw), 1,679 (vw), 1,684 (vw), 1,690 (vw), 1,694 (vw), 2,716 (w), 2,751 (w), 2,790 (w), 2,845 (vs), 2,883 (vs), 2,948 (m), 2,963 (m), 3,003 (w).

Data for [Y(Cp*)$_2$(I)(I^{Me4})] (2Y). YI_3 (76 mg, 162 μmol), KCp* (60 mg, 344 μmol, 2.1 equiv.), I^{Me4} (22mg, 177 μmol, 1.1 equiv.). Pale yellow solution and 1 crop of colorless blocks. Yield = 31.9 mg, 32%). Elemental analysis calc. (%): C = 53.12, H = 6.94, N = 4.59; found (%): C = 52.00, H = 6.96, N = 4.48.

^{1}H NMR (D$_6$-benzene, 400.13 MHz, 298 K): δ = 1.21 (s, 3 H, I^{Me4} C(C$\underline{H}_3$)), 1.35 (s, 3 H, I^{Me4} C(C$\underline{H}_3$)), 2.09 (s, 30 H, Cp* C$_5$(C$\underline{H}_3$)$_5$), 2.91 (s, 3 H, I^{Me4} N(C$\underline{H}_3$)), 3.66 (s, 3 H, I^{Me4} N(C$\underline{H}_3$)). ^{13}C{^{1}H} NMR (D$_6$-benzene, 100.62 MHz, 298 K): δ = 8.20 (I^{Me4} C($\underline{C}$H$_3$)), 8.42 (I^{Me4} C($\underline{C}$H$_3$)), 13.37 (Cp* C$_5$($\underline{C}$H$_3$)$_5$),

36.26 (I^{Me4} N($\underline{C}H_3$)), 38.14 (I^{Me4} N($\underline{C}H_3$)), 118.90 (d, $^1J_{CY}$ = 1.3 Hz, Cp* $\underline{C}_5(CH_3)_5$), 124.66 (I^{Me4} C($\underline{C}H_3$)), 126.48 (I^{Me4} C($\underline{C}H_3$)), 192.93 (d, $^1J_{CY}$ = 49 Hz, I^{Me4} carbenic C) ppm. The coupling constants are in accord with those from similar complexes.[9]

UV-vis-NIR (THF): A featureless peak extends from ~370 nm (27,030 cm^{-1}) into the UV region, and beyond our spectral range.

FT-IR (ATR, microcrystalline): cm^{-1} = 364 (w), 375 (w), 386 (w), 421 (vw), 463 (vw), 545 (vw), 568 (vw), 591 (w), 605 (vw), 625 (vw), 631 (vw), 657 (vw), 692 (vw), 717 (w), 722 (w), 793 (vw), 801 (vw), 841 (w), 878 (vw), 905 (vw), 944 (vw), 1,017 (w), 1,045 (w), 1,055 (vw), 1,076 (vw), 1,092 (vw), 1,121 (w), 1,152 (vw), 1,159 (vw), 1,175 (vw), 1,216 (vw), 1,290 (w), 1,358 (s), 1,374 (m), 1,395 (m), 1,422 (s), 1,431 (s), 1,472 (w), 1,493 (w), 1,600 (w), 1,640 (s), 1,671 (vw), 2,706 (w), 2,713 (w), 2,754 (w), 2,847 (vs), 2,884 (vs), 2,907 (s), 2,929 (s), 2,963 (m), 3,015 (w).

Synthesis of [Ce(Cp)$_2$(I)(I^{Me4})] (**2Ce**) from I^{Me4} and "Ce(Cp*)$_2$(I) (**3**)"*

A prior report on the synthesis of [M(Cp*)$_2$(I)(I^{Me4})] (M = U, **2U**; Ce, **2Ce**) detailed the use of a sonicator in the synthesis of **2Ce** from Lewis-base free "Ce(Cp*)$_2$(I)"[10] and I^{Me4}, whereas [U(Cp*)$_2$(I)(py)] (**1U**, py = pyridine) was used for U and no sonication was needed.[11] For **2Ce**, this report described essentially spontaneous formation of crystals of the complex from the reaction medium apparently under sonication conditions. In order to optimize a synthetic route to **2Np** and **2Pu** that used our transuranium resources efficiently, we had account for our inability to use a sonnicator in the event of low product or precursor solubility (*vide infra*), and for the likely differing lability of THF in the **1M** (M = La–Nd, Y, U–Pu) complexes.

Alternative synthesis of [Ce(Cp*)$_2$(I)(THF)] (1Ce), and of "Ce(Cp*)$_2$(I)" (3). THF (4 mL) was added to a mixture of CeI_3 (97.5 mg, 187 μmol) and KCp* (66 mg, 378 μmol, 2.02 equiv.) in a 20 mL glass scintillation vial with a Teflon-coated stirrer bar at room temperature. The mixture immediately began to turn fluorescent orange, and was stirred for 2 hours at room temperature. The cloudy orange mixture was reduced to dryness *in vacuo* to give an orange powder – note a previous report describes isolating a pink powder.[10] Toluene (3 mL) and THF (3 drops) were added to the

solids and stirred for 5 minutes. The orange suspension was centrifuged (5,000 rpm, 10 minutes), and filtered into a 20 mL glass scintillation vial through 2 half-discs of glass microfiber mounted in a glass pipette. *The fluorescence of the reaction mixture was sufficiently intense due to the glovebox lighting that it was difficult to determine whether the solution was initially free of suspended solids.* Slow concentration of this solution *in vacuo* gave crystals of orange [Ce(Cp*)$_2$(I)(THF)] (**1Ce**, **Figure S5-L**) in apparent quantitative yield (w.r.t. CeI_3 starting material) as dry free flowing crystals (**Figure S5-R**) after a hexane wash (2 × 2 mL) and subsequent drying *in vacuo*. The crystal structure of this complex was determined from several of these crystals for the first time. However, we noted that numerous pink flecks were interspersed with the orange crystals. When the orange crystals were redissolved in toluene (2 mL), at first a clear fluorescent orange solution was formed, but upon standing this slowly deposited a fine precipitate of fluorescent pink powder, presumed to be **3** by comparison to prior work.[10] The pink solids were isolated, washed with hexane (2 × 4 mL) and dried *in vacuo* to give a free flowing fluorescent pink powder (yield 93 mg, 92% w.r.t. CeI_3 starting material and assuming the formulation as **3** – no further analysis was attempted on this material as we typically used **1Ce** prepared *in situ* for further reactions).

Complex **3** is likely dimeric, evidenced by its low solubility and ready displacement of THF.[12] We note that the isolation of both solvated and unsolvated Ce(Cp^R)$_2$(I) complexes using the same Cp^R ligand is fairly common,[12] though few ligand sets are amenable to structural characterization in both forms as either 1) the Lewis-base adduct is too soluble to easily crystalize, or 2) the unsolvated form is dimeric and extremely insoluble, as is the case here using Cp*. We have found that stirring a suspension of blue **1Np** in hexane affords an emerald-green powder, insoluble in toluene, which readily redissolves in THF to re-form a blue solution.

Synthesis of [Ce(Cp*)$_2$(I)(I^{Me4})] (2Ce) from 3. Toluene (1 mL) was added to solid **3** (20 mg, 37 μmol) in a 20 mL scintillation vial with a Teflon-coated stirrer bar. This formed a pink suspension, where the solution had little coloration. THF (2 drops, 24 mg) was added to the suspension which rapidly turned orange, and most of the solid dissolved. A solution of I^{Me4} (4.6 mg, 37 μmol) in toluene

(0.5 mL) was added to the mixture, which caused further dissolution to afford a slightly turbid fluorescent orange solution. The solution was manually agitated occasionally for 20 minutes then filtered into a 4 mL glass vial through 2 half-discs of glass microfiber mounted in a glass pipette. The solution was concentrated *in vacuo* slightly past the point of incipient crystallization, and left to stand at room temperature for 1 hour. A large crop of bright yellow crystals (**Figure S7**) of $[Ce(Cp^*)_2(I)(I^{Me4})]$ (**2Ce**) was isolated from this. Storage of the supernatant at –35 °C for 16 hours gave a second crop. These crystalline crops were separately washed with hexane (2 × 2 mL) and dried *in vacuo* for 1 hour (combined yield 19.7 mg, 80% w.r.t. **3** starting material assuming the composition as "$Ce(Cp^*)_2(I)$" for **3**).

Synthesis of $[An(Cp^*)_2(I)(I^{Me4})]$ *(An = Np,* ***2Np****; Pu,* ***2Pu****)*

Synthesis of $[Np(Cp^*)_2(I)(I^{Me4})]$ (2Np). THF (1.5 mL) was added to solid $[NpI_3(THF)_4]$ (34.8 mg, 38.4 μmol) in a 20 mL scintillation vial with a Teflon-coated stirrer bar. This formed an orange solution with some suspended orange solids – $[NpI_3(THF)_4]$ is not very soluble in THF. The suspension was cooled to –35 °C, then solid KCp* (13.4 mg, 77 μmol, 2.0 equiv.) was added with stirring, which immediately caused a color change to purple. The mixture was stirred for 1 hour during which time is slowly turned blue/purple. The volatiles were removed *in vacuo*, which left a blue powder. Toluene (1 mL) and THF (3 drops, 36 mg) were added to the solids, and stirred for 2 minutes. The mixture was filtered into a 4 mL glass vial through 2 half-discs of glass microfiber mounted in a glass pipette. The slightly blue solids on the filter discs were washed with additional toluene (~0.5 mL) which afforded a dark blue solution. Slow concentration of this solution to dryness afforded crude $[Np(Cp^*)_2(I)(THF)]$ (25.3 mg, 36 μmol, **1Np**) as blue/black crystals. *A previous report of this complex describes solutions as purple rather than blue, which appears to be a concentration effect. In both cases, single crystals appear blue/black.* If pure, this would equate to a 93% yield. A solution of I^{Me4} (4.5 mg, 36 μmol) in toluene (0.5 mL) was added to a clear blue/black solution (**Figure S10-L**) of **1Np** prepared from the crude crystalline material above in toluene (0.5 mL). A slight color change to teal/black was observed (**Figure S10-R**). The solution was agitated

occasionally for 20 minutes then filtered into a 4 mL glass vial through 2 half-discs of glass microfiber mounted in a glass pipette. The solution was concentrated *in vacuo* slightly past the point of incipient crystallization, and left to stand at room temperature for 1 hour. A modest crop of teal crystals of [Np(Cp*)$_2$(I)(I^{Me4})] (**2Np**) was isolated from this. Storage of the supernatant at –35 °C for 16 hours gave a second crop. These crystalline crops were separately washed with hexane (2 × 2 mL) and dried *in vacuo* for 1 hour (combined yield 8.4 mg, 31% w.r.t. [NpI$_3$(THF)$_4$] starting material).

^{1}H NMR (D$_6$-benzene, 400.13 MHz, 298 K): δ = 0.18 (s, 3 H, I^{Me4} C(C$\underline{H}_3$)), 0.54 (s, 3 H, I^{Me4} C(C$\underline{H}_3$)), 0.77 (s, 30 H, Cp* C$_5$(C$\underline{H}_3$)$_5$), 2.84 (s, 3 H, I^{Me4} N(C$\underline{H}_3$)), 4.27 (s, 3 H, I^{Me4} N(C$\underline{H}_3$)).

^{13}C{^{1}H} NMR (D$_6$-benzene, 100.62 MHz, 298 K): δ = –67.33 (Cp* C$_5$($\underline{C}$H$_3$)$_5$), –33.69 (I^{Me4} C($\underline{C}$H$_3$)), 0.89 (I^{Me4} C($\underline{C}$H$_3$)), 12.30 (I^{Me4} N($\underline{C}$H$_3$)), 13.20 (I^{Me4} N($\underline{C}$H$_3$)), 171.35 (Cp* $\underline{C}_5$(CH$_3$)$_5$), 166.53, 173.78, 175.02 ppm – we could not determine which of these three peaks corresponds to the 2 × I^{Me4} $\underline{C}$(CH$_3$) peaks, and the carbene peak. No additional resonances were observed at ±600 ppm.

Magnetic moment (Evans method, D$_6$-benzene, 298 K): μ_{eff} = 2.42 μB.

UV-vis-NIR (toluene): λ_{max} (cm^{-1}; ε) = 381 (26,219, 1,640), 503 (19,889, 139), 565 (17,712, 336), 576 (17,373, 345), 605 (16,529, 423), 651 (15,370, 477), 697 (14,343, 194), 795 (12,572, 70), 807 (12,398, 85), 829 (12,063, 151), 851 (11,748, 84), 880 (11,361, 64), 898 (11,133, 70), 916 (10,917, 77), 962 (10,391, 33), 1,027 (9,739, 308), 1,080 (9,256, 22), 1,106 (9,038, 13), 1,123 (8,903, 10), 1,215 (8,233, 34), 1,238 (8,078, 28), 1,360 (7,354, 50), 1,398 (7,153, 150), 1,448 (6,904, 73).

Synthesis of [Pu(Cp*)$_2$(I)(I^{Me4})] (2Pu). The synthesis of this complex was performed analogously to the above. Crude [Pu(Cp*)$_2$(I)(THF)] (**1Pu**) was isolated in essentially quantitative yield as swamp green crystals. No significant color change was observed upon addition of a toluene solution of I^{Me4} to a gold/black (**Figure S13**) toluene solution of **1Pu**. Two crops of green/gold crystals of [Pu(Cp*)$_2$(I)(I^{Me4})] (**2Pu**) were isolated similarly to **2Np**, with one collected at room temperature and a second after storage at –35 °C for 16 hours. These were separately washed with hexane (2 × 2 mL) then dried *in vacuo* for 1 hour (combined yield 12.7 mg, 38% w.r.t. [PuI$_3$(THF)$_4$] starting material).

^1H NMR (D_6-benzene, 400.13 MHz, 298 K): δ = 1.25 (s, 6 H, 2 × I^{Me4} C(C$\underline{H}_3$)), 1.65 (s, 30 H, Cp* C_5(C$\underline{H}_3$)$_5$), 3.94 (s, 3 H, I^{Me4} N(C$\underline{H}_3$)), 4.98 (s, 3 H, I^{Me4} N(C$\underline{H}_3$)).

$^{13}C\{^1H\}$ NMR (D_6-benzene, 100.62 MHz, 298 K): δ = –0.17 (Cp* C_5($\underline{C}H_3$)$_5$), 9.36 (I^{Me4} C($\underline{C}H_3$)), 9.41 (I^{Me4} C($\underline{C}H_3$)), 21.13 (I^{Me4} N($\underline{C}H_3$)), 21.43 (I^{Me4} N($\underline{C}H_3$)), 103.30 (Cp* $\underline{C}_5$(CH_3)$_5$), 136.90 (I^{Me4} C($\underline{C}H_3$)), 139.91 (I^{Me4} C($\underline{C}H_3$)) ppm – we could not conclusively identify the carbene resonance, though note one additional peak at 29.92 ppm was present with comparable intensity to many of the other peaks, and which does not correspond to a known solvent residual signal. No additional resonances were observed at ±600 ppm.

Magnetic moment (Evans method, D_6-benzene, 298 K): μ_{eff} = 1.19 μB.

UV-vis-NIR (toluene): λ_{max} (cm^{-1}; ε) = 1,585 (6,308, 43), 1,564 (6,395, 18), 1,538 (6,502, 15), 1,498 (6,674, 28), 1,457 (6,865, 32), 1,411 (7,087, 42), 1,301 (7,688, 4), 1,218 (8,210, 11), 1,180 (8,475, 90), 1,162 (8,603, 16), 1,140 (8,775, 83), 1,109 (9,020, 22), 1,098 (9,111, 18), 1,067 (9,372, 33), 1,067 (9,376, 33), 1,052 (9,509, 37), 1,037 (9,641, 138), 1,022 (9,783, 101), 1,022 (9,787, 101), 1,008 (9,917, 78), 965 (10,361, 13), 940 (10,634, 104), 926 (10,799, 74), 917 (10,900, 83), 900 (11,111, 40), 865 (11,563, 13), 844 (11,851, 14), 826 (12,104, 40), 810 (12,340, 38), 803 (12,450, 37), 787 (12,703, 83), 777 (12,877, 46), 680 (14,697, 16), 654 (15,286, 20), 642 (15,576, 38), 632 (15,833, 75), 620 (16,139, 114), 609 (16,415, 113), 597 (16,739, 120), 585 (17,088, 140), 574 (17,422, 138), 559 (17,895, 70), 553 (18,077, 61), 537 (18,636, 88), 531 (18,832, 105), 488 (20,509, 501), 476 (21,026, 504), 463 (21,608, 614), 458 (21,815, 643), 448 (22,321, 761), 445 (22,482, 799), 443 (22,573, 780), 439 (22,779, 715), 434 (23,041, 667), 422 (23,708, 483), 385 (25,988, 895).

Synthesis of [Nd(Cp)$_2$(I_xCl_{1-x})(I^{Me4})] (**4Nd**) from $NdCl_3$ dissolved in $HCl_{(aq)}$*

As our Am^{3+} stock is in the form of an aqueous 6 M HCl solution, we had to devise a method to dehydrate this material and form an "AmI_3" precursor. While previous attempts at synthesizing air/moisture-sensitive Am^{3+} complexes have been successful in using Me_3SiCl to produce "$AmCl_3(DME)_n$", we needed an iodide source. Our laboratory was not equipped to perform the high-

temperature dehydration using NH_4I reported by Asprey and co-workers.[13] Instead, we turned to Me_3SiI as a combined halide-exchange and dehydration agent.

Isolation of [Nd(Cp*)$_2$(I_xCl_{1-x})(I^{Me4})] (4Nd). Anhydrous $NdCl_3$ (5.0 mg, 20 μmol) was dissolved in 6 M $HCl_{(aq)}$ (*ca.* 1 mL) in a 20 mL glass scintillation vial with a Teflon-coated stirrer bar. The pale pink solution was reduced to dryness under a stream of UHP Ar gas and brought into a transuranium He inert-atmosphere glovebox by an overnight (16 hour) vacuum cycle. The pale pink solid was suspended in DME (1.5 mL) with manual loosening of solids from the vial wall – the spatula was washed with DME (0.5 mL) – and then heated (hot plate set to 70 °C) for 15 minutes. The pale pink/white suspension was allowed to cool to room temperature where there was no discernible color to the supernatant. At room temperature, Me_3SiI (*ca.* 1.5 mL) was added with stirring which caused the vial to become cloudy with a fine pale blue solid and a pale yellow supernatant. The mixture was stirred at room temperature for 5 minutes, followed by heating (hot plate set to 60 °C) for 1 hour which caused most of the material to dissolve. The mixture was cooled to room temperature, then pentane (10 mL) was added which caused some precipitation of a pale powder. After stirring at room temperature for 2 minutes, the mixture was stored at –35 °C for 1 hour to settle the solids. The slightly turbid pale yellow supernatant was decanted, then Et_2O (1.5 mL) was added and the suspension was stirred for 2 minutes followed by the addition of more pentane (10 mL). The cloudy mixture was stored at –35 °C for 30 minutes and then pale yellow/colorless supernatant was decanted from the pale blue solids. Pentane (5 mL) was added to the pale blue solids followed by stirring for 2 minutes, all the solids were loosened with a spatula, and the suspension was stored at –35 °C for 30 minutes. The colorless supernatant was decanted and the pale blue powder was dried *in vacuo* for 1 hour.

THF (1.5 mL) was added with stirring to the pale blue powder prepared above, which afforded a pale blue solution with only a small amount of pale blue powder undissolved. Solid KCp* (7.5 mg, 43 μmol, 2.1 equiv.) was added to the stirred mixture which immediately became more cloudy and the blue coloration intensified. The mixture was stirred for 1 hour and then the volatiles were

removed *in vacuo* to afford a blue powder. Toluene (1.5 mL) was added followed by THF (2 drops, 24 mg), and the suspension was stirred for 10 minutes. The blue solution was filtered into a fresh 20 mL glass scintillation vial through 2 half-discs of glass microfiber mounted in a glass pipette, and a Teflon-coated stirrer bar was added. *Note the filter pipette was not rinsed with additional solvent*. The blue solution was stirred gently and solid I^{Me4} (2.6 mg, 21 μmol, 1.05 equiv.) was added which did not lead to any noticeable color change. The stir-rate was increased, and over the course of 10 minutes a very small amount of microcrystalline material formed. The blue solution was filtered into a 4 mL glass vial through 2 half-discs of glass microfiber mounted in a glass pipette and then concentrated to *ca.* 0.1 mL – the volume was small enough that the vial had to be stored at an angle so as to have all the solvent in one location rather than the small raised centre of the vial bottom leading to a ring of solvent around the edge. Storage of the solution for 30 minutes at –35 °C afforded a small crop of pale blue planks from which the structure of **4Nd** was gathered. Additional visually identical material was obtained after storage overnight (16 hours) at –35 °C and subjected to a unit-cell check only. A yield is not reported as this procedure was intended only to confirm that crystals suitable for single-crystal X-ray diffraction could be produced.

Purification of $Am^{3+}_{(aq)}$ from aged oxide and stainless steel contamination

A metal vessel, obtained internally from historic LANL inventory, contained ^{243}Am-oxide residue that was adhered to the inner walls of the vessel with no loose. Initial dose measurements suggested that the ^{243}Am content was an appreciable enough quantity to support a multi-milligram molecular synthetic chemistry reaction. The composition of the vessel was not known, likely to be either aluminum or steel but suspected to be steel based upon the strong yellow color that was brought into solution upon leaching with HCl solutions. First of all, the bottom and side walls of the vessel were scraped with a spatula to release a dark colored solid that was transferred to a glass vial. Substantial γ-dose was still measured coming through the vessel walls, so the water was added to the vessel with further scraping to release additional dark solid in the form of a suspension which was transferred to the glass vial with a pipette. The vessel was then leached with 1 M HCl solution,

again with scraping, and then the solution/suspension transferred to the glass vial. The contact time of the 1 M HCl solution with the vessel was minimized to reduce the amount of non-Am metal impurities dissolved into solution. The process was repeated until the dose rate coming from the vessel no longer appeared to be dropping as a result of the leaching. The suspension was heated to boiling, and the volume reduced to ~ 3 mL. The resultant solution was centrigued to remove trace solids and assayed by UV-vis-NIR spectroscopy and estimated to contain approximately 25-35 mg of ^{243}Am content, although this estimation was complication by an intense broad absorption feature in the visible region that was presumably due to one or more of the metal impurities from the vessel material. Initial purification broadly followed the basic steps outlined in the literature for Cm recovery and reprocessing,[14] with a series of Am precipitations consisting of Am^{3+} hydroxide, "$Am(OH)_3$", drop, by adding NH_4OH, an "AmF_3" drop by adding HF (the solid was then resdissolved using boric acid/HCl), then a repeat of the hydroxide drop followed by dissolution of the solid in 0.5 M HCl (for each precipitation, the solids were washed with water before being redissolved). Final purification was by cation exchange chromatography (AG 50-X8 resin, 100-200 mesh, loaded at a height of 17 cm onto a 30 cm x 0.7 cm Kontes Flex column equipped with an 80 mL funnel reservoir), the loading and elution conditions for which were first tested on Nd^{3+} as a surrogate for Am^{3+} in order to maximize Am recovery the elute fraction, but generally based on the literature.[15] The resin was pre-conditioned with 0.5 M HCl, the Am solution loaded onto the column, which was then washed with 5 x 25 mL portions of 2 M HCl. Based on testing with Nd^{3+}, it was estimated that 20 mL of 6 M HCl elute recovered ~92% of the Nd. For Am^{3+}, we decided elute 30 mL of 6 M HCl to result in high recovery without a high volume that was too unwiedly for subsequent manipulations. This ~30 mL elute was estimated to contain ~30 mg ^{243}Am by UV-vis-NIR spectroscopy. The elute was then precisely weighed (30.2677 g), and an aliquot of the elute was subjected to serial dilutions in 2% nitric acid by mass until a concentration of ~0.1 ng/mL was reached (using the approximate value from the UV-vis-NIR as a guide). An aliquot of the ^{243}Am dilution was traced with ^{241}Am (~100 pg of NIST SRM 4322C Am-241 Radioactivity Standard). The combined solutions were evaporated to dryness, redissolved in 2% HNO_3 (v/v), and analyzed using a Thermo X-Series Quadrupole ICP-MS. The samples were analyzed using an enhanced ionization setup consisting of an ESI Apex

desolvating nebulizer, affording an instrumental sensitivity of ~1.5 x 10^6 cps per ppb ^{238}U. A similar analysis with a second untraced aliquot was used to determine the isotopic composition of the americium. For the ICP-MS analysis, mass fractionation was corrected using NBL CRM128 (Plutonium-239/Plutonium-242 Atom Ratio Plutonium Isotopic Standard). The americium standard reference material IRMM-0243 was analyzed in parallel with the samples.[16] Based on the ICP-MS results of 0.131 ng/g of ^{243}Am in the serial diluted solution, the Am concentration of the elute was then back-calcuated as containing 30.6 mg ^{243}Am and this value was used as the basis for aliquoting an Am solution for the synthesis on **4Am** below.

Synthesis of [Am(Cp)$_2$(I$_x$Cl$_{1-x}$)(I^{Me4})] (**4Am**) from $^{243}Am^{3+}$ dissolved in HCl$_{(aq)}$*

An aqueous 6 M $HCl_{(aq)}$ (5.95860 g) solution containing $^{243}Am^{3+}$ (5.03 mg metal content), aliquoted from the above purified solution,) was reduced to dryness under a stream of UHP Ar gas in a 20 mL glass scintillation vial over *ca*. 12 hours then brought into a transuranium He inert-atmosphere glovebox by an overnight (16 hour) vacuum cycle. The pale pink solid was suspended in DME (1.5 mL) with manual loosening of solids from the vial wall – the spatula was washed with DME (0.5 mL) – and then heated (hot plate set to 70 °C) for 15 minutes. The pale pink/white suspension was allowed to cool to room temperature where there was no discernible color to the supernatant. At room temperature, Me_3SiI (*ca.* 1.5 mL) was added with stirring which caused the vial to become cloudy with a fine orange blue solid and a pale yellow supernatant. The mixture was stirred at room temperature for 5 minutes, followed by heating (hot plate set to 60 °C) for 1 hour. The mixture was cooled to room temperature, then pentane (10 mL) was added which caused some precipitation of a pale powder. After stirring at room temperature for 2 minutes, the mixture was stored at –35 °C for 1 hour to settle the solids. The slightly turbid pale yellow supernatant was decanted, then Et_2O (1.5 mL) was added and the suspension was stirred for 2 minutes followed by the addition of more pentane (10 mL). The cloudy mixture was stored at –35 °C for 30 minutes and then pale yellow/colorless supernatant was decanted from the pale orange solids. Pentane (5 mL) was added to the pale orange solids followed by stirring for 2 minutes, all the solids were loosened with a

spatula, and the suspension was stored at –35 °C for 30 minutes. The colorless supernatant was decanted and the pale orange powder was dried *in vacuo* for 1 hour.

THF (1.5 mL) was added with stirring to the pale orange powder prepared above, which afforded a pale yellow solution. Solid KCp* (7.5 mg, 43 μmol, 2.1 equiv.) was added to the stirred mixture which immediately became more cloudy and an orange/pink color developed in the solution. The mixture was stirred for 1 hour and then the volatiles were removed *in vacuo* to afford an orange solid. Toluene (1.5 mL) was added followed by THF (2 drops, 24 mg), and the suspension was stirred for 10 minutes. The golden/peach solution was filtered into a fresh 20 mL glass scintillation vial through 2 half-discs of glass microfiber mounted in a glass pipette, and a Teflon-coated stirrer bar was added. *Note the filter pipette was not rinsed with additional solvent*. The solution was stirred gently and solid I^{Me4} (2.6 mg, 21 μmol, 1.05 equiv.) was added which did not lead to any noticeable color change. The stir-rate was increased, and over the course of 10 minutes no visible solids precipitated. The golden solution was filtered into a 4 mL glass vial through 2 half-discs of glass microfiber mounted in a glass pipette and then concentrated to *ca.* 0.3 mL then stored at –35 °C overnight (12 hours) whereupon a small amount of colorless microcrystalline material formed. The solution was filtered into a fresh 4 mL glass vial through 2 half-discs of glass microfiber mounted in a glass pipette and then concentrated to *ca.* 0.1 mL – the volume was small enough that the vial had to be stored at an angle so as to have all the solvent in one location rather than the small raised centre of the vial bottom leading to a ring of solvent around the edge. Storage of the solution overnight (16 hours) at –35 °C afforded a crop of golden planks from which the structure of **4Am** was gathered. Additional visually identical material was obtained by layering the supernatant with hexane (0.5 mL) followed by storage overnight (16 hours) at –35 °C. A percentage yield is not given due to the mixed halide occupancy, though we obtained 4.2 mg over the two crystalline crops.

NMR and UV-vis-NIR spectroscopies were all performed from the same first crop of crystals from which the three X-ray diffraction data sets were obtained. Assignment of the two species in the ^{1}H NMR spectrum was performed by comparison of integral values with the Cl / I ratio obtained from

X-ray diffraction data – see **Figure S64** and **Figure S65**. We have not attempted to perform assignment of the Cl / I species in the $^{13}C\{^{1}H\}$ NMR spectrum – see **Figure S67**.

4Am-I ^{1}H NMR (D_6-benzene, 400.13 MHz, 298 K): δ = 1.13 (s, 30 H, Cp* $C_5(C\underline{H}_3)_5$), 1.91 (s, 3 H, I^{Me4} $C(C\underline{H}_3)$), 2.23 (s, 3 H, I^{Me4} $C(C\underline{H}_3)$), 6.41 (s, 3 H, I^{Me4} $N(C\underline{H}_3)$), 6.60 (s, 3 H, I^{Me4} $N(C\underline{H}_3)$).

4Am-Cl ^{1}H NMR (D_6-benzene, 400.13 MHz, 298 K): δ = 1.59 (s, 30 H, Cp* $C_5(C\underline{H}_3)_5$), 2.12 (s, 3 H, I^{Me4} $C(C\underline{H}_3)$), 2.39 (s, 3 H, I^{Me4} $C(C\underline{H}_3)$), 6.14 (s, 3 H, I^{Me4} $N(C\underline{H}_3)$), 6.39 (s, 3 H, I^{Me4} $N(C\underline{H}_3)$).

$^{13}C\{^{1}H\}$ NMR (D_6-benzene, 100.62 MHz, 298 K): δ = 7.35 (I^{Me4} $C(\underline{C}H_3)$), 7.44 (I^{Me4} $C(\underline{C}H_3)$), 7.45 (I^{Me4} $C(\underline{C}H_3)$), 7.57 (I^{Me4} $C(\underline{C}H_3)$), 29.83 (Cp* $C_5(\underline{C}H_3)_5$), 31.12 (Cp* $C_5(\underline{C}H_3)_5$), 46.68 (I^{Me4} $N(\underline{C}H_3)$), 50.45 (I^{Me4} $N(\underline{C}H_3)$), 56.91 (I^{Me4} $N(\underline{C}H_3)$), 56.98 (I^{Me4} $N(\underline{C}H_3)$), 100.34 (Cp* $\underline{C}_5(CH_3)_5$), 100.39 (Cp* $\underline{C}_5(CH_3)_5$), 113.84 (I^{Me4} $C(\underline{C}H_3)$), 114.31 (I^{Me4} $C(\underline{C}H_3)$), 114.81 (I^{Me4} $C(\underline{C}H_3)$), 115.15 (I^{Me4} $C(\underline{C}H_3)$) ppm – we could not observe the carbene resonance.

ε values are given for UV-vis-NIR data as a guide, and there will be significant errors due to not only the small quantity of material but also the uncertainty in the Cl / I ratio.

UV-vis-NIR (toluene): λ_{max} (cm^{-1}; ε) = 311 (32,154, 1,600), 346 (28,902, 1,370), 357 (28,011, 1,350), 377 (26,525, 619), 379 (26,385, 601), 380 (26,316, 601), 387 (25,840, 539), 394 (25,381, 452), 408 (24,510, 244), 415 (24,096, 158), 430 (23,256, 70), 435 (22,989, 54), 447 (22,371, 42), 451 (22,173, 47), 458 (21,834, 25), 461 (21,692, 32), 463 (21,598, 45), 467 (21,413, 39), 469 (21,322, 78), 476 (21,008, 92), 478 (20,921, 101), 522 (19,157, 108), 526 (19,011, 393), 529 (18,904, 339), 531 (18,832, 422), 797 (12,547, 31), 828 (12,077, 270), 851 (11,751, 264), 888 (11,261, 5), 892 (11,211, 7), 907 (11,025, 9), 916 (10,917, 6), 1,015 (9,852, 28), 1,037 (9,643, 6), 1,058 (9,452, 2), 1,104 (9,058, 29), 1,109 (9,017, 28), 1,121 (8,921, 5), 1,142 (8,757, 15), 1,148 (8,711, 12), 1,191 (8,396, 4), 1,305 (7,663, 2).

Synthesis of [Nd(Cp)$_2$(Cl)(I^{Me4})] (**5Nd**) from NdCl$_3$*

In order to more thoroughly investigate the differences between Nd, and Am, within this molecular framework, we have independently synthesized and characterized both the chloride and iodide forms of [Nd(Cp*)$_2$(X)(I^{Me4})] (X = Cl, I) as our initial single-crystal X-ray diffraction study used a crystal with mixed Cl / I occupancy (**4Nd**).

Synthesis of [Nd(Cp*)$_2$(Cl)(I^{Me4})] (5Nd). THF (1.5 mL) was added to solid NdCl$_3$ (40 mg, 160 μmol) in a 20 mL glass scintillation vial with a Teflon-coated stirrer bar, followed by solid KCp* (60 mg, 344 μmol, 2.15 equiv.). The colorless suspension rapidly turned pale blue and was heated/stirred to a very gentle boil in the sealed vial for 45 minutes and then allowed to cool to room temperature which afforded a pale blue turbid solution. The volatiles were removed *in vacuo*, then the blue solids were suspended in toluene (1 mL). Prolonged gentle reflux (2-3 minutes) did not cause any of the blue solids to dissolve, and the supernatant remained colorless. THF (total 1 mL) was added portionwise with modest heating (hot plate set to 50 °C) and stirring until the solids were no longer visibly blue, and then cooled to room temperature. The mixture was centrifuged (5,000 rpm, 10 minutes) which gave a pale blue solution with a small amount of grey/white solid. The solution was filtered into a 20 mL glass scintillation vial through 2 half-discs of glass microfiber mounted in a glass pipette. A Teflon-coated stirrer bar was added, followed by solid I^{Me4} (22 mg, 177 μmol, 1.11 equiv.). The pale blue solution was gently stirred for 10 minutes which gave a significant quantity of microcrystalline material on the vial walls. The vial was heated to reflux, allowed to cool just enough so that it could be filtered easily, and the supernatent was filtered into a 4 mL glass vial through 2 half-discs of glass microfiber mounted in a glass pipette. Storage at room temperature overnight (16 hours) afforded a small crop of pale blue planks, followed by a much larger second crop when the supernatant was stored at –35 °C overnight (16 hours). Both sets of crystals were washed with hexane (2 × 1 mL) and dried *in vacuo* for 1 hour (combined yield = 21.8 mg, 24%). Elemental analysis calc. (%): C = 56.46, H = 7.37, N = 4.88; found (%): C = 55.52, H = 7.56, N = 4.92.

^{1}H NMR (D$_6$-benzene, 400.13 MHz, 298 K): δ = −28.46 (s, 3 H, I^{Me4} C(C$\underline{H}_3$)), −24.39 (s, 3 H, I^{Me4} C(C$\underline{H}_3$)), −5.51 (s, 3 H, I^{Me4} N(C$\underline{H}_3$)), −5.15 (s, 3 H, I^{Me4} N(C$\underline{H}_3$)), 9.95 (s, 30 H, Cp* C$_5$(C$\underline{H}_3$)$_5$).

Magnetic moment (Evans method, D_6-benzene, 298 K): μ_{eff} = 3.68 μB.

UV-vis-NIR (THF): 367 (27,248, 209), 437 (22,883, 3), 439 (22,779, 4), 483 (20,704, 4), 515 (19,417, 5), 519 (19,268, 5), 522 (19,157, 7), 525 (19,048, 7), 527 (18,975, 5), 530 (18,868, 13), 534 (18,727, 7), 536 (18,657, 9), 538 (18,587, 6), 540 (18,519, 8), 543 (18,416, 6), 545 (18,349, 6), 548 (18,248, 5), 583 (17,153, 10), 587 (17,036, 28), 591 (16,920, 37), 593 (16,863, 66), 597 (16,750, 246), 597 (16,750, 225), 599 (16,694, 151), 601 (16,639, 69), 603 (16,584, 141), 607 (16,474, 28), 609 (16,420, 17), 612 (16,340, 13), 614 (16,287, 17), 616 (16,234, 14), 618 (16,181, 9), 677 (14,771, 3), 683 (14,641, 4), 739 (13,532, 3), 743 (13,459, 6), 745 (13,423, 8), 748 (13,369, 11), 750 (13,333, 11), 752 (13,298, 21), 760 (13,158, 8), 766 (13,055, 9), 777 (12,870, 7), 803 (12,453, 18), 806 (12,407, 7), 806 (12,407, 7), 814 (12,285, 6), 820 (12,195, 7), 823 (12,151, 6), 831 (12,034, 5), 875 (11,429, 2), 885 (11,299, 4), 893 (11,198, 2), 898 (11,136, 2), 903 (11,074, 2), 917 (10,905, 2).

FT-IR (ATR, microcrystalline): cm^{-1} = 368 (vw), 374 (w), 379 (w), 421 (vw), 544 (vw), 552 (vw), 567 (vw), 588 (vw), 600 (vw), 613 (vw), 627 (vw), 652 (vw), 692 (vw), 722 (w), 797 (vw), 802 (vw), 812 (vw), 838 (w), 906 (vw), 942 (vw), 948 (vw), 1,018 (w), 1,058 (vw), 1,078 (vw), 1,094 (vw), 1,108 (vw), 1,155 (vw), 1,218 (vw), 1,291 (w), 1,362 (s), 1,395 (w), 1,431 (m), 1,471 (w), 1,486 (w), 1,491 (w), 1,553 (vw), 1,639 (m), 1,664 (vw), 1,670 (vw), 2,713 (w), 2,750 (w), 2,791 (m), 2,843 (vs), 2,889 (vs), 2,943 (m), 2,962 (m).

S2. Photographs taken during synthesis

Lanthanide syntheses

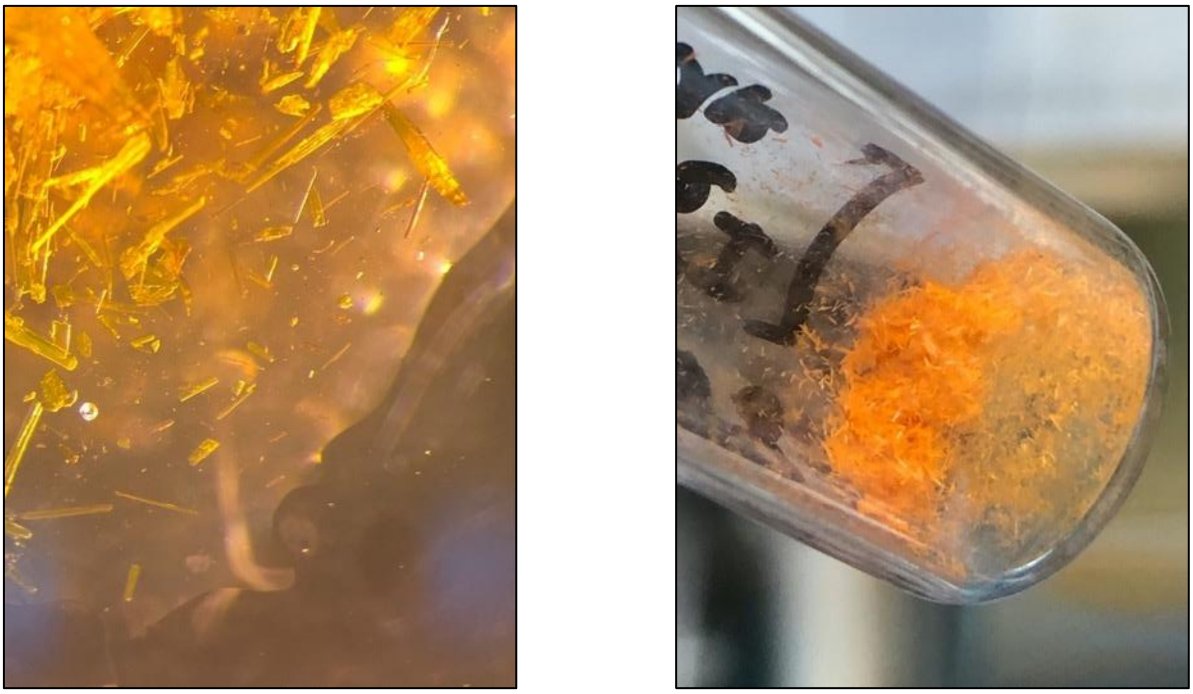

Figure S5. From left to right: 1) Crystals of [Ce(Cp*)$_2$(I)(THF)] (**1Ce**) under NVH oil, that formed upon slow concentration *in vacuo* of a toluene solution; 2) Crystals of **1Ce** after washing and drying *in vacuo* (10^{-2} mbar).

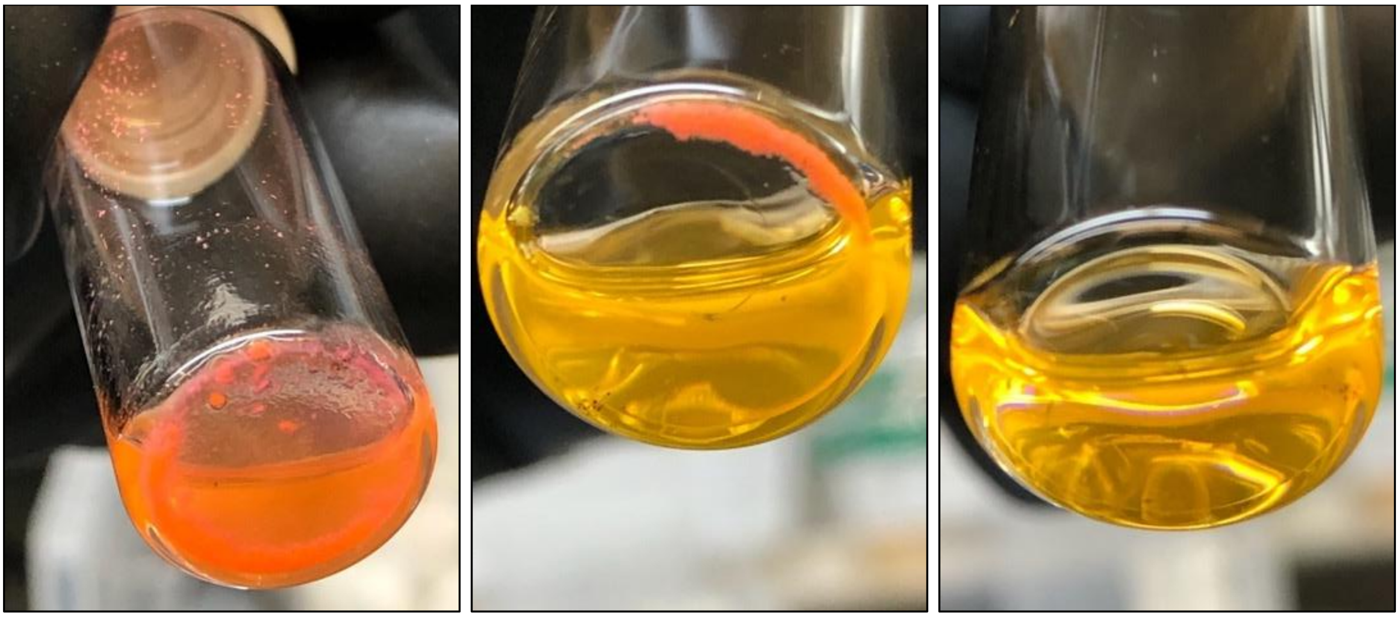

Figure S6. From left to right: 1) Pink solids, putative "Ce(Cp*)$_2$(I)" (**3**), precipitated from toluene; 2) the same material as (1) after addition of 1 drop (12 mg) THF; 3) the same material as (2) after several minutes of manual agitation.

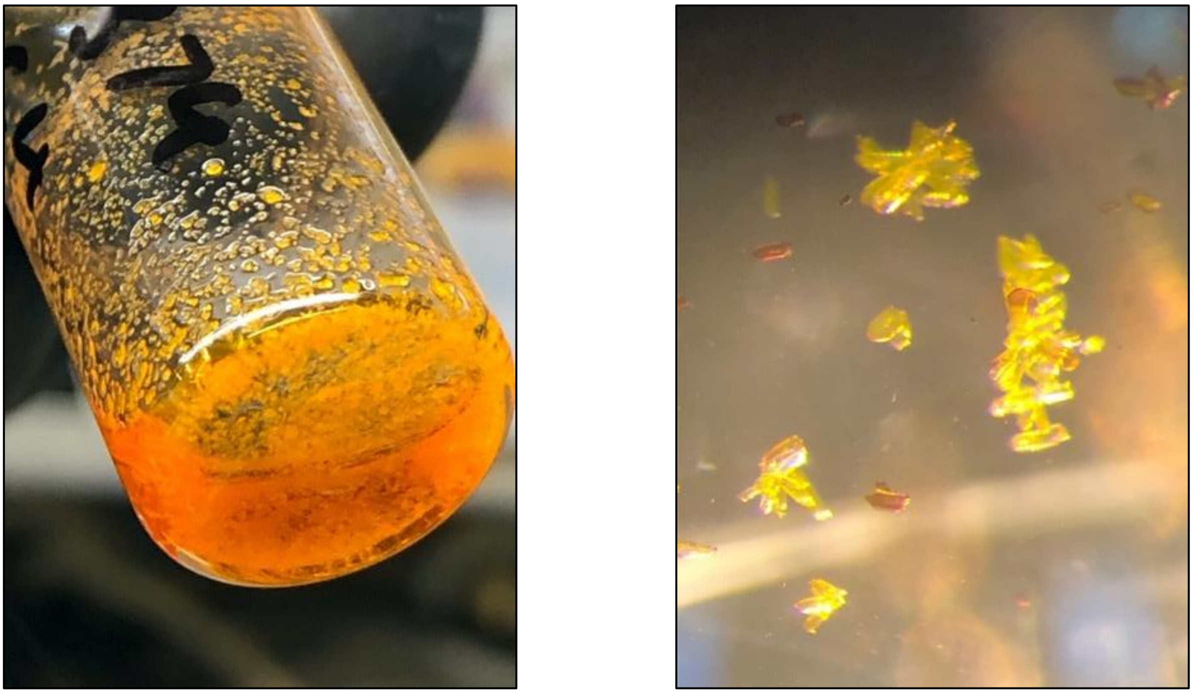

Figure S7. From left to right: 1) Crystals of $[Ce(Cp^*)_2(I)(I^{Me4})]$ (**2Ce**) that formed *in vacuo* during concentration of the reaction supernatent; 2) Crystals of **2Ce** under NVH oil. Note the morphology differs from that of $[Ce(Cp^*)_2(I)(THF)]$ (**1Ce**) shown in **Figure S5-L**. These crystals darkened over time due to air exposure, several darker ones can be seen.

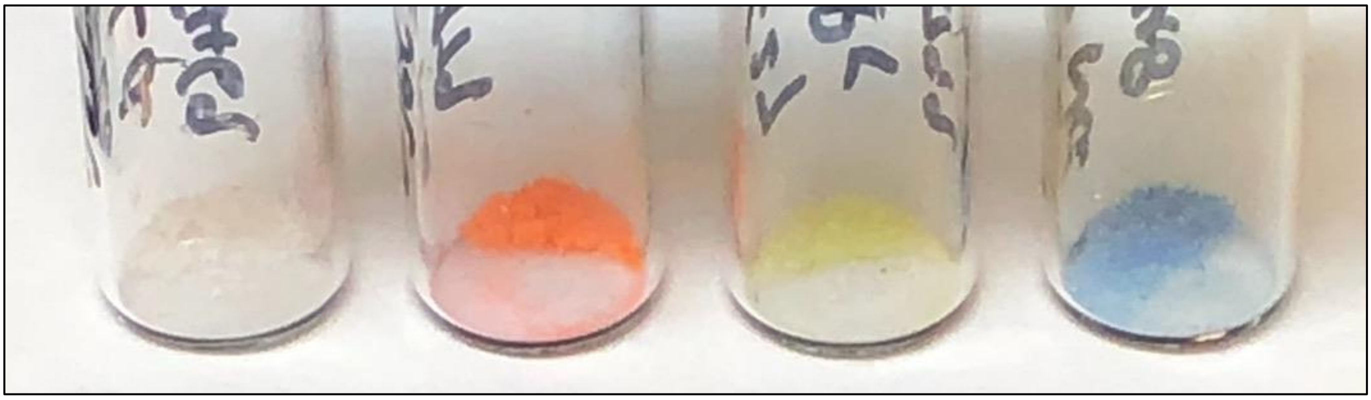

Figure S8. From left to right – crystals of $[Ln(Cp^*)_2(I)(I^{Me4})]$; Ln = La (**2La**), Ce (**2Ce**), Pr (**2Pr**), Nd (**2Nd**).

Neptunium and plutonium syntheses

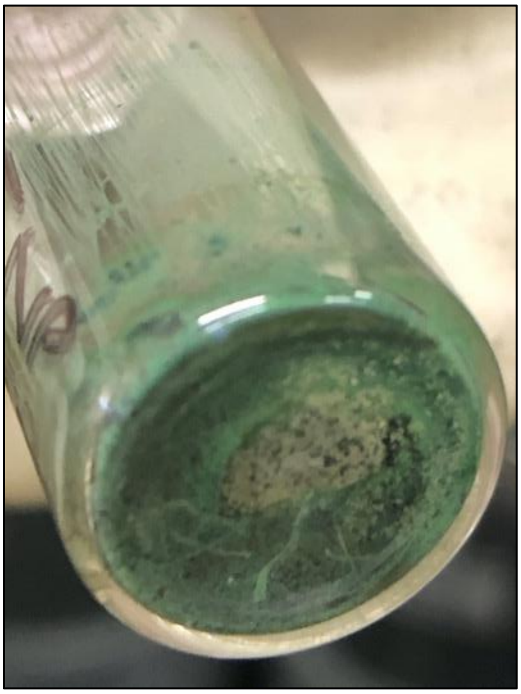

Figure S9. The emerald green powder which is obtained upon stirring crystals of [Np(Cp*)$_2$(I)(THF)] (**1Np**) in hexane for several minutes followed by decantation of the supernatant and drying of the solid. By analogy to observations with Ce (**Figure S6**), it is possibly a Lewis-base free complex "Np(Cp*)$_2$(I)" as it redissolves in THF to give a clear blue/black solution.

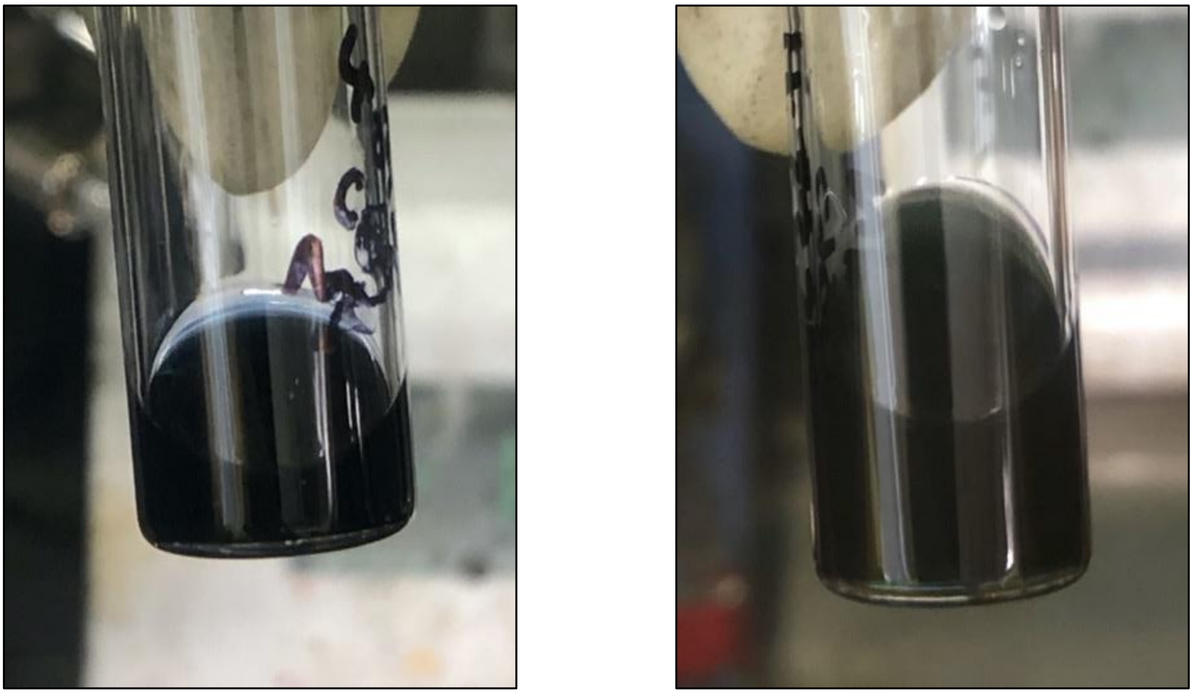

Figure S10. From left to right: 1) A solution of [Np(Cp*)$_2$(I)(THF)] (**1Np**) in toluene; 2) The same material several minutes after treatment with a toluene solution of I^{Me4}.

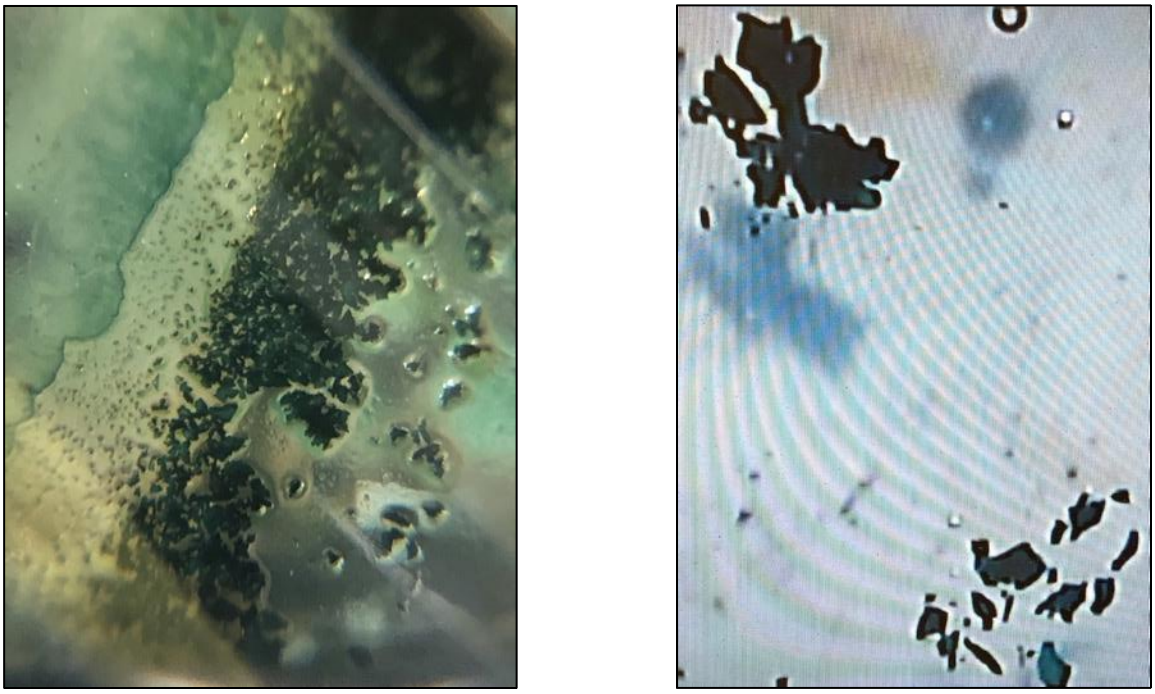

Figure S11. Crystals of [Np(Cp*)$_2$(I)(I^{Me4})] (**2Np**) that formed *in vacuo* during concentration of the reaction supernatent. These were used for a single-crystal X-ray diffraction study.

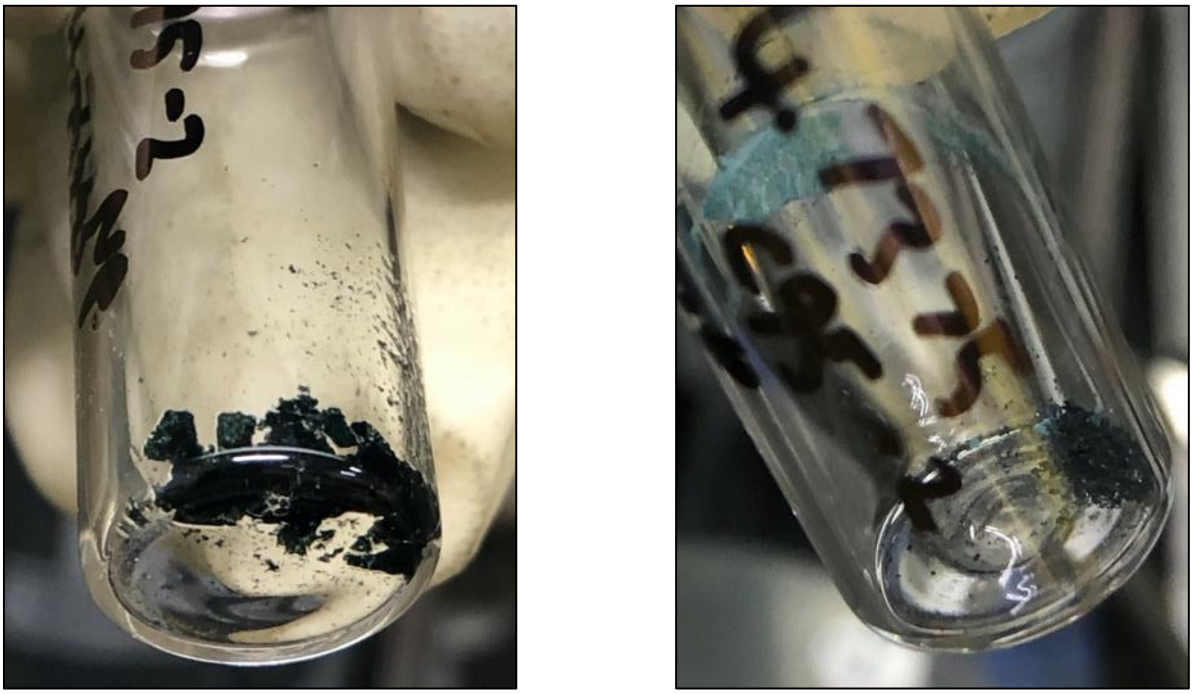

Figure S12. Both dried crystalline crops of [Np(Cp*)$_2$(I)(I^{Me4})] (**2Np**).

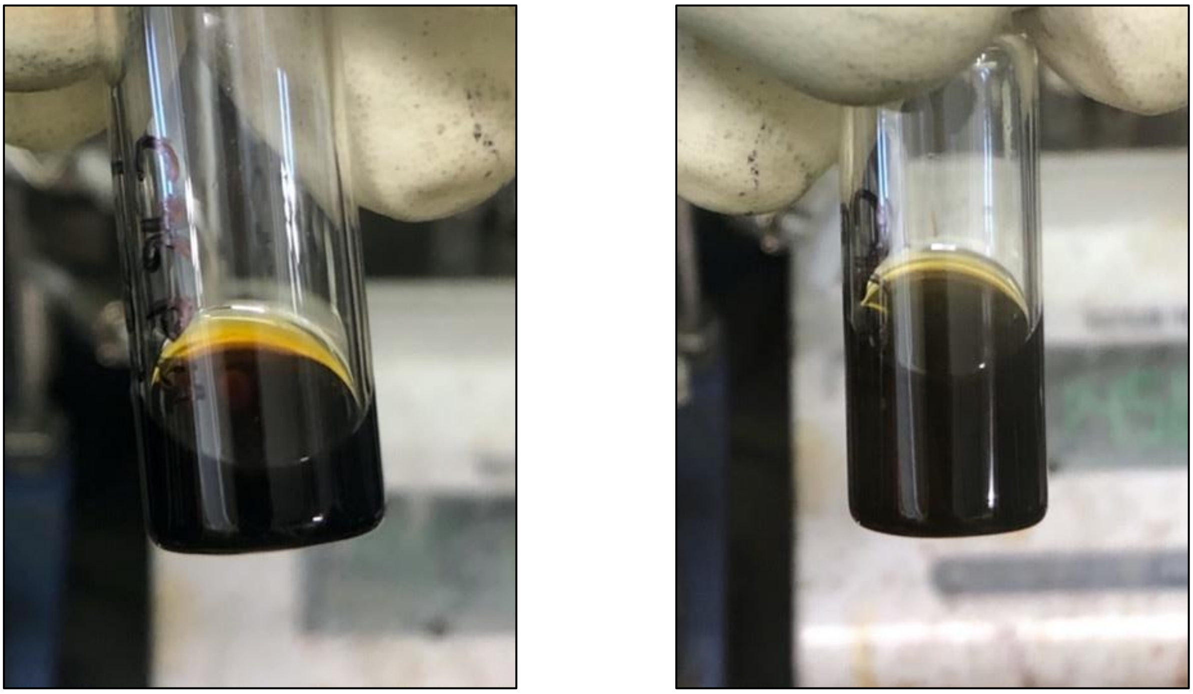

Figure S13. From left to right: 1) A solution of [Pu(Cp*)$_2$(I)(THF)] (**1Pu**) in toluene; 2) The same material several minutes after treatment with a toluene solution of I^{Me4}.

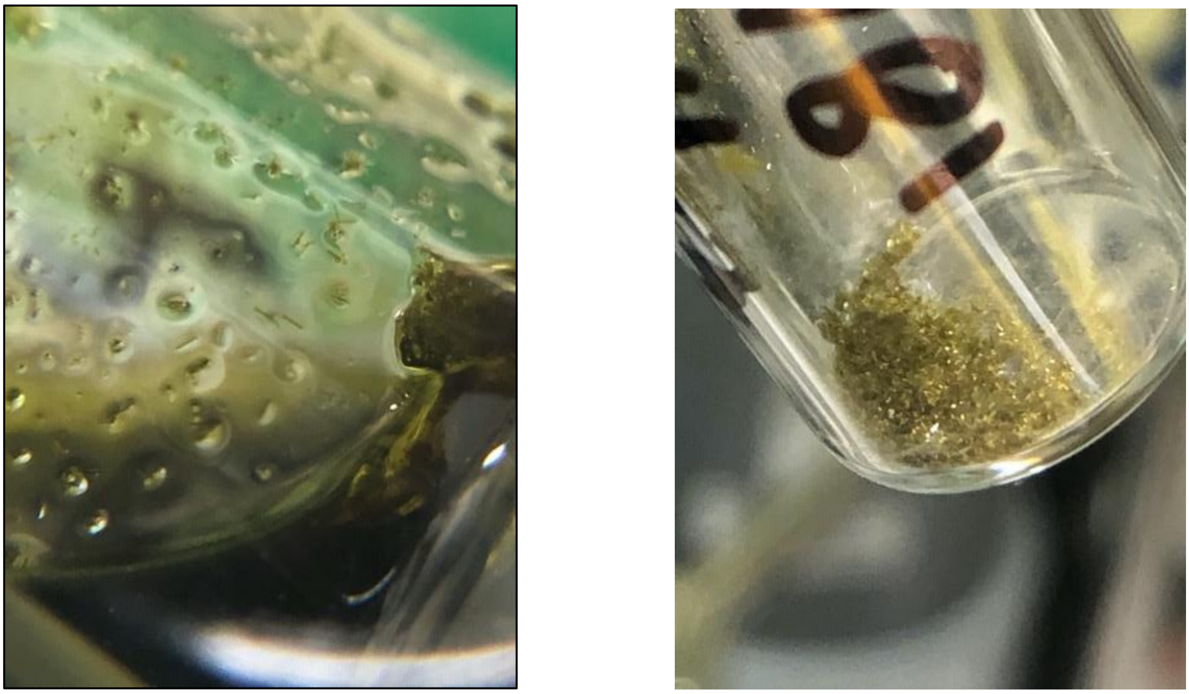

Figure S14. From left to right: 1) Crystals of [Pu(Cp*)$_2$(I)(I^{Me4})] (**2Pu**) that formed *in vacuo* during concentration of the reaction supernatent. These were used for a single-crystal X-ray diffraction study; 2) The combined washed and dried crops of **2Pu**.

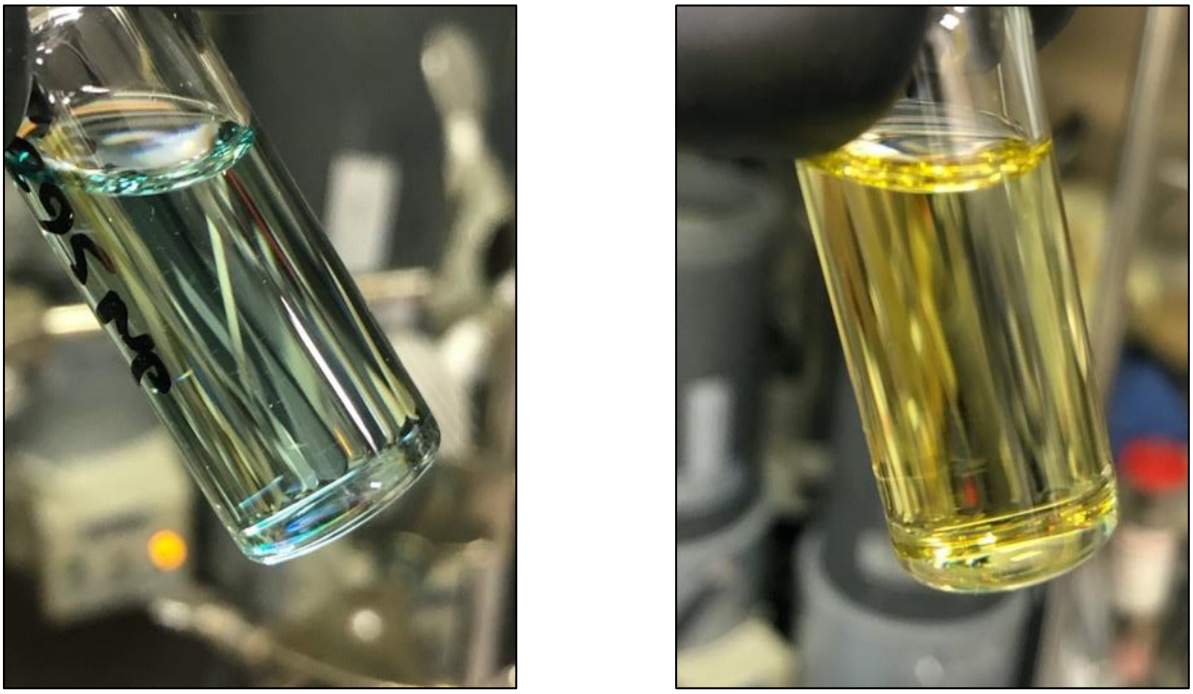

Figure S15. From left to right: 1) A 0.5 mM solution of [Np(Cp*)$_2$(I)(I^{Me4})] (**2Np**) in toluene; 2) A 0.5 mM solution of [Pu(Cp*)$_2$(I)(I^{Me4})] (**2Pu**) in toluene.

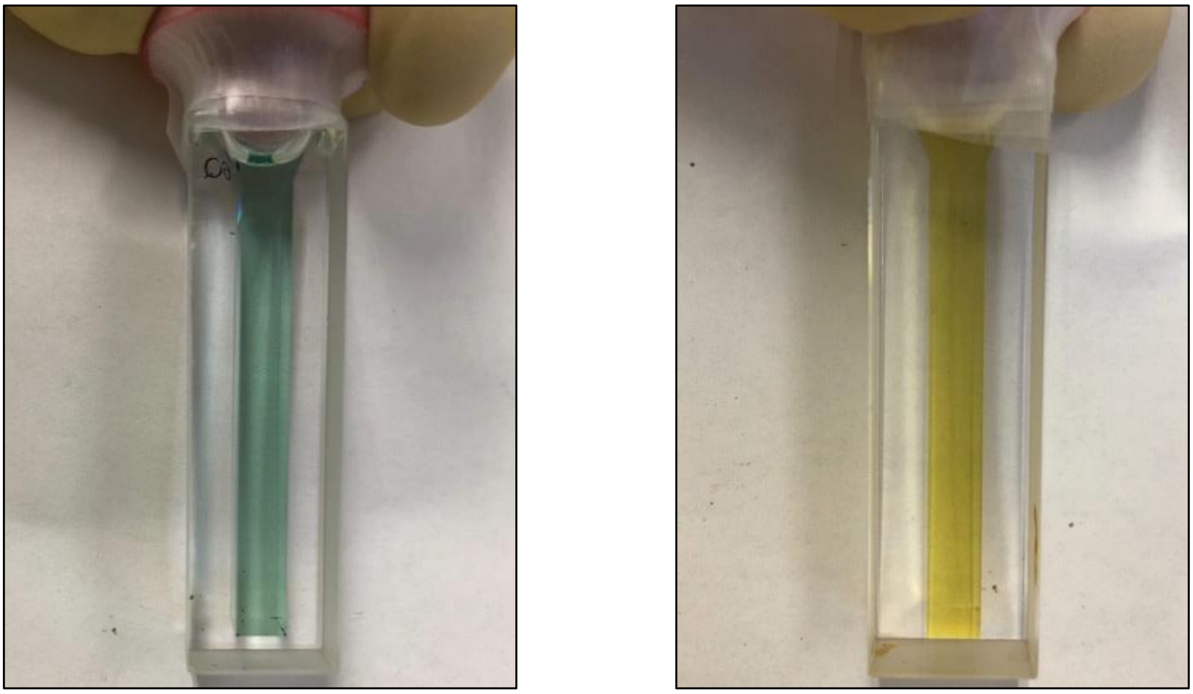

Figure S16. From left to right, the same solutions as **Figure S15** but in 1 cm path length quartz UV-vis-NIR cuvettes: 1) A 0.5 mM solution of [Np(Cp*)$_2$(I)(I^{Me4})] (**2Np**) in toluene; 2) A 0.5 mM solution of [Pu(Cp*)$_2$(I)(I^{Me4})] (**2Pu**) in toluene.

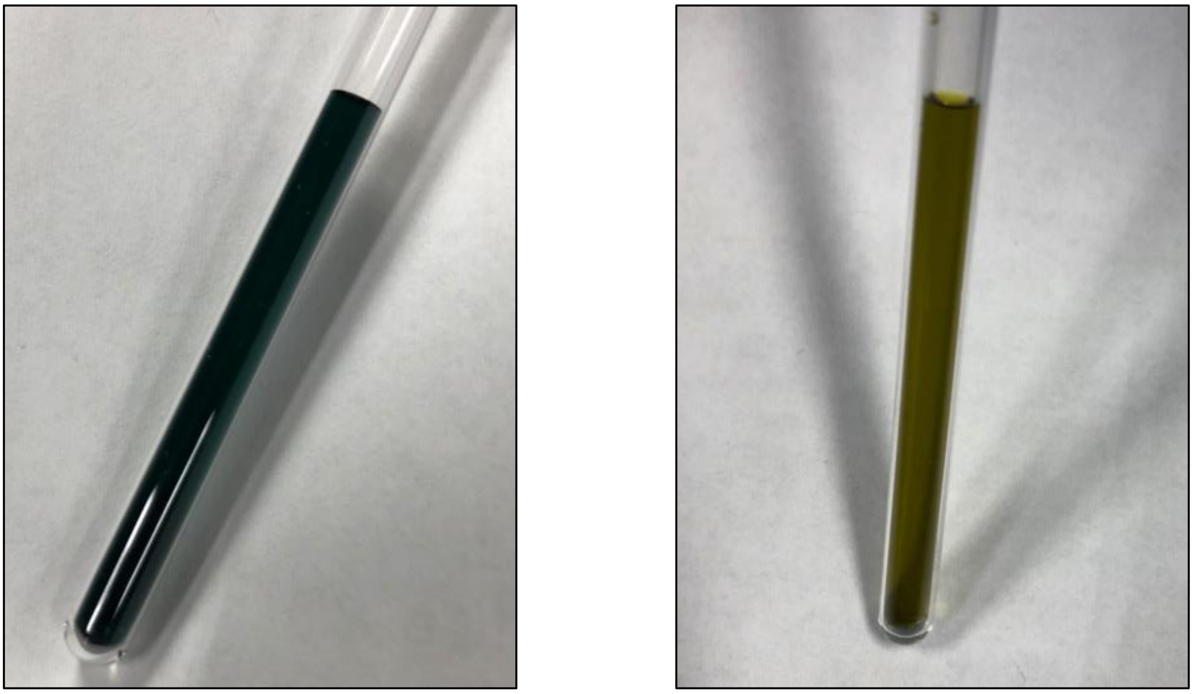

Figure S17. From left to right: 1) A solution of [Np(Cp*)$_2$(I)(I^{Me4})] (**2Np**) in D_6-benzene contained inside an FEP NMR tube liner, which is inside a J. Young tap appended NMR tube; 2) A solution of [Pu(Cp*)$_2$(I)(I^{Me4})] (**2Pu**) in D_6-benzene contained inside an FEP NMR tube liner, which is inside a J. Young tap appended NMR tube.

*Synthesis of **4Am***

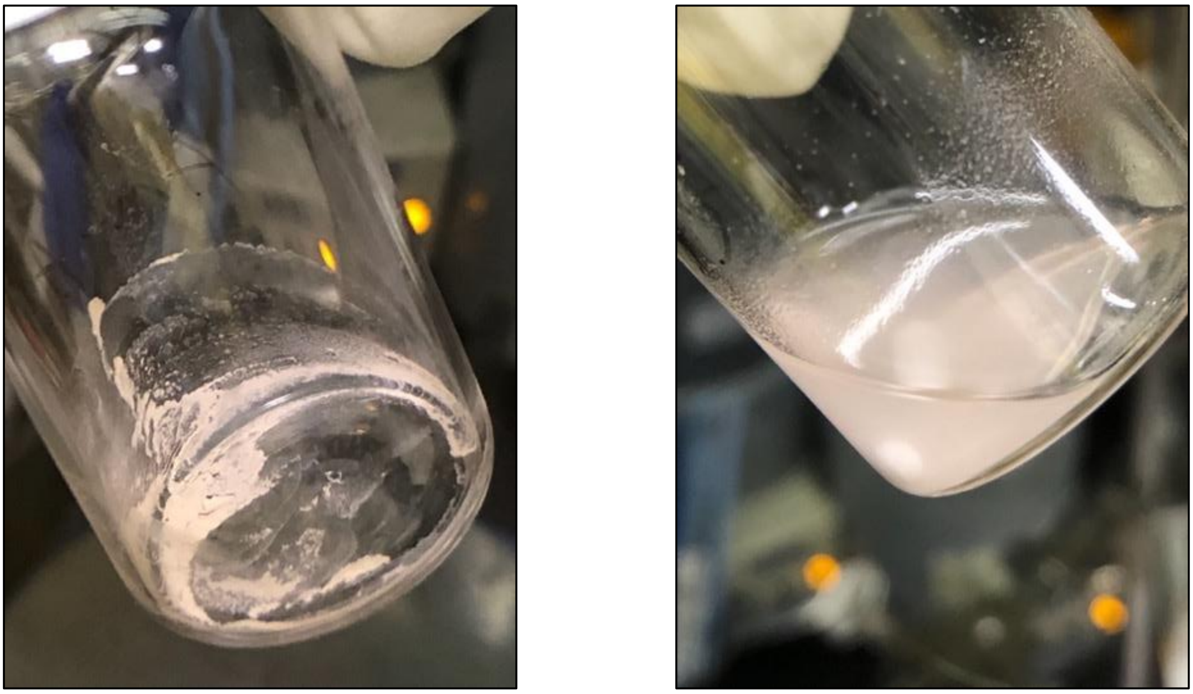

Figure S18. From left to right: 1) $^{243}Am^{3+}$ residue (5.0 mg metal content) after overnight (16 hours) *in vacuo* in the glovebox antechamber; 2) The same material after stirring and heating (70 °C) for 15 minutes in DME.

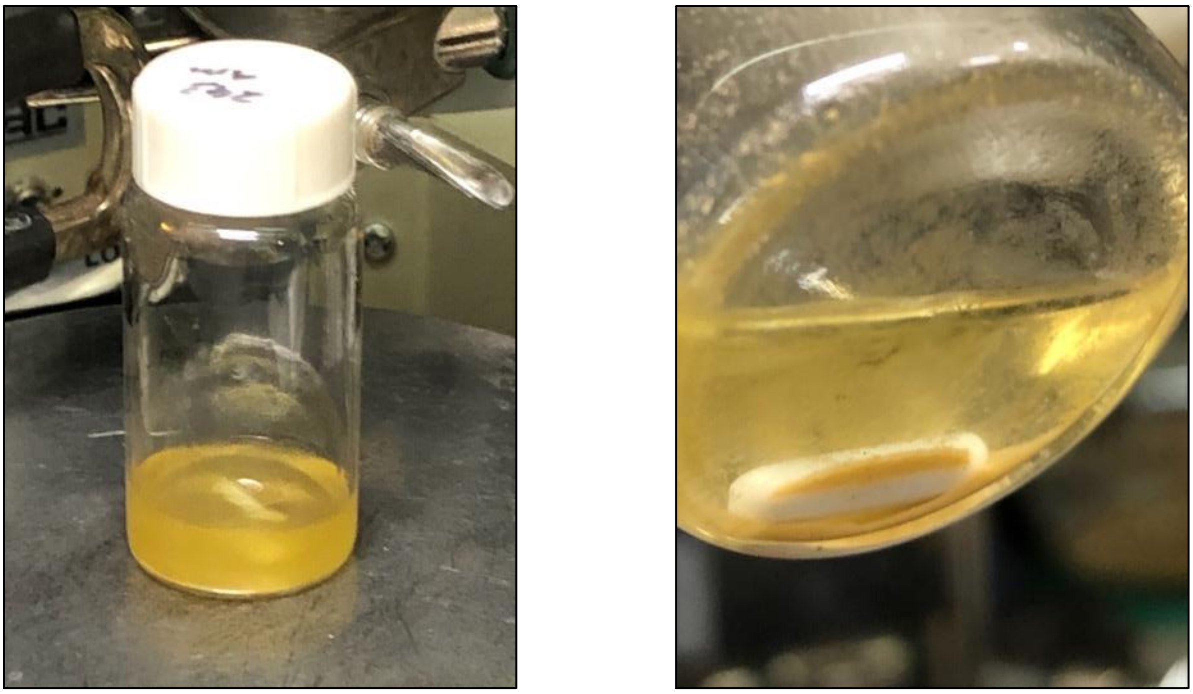

Figure S19. From left to right: 1) The same material as **Figure S18** immediately after the addition of Me_3SiI; 2) Putative "$AmX_3(DME)_n$" (X = I and Cl) mixture after stirring and heating (60 °C) for 1 hour.

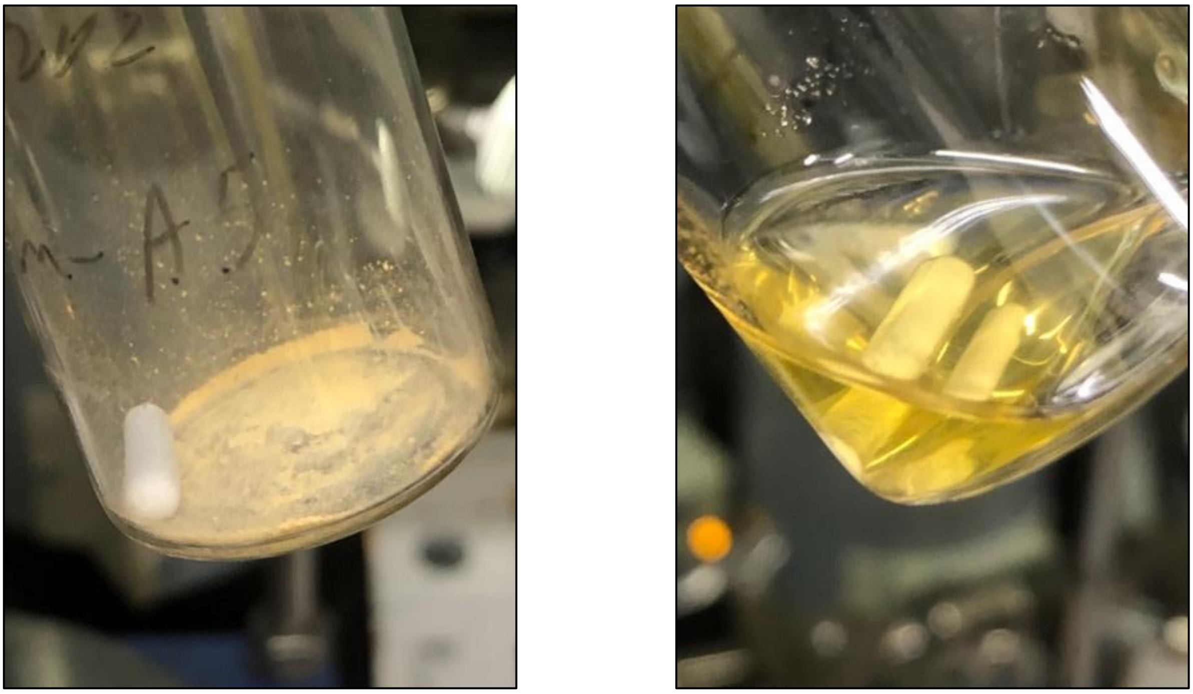

Figure S20. From left to right: 1) Putative "$AmX_3(DME)_n$" (X = I and Cl) solid after the washing procedure and after drying *in vacuo*; 2) The same material after addition of THF (1.5 mL) – note the nearly clear solution which contrasts prior observations when "$AmCl_3(DME)_n$" has been used,[17, 18] which suggested at the time that Cl / I exchange was at least partially successful.

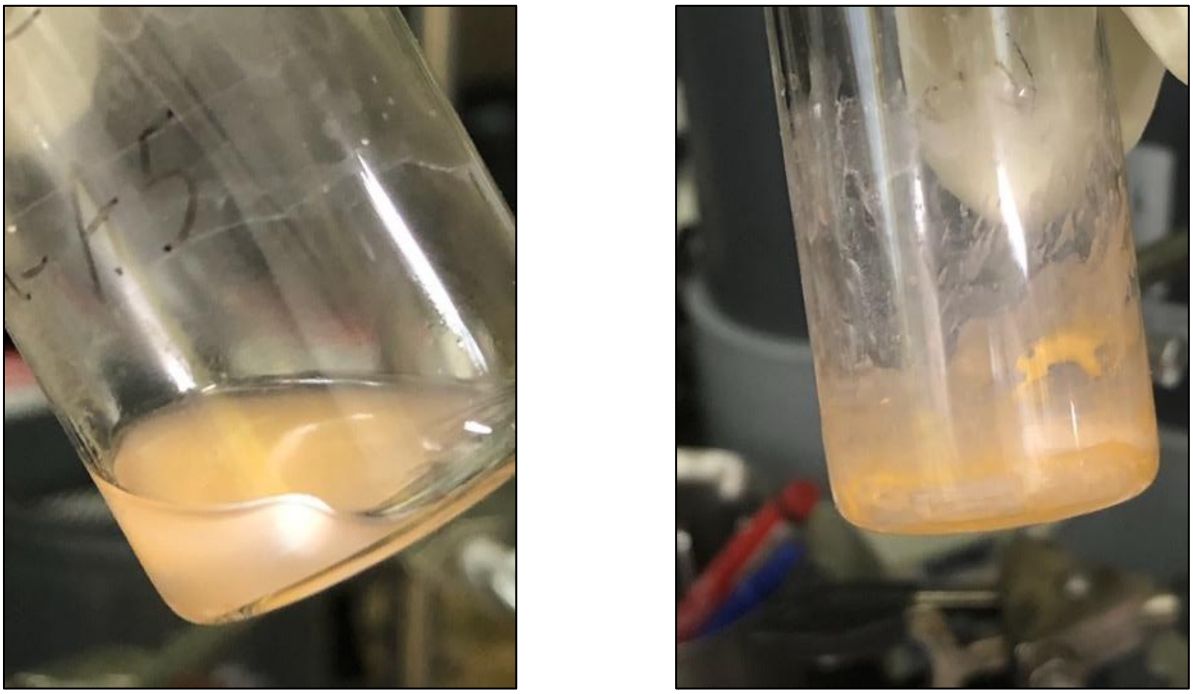

Figure S21. From left to right: 1) THF solution of "$AmX_3(DME)_n$" (X = I and Cl) immediately after the addition of KCp*; 2) Reaction mixture after stirring for 1 hour, then dried *in vacuo*.

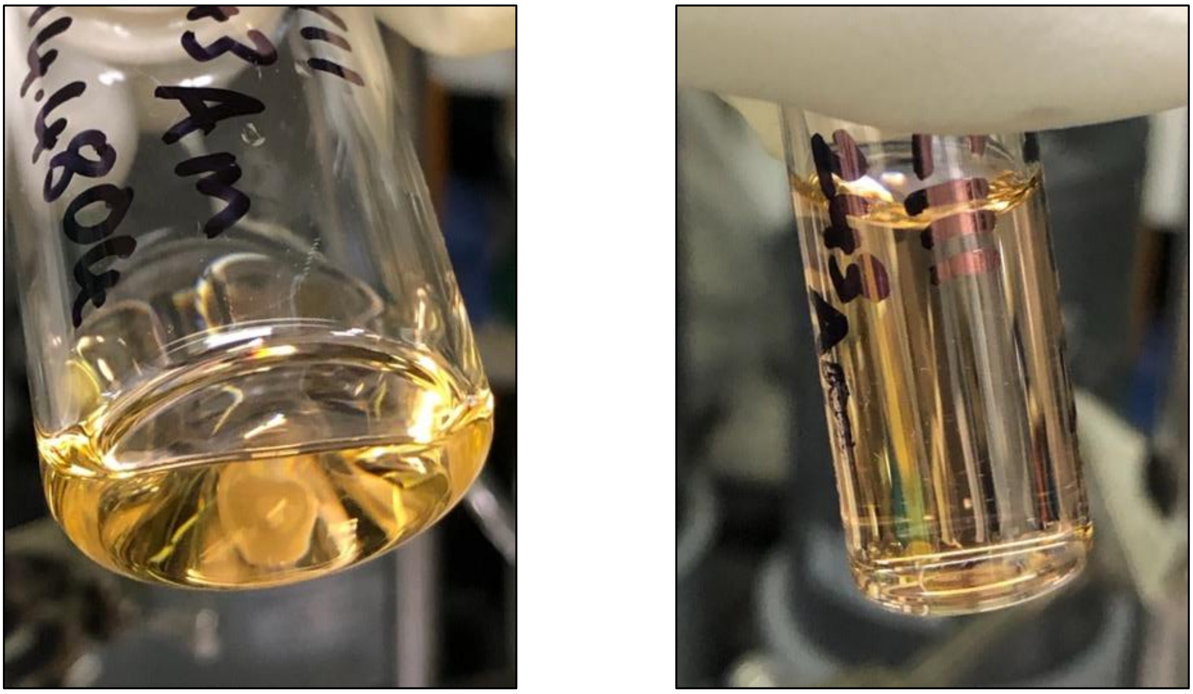

Figure S22. From left to right: 1) Solution of putative “Am(Cp*)$_2$(X)” (X = I and Cl) after the solid in **Figure S21** was extracted with toluene (1.5 mL) / THF (2 drops, 24 mg). A Teflon-coated stirrer bar was added, followed by solid I^{Me4} which caused no discernible color change or precipitation of solids; 2) Solution of putative [Am(Cp*)$_2$(Cl$_x$I$_{1-x}$)(I^{Me4})] (**4Am**) in a 4 mL glass vial after filtration (no discernible solids precipitated, filtration was a precaution to remove any fine solids).

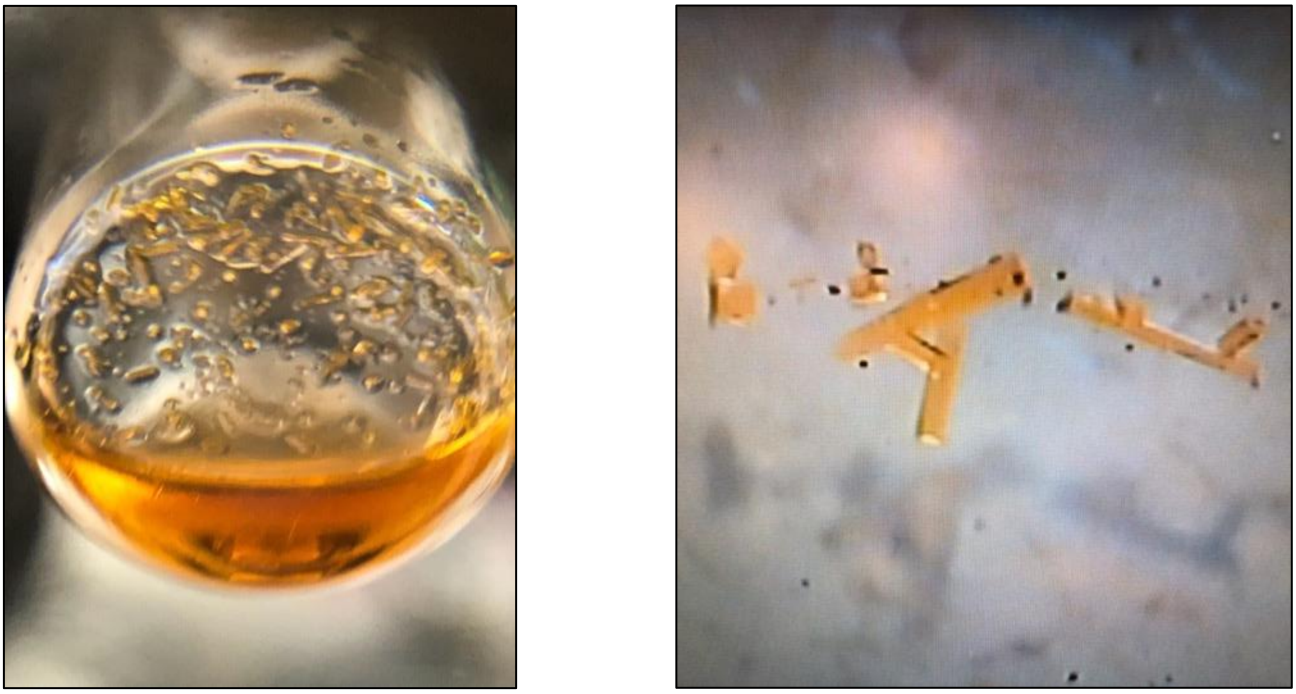

Figure S23. From left to right: 1) Crystals of [Am(Cp*)$_2$(I$_x$Cl$_{1-x}$)(I^{Me4})] (**4Am**) under the reaction supernatent; 2) Several crystals of [Am(Cp*)$_2$(I$_x$Cl$_{1-x}$)(I^{Me4})] (**4Am**) under NVH oil.

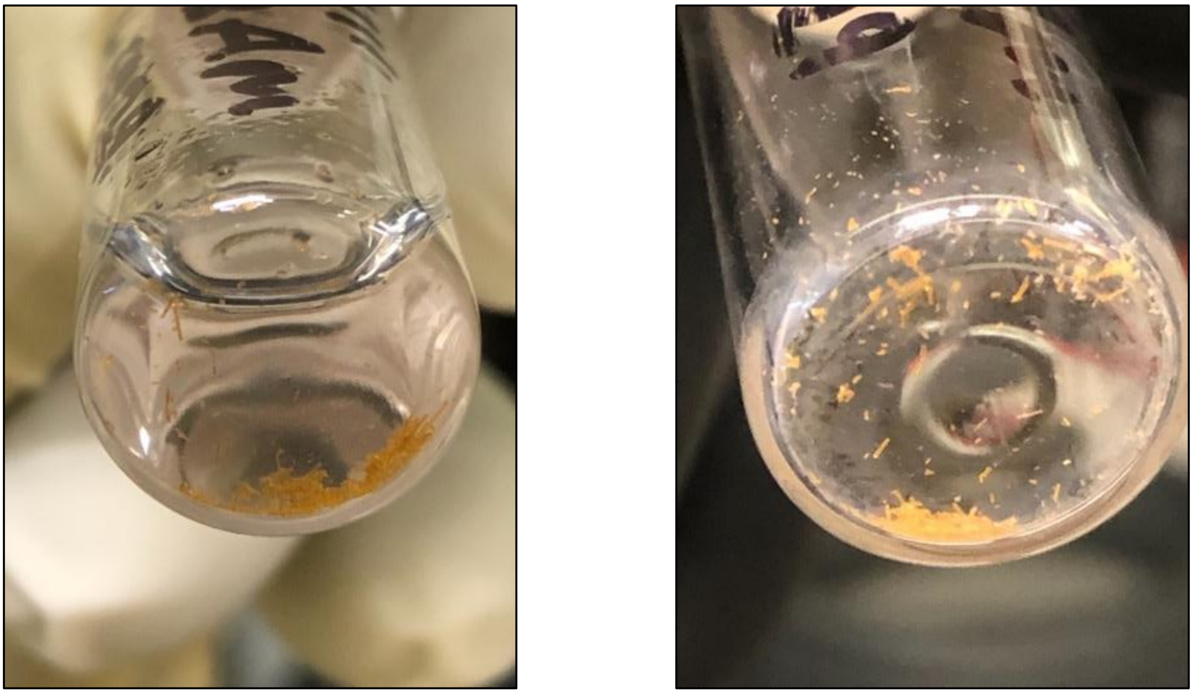

Figure S24. From left to right: 1) The first crop of crystals under hexane during the washing procedure; 2) The first crop of crystals after drying *in vacuo*.

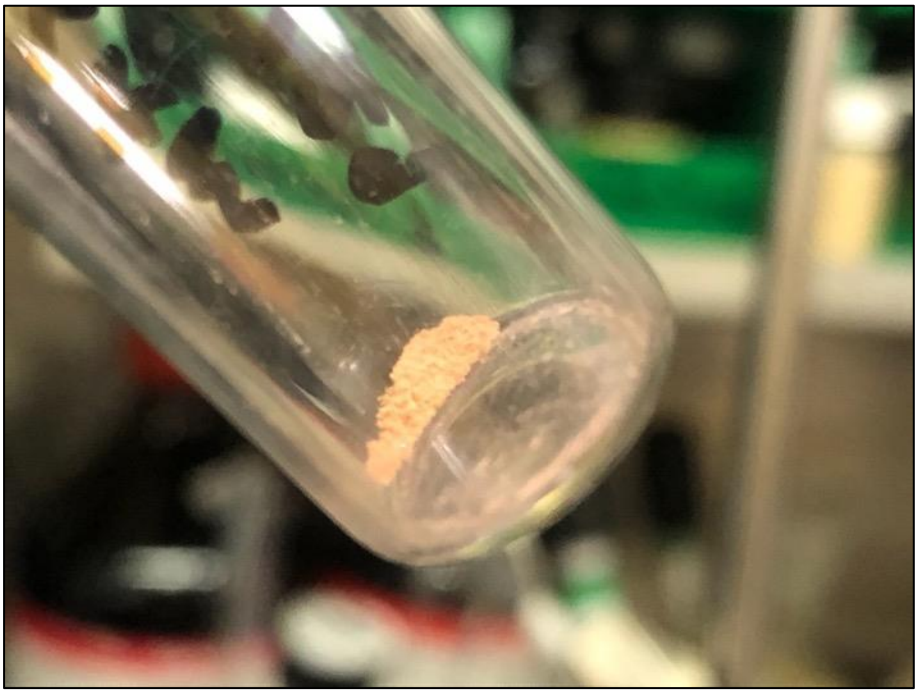

Figure S25. The second crop of crystals after drying *in vacuo*. The crystals were free-flowing.

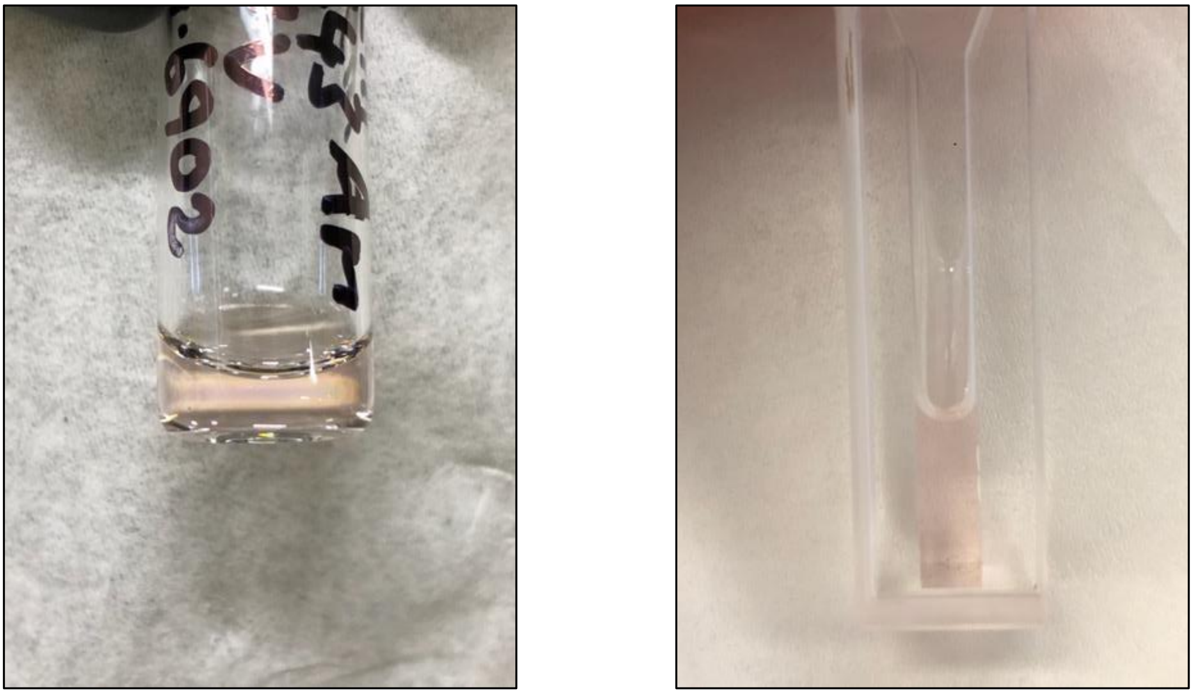

Figure S26. Solution of putative $[Am(Cp^*)_2(I_xCl_{1-x})(I^{Me4})]$ (**4Am**), prepared from the first crop of crystals, in toluene used for UV-vis-NIR spectroscopy.

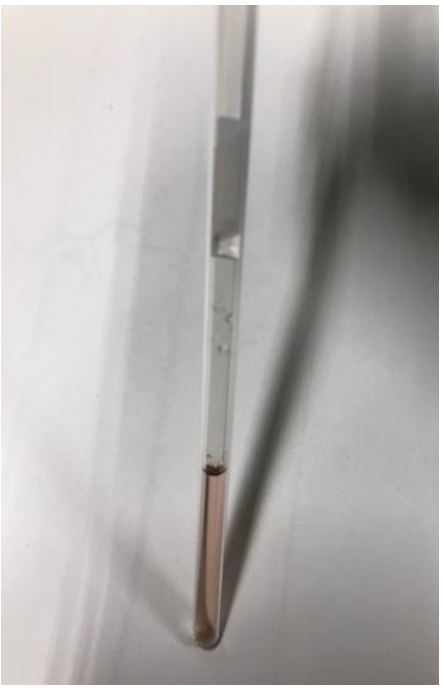

Figure S27. Solution of putative $[Am(Cp^*)_2(I_xCl_{1-x})(I^{Me4})]$ (**4Am**), prepared from the first crop of crystals, in D_6-benzene used for NMR spectroscopy.

S3. Crystallography

General considerations

All crystals were examined with a Bruker D8 Quest diffractometer equipped with a CMOS detector and using mirror-monochromated Mo Kα radiation (λ = 0.71073 Å) operating in shutterless mode; or, a Bruker microsource diffractometer equipped with a CCD detector and using mirror-monochromated Mo Kα radiation (λ = 0.71073 Å). APEX III software was used for control and solving the unit cells prior to data collection. Intensities were integrated from data recorded from ω or ω and φ rotation at the frame width and exposure time outlined in Table S2. The crystal data for complexes **1Ln** (Ln = La, Ce, Pr, Nd), **2Ln** (Ln = La, Ce, Pr, Nd, Y), **2Ln^{β}** (Ln = La, Ce), **2Np**, **2Pu**, **4Nd**, **4Am**, and **5Nd** are compiled in **Table S3** to **Table S6**.

Table S2. Data collection parameters and CCDC reference codes for all structures herein.

	Formula	Frame width (°)	Exposure time (s)	CCDC ref. code
**1La**	$[La(Cp^*)_2(I)(THF)]$	0.5	20	2271531
**1Ce**	$[Ce(Cp^*)_2(I)(THF)]$	0.6	60	2271532
**1Pr**	$[Pr(Cp^*)_2(I)(THF)]$	0.5	5	2271533
**1Nd**	$[Nd(Cp^*)_2(I)(THF)]$	0.5	4	2271534
**2La**	$[La(Cp^*)_2(I)(I^{Me4})]$	0.5	10	2271535
**2La^{α}**	$[La(Cp^*)_2(I)(I^{Me4})]\cdot(C_7H_8)$	0.5	15	2271536
**2Ce**	$[Ce(Cp^*)_2(I)(I^{Me4})]$	0.5	4	2271537
**2Ce^{α}**	$[Ce(Cp^*)_2(I)(I^{Me4})]\cdot(C_7H_8)$	0.5	4	2271538
**2Pr**	$[Pr(Cp^*)_2(I)(I^{Me4})]$	0.5	6	2271539
**2Nd**	$[Nd(Cp^*)_2(I)(I^{Me4})]$	0.5	5	2271540
**2Y**	$[Y(Cp^*)_2(I)(I^{Me4})]$	0.5	5	2271541
**2Np**	$[Np(Cp^*)_2(I)(I^{Me4})]$	0.6	80	2271542
**2Pu**	$[Pu(Cp^*)_2(I)(I^{Me4})]$	0.5	60	2271543
**4Nd**	$[Nd(Cp^*)_2(I_xCl_{1-x})(I^{Me4})]$	0.5	20	2271544
**4Am**	$[Am(Cp^*)_2(I_xCl_{1-x})(I^{Me4})]$	0.4	10	2271545
**5Nd**	$[Nd(Cp^*)_2(Cl)(I^{Me4})]$	0.5	15	2271546

CrysAlisPro[19] was used for final unit cell determination and parameters were refined from the observed positions of all strong reflections in each data set. An analytical absorption correction was applied.[19] The Olex2[20] GUI was used for structure solution and refinement utilizing the ShelX

software packages.[21, 22] The structures were solved using ShelXT[21]; the datasets were refined by ShelXL[22] using full-matrix least-squares on all unique F^2 values, with anisotropic displacement parameters for all non-hydrogen atoms, and with constrained riding hydrogen geometries; U_{iso}(H) was set at 1.2 (1.5 for methyl groups if applicable) times Ueq of the parent atom. The largest features in final difference syntheses were close to heavy atoms and were of no chemical significance. Olex2 combined with Inkscape was employed for molecular graphics.[20, 23]. CCDC 2271531–2271546 contain the supplementary crystal data for this article. **1Ln** (Ln = La [2271531], Ce [2271532], Pr [2271533], Nd [2271534]), **2Ln** (Ln = La [2271535], Ce [2271537], Pr [2271539], Nd [2271540], Y [2271541]), **2Ln^{β}** (Ln = La [2271536], Ce [2271538]), **2Np** (2271542), **2Pu** (2271543), **4Nd** (2271544), **4Am** (2271545), and **5Nd** (2271546). These data can be obtained free of charge from the Cambridge Crystallographic Data Centre via www.ccdc.cam.ac.uk/data_request/cif.

The combined error from two individual metrics that have their own associated errors (estimated standard deviation, or standard uncertainty used interchangeably here) can be calculated as the root of the sum of the square of each error (Equation S2). This is not strictly appropriate for combining more than two individual errors.[24]

Equation S2. $$\sigma_{tot} = \sqrt{{\sigma_1}^2 + {\sigma_2}^2}$$

The combined error for the numerical average for multiple (independent) bond lengths, such as the five independent M–C bonds in an M–($\eta^5C_5H_5$) complex, is calculated using the alternate weighted standard deviation from Parsons and Clegg (Equation S3).[24]

Equation S3. $$\sigma_{tot} = 1/\sqrt{\sum_{1\rightarrow n} W_n} \qquad W_n = 1/{\sigma_n}^2$$

Table S3. Crystallographic data for **1Ln** (Ln = La, Ce, Pr, Nd).

	**1La**	**1Ce**	**1Pr**	**1Nd**
Identification code	dq1448	ms0544	ms0641	ms0640
Formula	$LaIOC_{24}H_{38}$	$CeIOC_{24}H_{38}$	$PrIOC_{24}H_{38}$	$NdIOC_{24}H_{38}$
Fw	608.35	609.56	610.35	613.68
Temperature / K	102.5(7)	100.5(7)	100.00(10)	100.00(10)
Crystal system	triclinic	triclinic	triclinic	triclinic
Space group	$P\bar{1}$	$P\bar{1}$	$P\bar{1}$	$P\bar{1}$
a / Å	17.3276(6)	8.7201(5)	8.7057(4)	8.6968(3)
b / Å	18.5043(6)	17.4333(9)	17.3976(9)	17.3670(5)
c / Å	18.7993(6)	18.4296(12)	18.3787(10)	18.3250(5)
α / °	114.393(3)	62.226(6)	62.234(5)	62.368(3)
β / °	111.967(3)	86.918(5)	87.043(4)	87.067(2)
γ / °	92.839(3)	84.319(4)	84.257(4)	84.342(2)
Volume / $Å^3$	4940.3(3)	2466.6(3)	2450.7(2)	2440.09(14)
Z	8	4	4	4
ρ_{calcg} / cm^3	1.636	1.641	1.654	1.671
μ / mm^{-1}	2.99	3.107	3.258	3.404
F(000)	2400	1204	1208	1212
Crystal size / mm^3	0.3 × 0.1 × 0.01	0.2 × 0.05 × 0.05	0.3 × 0.1 × 0.01	0.3 × 0.1 × 0.01
Radiation	Mo Kα (λ = 0.71073)	Mo Kα (λ = 0.71073)	Mo Kα (λ = 0.71073)	Mo Kα (λ = 0.71073)
2Θ range / °	3.144 to 52.756	4.404 to 52.744	4.416 to 52.74	4.416 to 52.742
Index ranges	–21 ≤ h ≤ 21, –23 ≤ k ≤ 23, –23 ≤ l ≤ 23	–10 ≤ h ≤ 10, –21 ≤ k ≤ 21, –23 ≤ l ≤ 23	–10 ≤ h ≤ 9, –21 ≤ k ≤ 21, –22 ≤ l ≤ 22	–10 ≤ h ≤ 10, –21 ≤ k ≤ 21, –22 ≤ l ≤ 22
No. reflections	51034	18538	62306	18988
Unique reflections	19916 [R_{int} = 0.0436, R_σ = 0.0552]	18538 [R_{int} = 0.0692, R_σ = 0.2329]	18965 [R_{int} = 0.0949, R_σ = 0.3489]	18988 [R_{int} = N/A, R_σ = 0.1072]
Data / restraints / parameters	19916 / 649 / 1146	18538 / 712 / 508	18965 / 6 / 508	18988 / 36 / 508
GOOF on F^2	1.057	0.75	0.88	0.988
Final R indexes [I ≥ 2σ (I)][a]	R_1 = 0.0481, wR_2 = 0.0854	R_1 = 0.0464, wR_2 = 0.0730	R_1 = 0.0476, wR_2 = 0.1060	R_1 = 0.0658, wR_2 = 0.1855
Final R indexes [all data]	R_1 = 0.0743, wR_2 = 0.0946	R_1 = 0.0947, wR_2 = 0.0768	R_1 = 0.0777, wR_2 = 0.1098	R_1 = 0.0867, wR_2 = 0.1900
Largest diff. (peak / hole) / e $Å^{-3}$	1.76 / –1.99	1.88 / –1.07	1.28 / –1.09	2.25 / –2.40

[a] $R = \sum||F_O| - |F_C||/\sum|F_O|$; $R_W = [\sum w(F_O^2 - F_C^2)^2/\sum w(F_O^2)^2]^{0.5}$; $S = [\sum w(F_O^2 - F_C^2)^2/(\text{no. data} - \text{no. params})]^{0.5}$ for all data.

Table S4. Crystallographic data for **2Ln** (Ln = La, Ce – also SASBUK[11]), and **2Ln$^{\alpha}$** (Ln = La, Ce).

	**2La**	**2La$^{\beta}$**	**2Ce** [a]	**2Ce$^{\beta}$**
Identification code	ms0625	ms0634	ms0592	ms0543
Formula	$LaIN_2C_{27}H_{42}$	$LaIN_2C_{27}H_{42}\cdot C_7H_8$	$CeIN_2C_{27}H_{42}$	$CeIN_2C_{27}H_{42}\cdot C_7H_8$
Fw	660.43	752.57	661.64	753.78
Temperature / K	100.00(10)	100.00(10)	100.00(10)	100.5(7)
Crystal system	monoclinic	orthorhombic	monoclinic	orthorhombic
Space group	$P2_1/c$	$Cmc2_1$	$P2_1/c$	$Cmc2_1$
a / Å	9.0685(2)	10.9689(3)	9.1500(4)	11.0351(3)
b / Å	16.5041(4)	23.5657(6)	16.4426(4)	23.7152(6)
c / Å	18.7257(4)	12.8034(3)	18.7229(5)	12.8920(3)
β / °	101.152(2)	–	101.207(4)	–
Volume / Å^3	2749.71(11)	3309.55(14)	2763.15(17)	3373.83(15)
Z	4	4	4	4
ρ_{calcg} / cm^3	1.595	1.51	1.59	1.484
μ / mm^{-1}	2.692	2.247	2.78	2.287
F(000)	1312	1512	1316	1516
Crystal size / mm^3	0.1 × 0.05 × 0.05	0.3 × 0.2 × 0.2	0.2 × 0.1 × 0.1	0.2 × 0.2 × 0.05
Radiation	Mo Kα (λ = 0.71073)	Mo Kα (λ = 0.71073)	Mo Kα (λ = 0.71073)	Mo Kα (λ = 0.71073)
2Θ range / °	4.434 to 52.73	4.096 to 52.73	4.538 to 52.742	4.072 to 52.73
Index ranges	$-11 \le h \le 10, -17 \le k \le 20, -23 \le l \le 23$	$-13 \le h \le 12, -28 \le k \le 29, -16 \le l \le 15$	$-11 \le h \le 10, -13 \le k \le 20, -23 \le l \le 23$	$-13 \le h \le 13, -29 \le k \le 29, -16 \le l \le 16$
No. reflections	26498	15861	13224	23608
Unique reflections	5633 [R_{int} = 0.0554, R_{σ} = 0.0399]	3523 [R_{int} = 0.0702, R_{σ} = 0.0510]	5499 [R_{int} = 0.0698, R_{σ} = 0.1142]	3626 [R_{int} = 0.0515, R_{σ} = 0.0340]
Data / restraints / parameters	5633 / 0 / 294	3523 / 1 / 199	5499 / 392 / 294	3626 / 1 / 199
GOOF on F^2	1.034	1.066	0.979	1.04
Final R indexes [$I \ge 2\sigma$ (I)] [b]	R_1 = 0.0285, wR_2 = 0.0586	R_1 = 0.0383, wR_2 = 0.0876	R_1 = 0.0463, wR_2 = 0.0727	R_1 = 0.0217, wR_2 = 0.0441
Final R indexes [all data]	R_1 = 0.0386, wR_2 = 0.0626	R_1 = 0.0406, wR_2 = 0.0893	R_1 = 0.0836, wR_2 = 0.0896	R_1 = 0.0242, wR_2 = 0.0452
Largest diff. (peak / hole) / e Å^{-3}	0.49 / –0.67	1.33 / –0.76	1.63 / –0.97	0.61 / –0.35
Flack parameter	–	–0.01(3)	–	–0.002(11)

[a] [Ce(Cp*)$_2$(I)(I^{Me4})] (**2Ce**) has been reported previously,[11] however the unit cell of **2Ce$^{\beta}$** here is different as it contains one molecule of toluene per formula unit of the complex. [b] $R = \sum||F_O| - |F_C||/\sum|F_O|$; $R_W = [\sum w(F_O^2 - F_C^2)^2/\sum w(F_O^2)^2]^{0.5}$; $S = [\sum w(F_O^2 - F_C^2)^2/(\text{no. data} - \text{no. params})]^{0.5}$ for all data.

Table S5. Crystallographic data for **2Ln** (Ln = Pr, Nd, Y) and **2Np**.

	**2Pr**	**2Nd**	**2Y**	**2Np**
Identification code	ms0624	ms0675	ms0662	dq1402
Formula	$PrIN_2C_{27}H_{42}$	$NdIN_2C_{27}H_{42}$	$YIN_2C_{27}H_{42}$	$NpIN_2C_{27}H_{42}$
Fw	662.43	665.76	610.43	758.52
Temperature / K	100.00(10)	100.00(10)	100.00(10)	117(5)
Crystal system	monoclinic	monoclinic	monoclinic	monoclinic
Space group	$P2_1/c$	$P2_1/c$	$P2_1/c$	$P2_1/c$
a / Å	9.1280(2)	9.13490(10)	9.2733(2)	9.1557(4)
b / Å	16.3688(4)	16.3450(2)	16.0679(3)	16.3521(6)
c / Å	18.6828(4)	18.7053(2)	18.5661(4)	18.6729(7)
β / °	101.228(2)	100.9510(10)	100.765(2)	101.095(4)
Volume / $Å^3$	2738.06(11)	2742.03(5)	2717.71(11)	2743.36(18)
Z	4	4	4	4
ρ_{calcg} / cm^3	1.607	1.613	1.492	1.837
μ / mm^{-1}	2.923	3.035	3.298	4.929
F(000)	1320	1324	1240	1456
Crystal size / mm^3	0.2 × 0.1 × 0.1	0.3 × 0.2 × 0.2	0.2 × 0.2 × 0.1	0.1 × 0.05 × 0.05
Radiation	Mo Kα (λ = 0.71073)	Mo Kα (λ = 0.71073)	Mo Kα (λ = 0.71073)	Mo Kα (λ = 0.71073)
2Θ range / °	3.336 to 52.796	4.542 to 69.846	5.262 to 52.742	5.708 to 52.884
Index ranges	–11 ≤ h ≤ 11, –20 ≤ k ≤ 20, –23 ≤ l ≤ 23	–14 ≤ h ≤ 14, –25 ≤ k ≤ 26, –28 ≤ l ≤ 26	–11 ≤ h ≤ 10, –20 ≤ k ≤ 20, –23 ≤ l ≤ 23	–11 ≤ h ≤ 11, –20 ≤ k ≤ 20, –6 ≤ l ≤ 23
No. reflections	9474	69815	23177	5423
Unique reflections	9474 [R_{int} = 0.0774, R_σ = 0.1548]	10935 [R_{int} = 0.0622, R_σ = 0.0456]	5517 [R_{int} = 0.0484, R_σ = 0.0413]	5423 [R_{int} = N/A, R_σ = 0.0431]
Data / restraints / parameters	9474 / 0 / 295	10935 / 0 / 294	5517 / 0 / 294	5423 / 571 / 295
GOOF on F^2	0.954	1.044	1.053	1.149
Final R indexes [I ≥ 2σ (I)][a]	R_1 = 0.0369, wR_2 = 0.1001	R_1 = 0.0335, wR_2 = 0.0763	R_1 = 0.0318, wR_2 = 0.0690	R_1 = 0.0511, wR_2 = 0.0848
Final R indexes [all data]	R_1 = 0.0678, wR_2 = 0.1033	R_1 = 0.0566, wR_2 = 0.0846	R_1 = 0.0441, wR_2 = 0.0746	R_1 = 0.0722, wR_2 = 0.0925
Largest diff. (peak / hole) / e $Å^{-3}$	0.95 / –0.70	1.46 / –1.72	0.67 / –0.53	1.69 / –1.39

[a] $R = \sum||F_O| - |F_C||/\sum|F_O|$; $R_W = [\sum w(F_O^2 - F_C^2)^2/\sum w(F_O^2)^2]^{0.5}$; $S = [\sum w(F_O^2 - F_C^2)^2/(\text{no. data} - \text{no. params})]^{0.5}$ for all data.

Table S6. Crystallographic data for **2Pu**, **4Nd**, **4Am**, and **5Nd**.

	**2Pu**	**4Nd**	**4Am**	**5Nd**
Identification code	ms0552	ms0607	ms0657	ms0664
Formula	$PuIN_2C_{27}H_{42}$	$NdI_xCl_{1-x}N_2C_{27}H_{42}$	$AmI_xCl_{1-x}N_2C_{27}H_{42}$	$NdClN_2C_{27}H_{42}$
		$X = 0.66$	$X = 0.65$	
Fw	763.52	635.36	732.52	574.31
Temperature / K	100.5(7)	100.5(7)	100.00(10)	100.00(10)
Crystal system	monoclinic	monoclinic	monoclinic	triclinic
Space group	$P2_1/c$	$P2_1/c$	$P2_1/c$	$P\bar{1}$
a / Å	9.1883(6)	9.2747(6)	9.19154(18)	9.66860(10)
b / Å	16.3097(9)	16.4196(9)	16.2811(3)	16.3387(2)
c / Å	18.6693(10)	18.7011(12)	18.5401(4)	17.7436(3)
	–	–	–	86.2390(10)
β / °	101.217(6)	100.631(6)	100.770(2)	77.2040(10)
	–	–	–	81.4050(10)
Volume / Å^3	2744.3(3)	2799.0(3)	2725.64(10)	2701.15(6)
Z	4	4	4	4
ρ_{calcg} / cm^3	1.848	1.508	1.785	1.412
μ / mm^{-1}	3.543	2.638	3.601	2.037
F(000)	1460	1276	1414	1180
Crystal size / mm^3	0.1 × 0.05 × 0.05	0.15 × 0.1 × 0.02	0.1 × 0.1 × 0.1	0.2 × 0.1 × 0.01
Radiation	Mo Kα (λ = 0.71073)	Mo Kα (λ = 0.71073)	Mo Kα (λ = 0.71073)	Mo Kα (λ = 0.71073)
2Θ range / °	3.344 to 52.77	5.232 to 52.858	5.276 to 52.734	5.334 to 52.744
Index ranges	–11 ≤ h ≤ 11, –20 ≤ k ≤ 20, –22 ≤ l ≤ 23	–11 ≤ h ≤ 11, –20 ≤ k ≤ 20, –23 ≤ l ≤ 23	–11 ≤ h ≤ 11, –20 ≤ k ≤ 20, –23 ≤ l ≤ 23	–12 ≤ h ≤ 12, –20 ≤ k ≤ 20, –22 ≤ l ≤ 22
No. reflections	5563	34038	70671	74234
Unique reflections	5563 [R_{int} = N/A, R_σ = 0.0840]	9698 [R_{int} = 0.0875, R_σ = 0.1159]	5565 [R_{int} = 0.0591, R_σ = 0.0210]	11042 [R_{int} = 0.0606, R_σ = 0.0320]
Data / restraints / parameters	5563 / 0 / 295	9698 / 0 / 305	5565 / 0 / 304	11042 / 990 / 779
GOOF on F^2	1.116	0.888	1.091	1.052
Final R indexes [I ≥ 2σ (I)][a]	R_1 = 0.0522, wR_2 = 0.0852	R_1 = 0.0444, wR_2 = 0.0959	R_1 = 0.0233, wR_2 = 0.0543	R_1 = 0.0288, wR_2 = 0.0687
Final R indexes [all data]	R_1 = 0.0801, wR_2 = 0.0943	R_1 = 0.0887, wR_2 = 0.1016	R_1 = 0.0302, wR_2 = 0.0567	R_1 = 0.0339, wR_2 = 0.0720
Largest diff. (peak / hole) / e Å^{-3}	1.73 / –1.45	0.80 / –0.69	1.19 / –0.53	1.48 / –0.90

[a] $R = \sum||F_O| - |F_C|| / \sum|F_O|$; $R_W = [\sum w(F_O^2 - F_C^2)^2 / \sum w(F_O^2)^2]^{0.5}$; $S = [\sum w(F_O^2 - F_C^2)^2/(\text{no. data} - \text{no. params})]^{0.5}$ for all data.

S4. Molecular structures

*Complexes **1Ln** (Ln = La, Ce, Pr, Nd)*

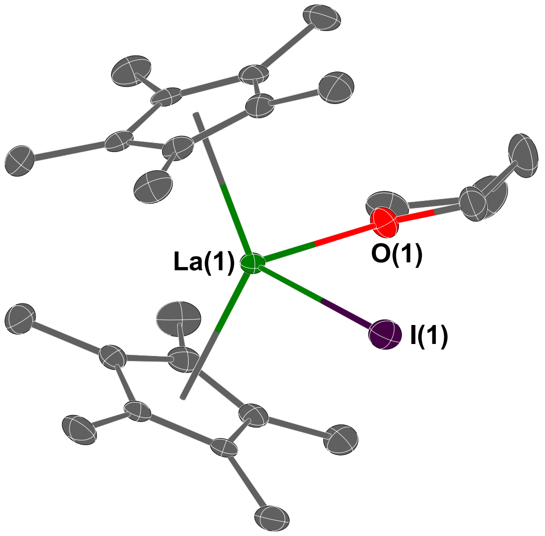

Figure S28. Molecular structure of **1La**. Ellipsoids set at 50% probability and H-atoms, and a second unit containing La(2), removed for clarity (operations: X, Y, Z).

La(1)–I(1) = 3.1400(6) Å; La(1)–O(1) = 2.536(4) Å; La(1)–Cp_{cent} = 2.562(4) Å; La(1)–Cp_{cent} = 2.542(16) Å; La(1)–CpC_{range} = 2.75(3)–2.91(3) Å; I(1)–La(1)–O(1) = 85.72(10)°; $\angle_{plane\cdots plane}$ Cp_{cent}–La–Cp_{cent} = 138.08(17)°.

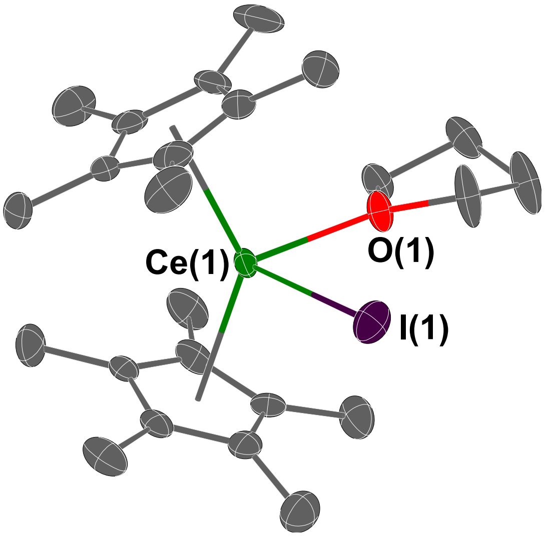

Figure S29. Molecular structure of **1Ce**. Ellipsoids set at 50% probability and H-atoms, and a second unit containing Ce(2), removed for clarity (operations: X, Y, Z).

Ce(1)–I(1) = 3.1027(8) Å; Ce(1)–O(1) = 2.511(5) Å; Ce(1)–Cp_{cent} = 2.497(5) Å; Ce(1)–Cp_{cent} = 2.506(4) Å; Ce(1)–CpC_{range} = 2.757(7)–2.806(7) Å; I(1)–Ce(1)–O(1) = 86.71(11)°; Cp_{cent}–Ce–Cp_{cent} = 136.04(11)°; $\angle_{plane\cdots plane}$ I(1)–Ce(1)–O(1)···Cp_{cent}–Ce(1)–Cp_{cent} = 88.89(13)°.

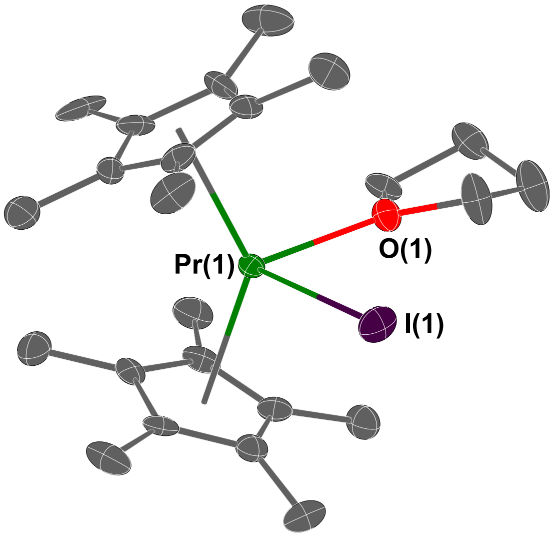

Figure S30. Molecular structure of **1Pr**. Ellipsoids set at 50% probability and H-atoms, and a second unit containing Pr(2), removed for clarity (operations: X, Y, Z).

Pr(1)–I(1) = 3.0851(8) Å; Pr(1)–O(1) = 2.491(5) Å; Pr(1)–Cp_{cent} = 2.473(5) Å; Pr(1)–Cp_{cent} = 2.500(5) Å; Pr(1)–CpC_{range} = 2.745(8)–2.805(8) Å; I(1)–Pr(1)–O(1) = 86.78(13)°; Cp_{cent}–Pr(1)–Cp_{cent} = 135.92(12)°; $\angle_{plane\cdots plane}$ I(1)–Pr(1)–O(1)···Cp_{cent}–Pr(1)–Cp_{cent} = 88.69(14)°.

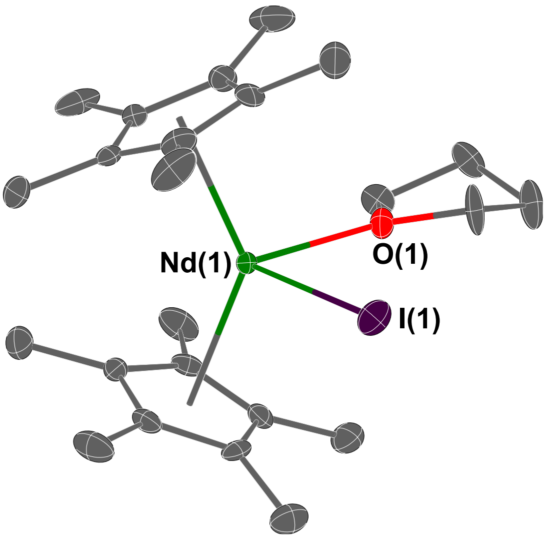

Figure S31. Molecular structure of **1Nd**. Ellipsoids set at 50% probability and H-atoms, and a second unit containing Nd(2), removed for clarity (operations: X, Y, Z).

Nd(1)–I(1) = 3.0618(10) Å; Nd(1)–O(1) = 2.461(8) Å; Nd(1)–Cp_{cent} = 2.467(6) Å; Nd(1)–Cp_{cent} = 2.471(7) Å; Nd(1)–CpC_{range} = 2.731(11)–2.785(11) Å; I(1)–Nd(1)–O(1) = 87.27(19)°; Cp_{cent}–Nd–Cp_{cent} = 135.48(16)°; $\angle_{plane\cdots plane}$ I(1)–Nd(1)–O(1)···Cp_{cent}–Nd(1)–Cp_{cent} = 88.6(2).

*Bond metrics for **1La***

Table S7. Bond lengths (Å) and angles (°) for **1La** for all 4 unique metal sites.

	**La1** ‡	**La2**	**La3**	**La4**
**M–I**	3.1400(6)	3.1492(5)	3.1485(5)	3.1613(6)
**M–O**	2.536(4)	2.531(4)	2.520(4)	2.533(4)
**M–Cp_{cent}**	2.562(4)	2.534(3)	2.534(3)	2.520(4)
	2.542(16)	2.540(3)	2.544(3)	2.543(3)
**M–CpC_{range}**	2.75(3)–	2.786(5)–	2.785(5)–	2.778(6)–
	2.91(3)	2.826(5)	2.836(6)	2.828(6)
**I–M–O**	85.72(10)	90.65(8)	90.27(8)	89.36(10)
**Cp_{cent}–M–Cp_{cent}**	138.08(17)	135.25(9)	135.54(9)	134.37(8)
$\angle_{plane\cdots plane}$ **I–M–O···Cp_{cent}–M–Cp_{cent}**	90.72(17)	91.22(9)	90.87(9)	91.13(11)

‡ Only the largest component of the disordered La(1)–Cp rings is listed.

*Bond metrics for **1Ln** (Ln = Ce, Pr, Nd)*

Table S8. Bond lengths (Å) and angles (°) for **1Ln** (Ln = Ce, Pr, Nd).

		**1Ce**	**1Pr**	**1Nd**
**M–I**	M(1)	3.1027(8)	3.0851(8)	3.0618(10)
	M(2)	3.1272(7)	3.1070(8)	3.0896(10)
**M–O**	M(1)	2.511(5)	2.491(5)	2.461(8)
	M(2)	2.507(4)	2.499(5)	2.453(8)
**M–Cp_{cent}**	M(1)	2.497(5)	2.473(5)	2.467(6)
		2.521(4)	2.500(5)	2.471(7)
	M(2)	2.506(4)	2.484(4)	2.456(7)
		2.518(4)	2.497(5)	2.483(6)
**M–CpC_{range}**	M(1)	2.757(7)–2.806(7)	2.745(8)–2.805(8)	2.731(11)–2.785(11)
	M(2)	2.760(7)–2.805(7)	2.739(8)–2.791(7)	2.729(10)–2.768(12)
**I–M–O**	M(1)	86.71(11)	86.78(13)	87.27(19)
	M(2)	91.18(10)	90.75(13)	90.98(18)
**Cp_{cent}–M–Cp_{cent}**	M(1)	136.04(11)	135.92(12)	135.48(16)
	M(2)	136.1(1)	136.31(11)	135.03(16)
$\angle_{plane\cdots plane}$ **I–M–O···Cp_{cent}–M–Cp_{cent}**	M(1)	88.89(13)	88.69(14)	88.6(2)
	M(2)	88.95(12)	89.27(13)	89.4(2)

*Bond metrics for previously-reported **1An** (An = U, Np, Pu)*

Table S9. Bond lengths (Å) and angles (°) for **1An** (Ln = U, Np, Pu).[3, 25]

		1U	1Np	1Pu
**M–I**	M(1)	3.0636(6)	3.0560(14)	3.0353(7)
	M(2)	3.0955(6)	3.0832(12)	3.0594(6)
**M–O**	M(1)	2.496(4)	2.504(13)	2.460(5)
	M(2)	2.486(4)	2.472(12)	2.463(6)
**M–Cp_{cent}**	M(1)	2.476(4)	2.530(5)	2.455(6)
		2.494(4)	2.561(5)	2.463(5)
	M(2)	2.487(3)	2.556(5)	2.461(4)
		2.491(4)	2.566(4)	2.473(5)
**M–CpC_{range}**	M(1)	2.739(6)–	2.781(8)–	2.712(8)–
		2.808(6)	2.839(7)	2.766(7)
	M(2)	2.748(6)–	2.778(8)–	2.723(8)–
		2.791(5)	2.862(7)	2.768(7)
**I–M–O**	M(1)	87.30(11)	87.7(3)	87.75(13)
	M(2)	91.70(9)	91.1(3)	91.49(13)
**Cp_{cent}–M–Cp_{cent}**	M(1)	135.56(9)	135.12(11)	135.49(13)
	M(2)	135.66(9)	135.49(10)	135.78(12)
**∠$_{plane\cdots plane}$ I–M–O···Cp_{cent}–M–Cp_{cent}**	M(1)	88.85(13)	88.6(3)	89.14(15)
	M(2)	89.23(10)	89.1(3)	89.12(14)

*Complexes **2Ln** (Ln = La, Ce, Pr, Nd, Y)*

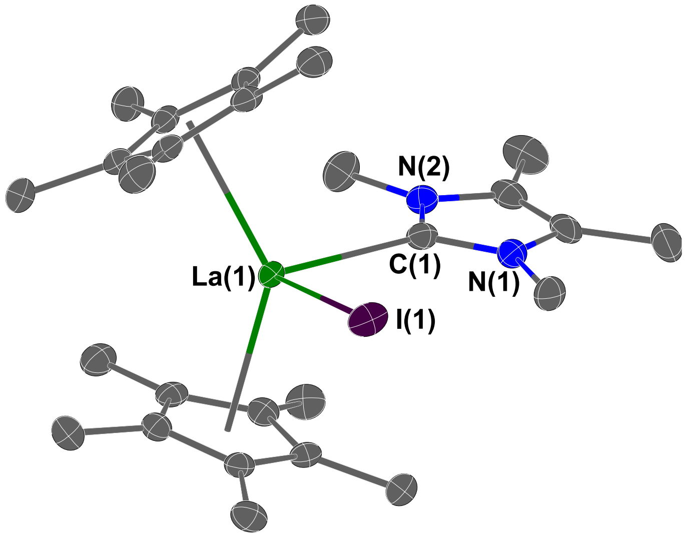

Figure S32. Molecular structure of **2La**. Ellipsoids set at 50% probability and H-atoms removed for clarity (operations: X, Y, Z).

La(1)–I(1) = 3.1769(3) Å; La(1)–C(1) = 2.751(3) Å; La(1)–Cp_{cent} = 2.5590(17) Å; La(1)–Cp_{cent} = 2.5723(15) Å; La–CpC_{range} = 2.806(3)–2.880(3) Å; I(1)–La(1)–C(1) = 99.83(7)°; Cp_{cent}–La–Cp_{cent} = 135.45(5)°; $\angle_{plane\cdots plane}$ I(1)–La(1)–C(1)···Cp_{cent}–La(1)–Cp_{cent} = 91.79(6)°.

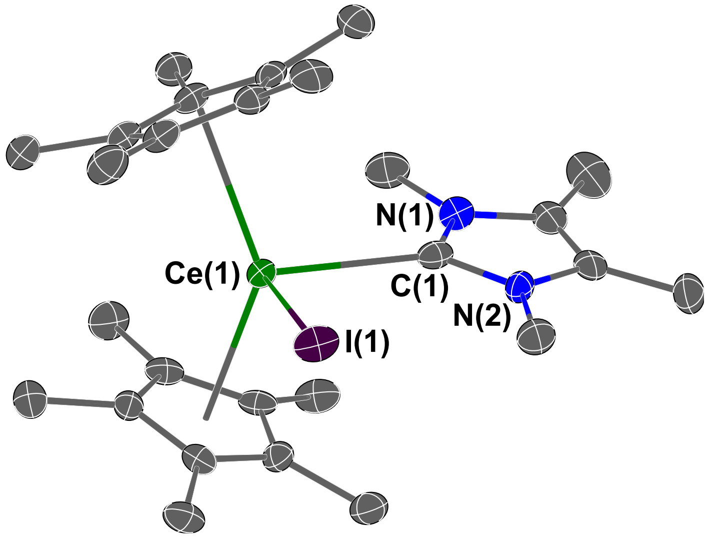

Figure S33. Molecular structure of **2Ce**. Ellipsoids set at 50% probability and H-atoms removed for clarity (operations: X, Y, Z).

Ce(1)–I(1) = 3.1584(6) Å; Ce(1)–C(1) = 2.724(7) Å; Ce(1)–Cp_{cent} = 2.534(4) Å; Ce(1)–Cp_{cent} = 2.547(3) Å; Ce–CpC_{range} = 2.780(6)–2.853(6) Å; I(1)–Ce(1)–C(1) = 99.42(14)°; Cp_{cent}–Ce–Cp_{cent} = 135.92(11)°; $\angle_{plane\cdots plane}$ I(1)–Ce(1)–C(1)···Cp_{cent}–Ce(1)–Cp_{cent} = 91.76(11)°.

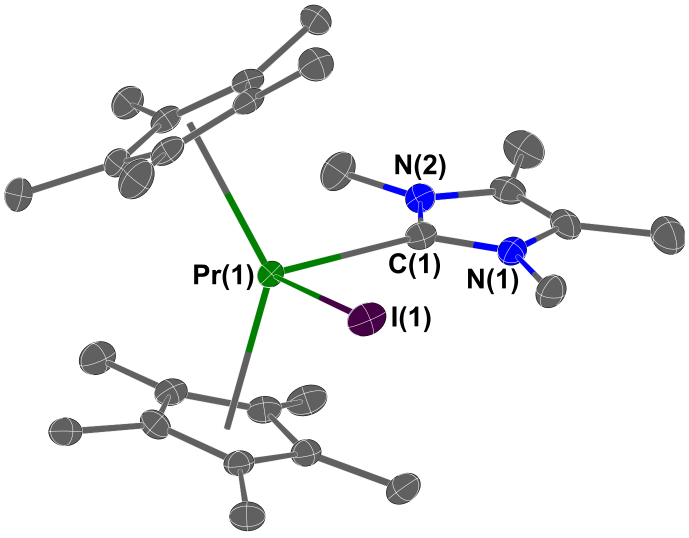

Figure S34. Molecular structure of **2Pr**. Ellipsoids set at 50% probability and H-atoms removed for clarity (operations: X, Y, Z).

Pr(1)–I(1) = 3.1393(6) Å; Pr(1)–C(1) = 2.700(7) Å; Pr(1)–Cp_{cent} = 2.511(3) Å; Pr(1)–Cp_{cent} = 2.529(3) Å; Pr–CpC_{range} = 2.766(7)–2.839(7) Å; I(1)–Pr(1)–C(1) = 99.45(15)°; Cp_{cent}–Pr–Cp_{cent} = 135.95(11)°; $\angle_{plane\cdots plane}$ I(1)–Pr(1)–C(1)···Cp_{cent}–Pr(1)–Cp_{cent} = 91.57(12)°.

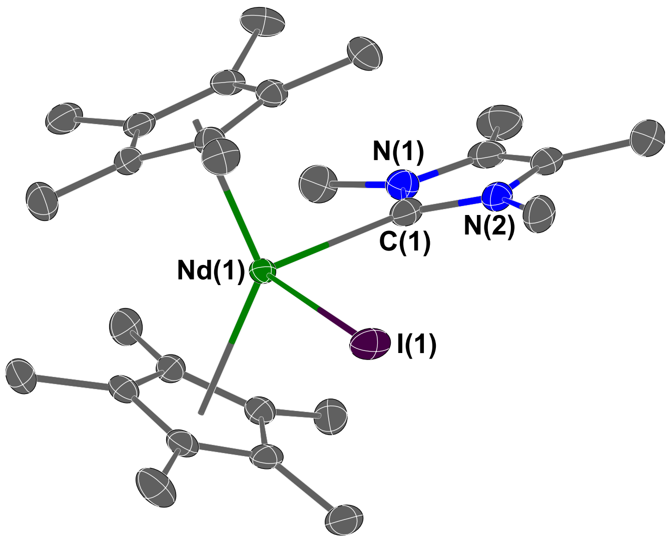

Figure S35. Molecular structure of **2Nd**. Ellipsoids set at 50% probability and H-atoms removed for clarity (operations: X, Y, Z).

Nd(1)–I(1) = 3.1209(2) Å; Nd(1)–C(1) = 2.693(3) Å; Nd(1)–Cp_{cent} = 2.4941(13) Å; Nd(1)–Cp_{cent} = 2.5081(12) Å; Nd–CpC_{range} = 2.745(3)–2.818(3) Å; I(1)–Nd(1)–C(1) = 99.59(6)°; Cp_{cent}–Nd–Cp_{cent} = 135.30(4)°; $\angle_{plane\cdots plane}$ I(1)–Nd1)–C(1)···Cp_{cent}–Nd(1)–Cp_{cent} = 91.75(4)°.

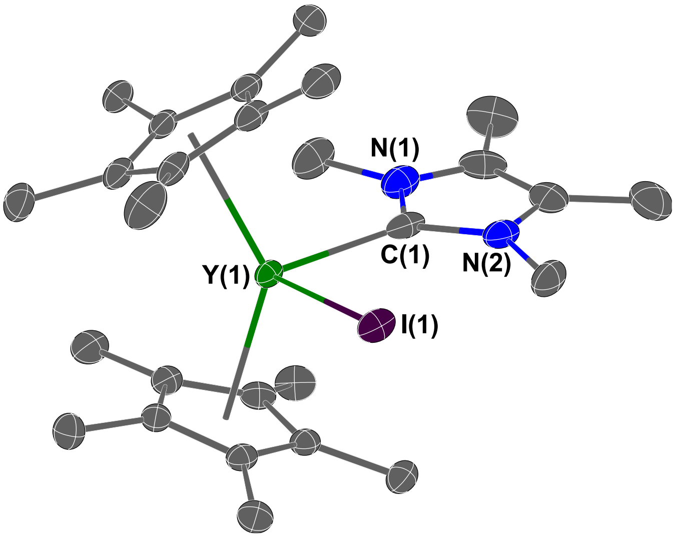

Figure S36. Molecular structure of **2Y**. Ellipsoids set at 50% probability and H-atoms removed for clarity (operations: X, Y, Z).

Y(1)–I(1) = 3.0409(4) Å; Y(1)–C(1) = 2.583(4) Å; Y(1)–Cp_{cent} = 2.3967(15) Å; Y(1)–Cp_{cent} = 2.4126(15) Å; Y(1)–CpC_{range} = 2.664(3)–2.729(3) Å; I(1)–Y(1)–C(1) = 98.20(7)°; Cp_{cent}–Y(1)–Cp_{cent} = 135.89(6)°; $\angle_{plane\cdots plane}$ I(1)–Y(1)–C(1)···Cp_{cent}–Y(1)–Cp_{cent} = 91.35(6)°.

*Bond metrics for **2Ln** (Ln = La, Ce, Pr, Nd, Y)*

Table S10. Bond lengths (Å) and angles (°) for **2Ln** (Ln = La, Ce, Pr, Nd, Y).

	**2La**	**2Ce**	**2Pr**	**2Nd**	**2Y**
**M–I**	3.1769(3)	3.1584(6)	3.1393(6)	3.1209(2)	3.0409(4)
**M–C(1)**	2.751(4)	2.724(7)	2.700(7)	2.693(3)	2.583(4)
**M–Cp_{cent}**	2.5590(17)<br>2.5723(15)	2.534(4)<br>2.547(3)	2.511(3)<br>2.529(3)	2.4941(13)<br>2.5081(12)	2.3967(15)<br>2.4126(15)
**M–CpC_{range}**	2.806(3)–<br>2.880(3)	2.780(6)–<br>2.853(6)	2.766(7)–<br>2.839(7)	2.745(3)–<br>2.818(3)	2.664(3)–<br>2.729(3)
**I–M–C(1)**	99.83(7)	99.42(14)	99.45(15)	99.59(6)	98.20(7)
**Cp_{cent}–M–Cp_{cent}**	135.45(5)	135.92(11)	135.95(11)	135.30(4)	135.89(6)
**$\angle_{plane\cdots plane}$ I–M–C···Cp_{cent}–M–Cp_{cent}**	91.79(6)	91.76(11)	91.57(12)	91.75(4)	91.35(6)

*Bond metric comparison for **2Ln** and **2Ln$^{\beta}$** (Ln = La, Ce).*

Table S11. Bond lengths (Å) and angles (°) comparison between **2Ln** and **2Ln$^{\alpha}$** (Ln = La, Ce).

	**2La**	**2La$^{\beta}$**	**2Ce**	**2Ce$^{\beta}$**
**M–I**	3.1757(3)	3.1930(9)	3.1553(4)	3.1960(5)
**M–C(1)**	2.752(3)	2.736(12)	2.726(5)	2.736(7)
**M–Cp$_{cent}$**	2.5576(17)<br>2.5708(15)	2.560(3)	2.533(3)<br>2.540(2)	2.5525(19)
**M–CpC$_{range}$**	2.804(4)–<br>2.878(3)	2.814(8)–<br>2.845(8)	2.782(5)–<br>2.851(5)	2.810(4)–<br>2.839(4)
**C(1)–M–I**	99.80(7)	103.1(2)	99.49(10)	102.79(14)
**Cp$_{cent}$–M–Cp$_{cent}$**	135.45(5)	138.16(17)	135.85(8)	138.64(10)
**∠$_{plane\cdots plane}$**<br>**I–M–C···Cp$_{cent}$–M–Cp$_{cent}$**	91.79(6)	90.0	91.74(11)	90.0

Note: Complexes **2Ln$^{\beta}$** are isostructural to **2Ln**, but feature a lattice toluene molecule and a mirror plane that bisects both the molecule (along the C(1)–M–I(1) axis) and bisects toluene molecule along H_3C–(centroid)–C_{para} line. This necessarily fixes the I–M–C···Cp$_{cent}$–M–Cp$_{cent}$ angle to be 90.0°.

*Complexes **2Np** and **2Pu***

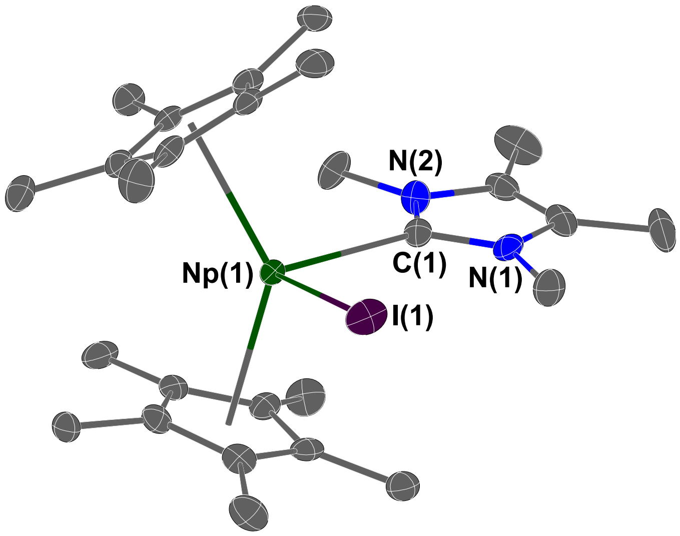

Figure S37. Molecular structure of **2Np**. Ellipsoids set at 50% probability and H-atoms removed for clarity (operations: X, Y, Z).

Np(1)–I(1) = 3.1003(7) Å; Np(1)–C(1) = 2.670(9) Å; Np(1)–Cp_{cent} = 2.495(4) Å; Np(1)–Cp_{cent} = 2.518(4) Å; Np(1)–CpC_{range} = 2.760(8)–2.818(8) Å; I(1)–Np(1)–C(1) = 99.95(18)°; Cp_{cent}–Np(1)–Cp_{cent} = 134.82(14)°; $\angle_{plane\cdots plane}$ I(1)–Np(1)–C(1)···Cp_{cent}–Np(1)–Cp_{cent} = 88.34(14)°.

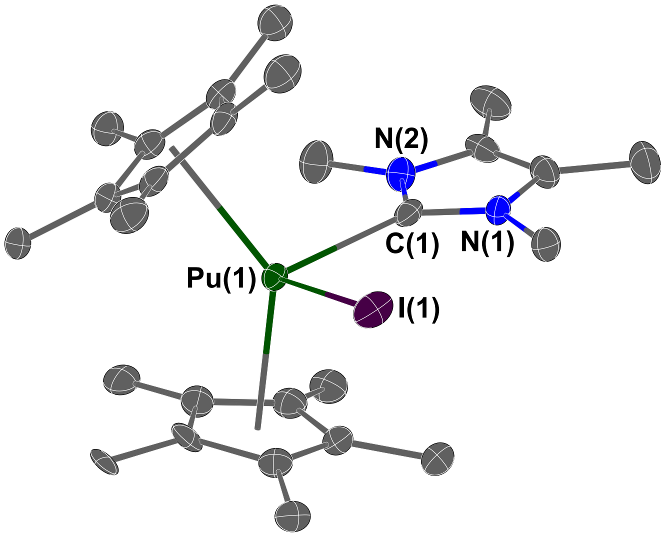

Figure S38. Molecular structure of **2Pu**. Ellipsoids set at 50% probability and H-atoms removed for clarity (operations: X, Y, Z).

Pu(1)–I(1) = 3.0938(7) Å; Pu(1)–C(1) = 2.637(8) Å; Pu(1)–Cp_{cent} = 2.489(4) Å; Pu(1)–Cp_{cent} = 2.510(4) Å; Pu(1)–CpC_{range} = 2.745(8)–2.820(8) Å; I(1)–Pu(1)–C(1) = 99.79(18)°; Cp_{cent}–Pu(1)–Cp_{cent} = 135.47(13)°; $\angle_{plane\cdots plane}$ I(1)–Pu(1)–C(1)···Cp_{cent}–Pu(1)–Cp_{cent} = 88.16(14)°.

*Bond metrics for **2An** (An = U, Np, Pu)*

Table S12. Bond lengths (Å) and angles (°) for **2An** (Ln = U, Np, Pu).

	**2U** ‡	**2Np**	**2Pu**
**M–I**	3.1266(4)	3.1003(7)	3.0938(7)
**M–C(1)**	2.687(5)	2.670(9)	2.637(8)
**M–Cp_{cent}**	2.514(3)<br>2.532(2)	2.495(4)<br>2.518(4)	2.489(4)<br>2.510(4)
**M–CpC_{range}**	2.765(5)–<br>2.841(5)	2.760(8)–<br>2.818(8)	2.745(8)–<br>2.820(8)
**I–M–C(1)**	100.33(11)	99.95(18)	99.79(18)
**Cp_{cent}–M–Cp_{cent}**	135.20(8)	134.82(14)	135.47(13)
**∠$_{plane \cdots plane}$**<br>**I–M–C···Cp_{cent}–M–Cp_{cent}**	91.96(9)	88.34(14)	88.16(14)

‡ **2U** has been reported previously,[11] and so we have reproduced the reported values here for ease of comparison.

*Comparison of **1M** to **2M** (M = La, Ce, Pr, Nd, U, Np, Pu)*

Table S13. Bond lengths (Å) and angles (°) for **1M** (M = La, Ce, Pr, Nd, U, Np, Pu).

		**1La**	**1U**	**1Ce**	**1Np**	**1Pr**	**1Pu**	**1Nd**
**M–I**	M(1)	3.1400(6)	3.0636(6)	3.1027(8)	3.056(14)	3.0851(8)	3.0353(7)	3.0618(10)
	M(2)	3.1492(5)	3.0955(6)	3.1272(7)	3.0832(12)	3.1070(8)	3.0594(6)	3.0896(10)
**M–O** or **M–C$_{NHC}$**	M(1)	2.536(4)	2.496(4)	2.511(5)	2.504(13)	2.491(5)	2.460(5)	2.461(8)
	M(2)	2.531(4)	2.486(4)	2.507(4)	2.472(12)	2.499(5)	2.463(6)	2.453(8)
**Cp$_{cent}$···M···Cp$_{cent}$**	M(1)	138.08(17)	135.56(9)	136.04(11)	135.12(11)	135.92(12)	135.49(13)	135.48(16)
	M(2)	135.25(9)	135.66(9)	136.1(1)	135.49(10)	136.31(11)	135.78(12)	135.03(16)
**% uncovered**	M(1)	9.907	11.378	11.958	10.410	11.378	10.449	10.875
	M(2)	11.881	11.339	12.152	10.604	11.610	10.797	11.339

Note: Lanthanide columns have been shaded to aid comparison.

Table S14. Bond lengths (Å) and angles (°) for **2M** (M = La, Ce, Pr, Nd, U, Np, Pu).

		**2La**	**2U**	**2Ce**	**2Np**	**2Pr**	**2Pu**	**2Nd**
**M–I**	M(1)	3.1769(3)	3.1266(4)	3.1584(6)	3.1003(7)	3.1393(6)	3.0938(7)	3.1209(2)
**M–O** or **M–C$_{NHC}$**	M(1)	2.751(4)	2.687(5)	2.724(7)	2.670(9)	2.700(7)	2.637(8)	2.693(3)
**Cp$_{cent}$···M···Cp$_{cent}$**	M(1)	135.45(5)	135.20(8)	135.92(11)	134.82(14)	135.95(11)	135.47(13)	135.30(4)
**% uncovered**	M(1)	11.726	11.068	11.184	10.449	10.294	10.101	10.178

Note: Lanthanide columns have been shaded to aid comparison.

*Complexes **4Nd** and **4Am***

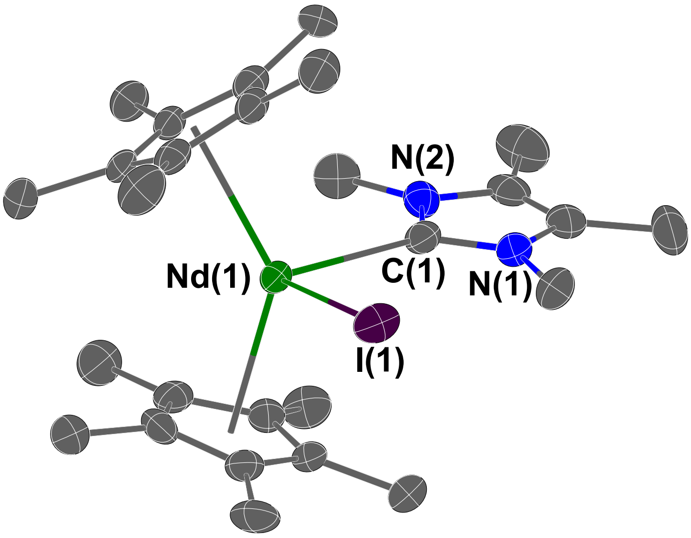

Figure S39. Molecular structures of the iodo component of **4Nd** (**4Nd-I**). Ellipsoids set at 50% probability, H-atoms, and Cl(1), removed for clarity (operations: X, Y, Z).

Nd(1)–I(1) = 3.103(2) Å; Nd(1)–C(1) = 2.683(7) Å; Nd(1)–Cp_{cent} = 2.500(4) Å; Nd(1)–Cp_{cent} = 2.511(3) Å; Nd–CpC_{range} = 2.741(7)–2.816(7) Å; I(1)–Nd(1)–C(1) = 99.29(16)°; Cp_{cent}–Nd–Cp_{cent} = 135.18(11)°; $\angle_{plane\cdots plane}$ I(1)–Nd(1)–C(1)···Cp_{cent}–Nd(1)–Cp_{cent} = 89.14(12)°.

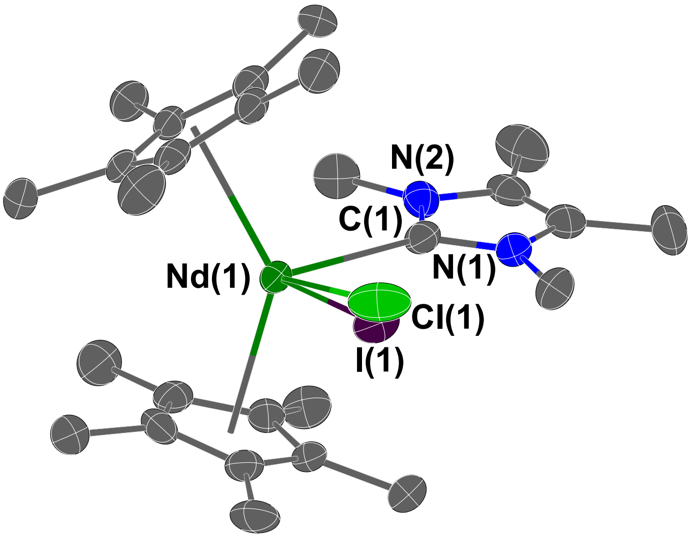

Figure S40. Molecular structure of the full model of **4Nd**. Ellipsoids set at 50% probability and H-atoms removed for clarity (operations: X, Y, Z).

Nd(1)–Cl(1) = 2.73(2) Å; Cl(1)–Nd(1)–C(1) = 96.2(6)°.

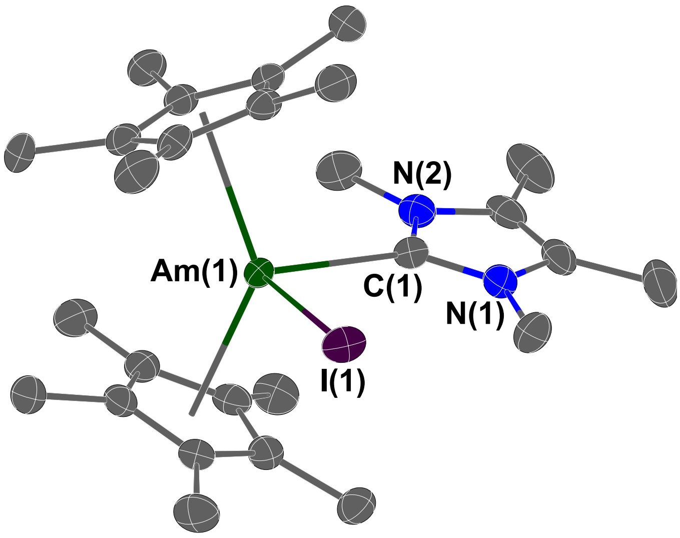

Figure S41. Molecular structures of the iodo component of **4Am** (**4Am-I**). Ellipsoids set at 50% probability, H-atoms, and Cl(1), removed for clarity (operations: X, Y, Z).

Am(1)–I(1) = 3.0666(11) Å; Am(1)–C(1) = 2.631(3) Å; Am(1)–Cp_{cent} = 2.4744(17) Å; Am(1)–Cp_{cent} = 2.4895(15) Å; Am–CpC_{range} = 2.718(3)–2.802(3) Å; I(1)–Am(1)–C(1) = 99.83(8)°; Cp_{cent}–Am–Cp_{cent} = 135.64(6)°; $\angle_{plane\cdots plane}$ I(1)–Am(1)–C(1)···Cp_{cent}–Am(1)–Cp_{cent} = 91.10(6)°.

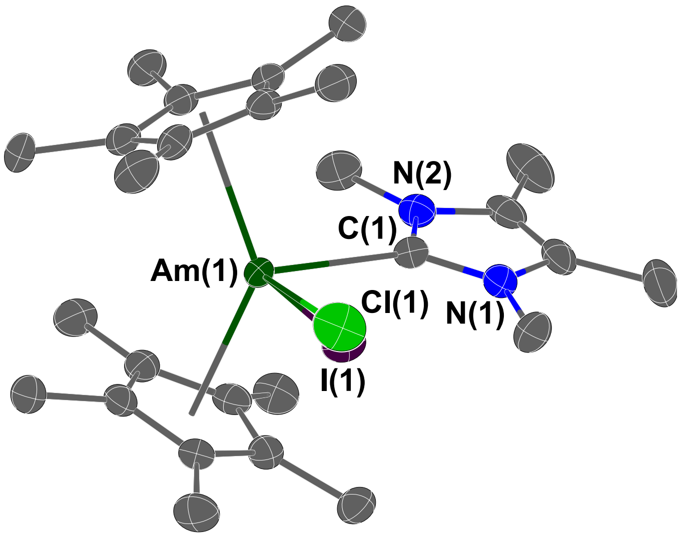

Figure S42. Molecular structure of the full model of **4Am**. Ellipsoids set at 50% probability, H-atoms removed for clarity (operations: X, Y, Z).

Am(1)–Cl(1) = 2.682(8) Å; Cl(1)–Am(1)–C(1) = 98.0(3)°.

Complex ***5Nd***

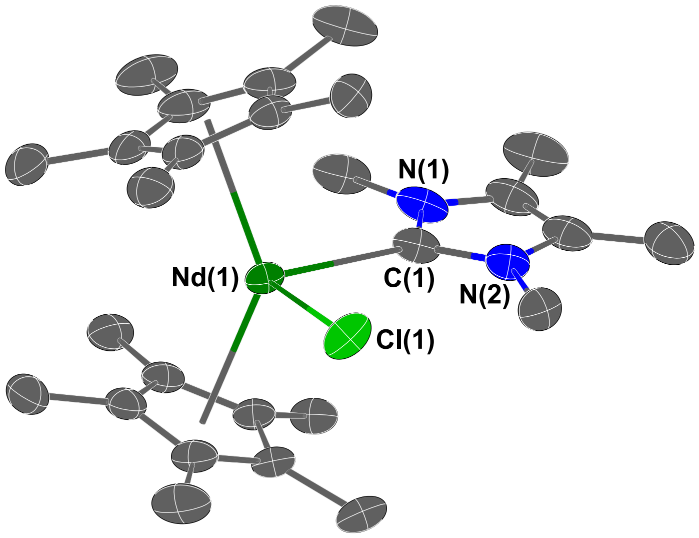

Figure S43. Molecular structure of **5Nd**. Ellipsoids set at 50% probability, H-atoms, and a second unit containing Nd(2), removed for clarity (operations: X, Y, Z).

Nd(1)–Cl(1) = 2.6730(7) Å; Nd(1)–C(1) = 2.667(3) Å; Nd(1)–Cp$_{cent}$ = 2.4858(13) Å; Nd(1)–Cp$_{cent}$ = 2.540(15) Å; Nd–CpC$_{range}$ = 2.74(4)–2.85(3) Å; Cl(1)–Nd(1)–C(1) = 98.23(7)°; Cp$_{cent}$–Nd–Cp$_{cent}$ = 134.3(4)°; $\angle_{plane\cdots plane}$ I(1)–Nd(1)–C(1)···Cp$_{cent}$–Nd(1)–Cp$_{cent}$ = 94.22(17)°.

*Bond metrics comparison for **2Nd**, **4Nd**, **4Am**, and **5Nd**.*

Table S15. Bond lengths (Å) and angles (°) for **4Nd**, **4Am**, and **5Nd**.

	**2Nd**	**4Nd**	**4Am**	**5Nd** Nd(1)	**5Nd** Nd(2)
**M–X** X = I	3.1209(2)	3.103(2)	3.0666(11)		
X = Cl		2.73(2)	2.682(8)	2.6730(7)	2.6976(7)
**M–C(1)**	2.693(3)	2.683(7)	2.631(3)	2.667(3)	2.708(3)
**$\Delta_{M–C(1)}$ w.r.t. 4Am**	+0.062(4)	+0.052(8)	–	+0.036(4)	+0.077(4)
**M–Cp_{cent}**	2.4941(13)<br>2.5081(12)	2.500(4)<br>2.511(3)	2.4744(17)<br>2.4895(15)	2.4858(13)<br>2.540(15)	2.4889(16)<br>2.499(3)
**M–CpC_{range}**	2.745(3)–<br>2.818(3)	2.741(7)–<br>2.816(7)	2.718(3)–<br>2.802(3)	2.74(4)–<br>2.85(3)	2.723(3)–<br>2.805(3)
**X–M–C(1)** X = I	99.59(6)	99.29(16)	99.83(8)		
X = Cl		96.2(6)	98.0(3)	98.23(7)	96.96(6)
**Cp_{cent}–M–Cp_{cent}**	135.30(4)	135.18(11)	135.64(6)	134.3(4)	136.91(8)
$\angle_{plane\cdots plane}$	X = I	X = I	X = I	X = Cl	X = Cl
**X–M–C···Cp_{cent}–M–Cp_{cent}**	91.75(4)	89.14(12)	91.10(6)	94.22(17)	87.46(6)

Note: Complexes **4Nd** and **4Am** have partial occupancy of both Cl and I halide present without significantly perturbing the rest of the structure whereas **2Nd** and **5Nd** feature pure I and Cl occupancy respectively as they were made from NdI_3 or $NdCl_3$. Furthermore, **5Nd** crystallized in $\bar{P}1$, and features 2 molecules in the asymmetric unit, whereas all others crystallized in $P2_1/c$ with a single molecule.

*Plots comparing M–C_{NHC} and M–I bond metrics for **1M**, **2M** and **4Am**.*

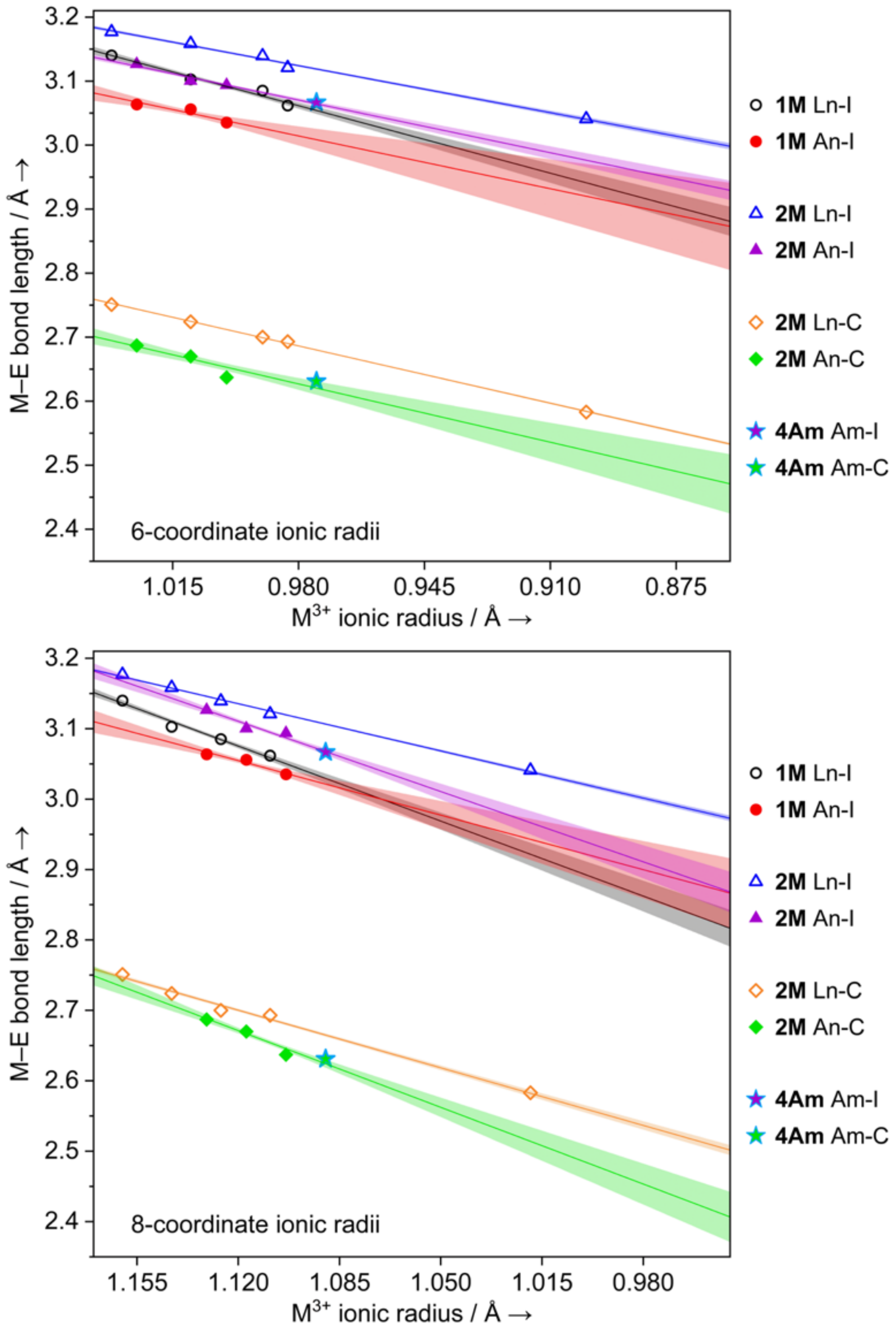

Figure S44. A plot of M^{3+} ionic radius (6-coordinate top, 8-coordinate bottom)[18] *vs* M–E bond length for M–I with both **1M** and **2M**, and M–C_{NHC} for **2M**. Open symbols are used for Ln complexes (La, Ce, Pr, Nd, Y from left to right), solid symbols are used for An complexes (U, Np, Pu, Am from left to right). Solid lines denote a linear fit of the data points, shaded areas show the 50% confidence interval for extrapolated data. For the **1M** series, only metal site (1) is shown here, which corresponds in all cases to the shorted M–I length.

The underlying reasons for the previous absence of clear Am^{3+}/Nd^{3+} distinction is a combination of the following factors: (i) the lack of Am/Nd complexes that are isomorphous or isostructural, and the general paucity of systematic studies from U to Am which would allow the Am data to be robustly interpreted in the context of other An/Ln metrical comparisons (indeed there are *ca*. 61 structures containing Am in the CCDC, *vs* >9,000 for U as of late 2023); (ii) historically there are relatively large esd values associated with Am–L/Nd–L bond distances, especially for complexes with low *Z* atoms bound to the metal center, which often precludes differences reaching significance at the 3σ level; (iii) donor types which do not invoke sufficient An/Ln bonding differences as the Lewis acidity of An^{3+} *vs* Ln^{3+} ions converges towards Am^{3+}/Nd^{3+}, and hence ionic bonding regimes are more likely to be encountered.

S5. NMR spectroscopy plots

NMR spectra of [Ln(Cp)$_2$(I)(THF)] (**1Ln**; Ln = La, Ce, Pr, Nd)*

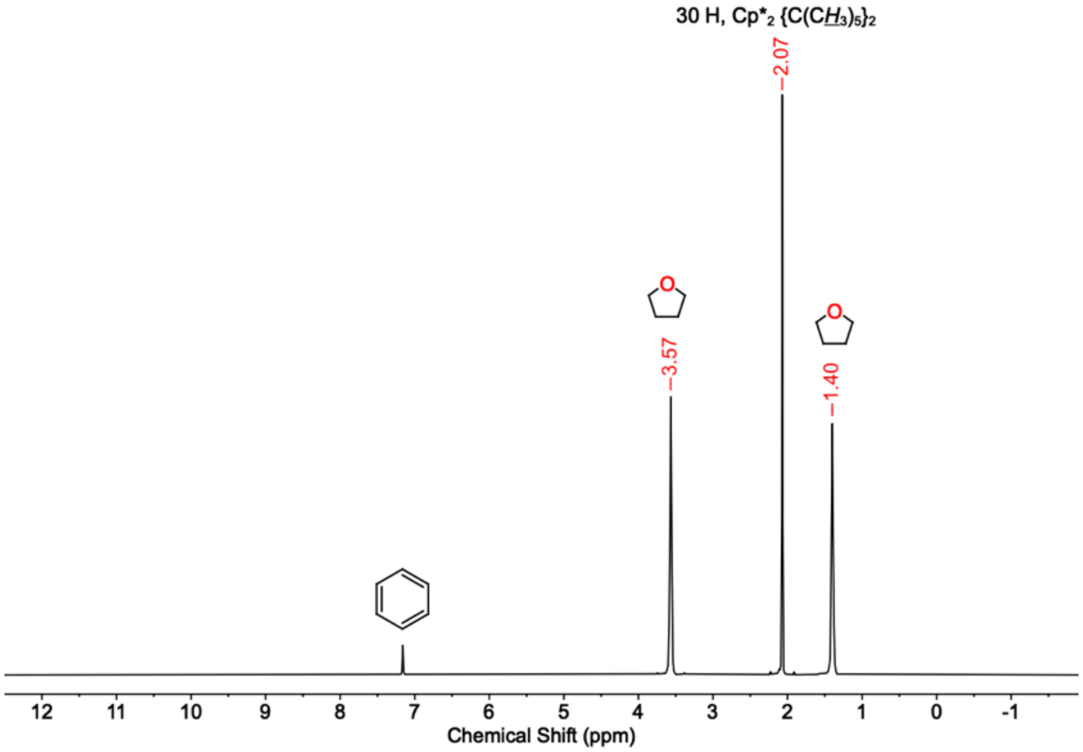

Figure S45. ^{1}H NMR spectrum of [La(Cp*)$_2$(I)(THF)] (**1La**) in D$_6$-benzene with 1 drop (12 mg) of [H]$_8$-THF. Collected at 296 K.

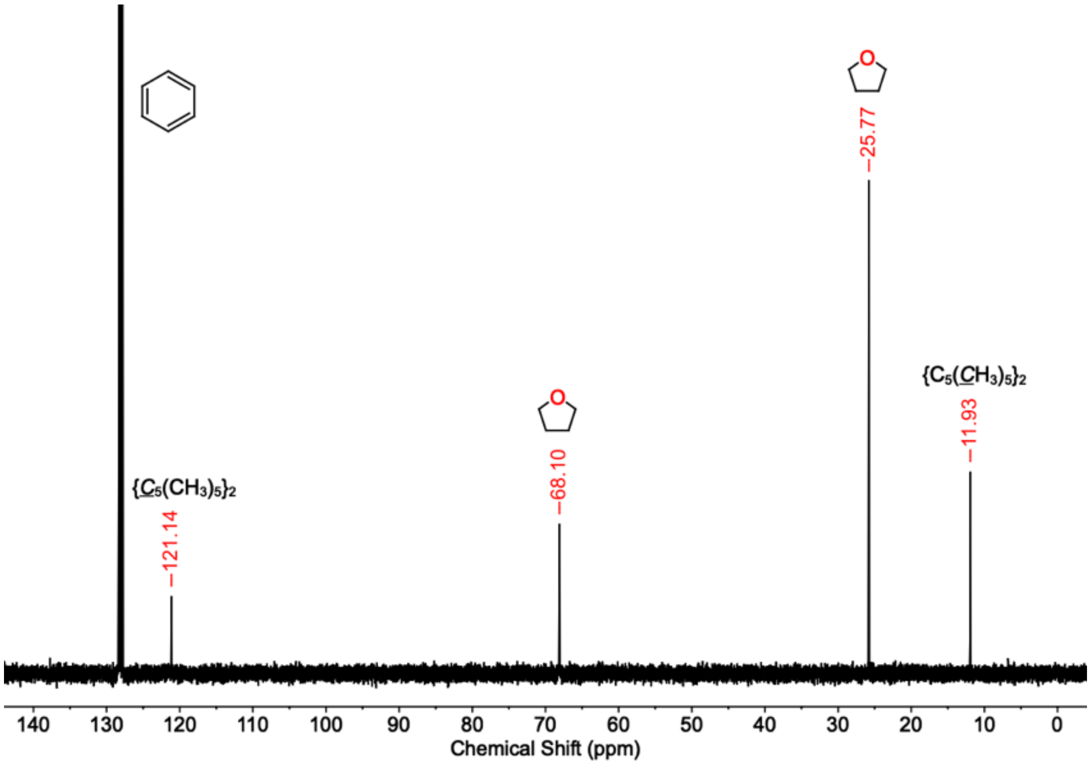

Figure S46. ^{13}C{^{1}H} NMR spectrum of [La(Cp*)$_2$(I)(THF)] (**1La**) in D$_6$-benzene with 1 drop (12 mg) of [H]$_8$-THF. Collected at 296 K.

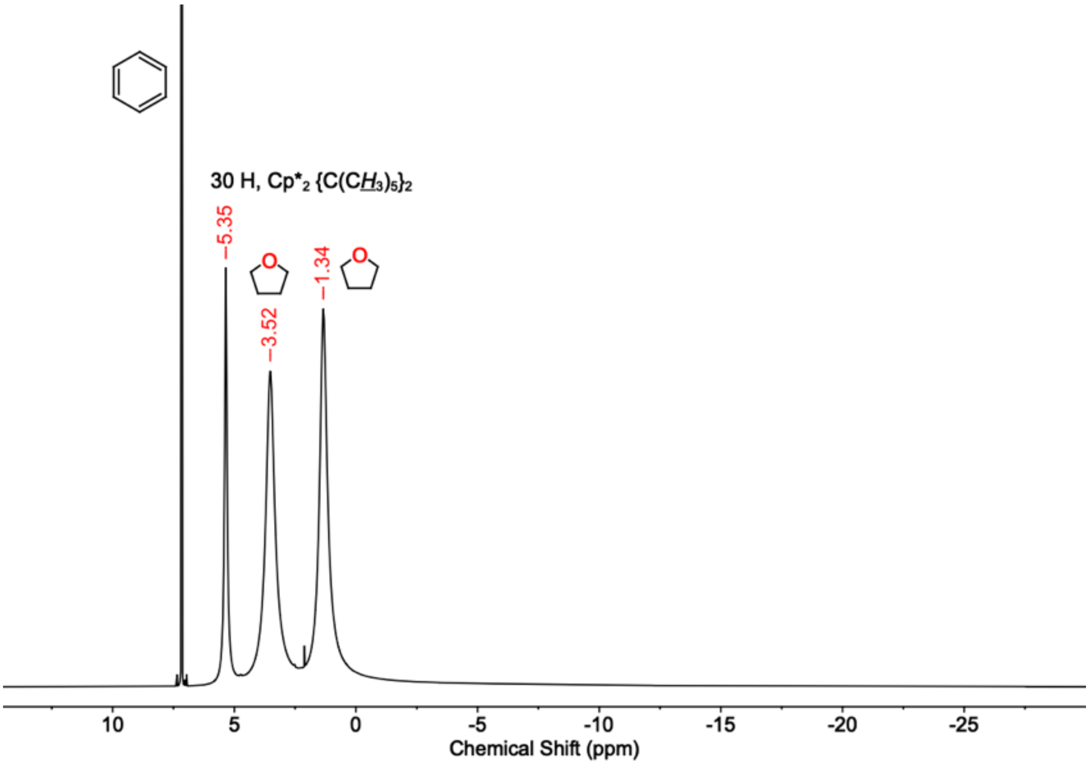

Figure S47. ^{1}H NMR spectrum of [Ce(Cp*)$_2$(I)(THF)] (**1Ce**) in D_6-benzene with 1 drop (12 mg) of [H]$_8$-THF. No other peaks with significant intensity were observed between ±150 ppm. Collected at 296 K.

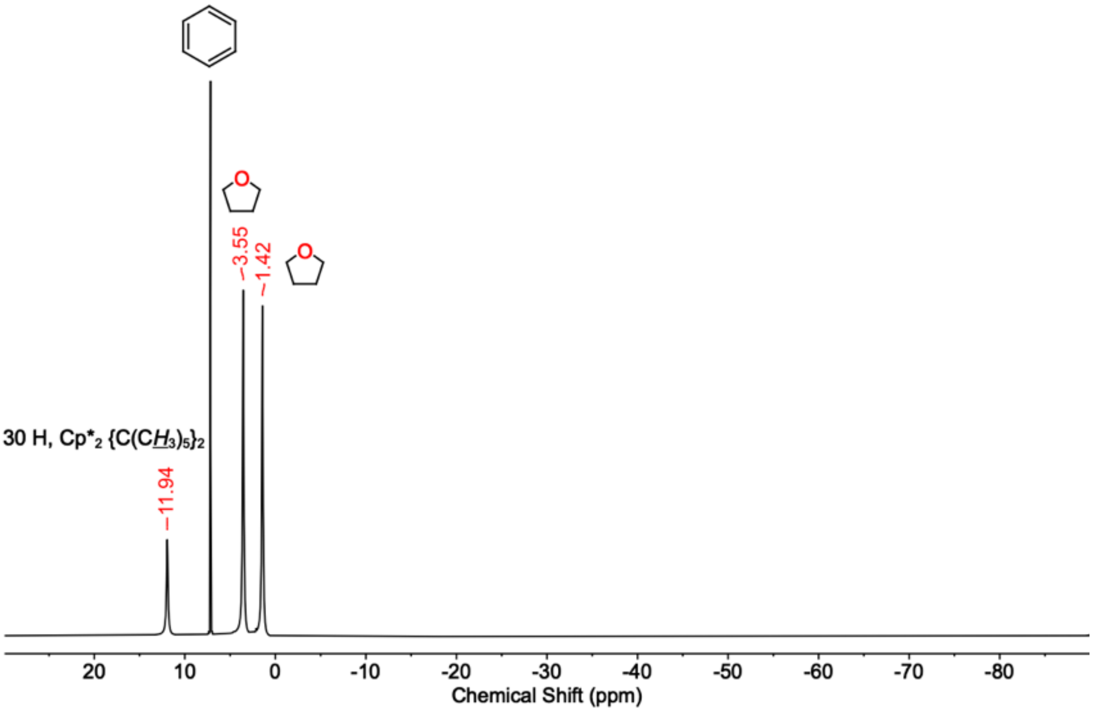

Figure S48. ^{1}H NMR spectrum of [Pr(Cp*)$_2$(I)(THF)] (**1Pr**) in D_6-benzene with 1 drop (12 mg) of [H]$_8$-THF. No other peaks with significant intensity were observed between ±150 ppm. Collected at 296 K.

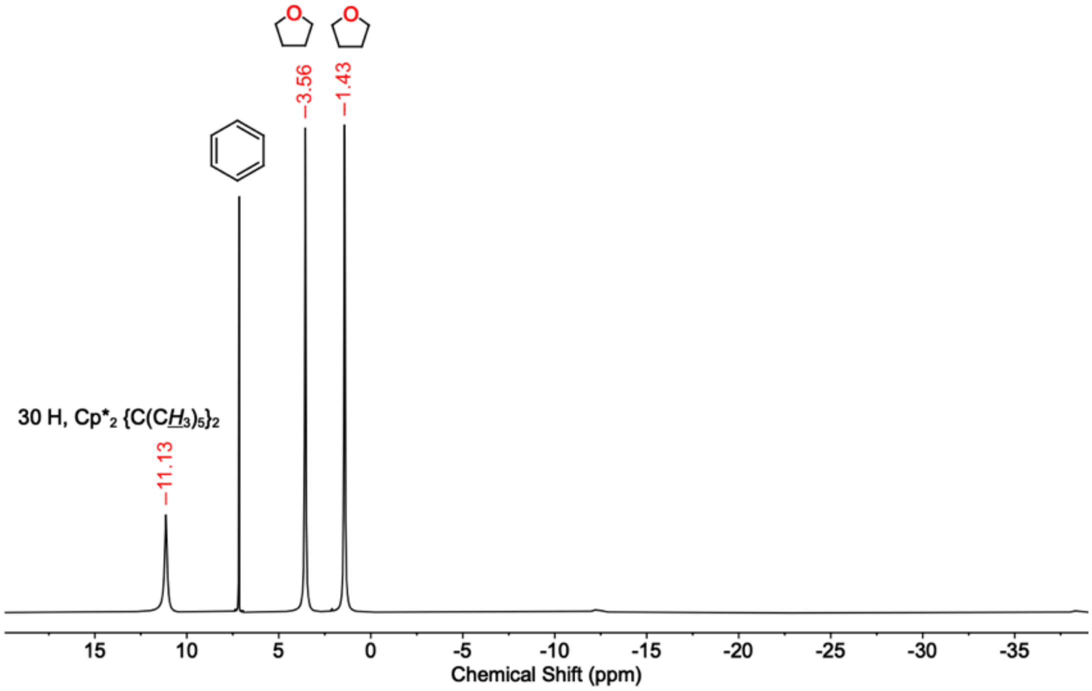

Figure S49. ^{1}H NMR spectrum of [Nd(Cp*)$_2$(I)(THF)] (**1Nd**) in D_6-benzene with 1 drop (12 mg) of [H]$_8$-THF. No other peaks with significant intensity were observed between ±150 ppm. Collected at 296 K.

NMR spectra of [Ln(Cp)$_2$(I)(I^{Me4})] (**2Ln**; Ln = La, Ce, Pr, Nd, Y)*

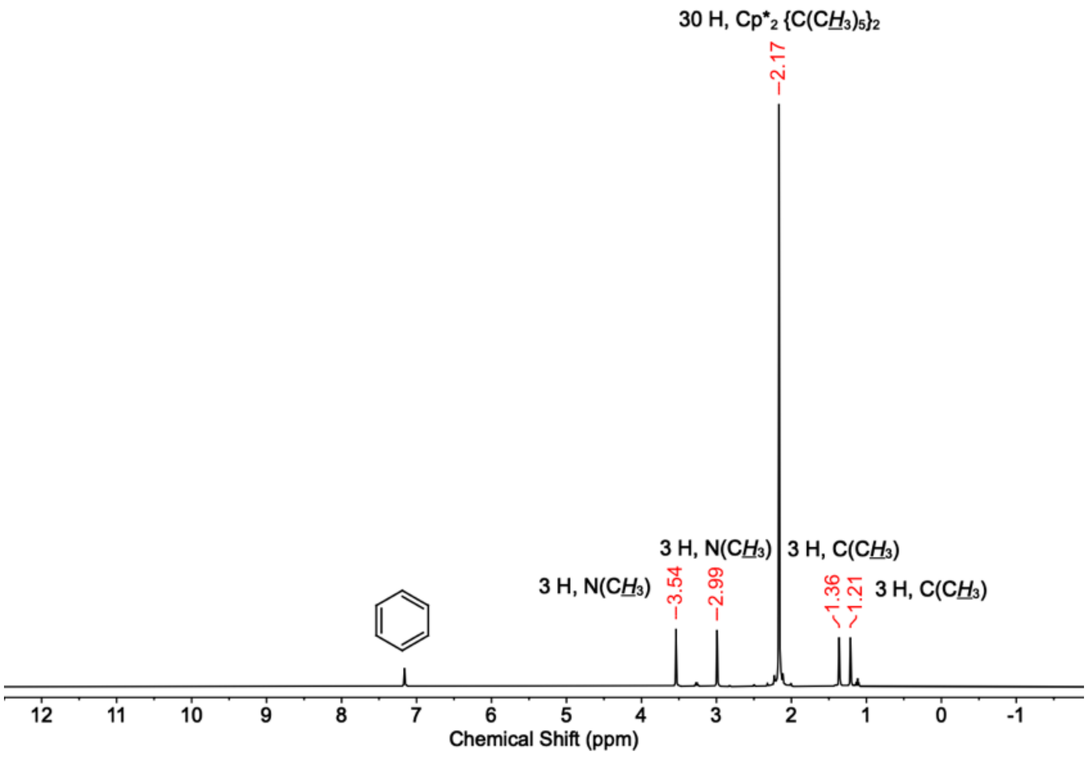

Figure S50. ^{1}H NMR spectrum of [La(Cp*)$_2$(I)(I^{Me4})] (**2La**) in D$_6$-benzene. Collected at 298 K.

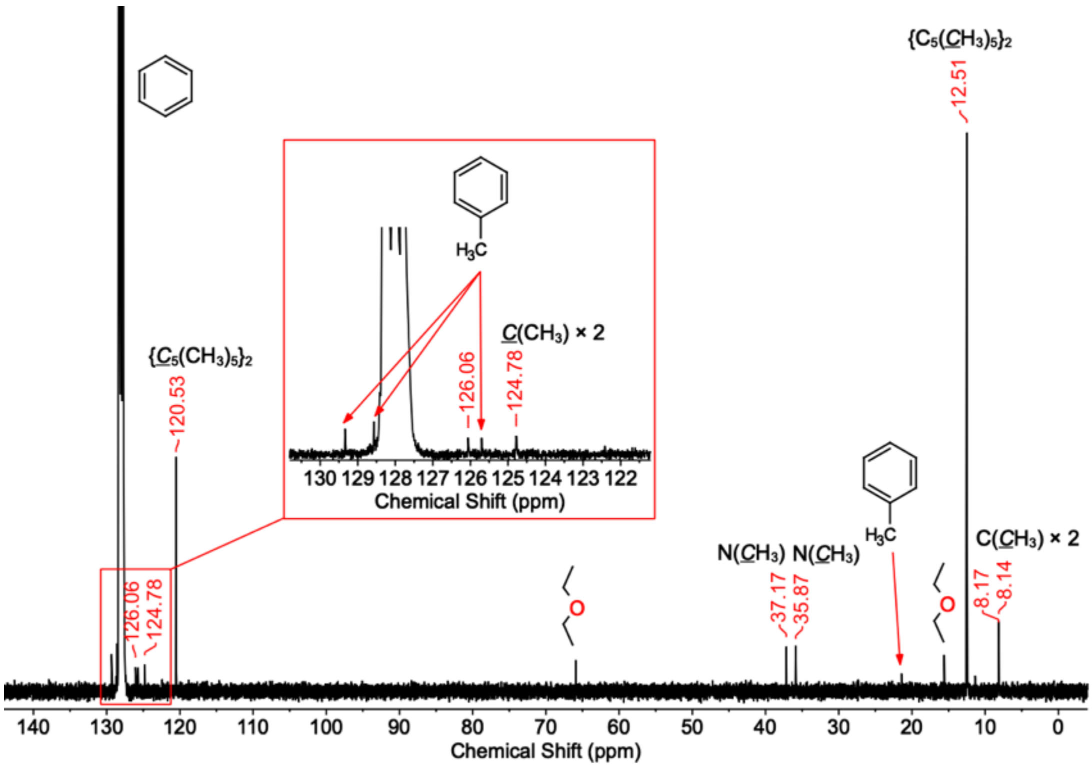

Figure S51. ^{13}C{^{1}H} NMR spectrum of [La(Cp*)$_2$(I)(I^{Me4})] (**2La**) in D$_6$-benzene. Scans up to +650 ppm could not locate the carbenic carbon atom of the I^{Me4} moiety presumably due to quadrupolar broadening from the >99.9% abundant ^{139}La (I = 7/2). Collected at 298 K.

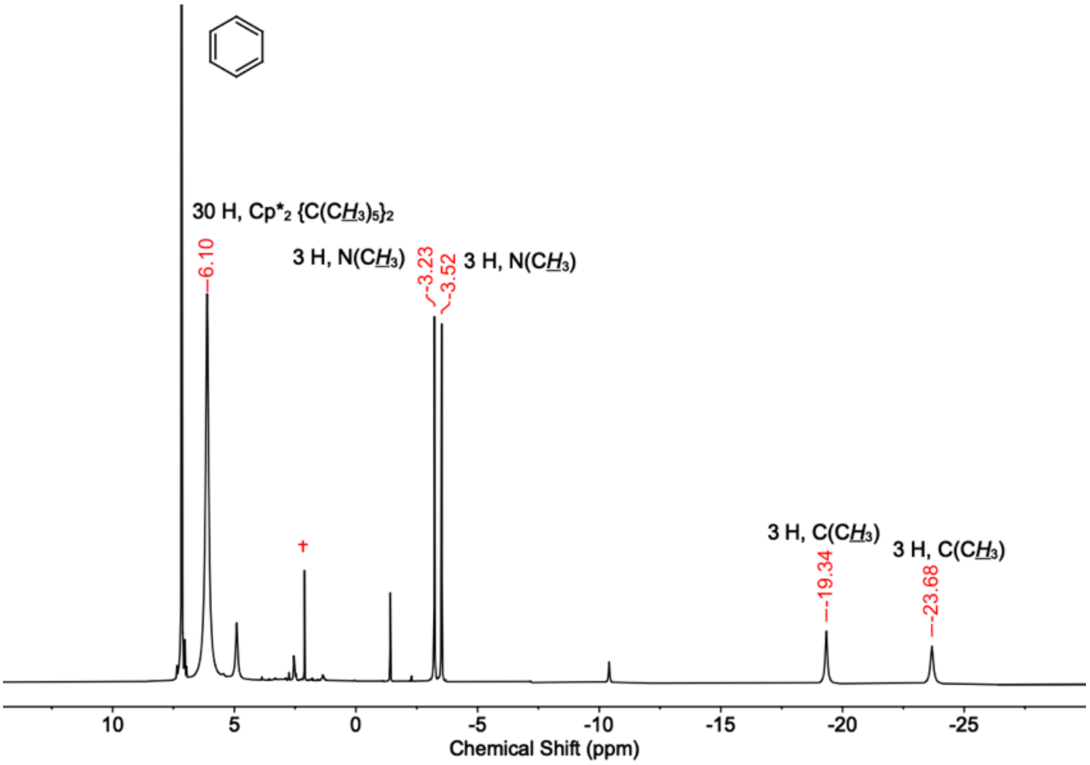

Figure S52. ^{1}H NMR spectrum of $[Ce(Cp^*)_2(I)(I^{Me4})]$ (**2Ce**) in D_6-benzene. No other peaks with significant intensity were observed between ±150 ppm. † denotes toluene C*H*3. We have not been able to conclusively identify any impurity peaks. Collected at 296 K.

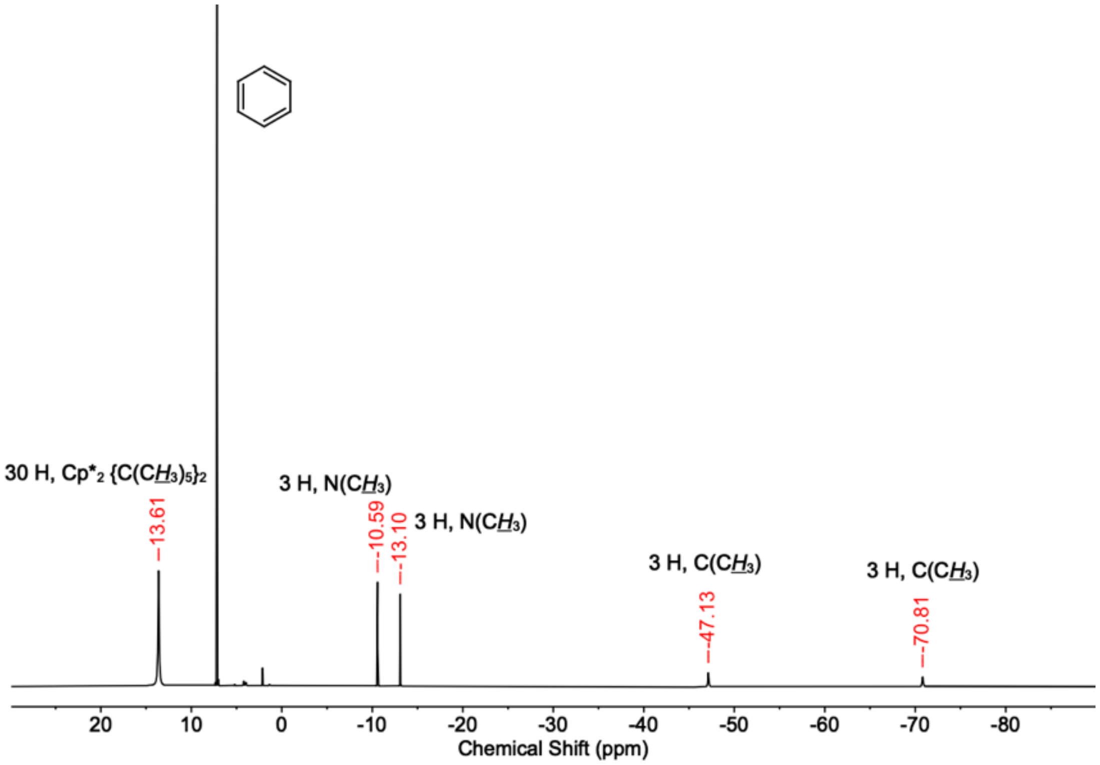

Figure S53. ^{1}H NMR spectrum of $[Pr(Cp^*)_2(I)(I^{Me4})]$ (**2Pr**) in D_6-benzene. No other peaks with significant intensity were observed between ±150 ppm. Collected at 296 K.

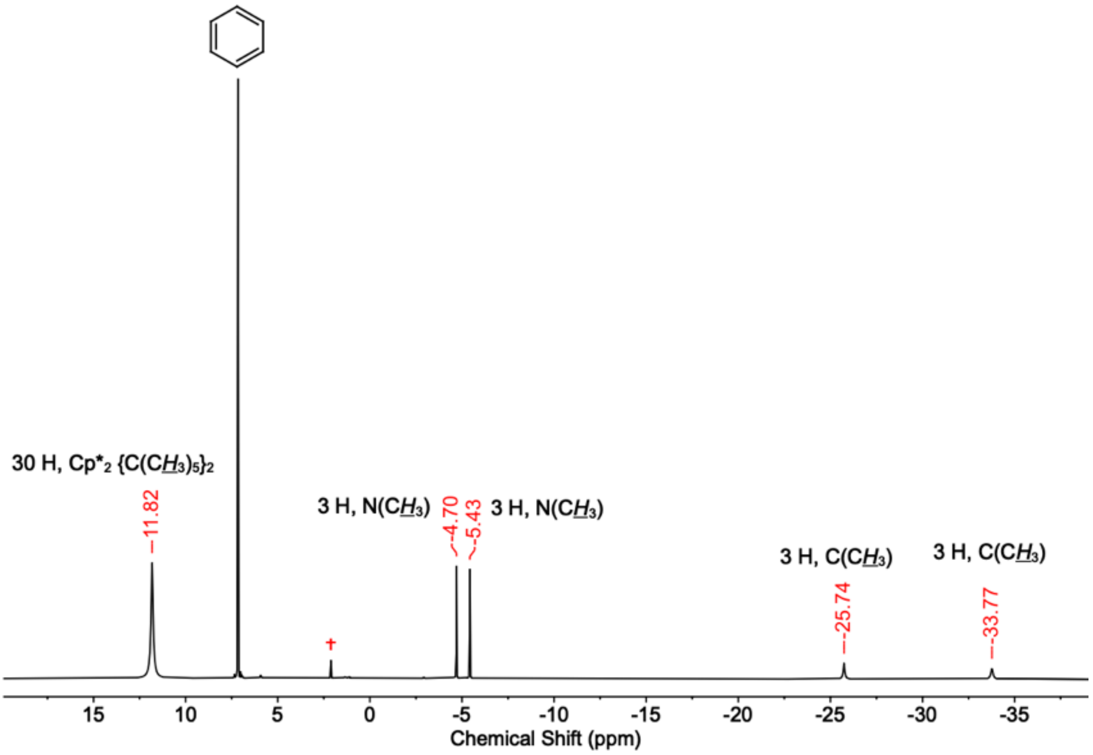

Figure S54. ^{1}H NMR spectrum of [Nd(Cp*)$_2$(I)(I^{Me4})] (**2Nd**) in D$_6$-benzene. No other peaks with significant intensity were observed between ±150 ppm. † denotes toluene C*H*$_3$. We have not been able to conclusively identify any impurity peaks. Collected at 298 K.

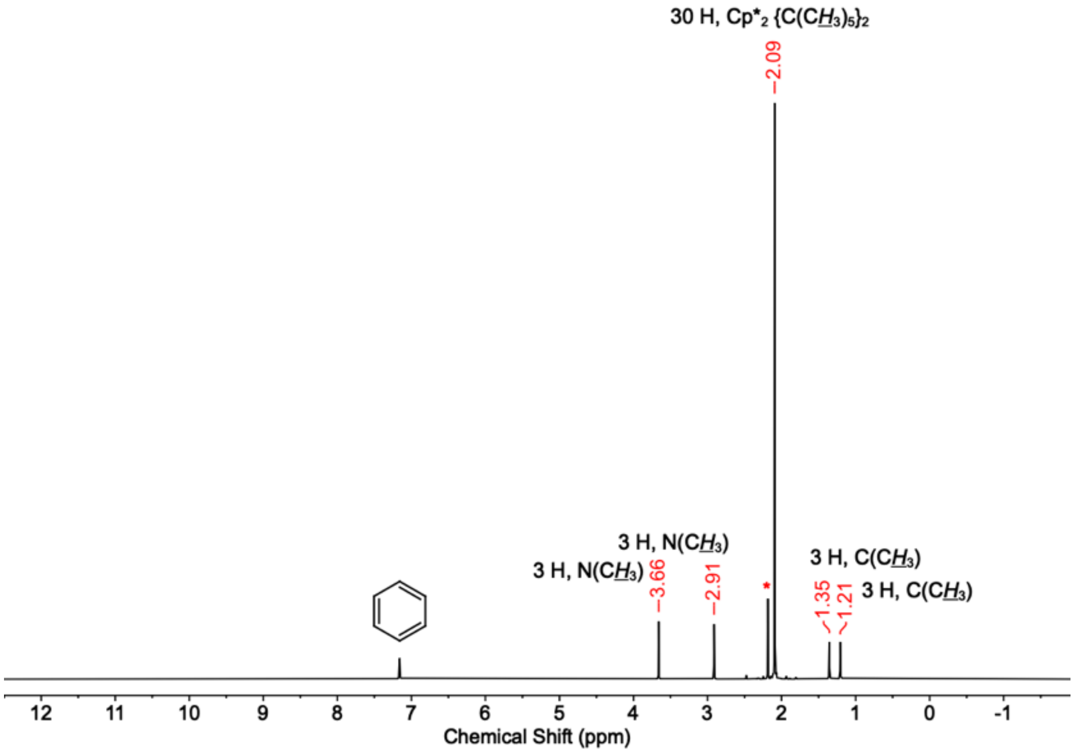

Figure S55. ^{1}H NMR spectrum of [Y(Cp*)$_2$(I)(I^{Me4})] (**2Y**) in D$_6$-benzene. * denotes an unidentified impurity. Collected at 296 K.

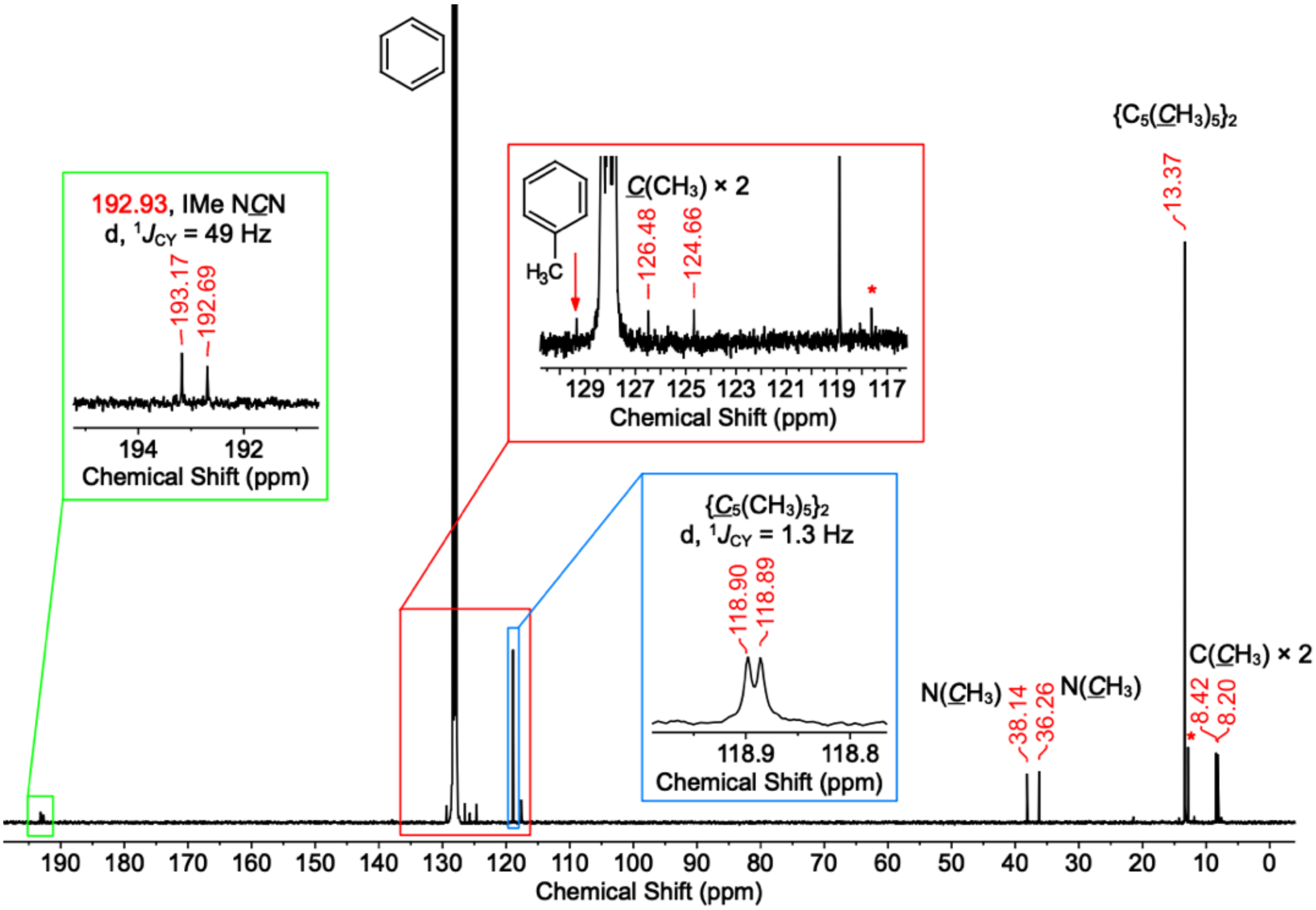

Figure S56. $^{13}C\{^{1}H\}$ NMR spectrum of $[Y(Cp^*)_2(I)(I^{Me4})]$ (**2Y**) in D_6-benzene. * denotes an unidentified impurity. Collected at 296 K.

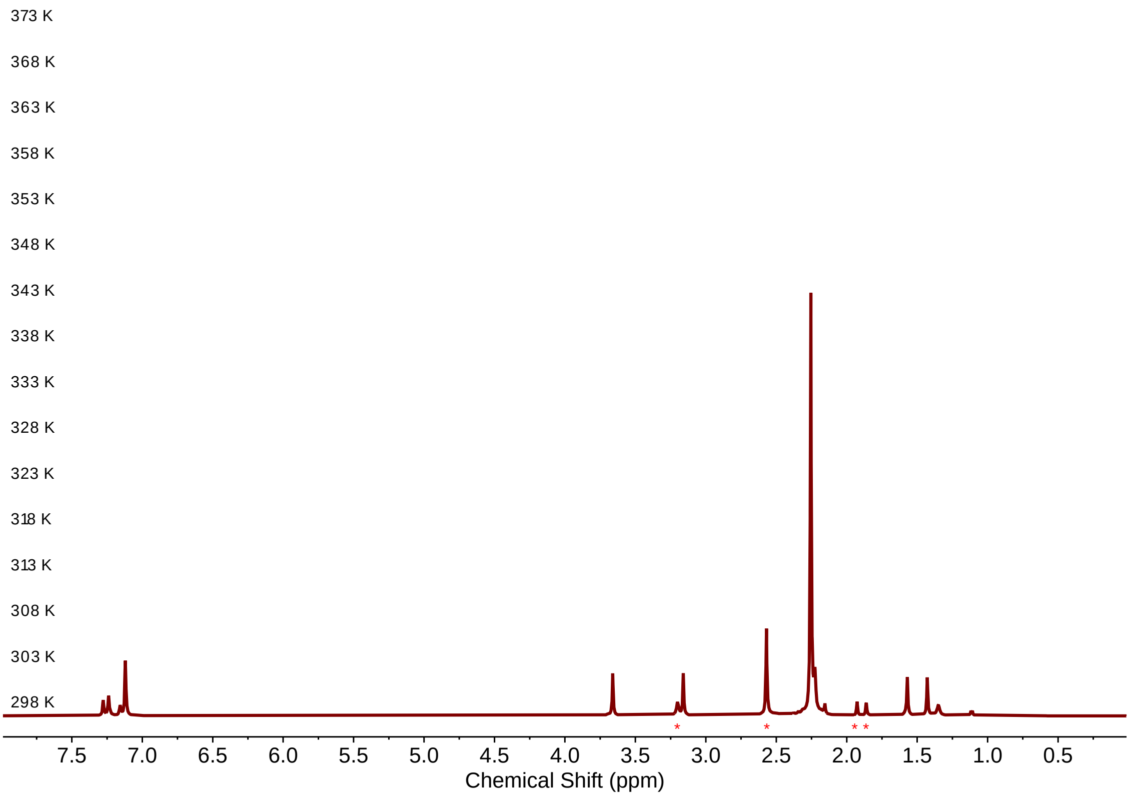

Figure S57. Variable-temperature ^{1}H NMR spectra of [La(Cp*)$_2$(I)(I^{Me4})] (**2La**) in D$_8$-toluene with several drops of D$_6$-benzene. * denotes an unknown impurity.

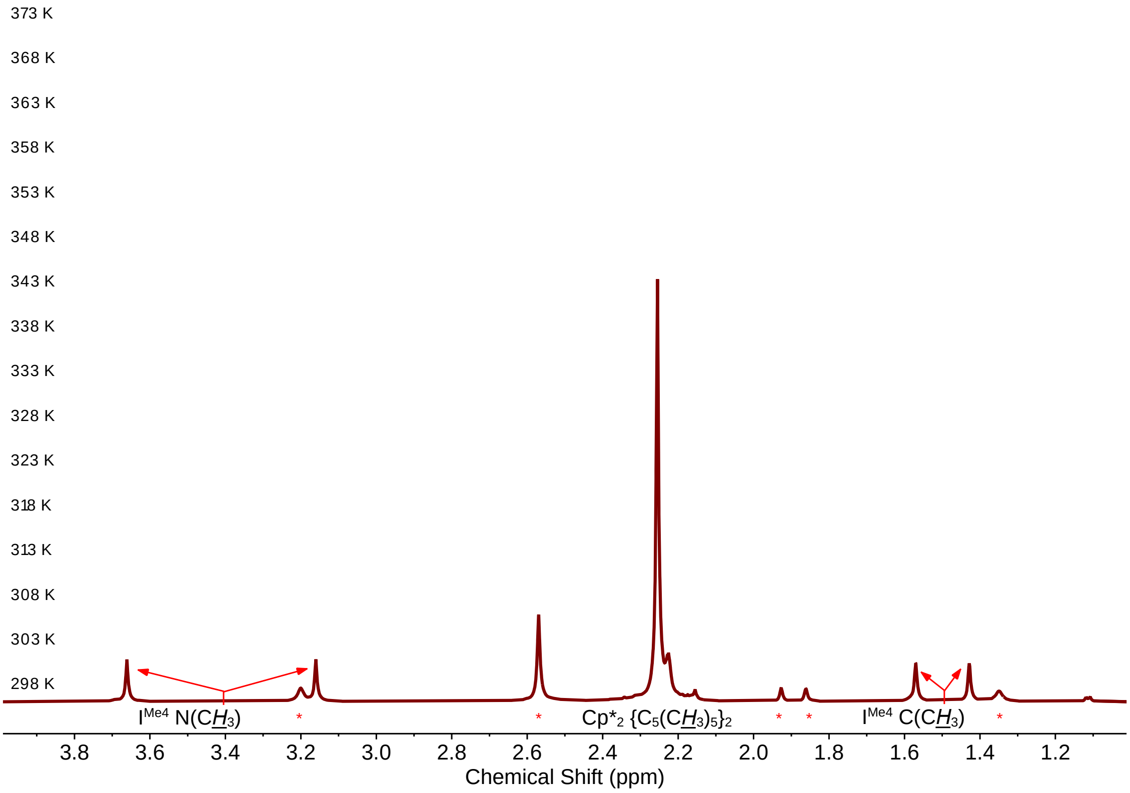

Figure S58. Variable-temperature ^{1}H NMR spectra of [La(Cp*)$_2$(I)(I^{Me4})] (**2La**) in D$_8$-toluene with several drops of D$_6$-benzene. Showing the region associated with the I^{Me4} N(CH$_3$) and I^{Me4} C(CH$_3$) peaks. * denotes an unknown impurity.

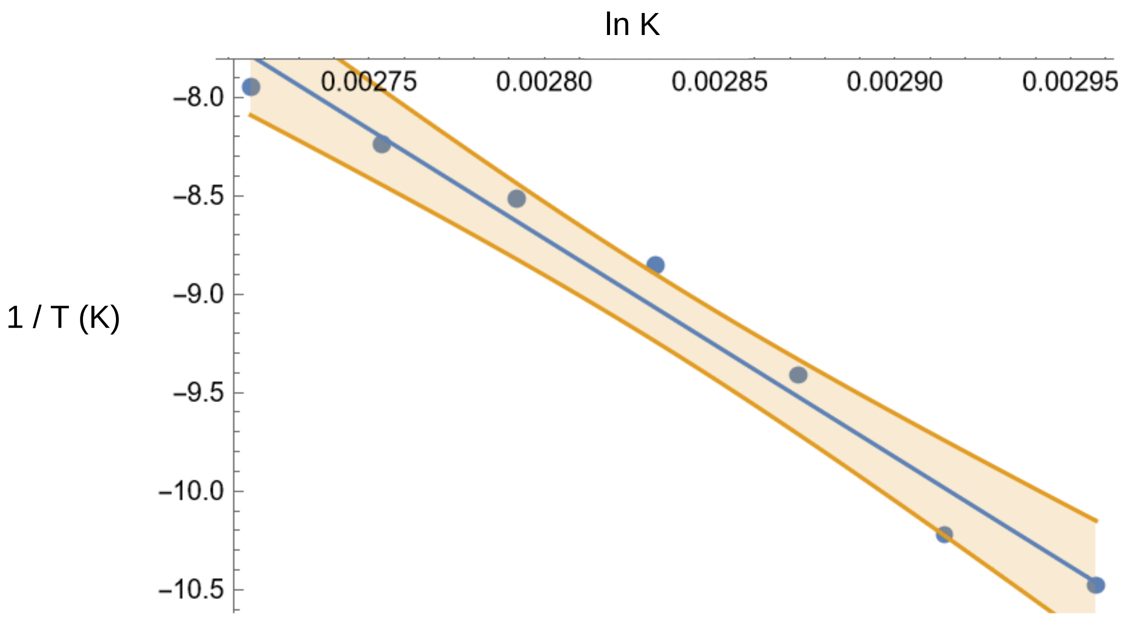

Figure S59. An Eyring plot using data from 338 K to 373 K. Data points are shown in blue circles, the line of best fit is indicated by the blue line, and the orange shaded area denotes the 95% confidence intervals. $\Delta S^{\ddagger}$ = –11.6 J·K^{-1}mol^{-1} (–61.8 J·K^{-1}mol^{-1} to 38.5 J·K^{-1}mol^{-1}) and $\Delta H^{\ddagger}$ = 92.3 kJ·mol^{-1} (74.6 kJ·mol^{-1} to 110.0 kJ·mol^{-1}) – the 95% confidence ranges are shown in the brackets.

^{1}H NMR measurements were performed on **2La** over the temperature range 298 – 373 K, at 700 MHz nominal Larmor frequency, to collect temperature-dependent ^{1}H spectra. The region (1.3 – 1.7 ppm) of the NMR spectrum that showed significant exchange characteristics was selected and the signals from that region were used for bandshape analysis.

Coalescence of the exchanging signals was not achieved at the highest temperature accessible. Bandshape analysis was performed on the real part of the selected region of each NMR spectrum which was converted to ascii format using the Topspin software package (Bruker Biospin Corporation, Version 3.6.1, Billerica, 2018). A non-linear least squares fit of the data to the Bloch equations modified for 2-site exchange,[26] with an additional Lorentzian to consider the known impurity, was performed to determine the rate of exchange at each temperature. Iterative fitting was completed using Wolfram Mathematica 13.0. The determined rates were used to calculate $\Delta H^{\ddagger}$ and $\Delta S^{\ddagger}$ using an Eyring plot, again fit using a non-linear least squares fitting algorithm in Mathematica.

NMR spectra of [An(Cp)$_2$(I)(I^{Me4})] (**2An**; An = Np, Pu), and [Am(Cp*)$_2$(I$_x$Cl$_{1-x}$)(I^{Me4})] (**4Am**)*

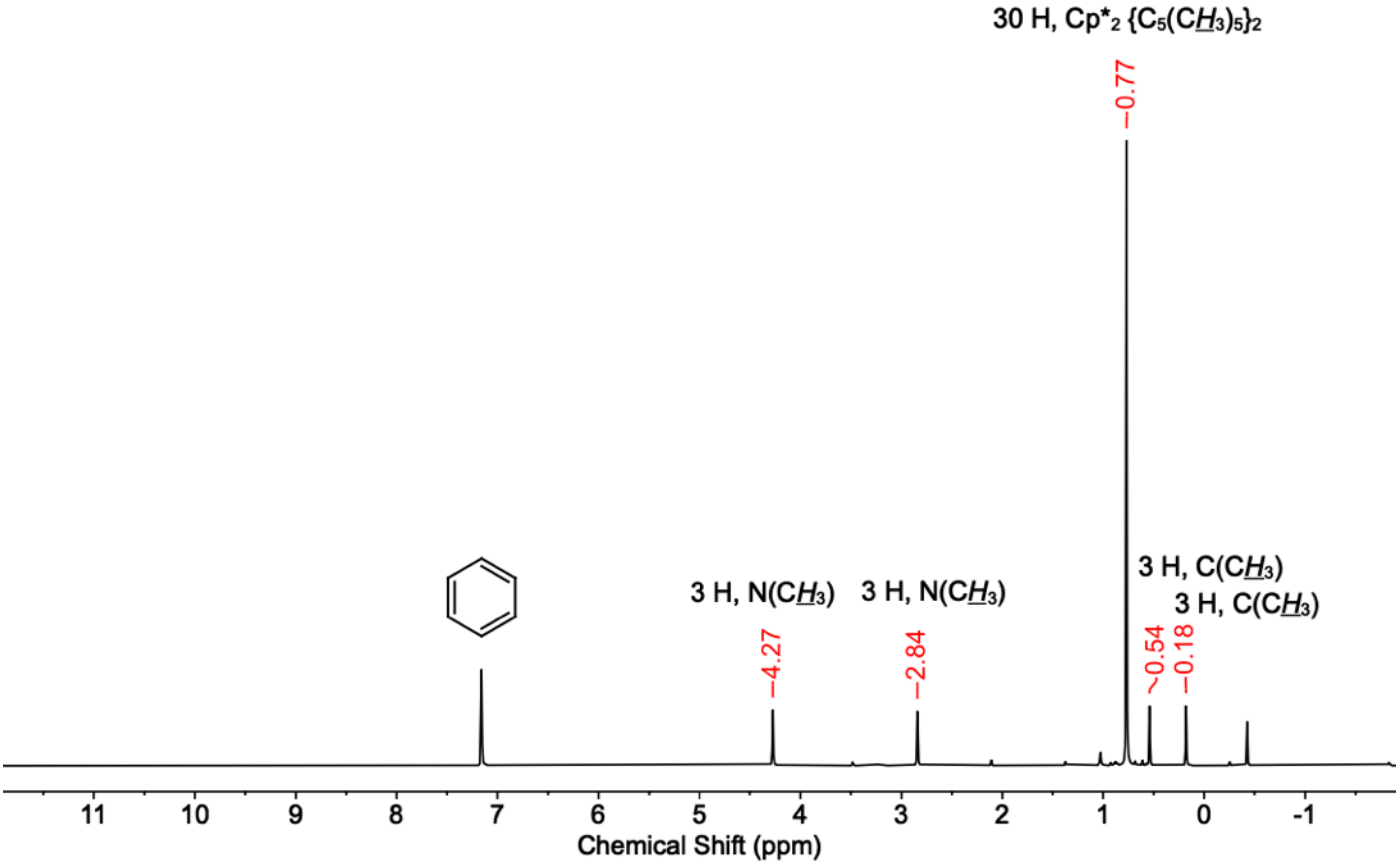

Figure S60. ^{1}H NMR spectrum of [Np(Cp*)$_2$(I)(I^{Me4})] (**2Np**) in D$_6$-benzene. The peak at –0.42 ppm integrates to 2, whereas all others integrate correctly. We suspect this peak is an unidentified impurity as no other peaks with significant intensity were observed between ±150 ppm. Collected at 298 K.

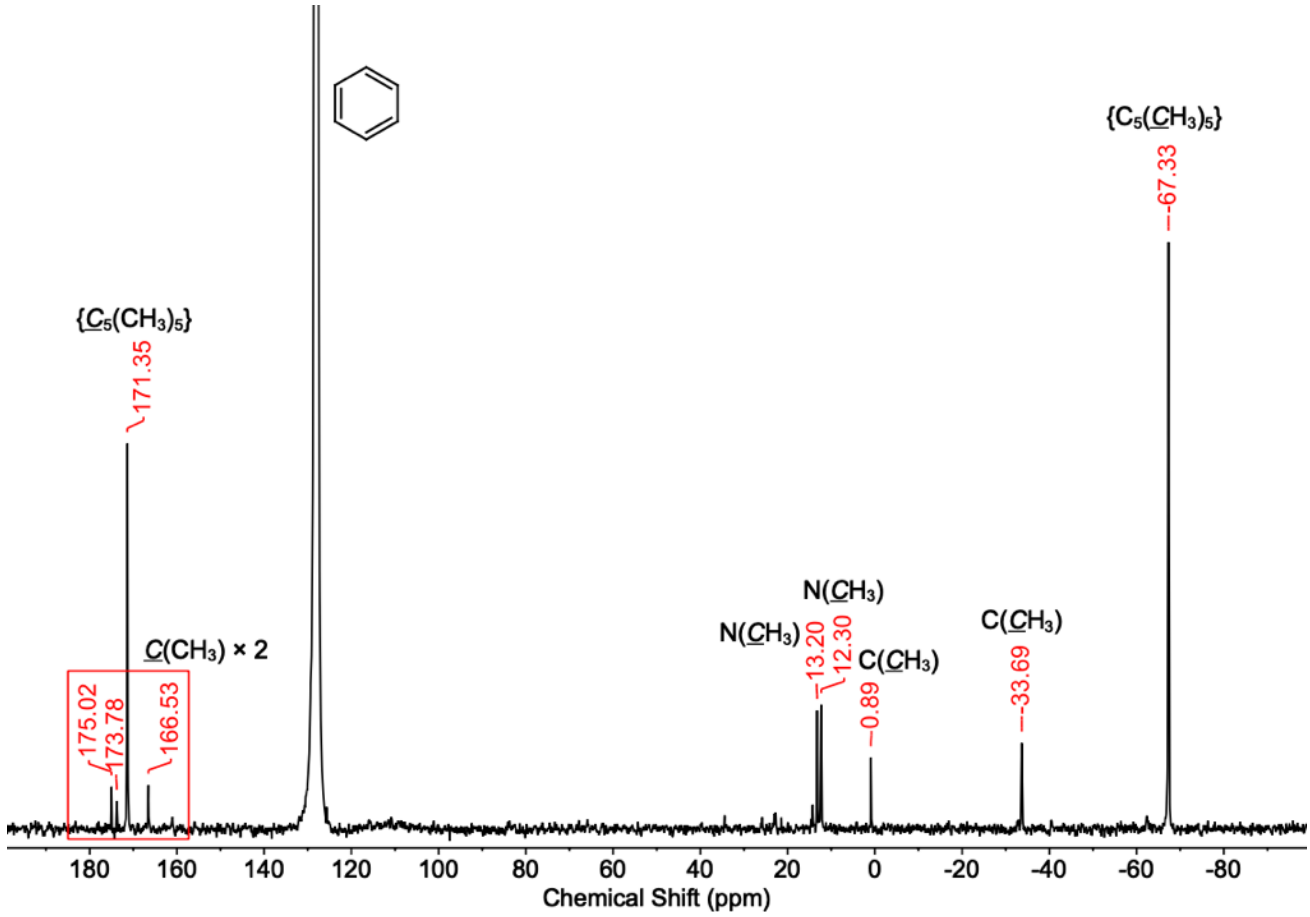

Figure S61. $^{13}C\{^1H\}$ NMR spectrum of [Np(Cp*)$_2$(I)(I^{Me4})] (**2Np**) in D$_6$-benzene. No other peaks with significant intensity were observed between ±500 ppm. Complex **2Np** should display nine resonances as all Me groups, and thus the two C(CH$_3$) are also inequivalent. We have observed nine peaks, shown above, however we cannot conclusively determine which two of the three peaks within the red box correspond to C(CH$_3$) resonances, and thus by process of elimination state which might be the carbene resonance or if indeed one of these peaks is simply an impurity. Sample longevity precluded a more detailed study by HSQC NMR. Collected at 298 K.

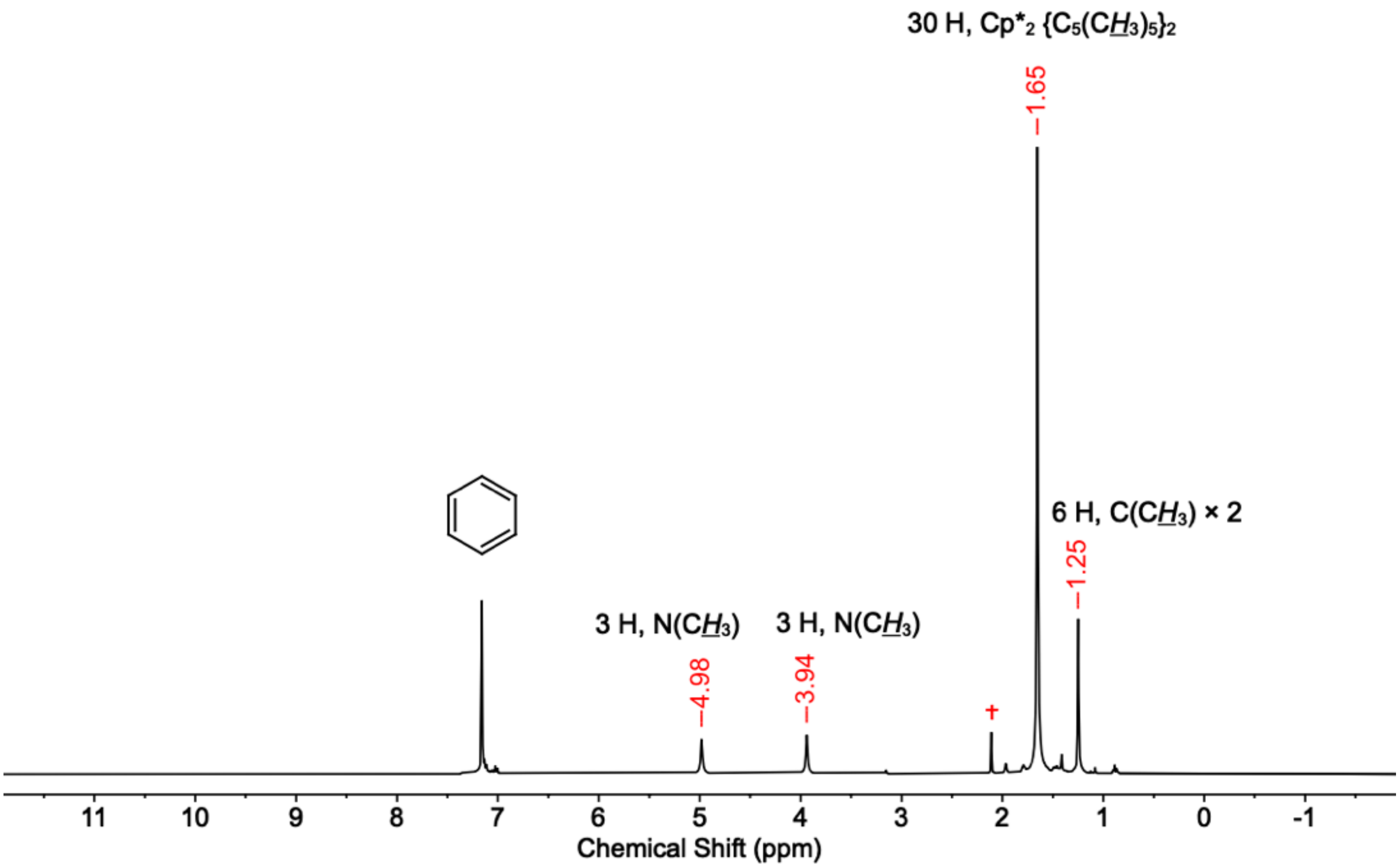

Figure S62. ^{1}H NMR spectrum of $[Pu(Cp^*)_2(I)(I^{Me4})]$ (**2Pu**) in D_6-benzene. No other peaks with significant intensity were observed between ±150 ppm. † denotes toluene C<u>*H*</u>$_3$. Note that while the two N(C<u>*H*</u>$_3$) peaks are inequivalent, the two C(C<u>*H*</u>$_3$) peaks overlap. Collected at 297 K.

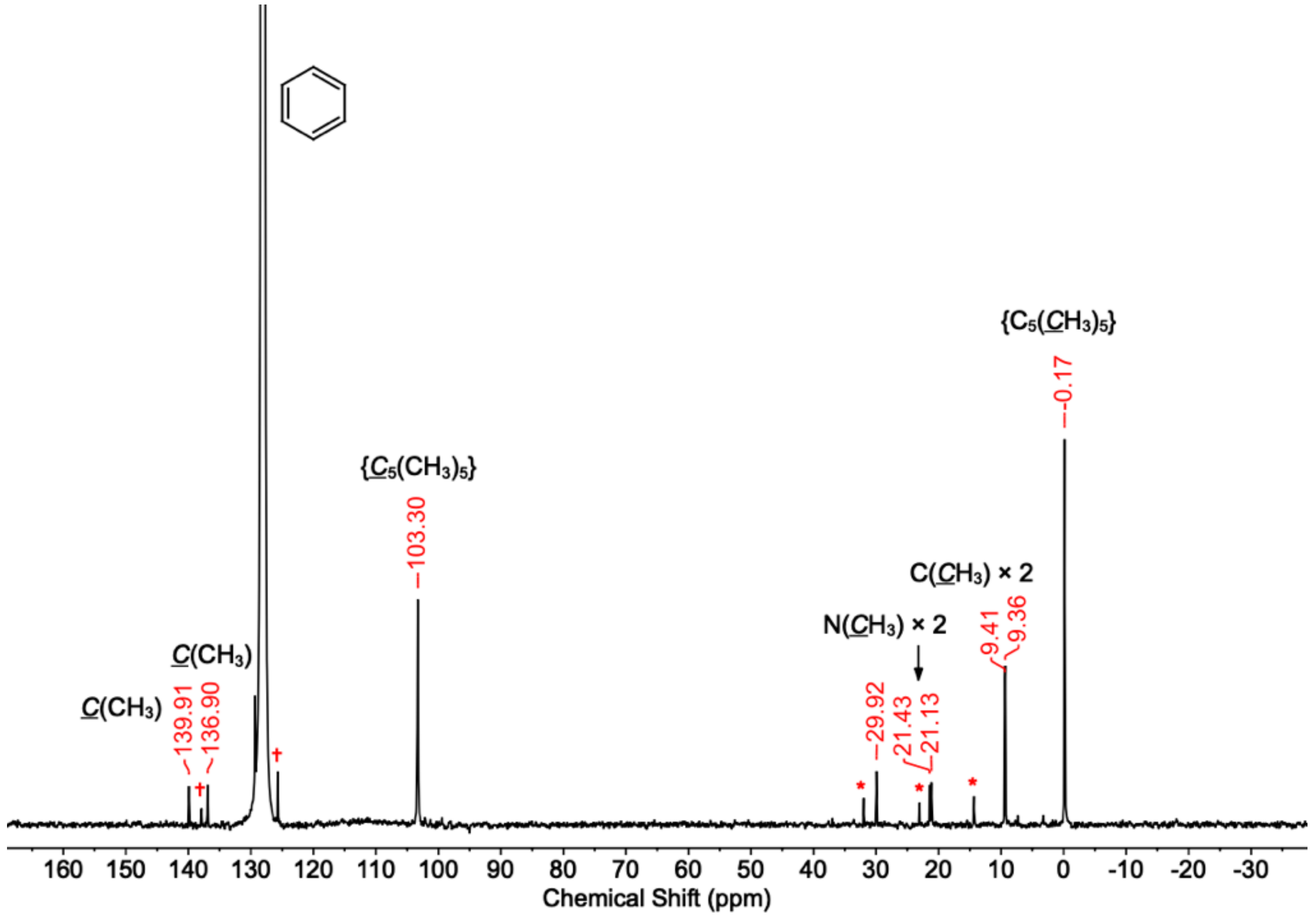

Figure S63. $^{13}C\{^1H\}$ NMR spectrum of [Pu(Cp*)$_2$(I)(I^{Me4})] (**2Pu**) in D$_6$-benzene. No other peaks with significant intensity were observed between ±500 ppm. † denotes toluene peaks, * denotes hexane peaks. Complex **2Pu** should display nine resonances as all Me groups, and thus the two C(CH$_3$), are potentially inequivalent. Note that while these overlapped in the ^1H spectrum, here the resonances at 9.36 / 9.41 ppm and 21.13 / 21.43 ppm show that these groups are magnetically inequivalent. Collected at 297 K.

We have observed nine potential peaks that could be from **2Pu**, or an impurity, shown above, however the peak 29.92 ppm does not seem a likely chemical shift for the carbenic carbon. It does not align with any known or suspected impurities in this crystalline sample.

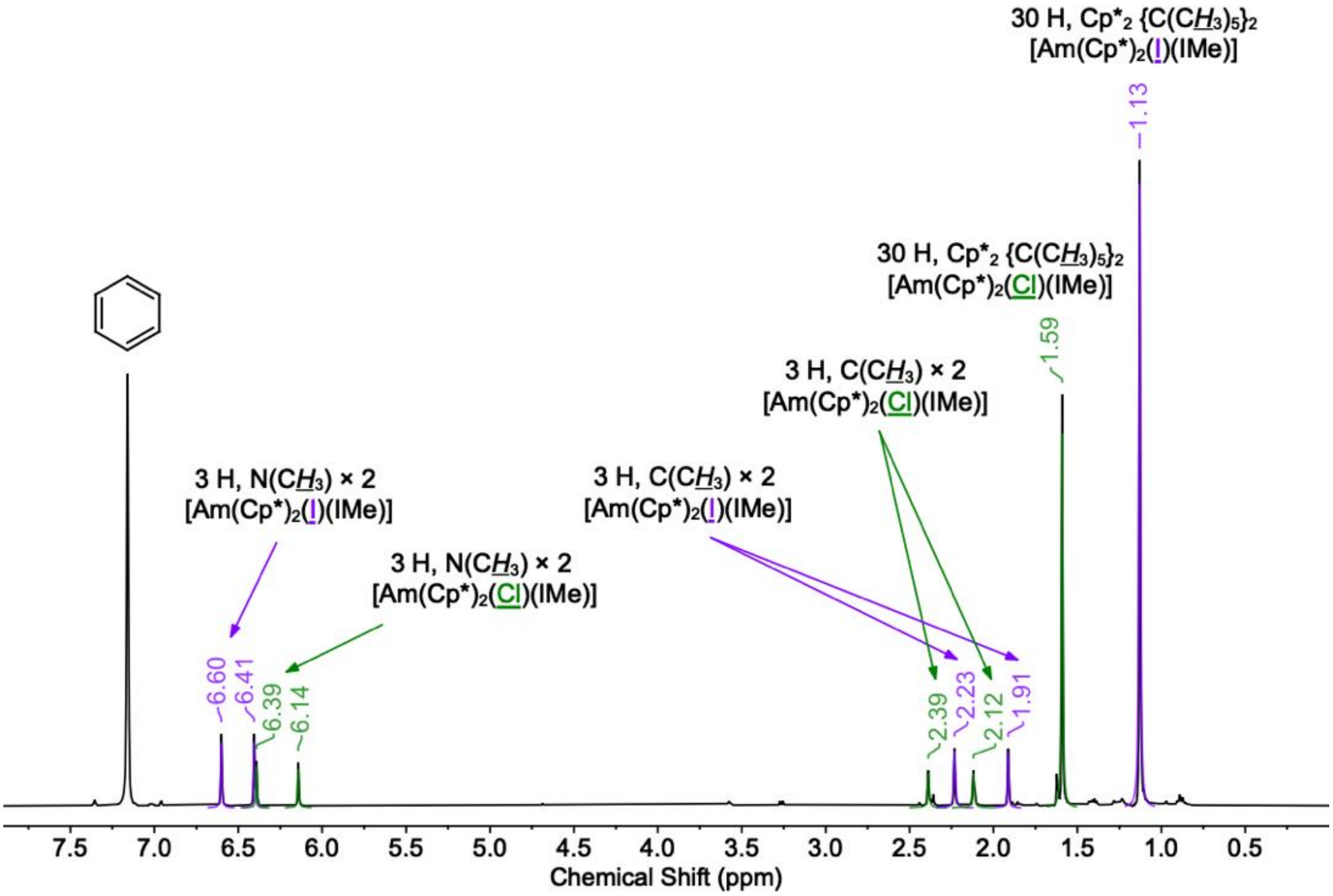

Figure S64. ^{1}H NMR spectrum of $[Am(Cp^*)_2(I_xCl_{1-x})(I^{Me4})]$ (**4Am**) in D_6-benzene. No other peaks with significant intensity were observed between ±150 ppm. Collected at 295 K.

This spectrum shows two species, both of which integrated well, independently, to '$(Cp^*)_2(I^{Me4})$' with the typically-observed 4 inequivelant I^{Me4} resonances. The two species integrate to approximately 0.6:0.40 (*vide infra*, **Figure S65**) using the Cp* C$\underline{H}_3$ peaks, and it prompted us to re-evaluate an initial refinement of our X-ray diffraction data where only an iodide had been assigned. Competetive refinement of a chloride *vs* an iodide (with unrestricted Am–X bond lengths) led to a ratio of approximately 0.65:0.35 across three independent data collections from three crystals.

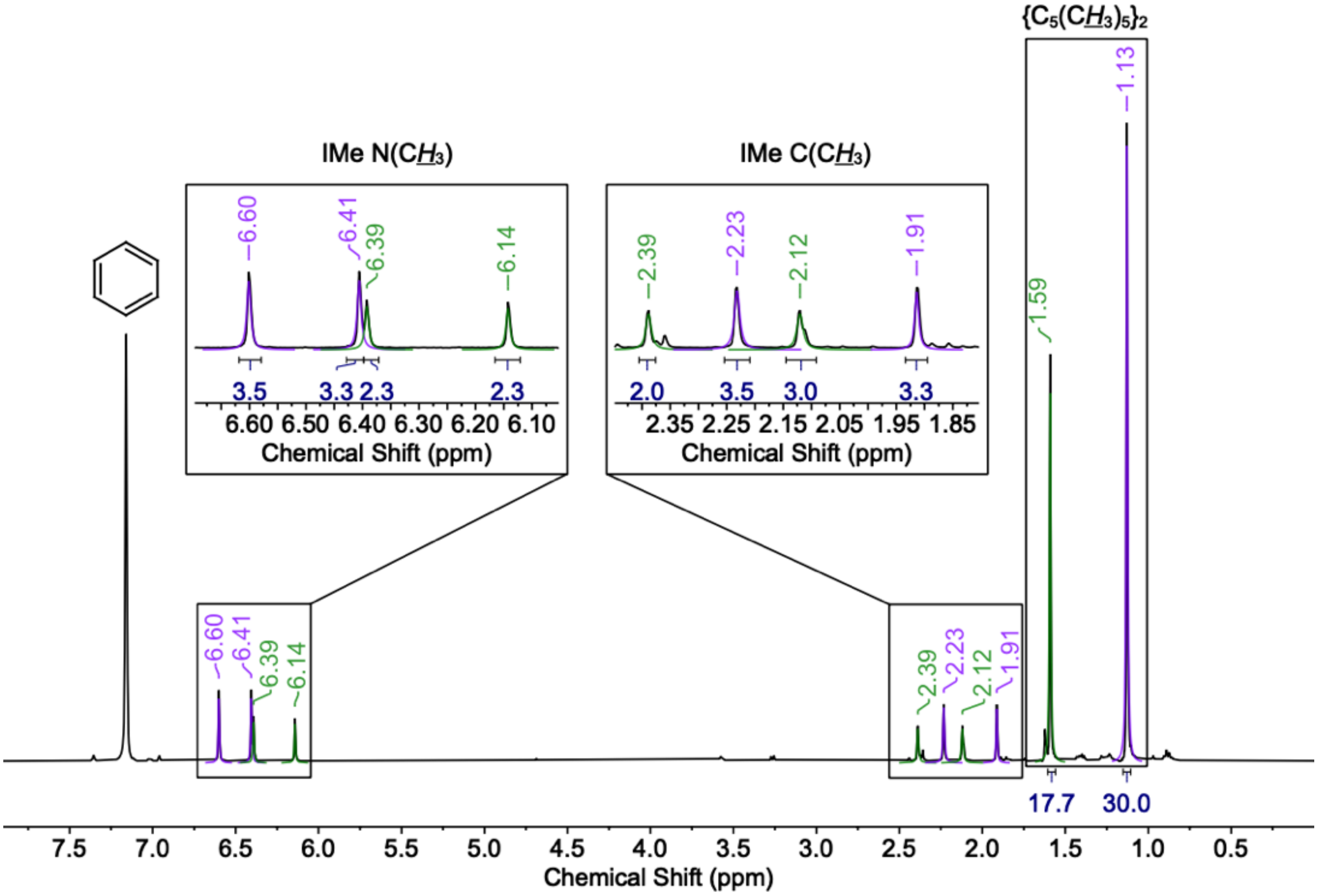

Figure S65. ^{1}H NMR spectrum of $[Am(Cp^*)_2(I_xCl_{1-x})(I^{Me4})]$ (**4Am**) in D_6-benzene. No other peaks with significant intensity were observed between ±150 ppm. Intended to show the relative integrals of the two species. Collected at 295 K.

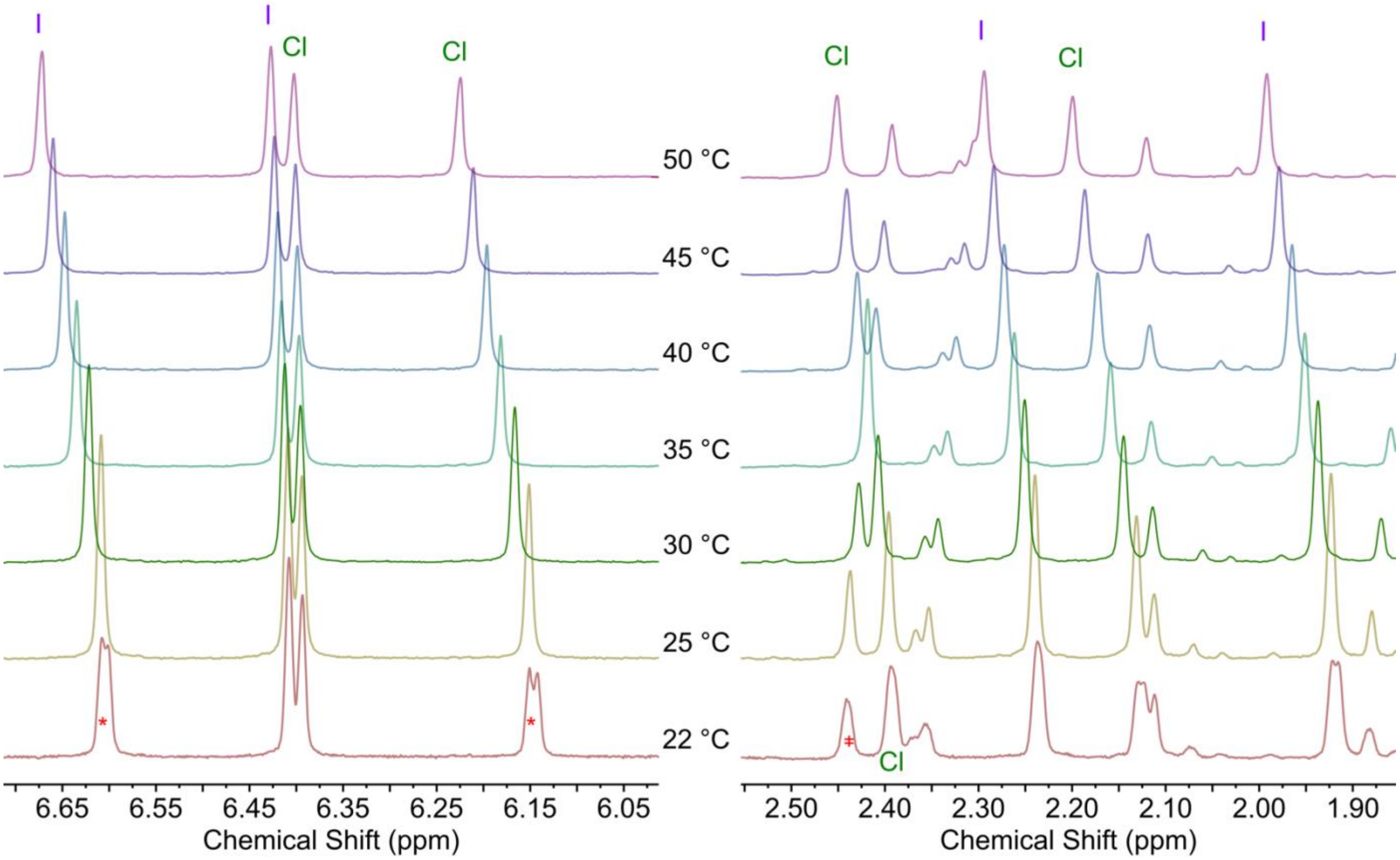

Figure S66. Variable-temperature ^{1}H NMR spectra of $[Am(Cp^*)_2(I_xCl_{1-x})(I^{Me4})]$ (**4Am**) in D_6-benzene. * denotes the apparent doubling of peaks at 22 °C which is an artefact, and ‡ denotes an unknown impurity which has become much more prominent in the time passed between the initial ^{1}H NMR study (**Figure S65**), and the VT NMR study here. The peak at 2.44 can be seen in **Figure S65** above, but is significantly smaller. We cannot identify this impurity.

We initially wondered if the two apparent species might have been due to steric-blocking of rotation about the Am–Cp* bond, resulting in two different chiral forms of the molecule. However (1) variable temperature ^{1}H NMR showed the two species to shift independently with temperature; (2) the subsequent synthesis and ^{1}H NMR spectrum of **2Y** showed that even a significantly smaller metal did not suffer from hindered rotation at room temperature.

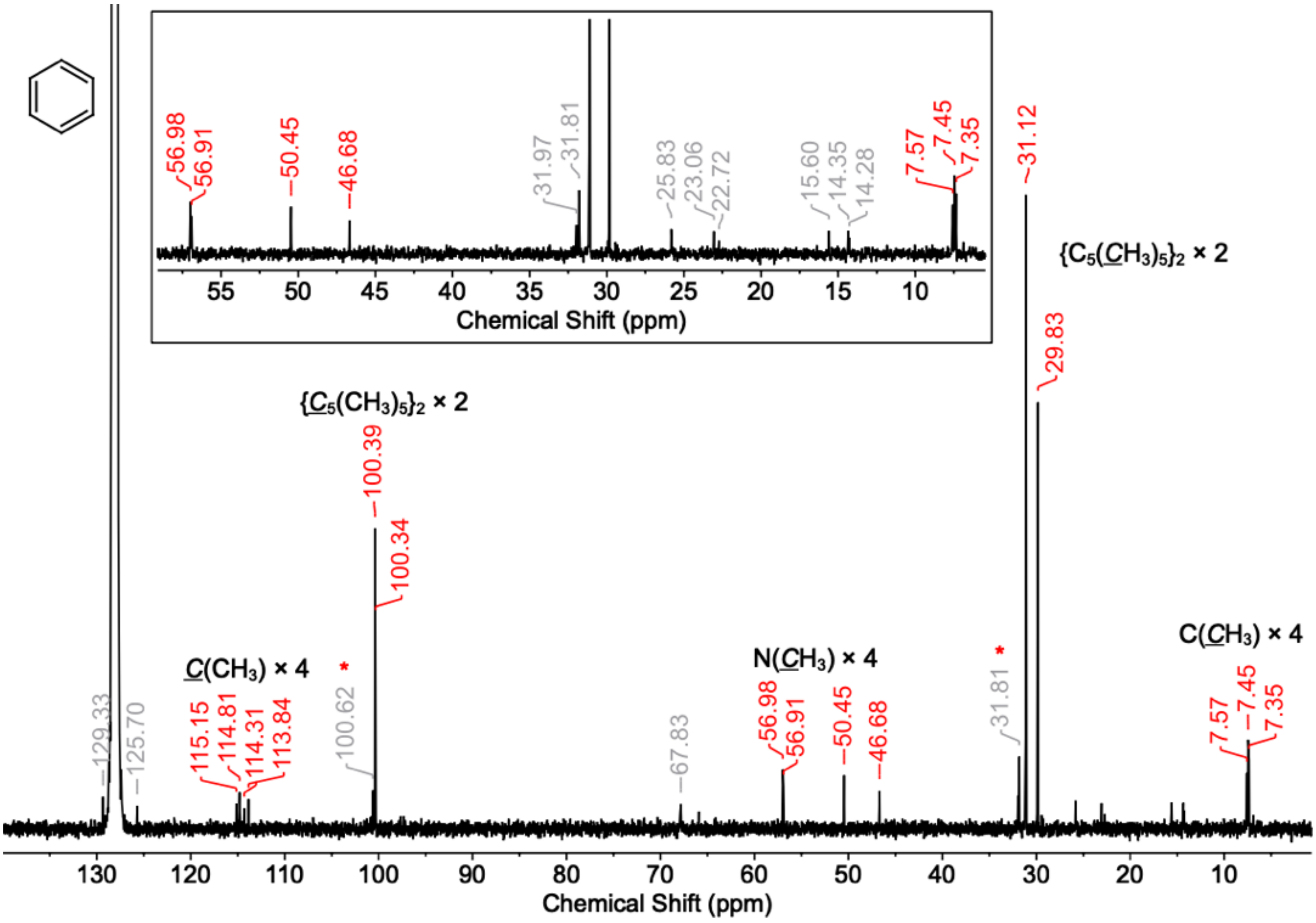

Figure S67. $^{13}C\{^1H\}$ NMR spectrum of $[Am(Cp^*)_2(I_xCl_{1-x})(I^{Me4})]$ (**4Am**) in D_6-benzene. No other peaks with significant intensity were observed from +555 to −155 ppm. Collected at 295 K.

*Comparison of ^{1}H NMR spectra between $[Nd(Cp^*)_2(X)(I^{Me4})]$ (X = I, **2Nd**; Cl, **5Nd**)*

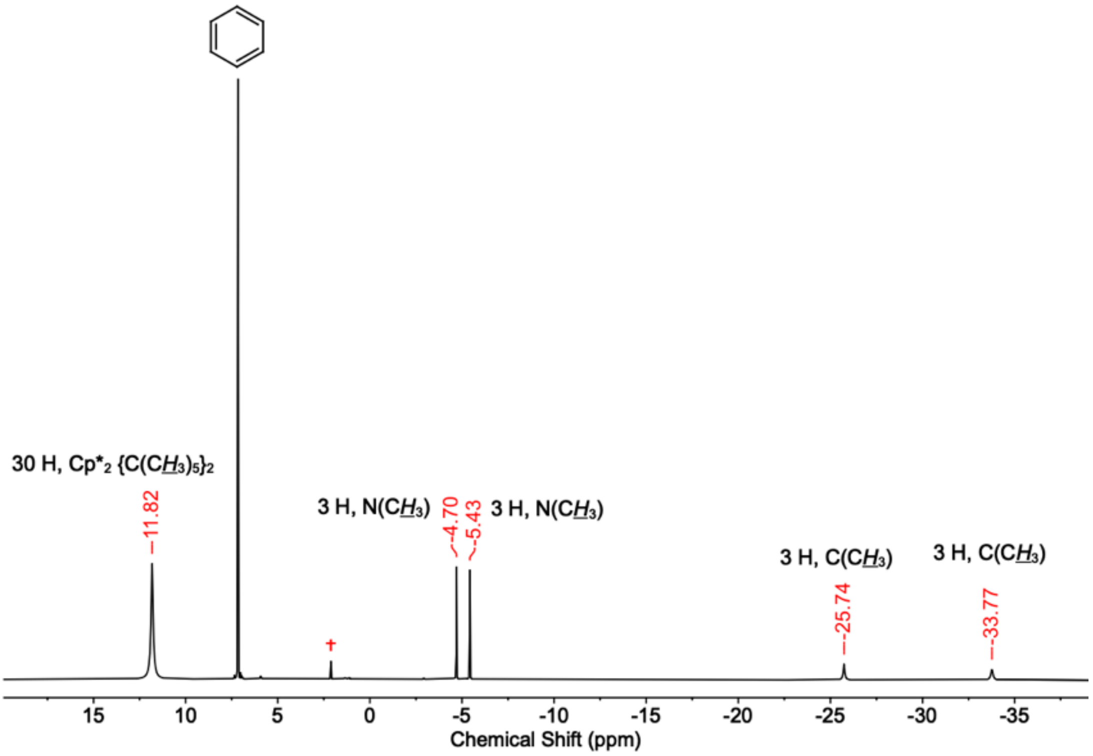

Figure S68. ^{1}H NMR spectrum of $[Nd(Cp^*)_2(I)(I^{Me4})]$ (**2Nd**) in D_6-benzene. † denotes toluene C*H*3.

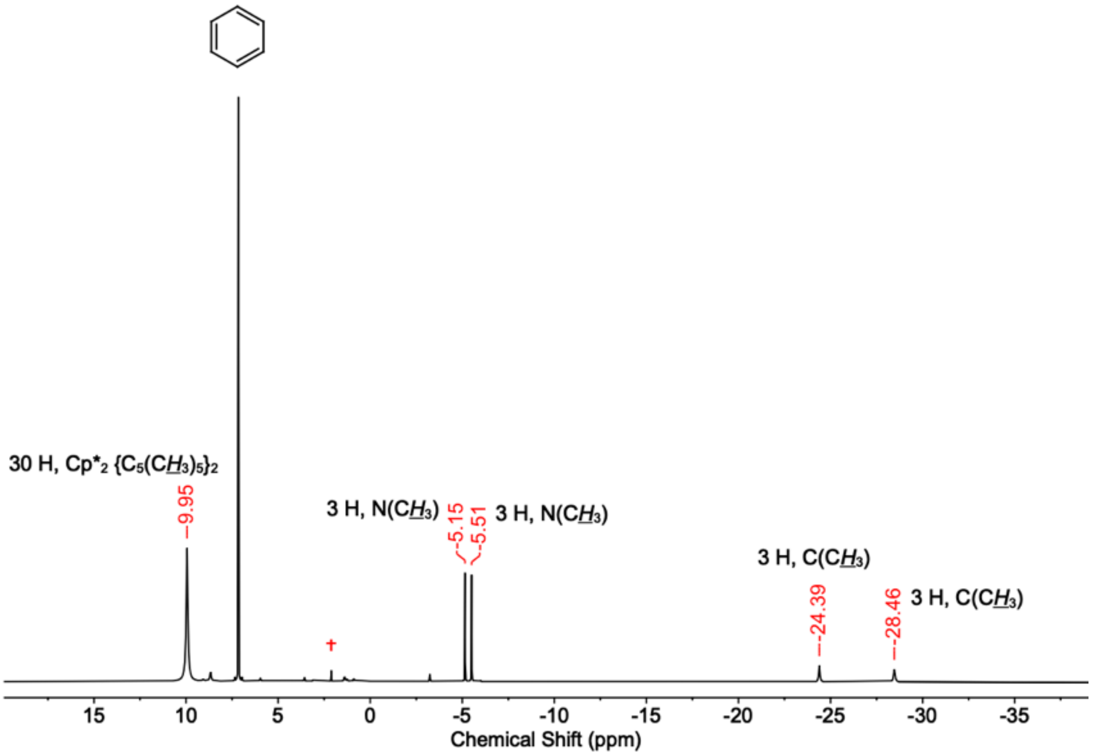

Figure S69. ^{1}H NMR spectrum of $[Nd(Cp^*)_2(Cl)(I^{Me4})]$ (**5Nd**) in D_6-benzene. † denotes toluene C*H*3.

Magnetic moments determined by NMR spectroscopy (Evans method)

Table S16. Data for the determination of the magnetic moments of complexes **1M** and **2M** (M = Ce, Pr, Nd, Np, Pu), and **5Nd**.

Sample / peak	μ_{eff} / B.M mol^{-1}	[a] mass of sample / g	mass of solvent / g	M_r / g mol^{-1}	[b] Δ peak / Hz
[Ce(Cp*)2(I)(THF)] (**1Ce**) [c]	**2.468**	0.0064	0.3550	609.578	106.80
[Ce(Cp*)2(I)(I^{Me4})] (**2Ce**)	**2.475**	0.0063	0.3520	661.656	95.60
[Pr(Cp*)2(I)(THF)] (**1Pr**) [c]	**3.484**	0.0061	0.3690	610.370	206.40
[Pr(Cp*)2(I)(I^{Me4})] (**2Pr**)	**3.185**	0.0063	0.4010	662.448	148.10
[Nd(Cp*)2(I)(THF)] (**1Nd**) [c]	**3.582**	0.0062	0.3630	613.704	224.80
[Nd(Cp*)2(I)(I^{Me4})] (**2Nd**)	**3.524**	0.0067	0.4600	665.782	169.80
[Nd(Cp*)2(Cl)(I^{Me4})] (**5Nd**)	**3.677**	0.0028	0.3380	574.331	123.61
[Np(Cp*)2(I)(THF)] (**1Np**)	**2.229**	0.0029	0.3259	706.511	35.01
[Np(Cp*)2(I)(I^{Me4})] (**2Np**)	**2.422**	0.0062	0.3652	758.588	75.40
[Pu(Cp*)2(I)(THF)] (**1Pu**)	**0.967**	0.0042	0.3792	708.527	0.95
[Pu(Cp*)2(I)(I^{Me4})] (**2Pu**)	**1.194**	0.0065	0.3675	760.604	8.19

[a] The small masses engender large errors in this methodology, the results should be cautiously interpreted along with other data. [b] Spectrometer frequency 400.130 MHz. Diamagnetic correction of M_r / −2,000,000 applied. ρD6-benzene = 0.950 g mL^{-1}. [c] The addition of a small quantity of $[H]_8$-THF (12 mg) for each **1Ln** leads to a small error in the solvent density, though it is likely to be within the error of the calculation as a whole.

Ce^{3+}	$4f^1$	$^2F_{5/2}$	$g\sqrt{J(J+1)}$ = 2.54 μB		Np^{3+}	$5f^4$	$^5I_4$	= 2.68 μB
Pr^{3+}	$4f^2$	$^3H_4$	= 3.58 μB		Pu^{3+}	$5f^5$	$^6H_{5/2}$	= 0.85 μB
Nd^{3+}	$4f^3$	$^4I_{9/2}$	= 3.62 μB		Np^{3+} / Pu^{3+} are isoelectronic to Pm^{3+} / Sm^{3+}			

Note that while the Russel-Saunders term symbol is not a good descriptor for ions with large spin-orbit coupling, like Np and Pu, our values using this simple NMR experiment are remarkably close to the theoretical values within the Russel-Saunders regime.

S6. UV-vis-NIR spectra

UV-vis-NIR spectra of [Ln(Cp)$_2$(I)(THF)] (**1Ln**, Ln = La, Ce, Pr, Nd)*

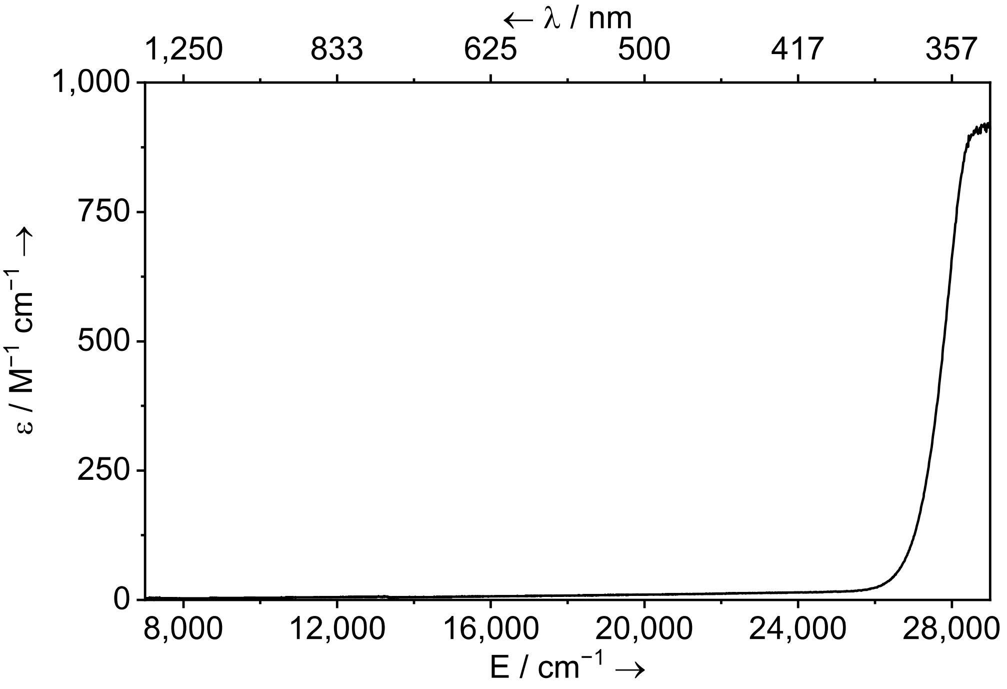

Figure S70. Solution UV-vis-NIR spectrum of [La(Cp*)$_2$(I)(THF)] (**1La**) (2.96 mM) in THF shown between 7,000–29,000 cm^{-1} (1,429–345 nm) at ambient temperature.

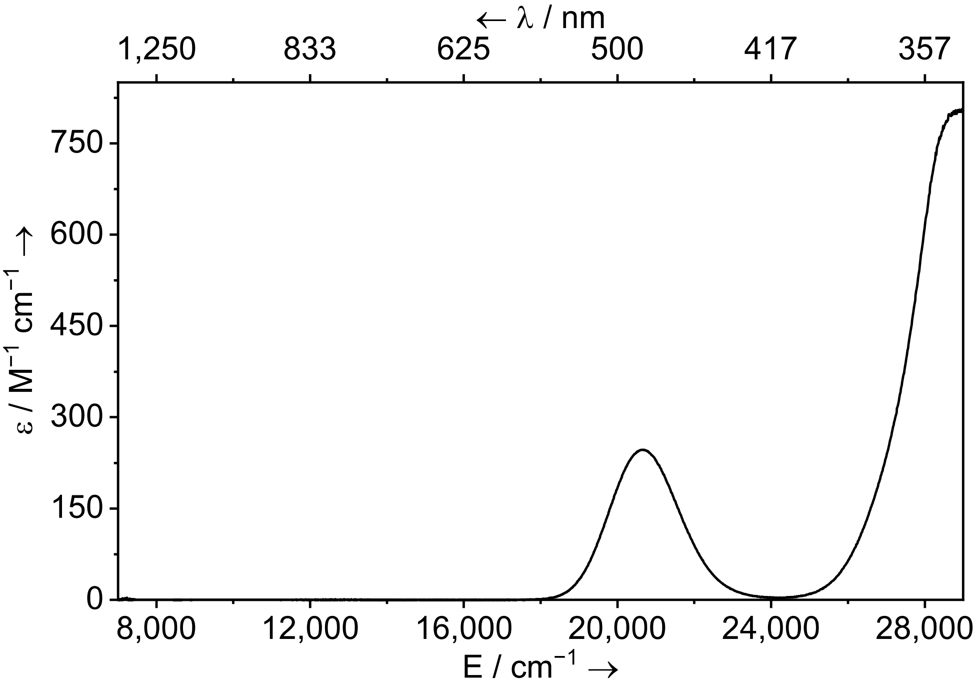

Figure S71. Solution UV-vis-NIR spectrum of [Ce(Cp*)$_2$(I)(THF)] (**1Ce**) (3.16 mM) in THF shown between 7,000–29,000 cm^{-1} (1,429–345 nm) at ambient temperature.

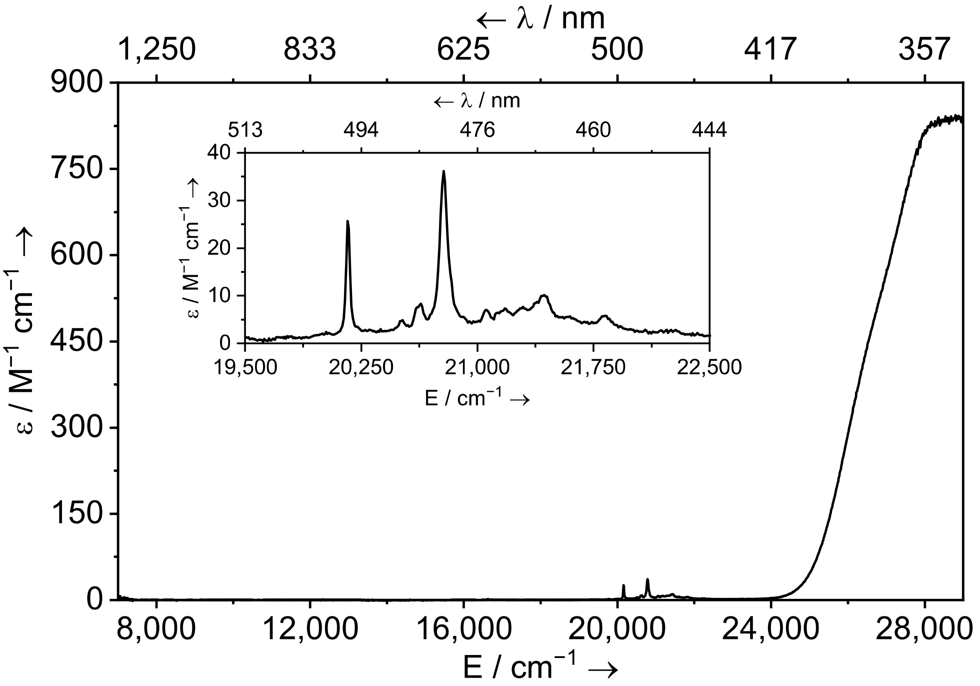

Figure S72. Solution UV-vis-NIR spectrum of [Pr(Cp*)$_2$(I)(THF)] (**1Pr**) (3.19 mM) in THF shown between 7,000–29,000 cm^{-1} (1,429–345 nm) at ambient temperature.

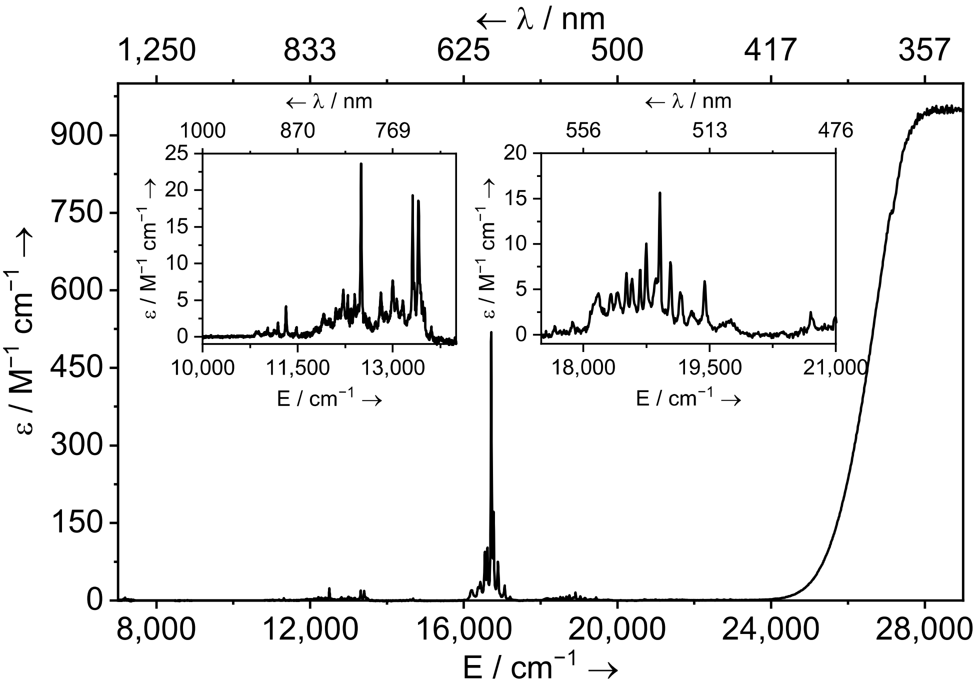

Figure S73. Solution UV-vis-NIR spectrum of [Nd(Cp*)$_2$(I)(THF)] (**1Nd**) (2.82 mM) in THF shown between 7,000–29,000 cm^{-1} (1,429–345 nm) at ambient temperature.

UV-vis-NIR spectra of [Ln(Cp)$_2$(I)(I^{Me4})] (**2Ln**, Ln = La, Ce, Pr, Nd, Y)*

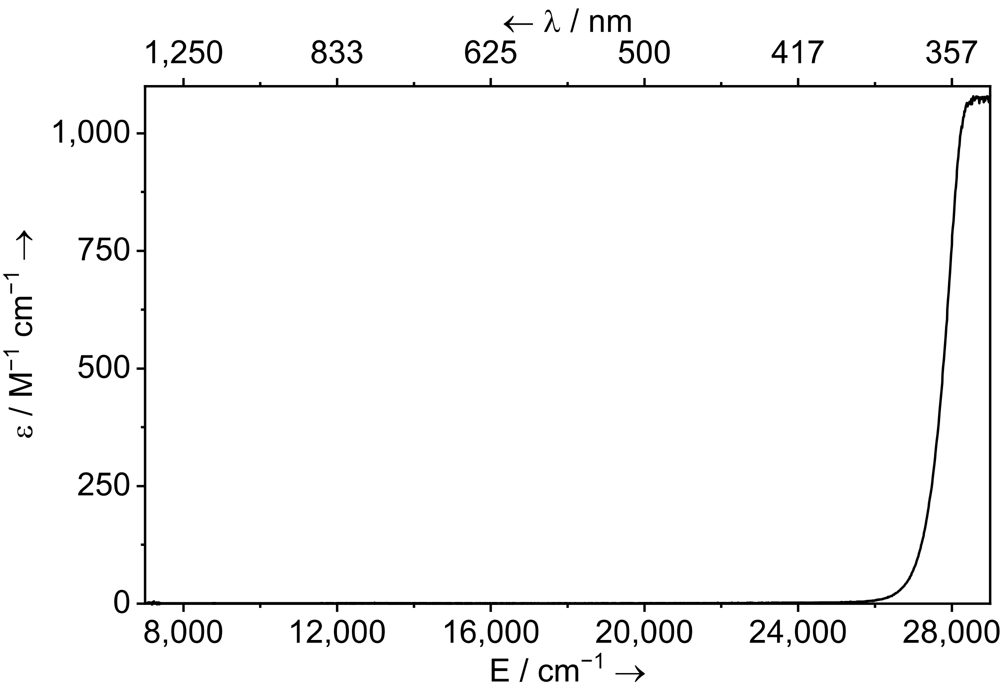

Figure S74. Solution UV-vis-NIR spectrum of [La(Cp*)$_2$(I)(I^{Me4})] (**2La**) (2.50 mM) in toluene shown between 7,000–29,000 cm^{-1} (1,429–345 nm) at ambient temperature.

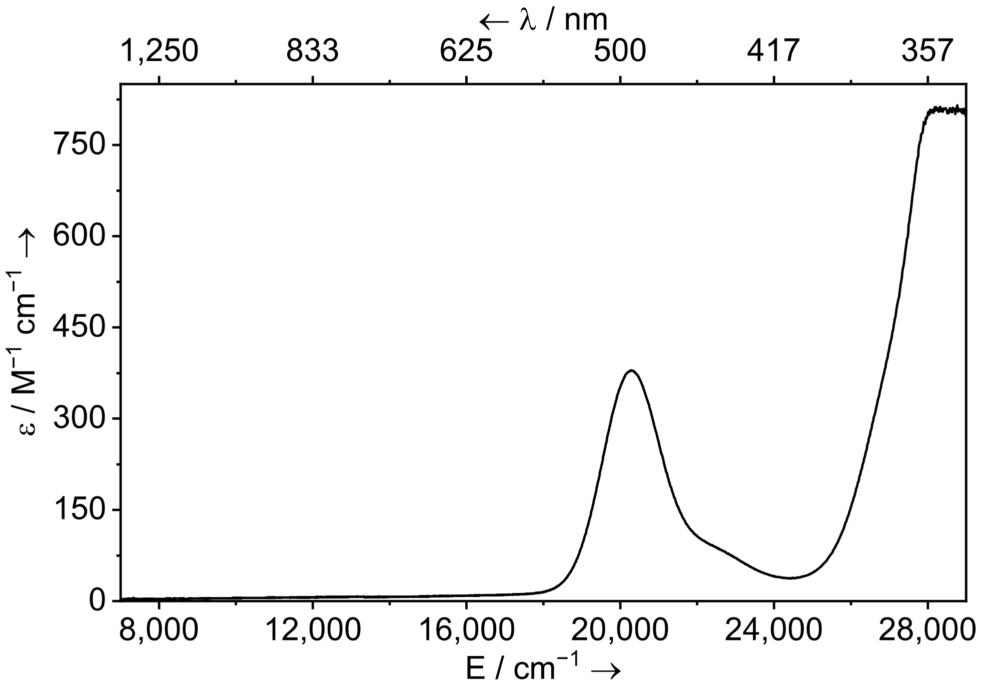

Figure S75. Solution UV-vis-NIR spectrum of [Ce(Cp*)$_2$(I)(I^{Me4})] (**2Ce**) (3.21 mM) in toluene shown between 7,000–29,000 cm^{-1} (1,429–345 nm) at ambient temperature.

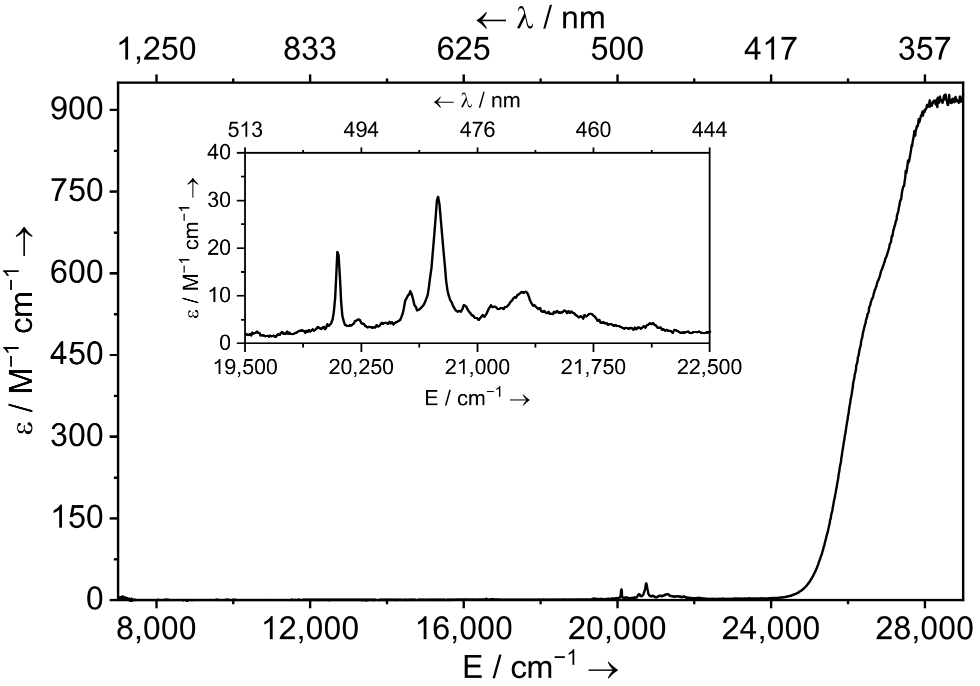

Figure S76. Solution UV-vis-NIR spectrum of $[Pr(Cp^*)_2(I)(I^{Me4})]$ (**2Pr**) (2.94 mM) in toluene shown between 7,000–29,000 cm^{-1} (1,429–345 nm) at ambient temperature.

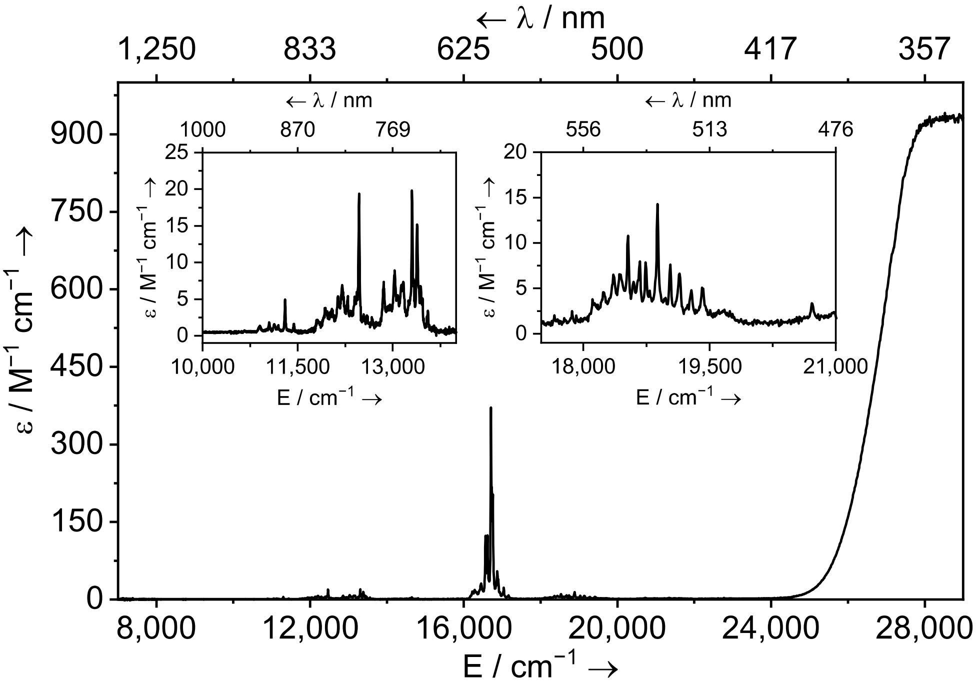

Figure S77. Solution UV-vis-NIR spectrum of $[Nd(Cp^*)_2(I)(I^{Me4})]$ (**2Nd**) (2.91 mM) in toluene shown between 7,000–29,000 cm^{-1} (1,429–345 nm) at ambient temperature.

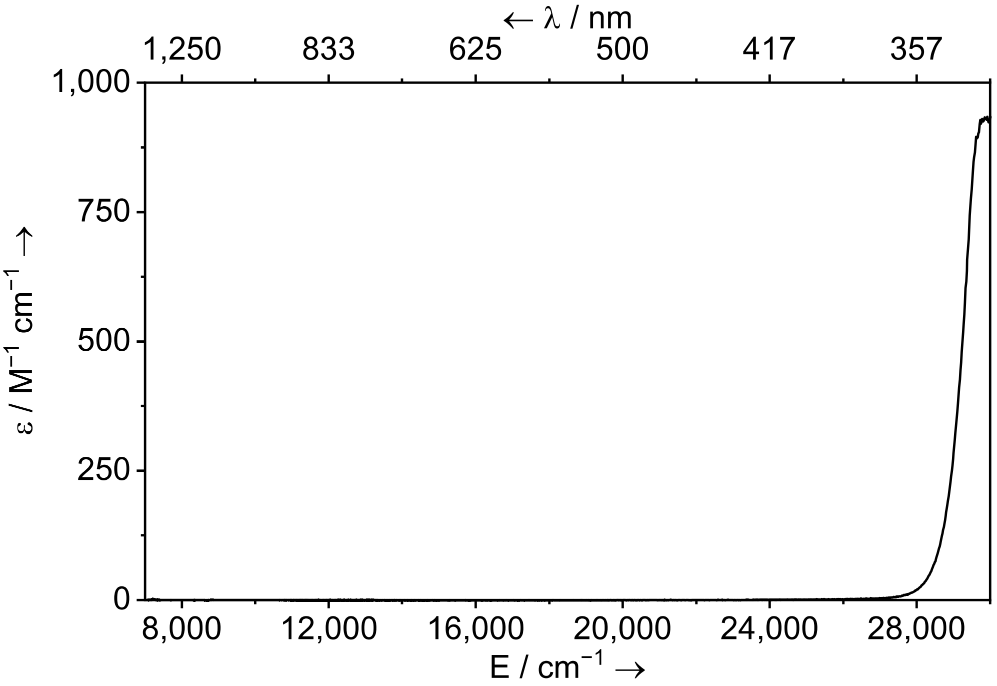

Figure S78. Solution UV-vis-NIR spectrum of $[Y(Cp^*)_2(I)(I^{Me4})]$ (**2Y**) (2.88 mM) in toluene shown between 7,000–30,000 cm^{-1} (1,429–333 nm) at ambient temperature.

*Comparison of UV-vis-NIR spectra between **1Ln** and **2Ln** (Ln = La, Ce, Pr, Nd)*

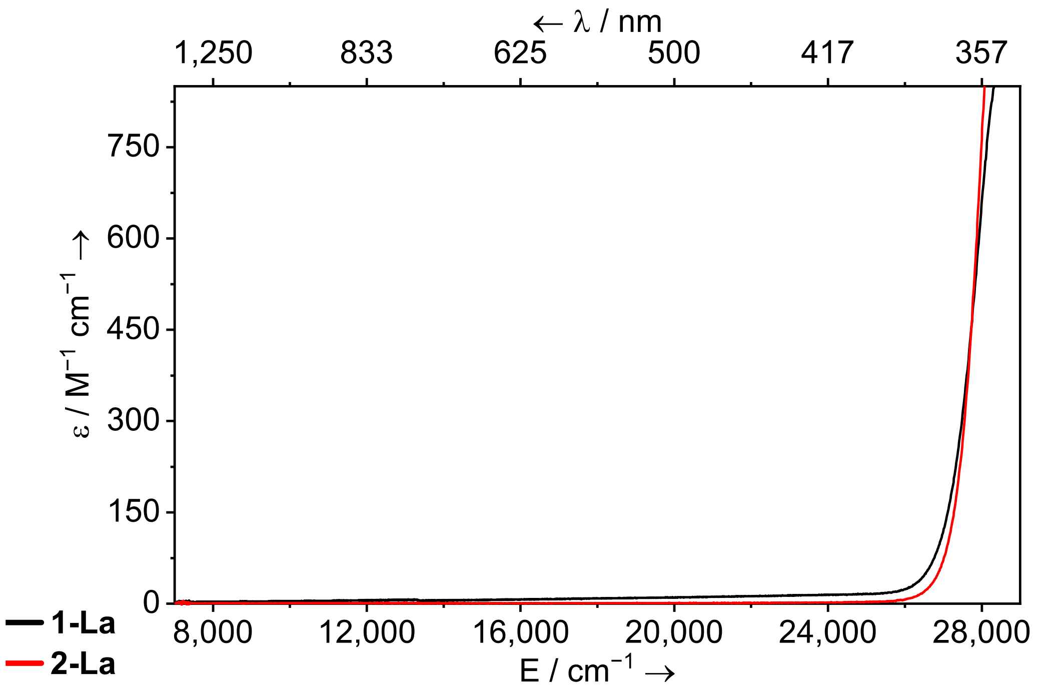

Figure S79. Solution UV-vis-NIR spectra of [La(Cp*)$_2$(I)(THF)] (**1La**) (2.96 mM, THF, black line) and [La(Cp*)$_2$(I)(I^{Me4})] (**2La**) (2.50 mM, toluene, red line) shown between 7,000–29,000 cm^{-1} (1,429–345 nm) at ambient temperature.

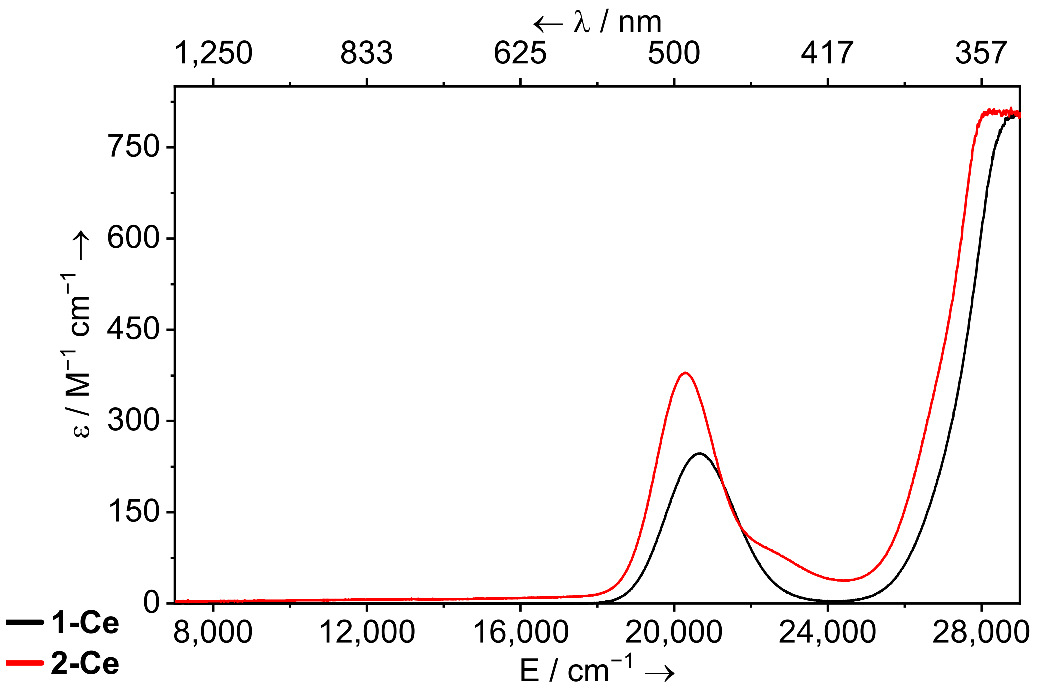

Figure S80. Solution UV-vis-NIR spectra of [Ce(Cp*)$_2$(I)(THF)] (**1Ce**) (3.16 mM, THF, black line) and [Ce(Cp*)$_2$(I)(I^{Me4})] (**2Ce**) (3.21 mM, toluene, red line) shown between 7,000–29,000 cm^{-1} (1,429–345 nm) at ambient temperature.

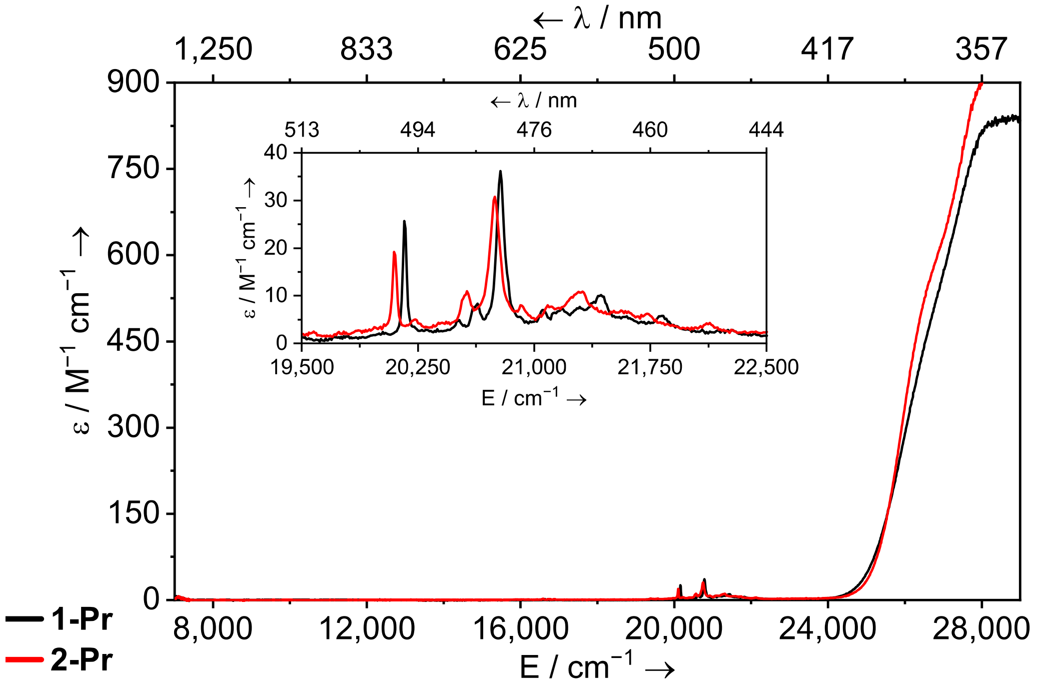

Figure S81. Solution UV-vis-NIR spectra of [Pr(Cp*)2(I)(THF)] (**1Pr**) (3.19 mM, THF, black line) and [Pr(Cp*)2(I)(I^{Me4})] (**2Pr**) (2.94 mM, toluene, red line) shown between 7,000–29,000 cm^{-1} (1,429–345 nm) at ambient temperature.

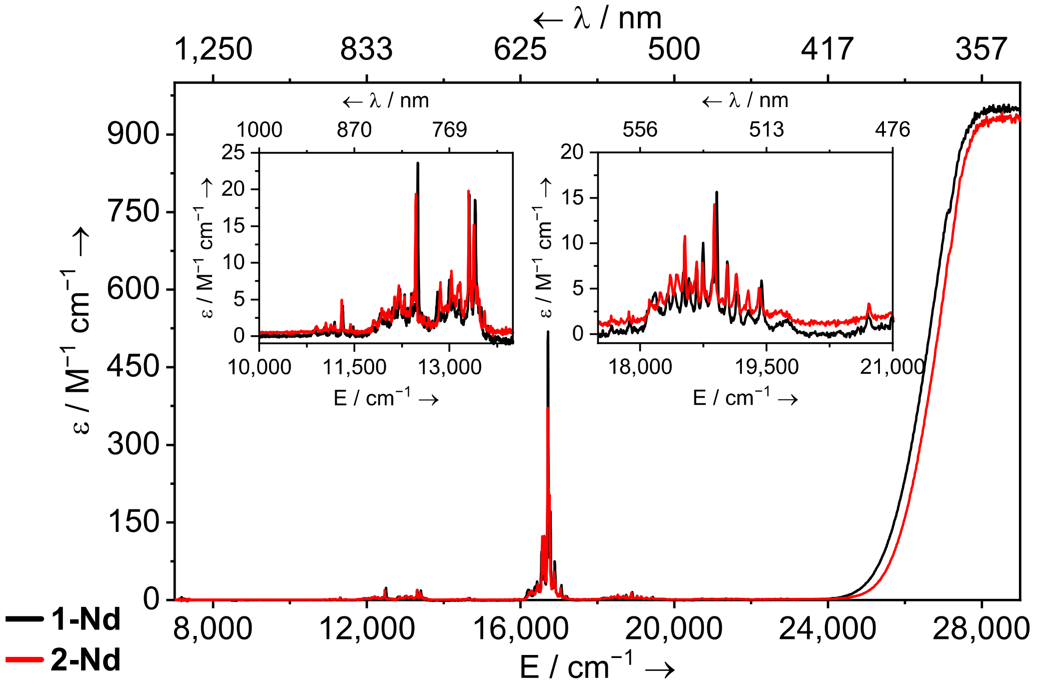

Figure S82. Solution UV-vis-NIR spectra of [Nd(Cp*)2(I)(THF)] (**1Nd**) (2.82 mM, THF, black line) and [Nd(Cp*)2(I)(I^{Me4})] (**2Nd**) (2.91 mM, toluene, red line) shown between 7,000–29,000 cm^{-1} (1,429–345 nm) at ambient temperature.

UV-vis-NIR spectra of [Np(Cp)$_2$(I)(THF)] (**1Np**) and [Np(Cp*)$_2$(I)(I^{Me4})] (**2Np**)*

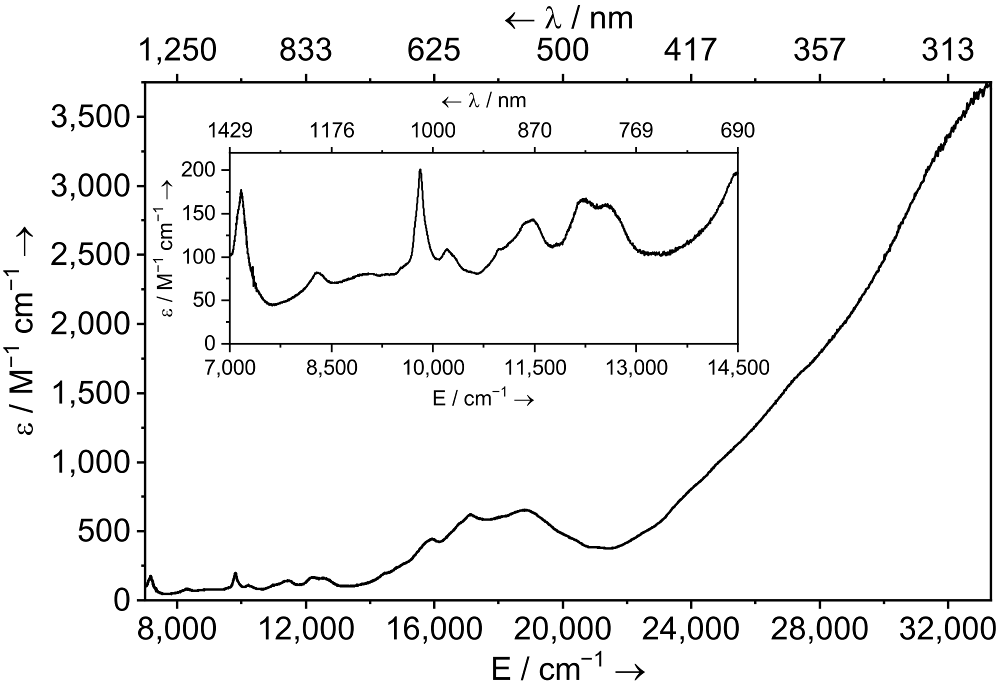

Figure S83. Solution UV-vis-NIR spectrum of [Np(Cp*)$_2$(I)(THF)] (**1Np**) (0.65 mM) in toluene shown between 7,000–33,333 cm^{-1} (1,429–333 nm) at ambient temperature. This is reproduced from prior work by some of us.[3]

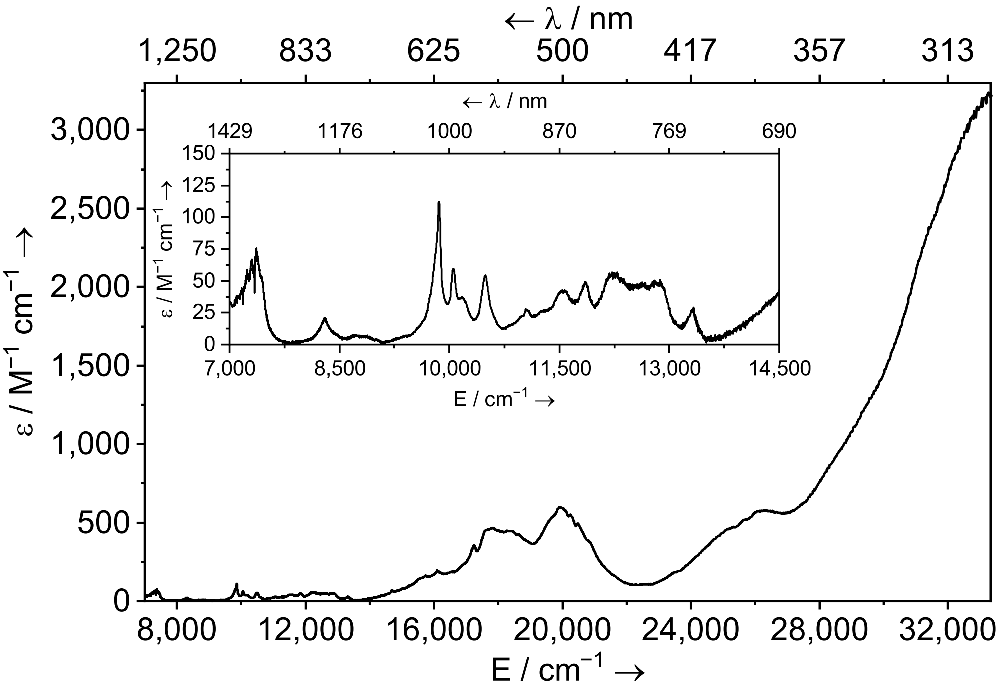

Figure S84. Solution UV-vis-NIR spectrum of [Np(Cp*)$_2$(I)(THF)] (**1Np**) (0.55 mM) in THF shown between 7,000–33,333 cm^{-1} (1,429–333 nm) at ambient temperature. This is reproduced from prior work by some of us.[3]

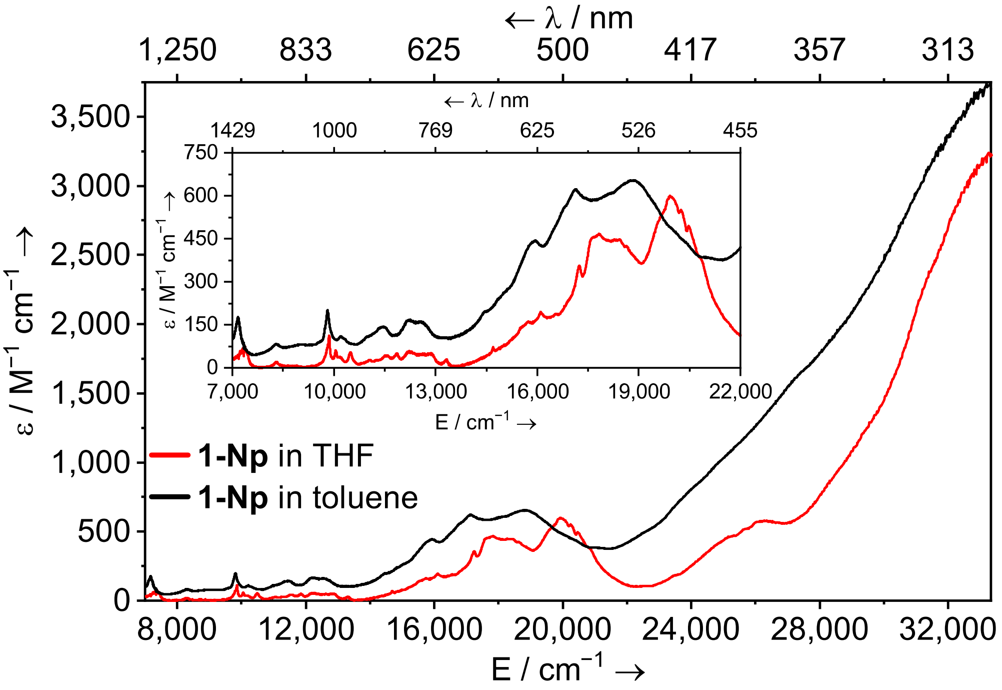

Figure S85. Solution UV-vis-NIR spectra of [Np(Cp*)$_2$(I)(THF)] (**1Np**) (0.65 mM, toluene, black line) and [Np(Cp*)$_2$(I)(THF)] (**1Np**) (0.55 mM, THF, red line) shown between 7,000–33,333 cm^{-1} (1,429–333 nm) at ambient temperature. The slight spectral differences might be due to a monomer/dimer equilibrium in toluene, related to a desolvation processes suggested in **Figure S9**.

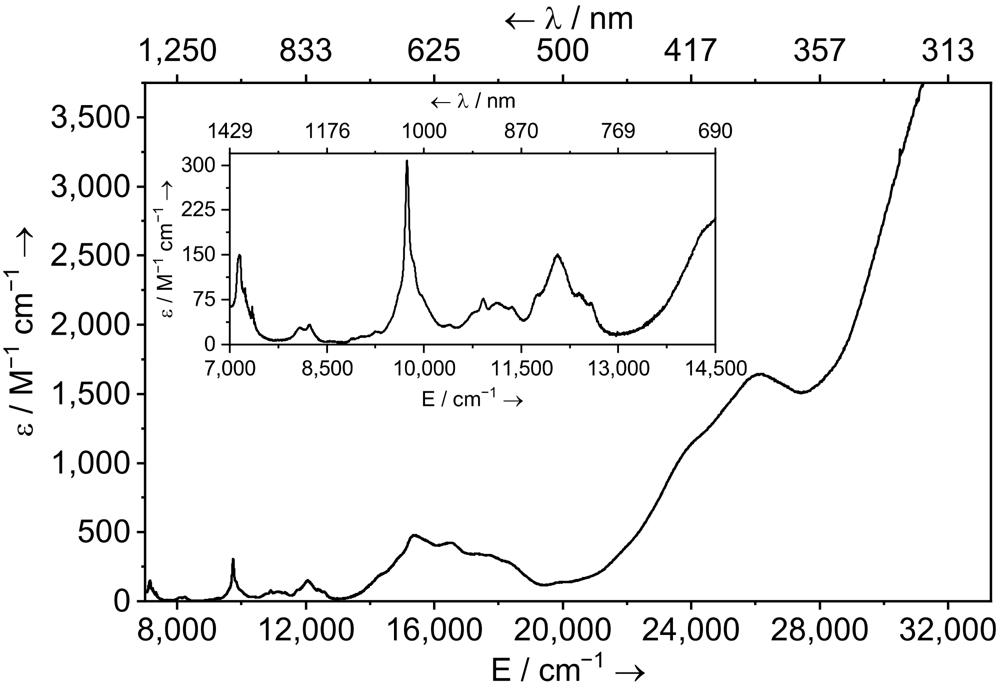

Figure S86. Solution UV-vis-NIR spectrum of [Np(Cp*)$_2$(I)(I^{Me4})] (**2Np**) (0.53 mM) in toluene shown between 7,000–33,333 cm^{-1} (1,429–333 nm) at ambient temperature.

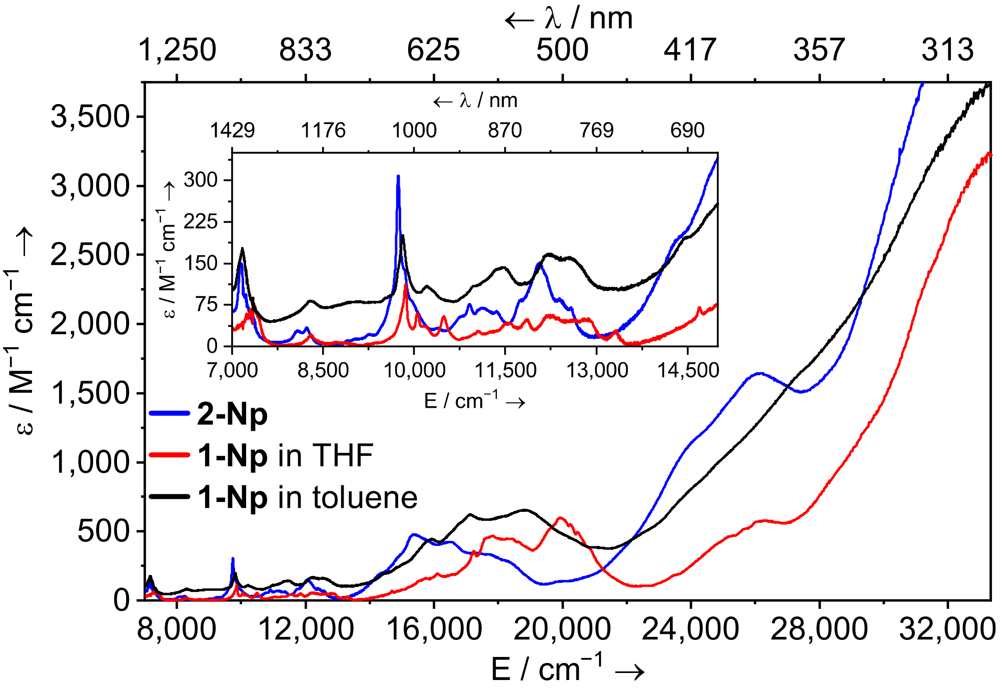

Figure S87. Solution UV-vis-NIR spectra of [Np(Cp*)$_2$(I)(THF)] (**1Np**) (0.65 mM, toluene, black line), [Np(Cp*)$_2$(I)(THF)] (**1Np**) (0.55 mM, THF, red line), and [Np(Cp*)$_2$(I)(I^{Me4})] (**2Np**) (0.53 mM, toluene, blue line) shown between 7,000–33,333 cm^{-1} (1,429–333 nm) at ambient temperature.

UV-vis-NIR spectra of [Pu(Cp)$_2$(I)(THF)] (**1Pu**) and [Pu(Cp*)$_2$(I)(I^{Me4})] (**2Pu**)*

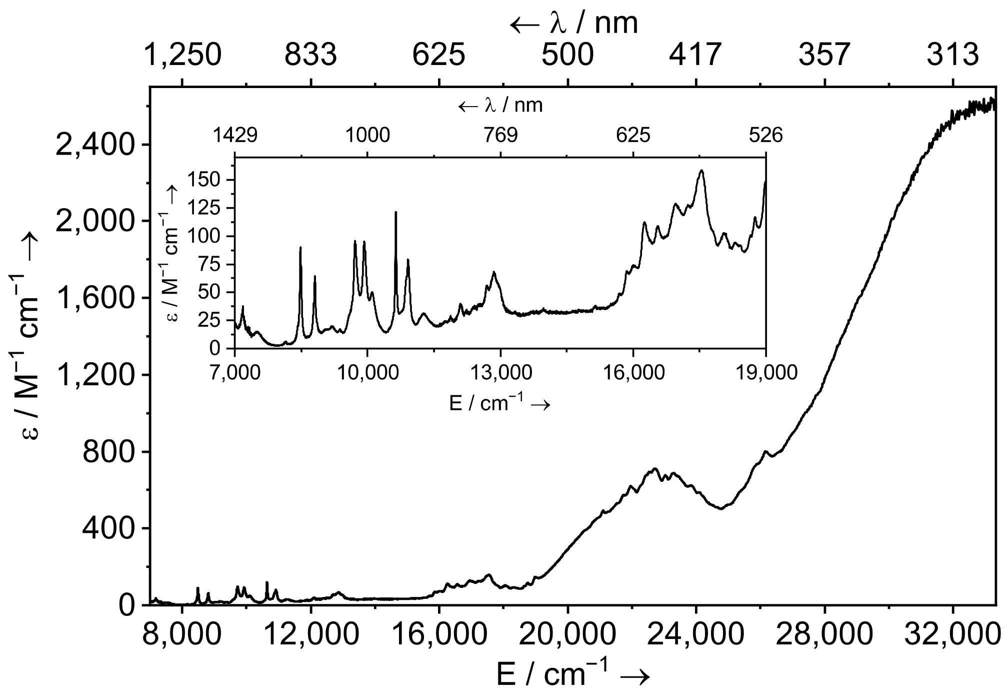

Figure S88. Solution UV-vis-NIR spectrum of [Pu(Cp*)$_2$(I)(THF)] (**1Pu**) (1.00 mM) in toluene shown between 7,000–33,333 cm^{-1} (1,429–333 nm) at ambient temperature. This is reproduced from prior work by some of us.[3]

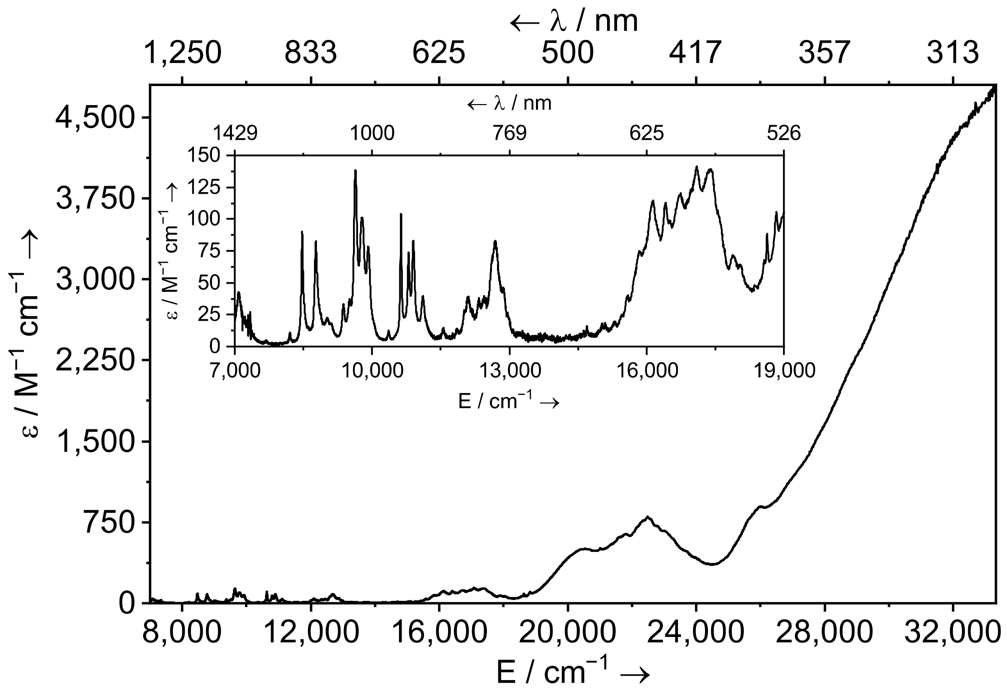

Figure S89. Solution UV-vis-NIR spectrum of [Pu(Cp*)$_2$(I)(I^{Me4})] (**2Pu**) (0.52 mM) in toluene shown between 7,000–33,333 cm^{-1} (1,429–333 nm) at ambient temperature.

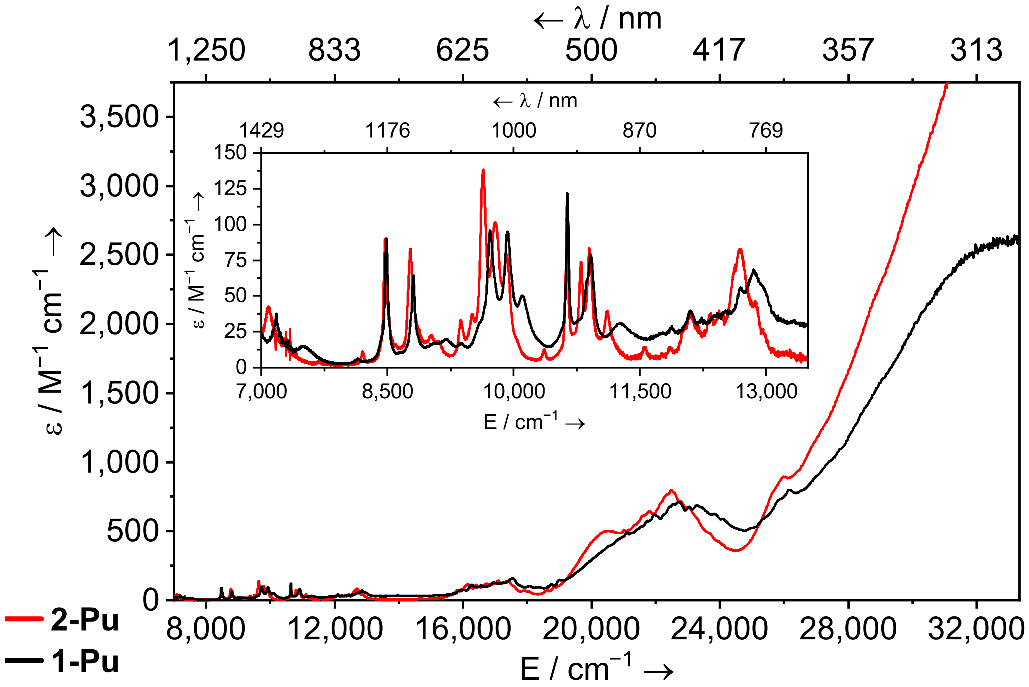

Figure S90. Solution UV-vis-NIR spectra of [Pu(Cp*)$_2$(I)(THF)] (**1Pu**) (1.00 mM, toluene, black line) and [Pu(Cp*)$_2$(I)(I^{Me4})] (**2Pu**) (0.52 mM, toluene, red line) shown between 7,000–33,333 cm^{-1} (1,429–333 nm) at ambient temperature.

UV-vis-NIR spectrum of [Am(Cp)$_2$(I$_x$Cl$_{1-x}$)(I^{Me4})] (**4Am**).*

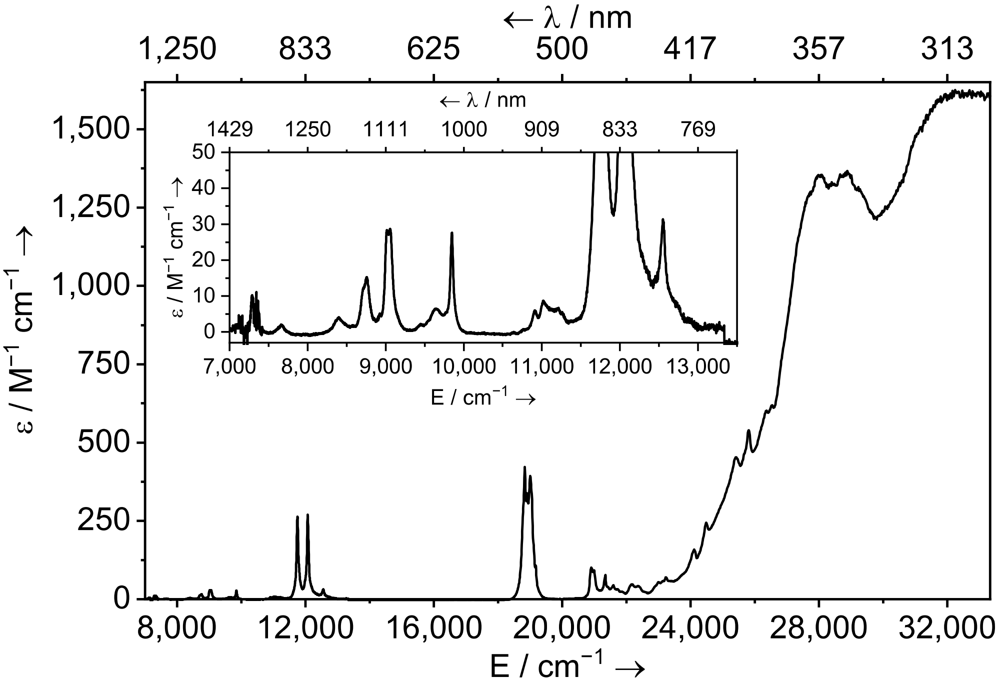

Figure S91. Solution UV-vis-NIR spectrum of [Am(Cp*)$_2$(I$_x$Cl$_{1-x}$)(I^{Me4})] (**4Am**) (*ca*. 1.3 mM, toluene, shown between 7,000–33,333 cm^{-1} (1,429–333 nm) at ambient temperature. Note the sets of doubled peaks at *ca.* 12,000 cm^{-1} and *ca*. 19,000 cm^{-1} – likely one peak from each halide complex.

Comparison of UV-vis-NIR spectra between [Nd(Cp)$_2$(X)(I^{Me4})] (X = I, **2Nd**; Cl = **5Nd**)*

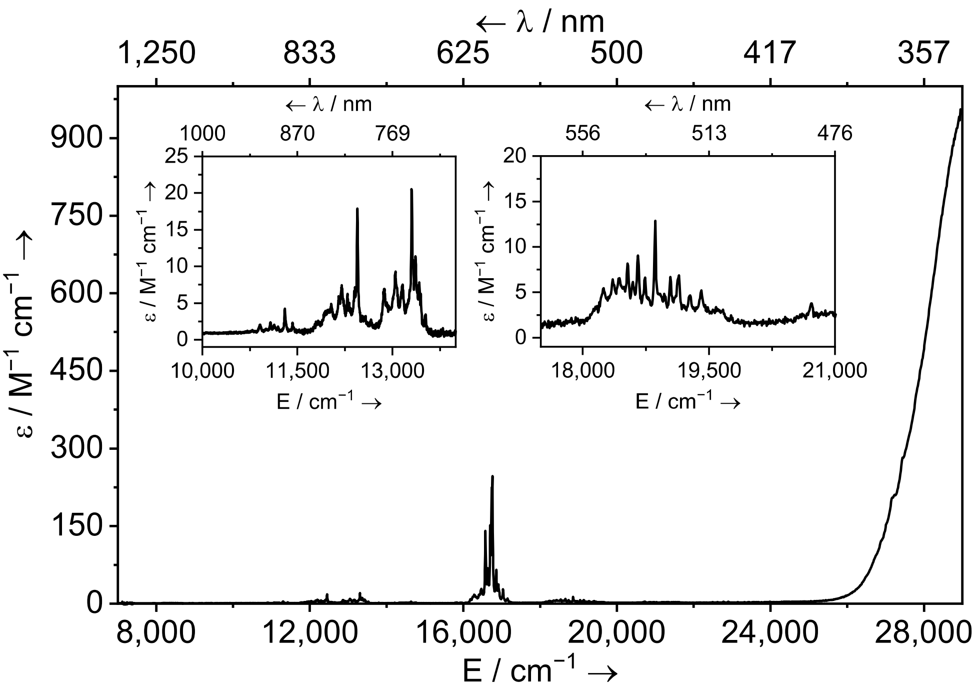

Figure S92. Solution UV-vis-NIR spectrum of [Nd(Cp*)$_2$(Cl)(I^{Me4})] (**5Nd**) (2.66 mM) in toluene shown between 7,000–30,000 cm^{-1} (1,429–333 nm) at ambient temperature.

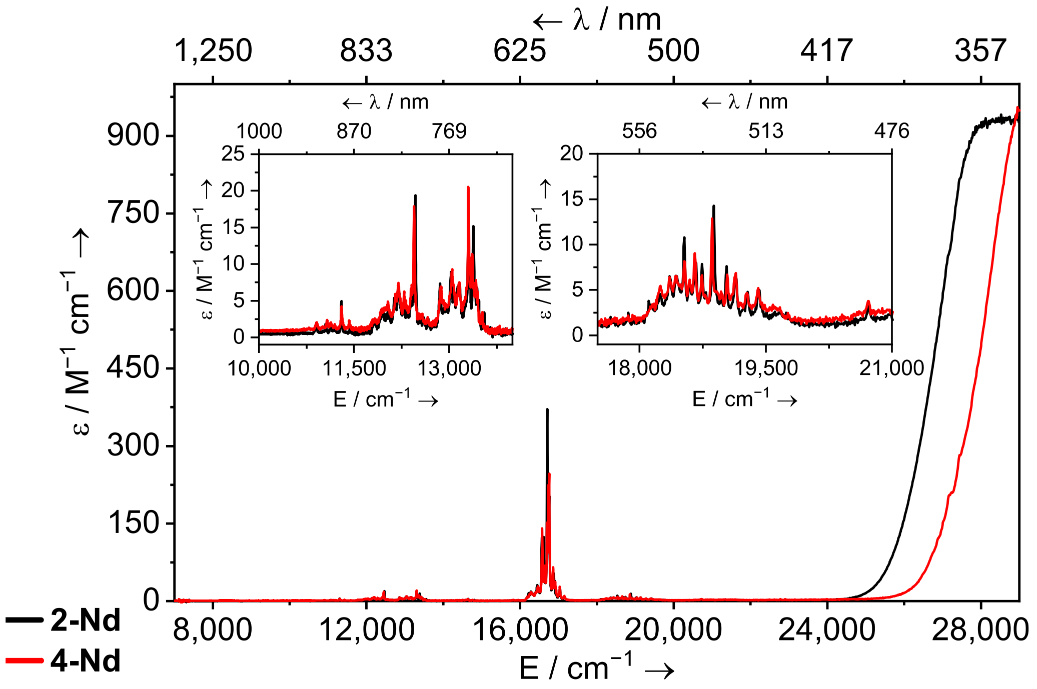

Figure S93. Solution UV-vis-NIR spectra of [Nd(Cp*)$_2$(I)(I^{Me4})] (**2Nd**) (2.91 mM, toluene, black line) and [Nd(Cp*)$_2$(Cl)(I^{Me4})] (**5Nd**) (2.66 mM, toluene, red line) shown between 7,000–29,000 cm^{-1} (1,429–345 nm) at ambient temperature. The two are essentially identical whereas the spectrum of **4Am** showed noticeable doubling of some sets of peaks with significant shifts between them.

*Gaussian fitting of 6d←5f peaks for **1Pu**, **2Pu**, **1Np**, and **2Np***

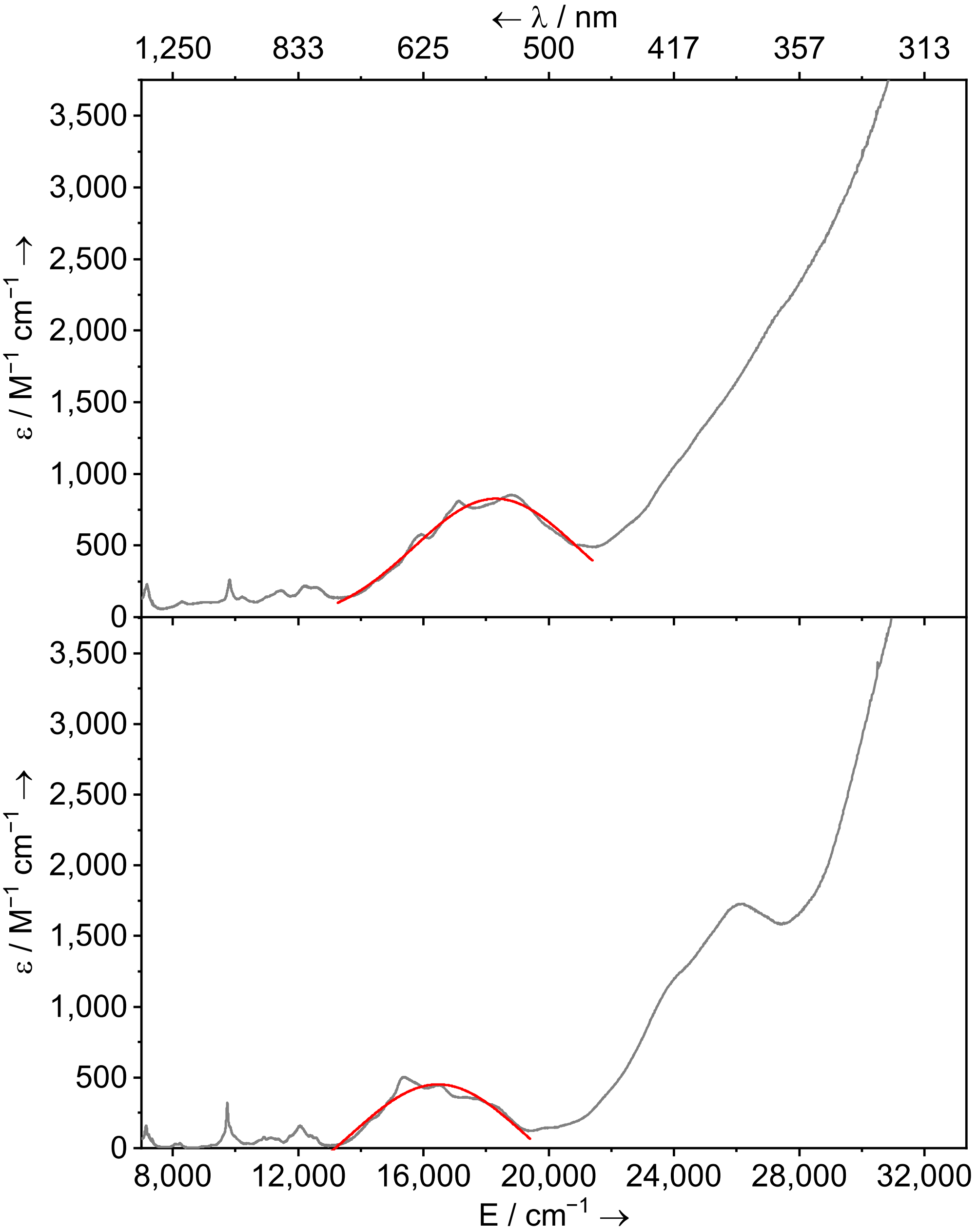

Figure S94. Solution UV-vis-NIR spectra of [Np(Cp*)$_2$(I)(THF)] (**1Np**, top) and [Np(Cp*)$_2$(I)(I^{Me4})] (**2Np**, bottom) between 7,000–33,333 cm^{-1} (1,429–333 nm) at ambient temperature. The red curve denotes a Gaussian fit over the broad 6*d*←5*f* transition feature. See **Table S17** for the fitting parameters.

Table S17. Gaussian fit parameters for **1Np** and **2Np**.

	**1Np**		**2Np**	
	Value	Error	Value	Error
y_0	-33.09905	7.03864	-456.58158	60.64984
x_C	18308.6725	6.20095	16466.51481	8.50545
w	5231.78027	42.0305	5598.96305	248.8601
A	5638655.96769	85286.44262	6367940.67069	699072.7684
σ	2615.89013	21.01525	2799.48152	124.43005
**FWHM**	6159.95052	49.48713	6592.27521	293.01037
**Height**	859.93607	6.491	907.46831	59.48655
**Reduced** χ^2	860.34659	–	1655.93704	–
**Adjusted** R^2	0.98512	–	0.92622	–

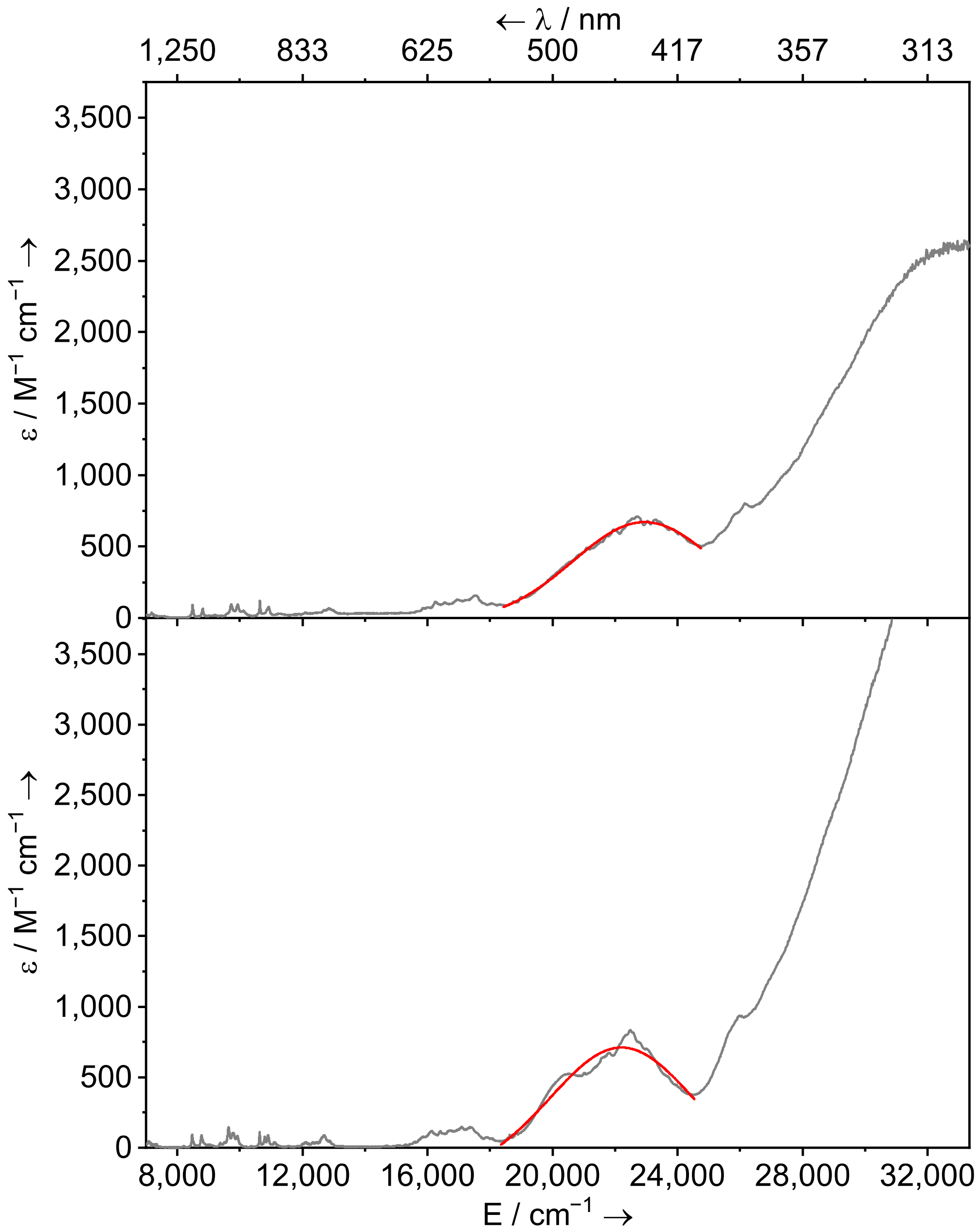

Figure S95. Solution UV-vis-NIR spectra of [Pu(Cp*)$_2$(I)(THF)] (**1Pu**, top) and [Pu(Cp*)$_2$(I)(I^{Me4})] (**2Pu**, bottom) between 7,000–33,333 cm^{-1} (1,429–333 nm) at ambient temperature. The red curve denotes a Gaussian fit over the broad 6*d*←5*f* transition feature. See **Table S18** for the fitting parameters.

Table S18. Gaussian fit parameters for **1Pu** and **2Pu**.

	**1Pu**		**2Pu**	
	Value	Error	Value	Error
y_0	-22.74408	5.01648	-195.49616	28.44239
x_C	22947.30389	6.48771	22210.75979	12.57881
w	4590.48156	36.08926	4575.01683	121.17959
A	3991547.02435	56157.61448	5194880.53936	287831.88624
σ	2295.24078	18.04463	2287.50842	60.5898
**FWHM**	5404.879	42.49185	5386.67067	142.67807
**Height**	693.78206	4.61936	905.98901	26.91435
**Reduced χ^2**	218.47089	–	2441.66891	–
**Adjusted R^2**	0.99465	–	0.9506	–

S7. Quantum chemical calculations

General considerations

The Gaussian 16 software package, revision C.01, was used for all density functional theory (DFT) calculations.[27] The hybrid density functional approximation, PBE0,[28, 29] was used with Grimme's D3[30] and the Becke-Johnson damping parameters for dispersion corrections (bar calculations on the Am systems, as these corrections are not available for Am).[31-33] Dunning's correlation consistent basis sets of polarized triple-ζ quality were employed for C, N and Cl, with the double-ζ variant used for H.[34-37] Stuttgart-Bonn relativistic effective core potentials (ECP) were used for the iodine (46 electrons), lanthanide (28 electrons) and actinide (60 electrons) atoms, and the associated segmented valence basis sets were used.[38-40] The spin states computed were the highest possible resulting from the number of unpaired f electrons expected for M(III).

Geometry optimizations of all the systems bar those containing Am were performed starting from the crystallographic co-ordinates. Default settings were used for the optimizations. Analysis of the harmonic vibrational frequencies confirmed the optimized geometries as energetic minima. For the Am molecules, single point calculations were performed at the crystallographic co-ordinates.

30-state, full TDDFT calculations were performed.

Electron density-based analysis of metal–ligand bonding utilized the AIMAll software package,[41] using the WFX files generated from the DFT calculations (in Gaussian 16).

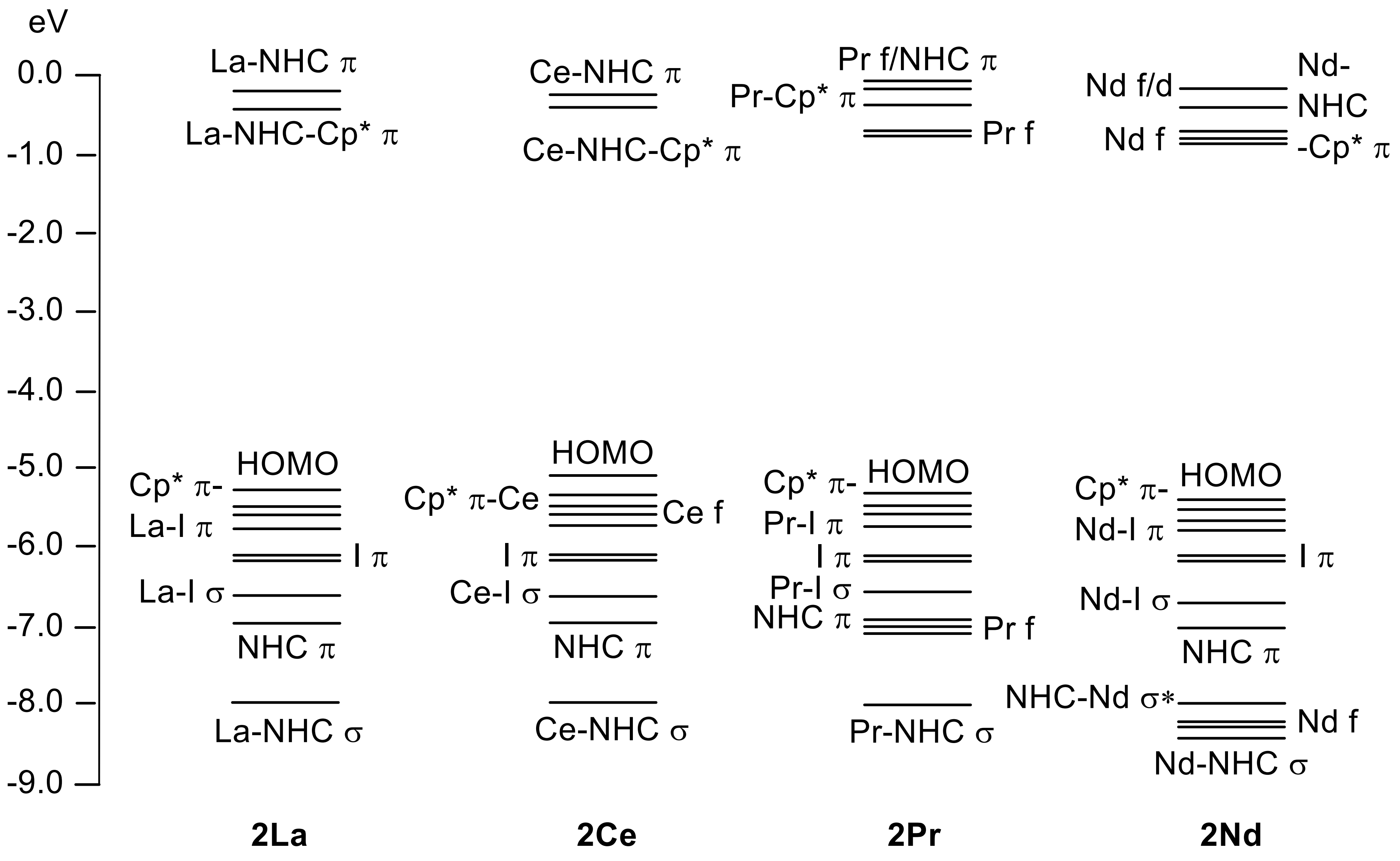

Figure S96. Molecular orbital energy level diagrams for **2Ln** (Ln = La, Ce, Pr, Nd), obtained from the optimized geometries. Principal orbital character is indicated, though sometimes indicated character is present in other, energetically-close, orbitals. HOMO = highest occupied molecular orbital.

TD-DFT calculations

To further characterize the UV-vis-NIR absorption spectra of **1Ce** and **2Ce** and determine the nature of the low-energy transition, TD-DFT calculations were performed on both. Geometry optimization was performed on **1Ce**, and the previously optimized geometry of **2Ce** was used. The transitions found experimentally at 20,675 cm^{-1} and 20,308 cm^{-1} for the THF and I^{Me4} systems, respectively, are reproduced reasonably well, at 22,768 cm^{-1} and 22,532 cm^{-1}; the experimental redshift of 367 cm^{-1} reduces to 236 cm^{-1} by calculation. The orbital nature of the transition is very mixed at the Kohn-Sham level, with that for **1Ce** involving six orbital pairs with significant coefficients and that for **2Ce** eight orbital pairs. To better understand the computed transition, we calculated the Natural Transition Orbitals (NTOs) associated with it. In both complexes, the transition is essentially entirely between two NTOs; these are shown for **2Ce** in **Figure S97**, and yield a much clearer orbital picture, confirming the experimental assignment of an $5d \leftarrow 4f$ transition. Note that as there are no energies associated with NTOs, we cannot comment on the experimentally observed redshift at the NTO level. The single-determinantal nature of DFT means that TD-DFT is not well-suited to computing f-based electronic transitions in complexes with many unpaired f electrons, such as the remainder of our **2M** series, and so this analysis could not be reliably extended. Nevertheless, these calculations lend confidence to the assignment of transitions in **1Ce** and **2Ce** shown in the main text and **Figure S71**, **Figure S75**, and **Figure S80**.

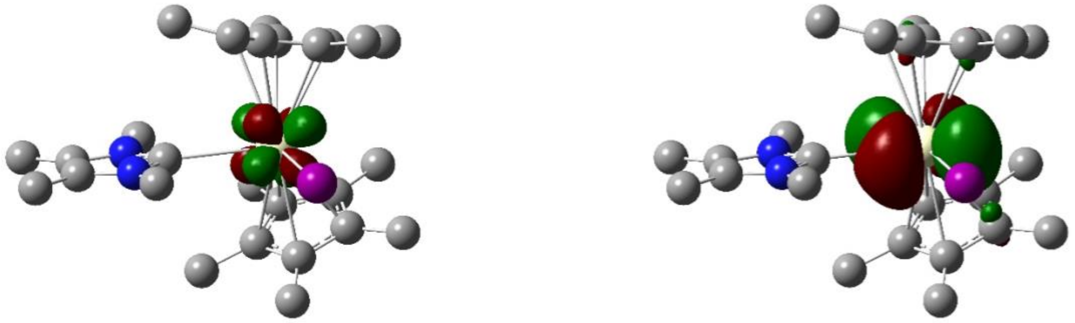

Figure S97. Natural Transition Orbitals associated with the transition at 20,308 cm^{-1} in **2Ce**, isovalue = 0.05. Hydrogen atoms omitted for clarity. NTO128 (left) is 97.8% Ce 4f (Mulliken analysis) and NTO129 (right) is 74.4% Ce 5d. Analogous NTOs for **1Ce** are 89.7 4f/1.6% 5d and 77.2% 5d, respectively.

Cartesian coordinates (Å) and SCF energies

2La

-1609.8486317 H

La	0.529444	0.197793	0.042644
I	1.563247	-2.708052	-0.547157
N	-2.893199	-1.311280	-0.282980
N	-3.158759	0.782169	-0.115792
C	-2.192410	-0.161213	-0.178345
C	-4.261646	-1.097498	-0.283747
C	-4.431700	0.243614	-0.176651
C	-2.311058	-2.629860	-0.377061
H	-1.222588	-2.553496	-0.395509
H	-2.638451	-3.125233	-1.295651
H	-2.605122	-3.240261	0.481894
C	-5.245957	-2.196895	-0.387327
H	-6.261502	-1.798406	-0.358473
H	-5.142084	-2.911208	0.436975
H	-5.131337	-2.756082	-1.322669
C	-5.657138	1.070604	-0.124415
H	-6.542926	0.437069	-0.193120
H	-5.696293	1.790139	-0.950181
H	-5.724010	1.637539	0.811237
C	-2.911517	2.192462	0.019900
H	-1.837665	2.356318	0.080560
H	-3.383301	2.581995	0.925972
H	-3.299721	2.740387	-0.843451
C	0.495879	1.644926	2.464537
C	1.767781	1.021510	2.411337

C	1.582083	-0.371334	2.582102
C	0.198533	-0.613167	2.724346
C	-0.473282	0.632242	2.668618
C	0.244755	3.116559	2.493798
H	-0.740862	3.377687	2.094720
H	0.988150	3.669833	1.912729
H	0.283962	3.509854	3.518378
C	3.099072	1.696157	2.394977
H	3.019600	2.753052	2.135153
H	3.797089	1.232307	1.688724
H	3.572790	1.638437	3.383684
C	2.681868	-1.366073	2.743392
H	3.103646	-1.309691	3.756526
H	3.502817	-1.194715	2.040453
H	2.330936	-2.386160	2.578161
C	-0.441880	-1.936197	2.983788
H	0.065242	-2.740798	2.442435
H	-1.492365	-1.936782	2.675141
H	-0.421864	-2.191011	4.051749
C	-1.908129	0.845523	3.021222
H	-2.051652	0.775455	4.107675
H	-2.571067	0.105452	2.563101
H	-2.254964	1.837936	2.724019
C	1.879240	2.214216	-1.372689
C	2.190425	1.006811	-2.043326
C	1.020663	0.561001	-2.700465
C	-0.011603	1.496430	-2.443168
C	0.519288	2.521862	-1.625824

C	2.860625	3.142564	-0.739940
H	3.248992	3.857997	-1.478182
H	3.722661	2.614173	-0.325991
H	2.412806	3.731111	0.066096
C	3.541667	0.375261	-2.108652
H	4.092779	0.489671	-1.168724
H	4.155636	0.833554	-2.895203
H	3.475391	-0.695633	-2.313359
C	0.898496	-0.602335	-3.626816
H	0.932321	-0.271297	-4.673914
H	-0.045499	-1.140499	-3.489789
H	1.700277	-1.326617	-3.474015
C	-1.350635	1.464038	-3.103507
H	-1.263908	1.700315	-4.172238
H	-2.039272	2.192573	-2.669910
H	-1.824722	0.479750	-3.032671
C	-0.120291	3.830234	-1.288545
H	-0.119284	4.052198	-0.215016
H	-1.154559	3.875437	-1.637099
H	0.414465	4.655324	-1.775463

2Ce

-1649.3151462 H

Ce	0.519295	0.197452	0.027131
I	1.572136	-2.708288	-0.387674
N	-2.867954	-1.348687	-0.142661
N	-3.139874	0.748071	-0.036814
C	-2.170995	-0.193764	-0.073181

C	-4.236781	-1.138831	-0.150728
C	-4.410774	0.204226	-0.083268
C	-2.283319	-2.668326	-0.192267
H	-1.195025	-2.589994	-0.201323
H	-2.600596	-3.191511	-1.098976
H	-2.586119	-3.253112	0.681419
C	-5.217853	-2.243660	-0.222172
H	-6.234471	-1.847063	-0.210474
H	-5.115698	-2.930561	0.625340
H	-5.097929	-2.832679	-1.138319
C	-5.638600	1.028869	-0.057383
H	-6.522494	0.391096	-0.109225
H	-5.677898	1.723733	-0.904007
H	-5.708888	1.622922	0.861018
C	-2.897024	2.162523	0.056002
H	-1.823660	2.330364	0.116650
H	-3.373536	2.577411	0.948064
H	-3.284292	2.682400	-0.824696
C	0.569866	1.825573	2.305193
C	1.851215	1.221901	2.230467
C	1.704049	-0.157456	2.513089
C	0.334462	-0.412877	2.741949
C	-0.365175	0.814150	2.633416
C	0.297367	3.292520	2.253183
H	-0.724535	3.514188	1.929893
H	0.975223	3.810984	1.568932
H	0.424152	3.757453	3.239991
C	3.165855	1.919329	2.119405

H	3.055725	2.950303	1.779761
H	3.852924	1.414298	1.431403
H	3.668214	1.949883	3.095337
C	2.834180	-1.112751	2.699434
H	3.314528	-0.947858	3.673892
H	3.606699	-0.998129	1.933077
H	2.497299	-2.149704	2.658347
C	-0.269594	-1.722776	3.124777
H	0.253855	-2.558339	2.651013
H	-1.321719	-1.777782	2.825887
H	-0.236578	-1.880165	4.211149
C	-1.783317	1.021207	3.051139
H	-1.870586	0.968531	4.144343
H	-2.461858	0.267246	2.640599
H	-2.152780	2.005488	2.755212
C	1.770989	2.099548	-1.571909
C	2.062427	0.848633	-2.166739
C	0.866569	0.345854	-2.730098
C	-0.161975	1.290511	-2.491784
C	0.396194	2.377570	-1.778741
C	2.774781	3.085837	-1.078830
H	3.107156	3.739194	-1.897693
H	3.667295	2.601751	-0.676362
H	2.368190	3.736629	-0.299763
C	3.417386	0.228379	-2.254236
H	4.001335	0.389848	-1.341465
H	3.995433	0.658356	-3.082886
H	3.356005	-0.851014	-2.408278

C	0.711124	-0.881537	-3.564102
H	0.671607	-0.623250	-4.631247
H	-0.210311	-1.425249	-3.329832
H	1.536404	-1.579164	-3.413821
C	-1.527728	1.202510	-3.088825
H	-1.491173	1.387006	-4.170648
H	-2.211822	1.938917	-2.661791
H	-1.978073	0.214573	-2.949526
C	-0.236166	3.707305	-1.522400
H	-0.182986	4.020407	-0.473624
H	-1.286757	3.718462	-1.820713
H	0.267210	4.488734	-2.105518

2Pr

-1691.2123191 H

Pr	0.510047	0.202541	0.025181
I	1.560600	-2.685310	-0.384634
N	-2.856774	-1.349001	-0.106595
N	-3.126895	0.748116	-0.005606
C	-2.158008	-0.194614	-0.039623
C	-4.225663	-1.137889	-0.115789
C	-4.398521	0.205267	-0.051572
C	-2.275538	-2.670309	-0.151614
H	-1.187226	-2.594139	-0.163133
H	-2.595855	-3.196235	-1.055658
H	-2.578630	-3.250484	0.725059
C	-5.207600	-2.242065	-0.184870
H	-6.223867	-1.844543	-0.173484

H	-5.105679	-2.927523	0.663826
H	-5.088532	-2.832877	-1.099968
C	-5.625553	1.031159	-0.028860
H	-6.509914	0.393944	-0.079616
H	-5.663639	1.723615	-0.877524
H	-5.696209	1.627930	0.887755
C	-2.885780	2.163031	0.083590
H	-1.813048	2.333086	0.146106
H	-3.364558	2.579692	0.973497
H	-3.271783	2.679852	-0.799338
C	0.621954	1.833397	2.268248
C	1.891972	1.208201	2.191319
C	1.724677	-0.166066	2.483634
C	0.352665	-0.397814	2.721667
C	-0.328136	0.839137	2.609684
C	0.372136	3.304411	2.214394
H	-0.647238	3.541414	1.893824
H	1.055545	3.811636	1.527548
H	0.508259	3.768176	3.200466
C	3.217294	1.880447	2.057137
H	3.120078	2.916555	1.729640
H	3.878975	1.368171	1.349762
H	3.740891	1.890812	3.022125
C	2.841194	-1.135648	2.675511
H	3.328523	-0.965189	3.645617
H	3.610536	-1.042267	1.903308
H	2.488539	-2.167717	2.649395
C	-0.268632	-1.694926	3.119280

H	0.234669	-2.541298	2.642822
H	-1.325357	-1.732861	2.834758
H	-0.223161	-1.847128	4.205933
C	-1.734352	1.075145	3.052039
H	-1.802685	1.022430	4.146550
H	-2.436190	0.336732	2.652810
H	-2.087649	2.067823	2.764738
C	1.742572	2.072253	-1.587713
C	2.022523	0.813153	-2.172025
C	0.818904	0.311278	-2.718350
C	-0.202953	1.262793	-2.479408
C	0.368087	2.354999	-1.785274
C	2.757030	3.060331	-1.120600
H	3.074930	3.706327	-1.950967
H	3.655833	2.576913	-0.731909
H	2.367052	3.718152	-0.338968
C	3.376041	0.192071	-2.273999
H	3.965600	0.341053	-1.362686
H	3.949516	0.631293	-3.100996
H	3.311580	-0.885192	-2.439387
C	0.647228	-0.920948	-3.541581
H	0.582782	-0.667343	-4.608647
H	-0.267384	-1.465250	-3.283235
H	1.477030	-1.615967	-3.406264
C	-1.573541	1.175579	-3.065002
H	-1.543497	1.345438	-4.149421
H	-2.250039	1.921965	-2.643258
H	-2.028741	0.192348	-2.909426

C	-0.254838	3.690457	-1.536438
H	-0.190952	4.013418	-0.491326
H	-1.307555	3.705219	-1.826752
H	0.249199	4.462782	-2.130942

2Nd

-1735.6202558 H

Nd	0.507200	0.189795	0.023188
I	1.503711	-2.705285	-0.350622
N	-2.874666	-1.325371	-0.098943
N	-3.108567	0.777272	-0.029585
C	-2.155690	-0.182532	-0.044263
C	-4.239485	-1.090473	-0.119825
C	-4.389155	0.256161	-0.075899
C	-2.318046	-2.657690	-0.120111
H	-1.228687	-2.600586	-0.125091
H	-2.641934	-3.191017	-1.018550
H	-2.638424	-3.218744	0.762863
C	-5.240349	-2.178085	-0.179338
H	-6.249421	-1.762473	-0.181653
H	-5.156840	-2.852123	0.680447
H	-5.125208	-2.784939	-1.084373
C	-5.601638	1.103576	-0.072723
H	-6.496634	0.481273	-0.122155
H	-5.621353	1.785829	-0.930220
H	-5.668584	1.713071	0.835768
C	-2.844629	2.189023	0.041782
H	-1.770110	2.341555	0.117597

H	-3.328956	2.626689	0.918406
H	-3.209538	2.698932	-0.853972
C	0.635795	1.857269	2.227576
C	1.893844	1.208119	2.158711
C	1.701217	-0.159459	2.468547
C	0.325478	-0.362870	2.709605
C	-0.332689	0.884415	2.579635
C	0.413065	3.331981	2.163676
H	-0.600077	3.585879	1.836513
H	1.109277	3.822256	1.477830
H	0.552649	3.798171	3.148164
C	3.232992	1.853623	2.031385
H	3.158321	2.892291	1.706762
H	3.887315	1.329923	1.325764
H	3.751525	1.850228	2.999162
C	2.801562	-1.143533	2.679729
H	3.293682	-0.958992	3.644848
H	3.570428	-1.081065	1.904017
H	2.431295	-2.169691	2.678128
C	-0.320305	-1.642086	3.125061
H	0.174086	-2.505026	2.669678
H	-1.374708	-1.668675	2.830867
H	-0.287569	-1.773918	4.214852
C	-1.733289	1.151291	3.022013
H	-1.801258	1.103164	4.116723
H	-2.450859	0.426416	2.626010
H	-2.065609	2.150418	2.732259
C	1.779995	2.038060	-1.571884

C	2.061744	0.770188	-2.136707
C	0.863917	0.268990	-2.696953
C	-0.157344	1.225590	-2.478760
C	0.409564	2.323308	-1.788423
C	2.793948	3.032777	-1.119011
H	3.106631	3.668608	-1.959239
H	3.694940	2.555450	-0.728736
H	2.406148	3.699980	-0.344552
C	3.413510	0.142011	-2.216328
H	3.995373	0.304785	-1.302580
H	3.996065	0.563699	-3.046097
H	3.345441	-0.937763	-2.363606
C	0.700268	-0.965851	-3.517764
H	0.653638	-0.714517	-4.586322
H	-0.219883	-1.506247	-3.271956
H	1.525400	-1.663282	-3.367587
C	-1.520799	1.138300	-3.081085
H	-1.476398	1.300120	-4.166238
H	-2.200248	1.889685	-2.673337
H	-1.981090	0.157692	-2.924049
C	-0.207522	3.667074	-1.572095
H	-0.156242	4.008333	-0.532396
H	-1.255544	3.684292	-1.878493
H	0.311161	4.424360	-2.173369

2U

-1651.1508234 H

U	0.460023	0.162468	0.011551

I	1.517757	-2.733263	-0.260123
N	-2.894011	-1.374737	-0.087534
N	-3.131367	0.728955	-0.024069
C	-2.177423	-0.229033	-0.050397
C	-4.259401	-1.142098	-0.084454
C	-4.410445	0.204737	-0.044197
C	-2.334494	-2.705541	-0.119782
H	-1.245205	-2.646185	-0.138176
H	-2.669631	-3.237715	-1.014837
H	-2.640165	-3.270052	0.766196
C	-5.259252	-2.231620	-0.120243
H	-6.268741	-1.817356	-0.105293
H	-5.158738	-2.901547	0.740959
H	-5.160654	-2.842357	-1.024643
C	-5.623810	1.050324	-0.021433
H	-6.518917	0.426909	-0.049414
H	-5.661138	1.727988	-0.881939
H	-5.672381	1.664534	0.885016
C	-2.864455	2.140852	0.029385
H	-1.786911	2.292037	0.071873
H	-3.322553	2.585982	0.916268
H	-3.255611	2.643816	-0.859009
C	0.484177	1.915874	2.170781
C	1.791357	1.372653	2.092516
C	1.718965	-0.001986	2.429231
C	0.367874	-0.314088	2.701699
C	-0.394636	0.872376	2.557464
C	0.151280	3.369312	2.105058

H	-0.875915	3.549794	1.774412
H	0.813831	3.906622	1.420848
H	0.254832	3.844429	3.090124
C	3.070263	2.129924	1.967679
H	2.916732	3.138099	1.580830
H	3.791658	1.629135	1.313933
H	3.551867	2.227164	2.950097
C	2.903229	-0.887587	2.628514
H	3.413143	-0.636594	3.568920
H	3.638708	-0.786359	1.824227
H	2.617893	-1.939906	2.667194
C	-0.168258	-1.623829	3.175466
H	0.443800	-2.458488	2.824866
H	-1.189743	-1.792061	2.816471
H	-0.204576	-1.667348	4.272515
C	-1.803827	1.026789	3.026321
H	-1.844595	0.997592	4.123273
H	-2.463591	0.233396	2.661857
H	-2.228771	1.986040	2.723481
C	1.697752	1.977895	-1.651977
C	1.958503	0.692019	-2.188678
C	0.743218	0.181288	-2.704145
C	-0.266492	1.153946	-2.491889
C	0.324172	2.266643	-1.845405
C	2.728666	2.980884	-1.260473
H	3.049355	3.561767	-2.137049
H	3.623477	2.514649	-0.842688
H	2.350091	3.695396	-0.525006

C	3.308157	0.060650	-2.284024
H	3.890743	0.500203	-3.104613
H	3.237130	-1.015025	-2.455948
H	3.892876	0.199913	-1.367690
C	0.550582	-1.077691	-3.482215
H	0.474273	-0.861456	-4.556718
H	-0.365312	-1.605191	-3.193996
H	1.376707	-1.774594	-3.333378
C	-1.635996	1.068617	-3.081110
H	-1.599705	1.220918	-4.168085
H	-2.308114	1.827527	-2.675448
H	-2.100459	0.092070	-2.911681
C	-0.278797	3.621652	-1.663193
H	-0.192771	4.000356	-0.639377
H	-1.336489	3.632692	-1.935025
H	0.222497	4.351784	-2.311220

2Np

-1688.6648763 H

Np	0.453431	0.159509	0.030070
I	1.432039	-2.728305	-0.415138
N	-2.921411	-1.324946	-0.125191
N	-3.125539	0.780727	-0.028263
C	-2.186828	-0.193102	-0.045099
C	-4.282066	-1.068990	-0.159096
C	-4.412413	0.278956	-0.097589
C	-2.385111	-2.665304	-0.155362
H	-1.295256	-2.623528	-0.141077

H	-2.700860	-3.181201	-1.066767
H	-2.729596	-3.232945	0.714205
C	-5.298007	-2.140540	-0.246481
H	-5.180038	-2.736661	-1.158255
H	-6.300605	-1.709691	-0.256115
H	-5.235979	-2.827363	0.604961
C	-5.612013	1.144365	-0.096847
H	-5.681352	1.741030	0.819922
H	-6.515482	0.536528	-0.167116
H	-5.610144	1.839683	-0.943994
C	-2.841502	2.186958	0.070818
H	-1.765323	2.321916	0.162480
H	-3.329786	2.615981	0.949416
H	-3.188001	2.717374	-0.819996
C	1.838164	1.120833	2.164742
C	0.585056	1.779681	2.245291
C	-0.392727	0.809428	2.581119
C	0.256030	-0.445779	2.691011
C	1.632789	-0.251660	2.452890
C	3.184506	1.756247	2.065861
H	3.838445	1.247471	1.349137
H	3.695569	1.718920	3.037046
H	3.122143	2.804595	1.771581
C	0.376579	3.257364	2.219338
H	1.080986	3.758465	1.550221
H	0.515240	3.695009	3.217095
H	-0.632259	3.529384	1.893615
C	-1.788072	1.081124	3.037211

H	-2.112344	2.088715	2.768452
H	-1.849735	1.013368	4.131330
H	-2.515443	0.370848	2.632334
C	-0.402349	-1.725599	3.084115
H	-1.452978	-1.742961	2.775936
H	-0.384754	-1.867994	4.172971
H	0.093347	-2.586680	2.626968
C	2.725004	-1.247498	2.651245
H	2.347362	-2.270860	2.630759
H	3.214879	-1.083428	3.621241
H	3.497139	-1.176803	1.879442
C	2.038715	0.798787	-2.092632
C	1.760601	2.054549	-1.496874
C	0.395415	2.358457	-1.723698
C	-0.173021	1.284744	-2.450401
C	0.842386	0.324737	-2.681871
C	3.387489	0.164824	-2.180379
H	3.962335	0.296715	-1.257298
H	3.979433	0.606804	-2.992916
H	3.313935	-0.909741	-2.360162
C	2.779708	3.035182	-1.025868
H	2.397267	3.687062	-0.236219
H	3.091070	3.687124	-1.854342
H	3.679900	2.545947	-0.649386
C	-0.208621	3.704965	-1.489384
H	-1.256431	3.737237	-1.795255
H	0.317684	4.464671	-2.081221
H	-0.153290	4.030528	-0.445238

C	-1.530323	1.224258	-3.070147
H	-2.005526	0.247443	-2.933889
H	-1.471387	1.402472	-4.152006
H	-2.204362	1.978428	-2.658640
C	0.676872	-0.884031	-3.540692
H	1.499270	-1.588493	-3.409678
H	0.635220	-0.599024	-4.600956
H	-0.246336	-1.428695	-3.315939

2Pu

-1728.1533316 H

Pu	0.459334	0.161445	-0.003300
I	1.578925	-2.699389	-0.102611
N	-2.834002	-1.410505	0.084732
N	-3.131813	0.684834	0.136948
C	-2.149757	-0.244503	0.109048
C	-4.205821	-1.218050	0.091909
C	-4.395917	0.123414	0.124434
C	-2.239259	-2.725662	0.056994
H	-1.151794	-2.639596	0.043919
H	-2.555795	-3.268235	-0.838747
H	-2.534031	-3.296808	0.942364
C	-5.173187	-2.336820	0.068569
H	-5.057469	-2.953440	-0.829767
H	-6.194386	-1.952184	0.080546
H	-5.052284	-2.995021	0.936193
C	-5.633260	0.933595	0.145256
H	-5.698880	1.550967	1.048501

H	-6.509805	0.284111	0.121694
H	-5.691659	1.605506	-0.718618
C	-2.910986	2.105471	0.157720
H	-1.839594	2.287936	0.208515
H	-3.393379	2.556318	1.028274
H	-3.307958	2.572850	-0.747226
C	1.933414	1.517825	1.847809
C	0.647852	2.107096	1.954469
C	-0.226357	1.136565	2.504440
C	0.514669	-0.051394	2.715816
C	1.852096	0.189635	2.332441
C	3.217753	2.212919	1.543304
H	3.853745	1.640988	0.858958
H	3.799517	2.357219	2.463369
H	3.057436	3.197601	1.102834
C	0.346352	3.556175	1.763786
H	0.947132	3.996375	0.963429
H	0.561510	4.127738	2.676588
H	-0.704720	3.736853	1.519433
C	-1.593103	1.376098	3.055422
H	-1.971105	2.362507	2.779813
H	-1.566700	1.343981	4.152194
H	-2.324207	0.627960	2.733258
C	-0.026790	-1.299286	3.328440
H	-1.067016	-1.471749	3.030552
H	-0.012813	-1.244938	4.425445
H	0.551804	-2.176385	3.027928
C	3.027272	-0.699236	2.562460

H	2.725899	-1.735489	2.721483
H	3.585452	-0.365280	3.448154
H	3.723100	-0.696235	1.718190
C	1.743528	0.544102	-2.345870
C	1.477082	1.859937	-1.893129
C	0.082852	2.089011	-1.996224
C	-0.514457	0.908814	-2.499183
C	0.512339	-0.044136	-2.715593
C	3.102650	-0.055985	-2.488729
H	3.745488	0.172990	-1.631353
H	3.610626	0.330915	-3.381960
H	3.054620	-1.143493	-2.568086
C	2.498460	2.922248	-1.669679
H	2.163926	3.675065	-0.951360
H	2.710765	3.450371	-2.610020
H	3.446452	2.515908	-1.311485
C	-0.550802	3.437154	-1.884211
H	-1.621538	3.397344	-2.093746
H	-0.109325	4.124772	-2.616630
H	-0.418688	3.901357	-0.901525
C	-1.920644	0.735045	-2.969159
H	-2.318963	-0.253885	-2.722483
H	-1.979835	0.840974	-4.060637
H	-2.596261	1.476676	-2.538502
C	0.303074	-1.376023	-3.354515
H	1.170752	-2.025090	-3.229935
H	0.110643	-1.261759	-4.429983
H	-0.554736	-1.908271	-2.928887

S8. References

(1) Morss, L. R.; Edelstein, N. M.; Fuger, J. *The Chemistry of the Actinide and Transactinide Elements*; Springer Dordrecht, 2011. DOI: https://doi.org/10.1007/978-94-007-0211-0.

(2) Scott, B. L. *Actinide Research Quarterly*; Los Alamos National Laboratory, 2015. https://www.osti.gov/servlets/purl/1188164DOI: https://doi.org/10.2172/1188164.

(3) Goodwin, C. A. P.; Janicke, M. T.; Scott, B. L.; Gaunt, A. J. [$AnI_3(THF)_4$] (An = Np, Pu) preparation bypassing An^0 metal precursors: access to Np^{3+}/Pu^{3+} nonaqueous and organometallic complexes. *J. Am. Chem. Soc.* **2021**, *143* (49), 20680-20696. DOI: https://doi.org/10.1021/jacs.1c07967

(4) Evans, W. J.; Kozimor, S. A.; Ziller, J. W.; Fagin, A. A.; Bochkarev, M. N. Facile syntheses of unsolvated UI_3 and tetramethylcyclopentadienyl uranium halides. *Inorg. Chem.* **2005**, *44* (11), 3993-4000. DOI: https://doi.org/10.1021/ic0482685

(5) Talavera, G.; Pena, J.; Alcarazo, M. Dihalo(imidazolium)sulfuranes: A Versatile Platform for the Synthesis of New Electrophilic Group-Transfer Reagents. *J. Am. Chem. Soc.* **2015**, *137* (27), 8704-8707. DOI: https://doi.org/10.1021/jacs.5b05287

(6) Ansell, M. B.; Roberts, D. E.; Cloke, F. G. N.; Navarro, O.; Spencer, J. Synthesis of an [$(NHC)_2Pd(SiMe_3)_2$] Complex and Catalytic *cis*-Bis(silyl)ations of Alkynes with Unactivated Disilanes. *Angew. Chem., Int. Ed.* **2015**, *54* (19), 5578-5582. DOI: https://doi.org/10.1002/anie.201501764

(7) Parker, D.; Suturina, E. A.; Kuprov, I.; Chilton, N. F. How the Ligand Field in Lanthanide Coordination Complexes Determines Magnetic Susceptibility Anisotropy, Paramagnetic NMR Shift, and Relaxation Behavior. *Acc. Chem. Res.* **2020**, *53* (8), 1520-1534. DOI: https://doi.org/10.1021/acs.accounts.0c00275

(8) Muller, N.; Lauterbur, P. C.; Goldenson, J. Nuclear Magnetic Resonance Spectra of Phosphorus Compounds. *J. Am. Chem. Soc.* **2002**, *78* (15), 3557-3561. DOI: https://doi.org/10.1021/ja01596a002

(9) den Haan, K. H.; Teuben, J. H. Formation of a novel yttrium hydride with bridging 1,2,3,4-tetramethylfulvene and hydride ligands. *J. Chem. Soc., Chem. Commun.* **1986**, (9), 682-683. DOI: https://doi.org/10.1039/C39860000682

(10) Mehdoui, T.; Berthet, J. C.; Thuéry, P.; Salmon, L.; Riviere, E.; Ephritikhine, M. Lanthanide(III)/actinide(III) differentiation in the cerium and uranium complexes $[M(C_5Me_5)_2(L)]^{0,+}$ (L=2,2'-bipyridine, 2,2':6',2''-terpyridine): structural, magnetic, and reactivity studies. *Chem. Eur. J.* **2005**, *11* (23), 6994-7006. DOI: https://doi.org/10.1002/chem.200500479

(11) Mehdoui, T.; Berthet, J. C.; Thuéry, P.; Ephritikhine, M. The remarkable efficiency of N-heterocyclic carbenes in lanthanide(III)/actinide(III) differentiation. *Chem. Commun.* **2005**, 2860-2862. DOI: https://doi.org/10.1039/B503526K

(12) Ortu, F.; Fowler, J. M.; Burton, M.; Formanuik, A.; Mills, D. P. A structural investigation of heteroleptic lanthanide substituted cyclopentadienyl complexes. *New J. Chem.* **2015**, *39* (10), 7633-7639. DOI: https://doi.org/10.1039/C5NJ00761E

(13) Asprey, L. B.; Keenan, T. K.; Kruse, F. H. Preparation and Crystal Data for Lanthanide and Actinide Triiodides. *Inorg. Chem.* **2002**, *3* (8), 1137-1141. DOI: https://doi.org/10.1021/ic50018a015

(14) Cary, S. K.; Su, J.; Galley, S. S.; Albrecht-Schmitt, T. E.; Batista, E. R.; Ferrier, M. G.; Kozimor, S. A.; Mocko, V.; Scott, B. L.; Van Alstine, C. E.; White, F. D.; Yang, P. A series of dithiocarbamates for americium, curium, and californium. *Dalton Trans.* **2018**, *47* (41), 14452-14461. DOI: https://doi.org/10.1039/C8DT02658K

(15) Knab, D. Determination of americium in small environmental samples. *Anal. Chem.* **2002**, *51* (7), 1095-1097. DOI: https://doi.org/10.1021/ac50043a077

(16) Jakopič, R.; Fankhauser, A.; Aregbe, Y.; Richter, S.; Crozet, M.; Maillard, C.; Rivier, C.; Roudil, D.; Marouli, M.; Tzika, F.; Altzitzoglou, T.; Pommé, S. ^{243}Am certified reference material for mass spectrometry. *J. Radioanal. Nucl. Chem.* **2021**, *327* (1), 495-504. DOI: https://doi.org/10.1007/s10967-020-07521-x

(17) Goodwin, C. A. P.; Su, J.; Albrecht-Schmitt, T. E.; Blake, A. V.; Batista, E. R.; Daly, S. R.; Dehnen, S.; Evans, W. J.; Gaunt, A. J.; Kozimor, S. A.; Lichtenberger, N.; Scott, B. L.; Yang, P. [$Am(C_5Me_4H)_3$]: An Organometallic Americium Complex. *Angew. Chem., Int. Ed.* **2019**, *58* (34), 11695-11699. DOI: https://doi.org/10.1002/anie.201905225

(18) Goodwin, C. A. P.; Schlimgen, A. W.; Albrecht-Schönzart, T. E.; Batista, E. R.; Gaunt, A.; Janicke, M. T.; Kozimor, S. A.; Scott, B. L.; Stevens, L. M.; White, F. D.; Yang, P. Structural and spectroscopic comparison of soft-Se vs hard-O donor bonding in trivalent americium/neodymium molecules. *Angew. Chem., Int. Ed.* **2021**, *60* (17), 9459-9466. DOI: https://doi.org/10.1002/anie.202017186

(19) *Rigaku Oxford Diffraction, (2022), CrysAlisPro Software system, version 1.171.42, Rigaku Corporation, Wroclaw, Poland.*; Rigaku Oxford Diffraction: 2017.

(20) Dolomanov, O. V.; Bourhis, L. J.; Gildea, R. J.; Howard, J. A. K.; Puschmann, H. OLEX2: a complete structure solution, refinement and analysis program. *J. Appl. Crystallogr.* **2009**, *42* (2), 339-341. DOI: https://doi.org/10.1107/s0021889808042726

(21) Sheldrick, G. M. Crystal structure refinement with SHELXL. *Acta Crystallogr. C* **2015**, *71* (Pt 1), 3-8. DOI: https://doi.org/10.1107/S2053229614024218

(22) Sheldrick, G. M. A short history of SHELX. *Acta Crystallogr. A* **2008**, *64* (Pt 1), 112-122. DOI: https://doi.org/10.1107/S0108767307043930

(23) *Inkscape: Open Source Scalable Vector Graphics Editor. https://inkscape.org/.*

(24) Clegg, W.; Blake, A. J.; Cole, J. M.; Evans, J. S. O.; Main, P.; Parsons, S.; Watkin, D. J. *Crystal Structure Analysis*; Oxford University Press, 2009. DOI: http://doi.org/10.1093/acprof:oso/9780199219469.001.0001.

(25) Wedal, J. C.; Windorff, C. J.; Huh, D. N.; Ryan, A. J.; Ziller, J. W.; Evans, W. J. Structural variations in cyclopentadienyl uranium(III) iodide complexes. *J. Coord. Chem.* **2020**, *74* (1-3), 74-91. DOI: https://doi.org/10.1080/00958972.2020.1856824

(26) Bain, A. D. Chapter 2 - Chemical Exchange. In *Annual Reports on NMR Spectroscopy*, Webb, G. A. Ed.; Vol. 63; Academic Press, 2008; pp 23-48.

(27) *Gaussian 16 Rev. C.01*; Wallingford, CT, 2016.

(28) Ernzerhof, M.; Scuseria, G. E. Assessment of the Perdew–Burke–Ernzerhof exchange-correlation functional. *J. Chem. Phys.* **1999**, *110* (11), 5029-5036. DOI: https://doi.org/10.1063/1.478401

(29) Adamo, C.; Barone, V. Toward reliable density functional methods without adjustable parameters: The PBE0 model. *J. Chem. Phys.* **1999**, *110* (13), 6158-6170. DOI: https://doi.org/10.1063/1.478522

(30) Grimme, S.; Antony, J.; Ehrlich, S.; Krieg, H. A consistent and accurate ab initio parametrization of density functional dispersion correction (DFT-D) for the 94 elements H-Pu. *J. Chem. Phys.* **2010**, *132* (15), 154104. DOI: https://doi.org/10.1063/1.3382344

(31) Becke, A. D.; Johnson, E. R. A density-functional model of the dispersion interaction. *J. Chem. Phys.* **2005**, *123* (15), 154101. DOI: https://doi.org/10.1063/1.2065267

(32) Johnson, E. R.; Becke, A. D. A post-Hartree-Fock model of intermolecular interactions. *J. Chem. Phys.* **2005**, *123* (2), 024101. DOI: https://doi.org/10.1063/1.1949201

(33) Johnson, E. R.; Becke, A. D. A post-Hartree-Fock model of intermolecular interactions: inclusion of higher-order corrections. *J. Chem. Phys.* **2006**, *124* (17), 174104. DOI: https://doi.org/10.1063/1.2190220

(34) Dunning, T. H. Gaussian basis sets for use in correlated molecular calculations. I. The atoms boron through neon and hydrogen. *J. Chem. Phys.* **1989**, *90* (2), 1007-1023. DOI: https://doi.org/10.1063/1.456153

(35) Kendall, R. A.; Dunning, T. H.; Harrison, R. J. Electron affinities of the first-row atoms revisited. Systematic basis sets and wave functions. *J. Chem. Phys.* **1992**, *96* (9), 6796-6806. DOI: https://doi.org/10.1063/1.462569

(36) Woon, D. E.; Dunning, T. H. Gaussian basis sets for use in correlated molecular calculations. III. The atoms aluminum through argon. *J. Chem. Phys.* **1993**, *98* (2), 1358-1371. DOI: https://doi.org/10.1063/1.464303

(37) Wilson, A. K.; van Mourik, T.; Dunning, T. H. Gaussian basis sets for use in correlated molecular calculations. VI. Sextuple zeta correlation consistent basis sets for boron through

neon. *J. Mol. Struct. THEOCHEM* **1996**, *388*, 339-349. DOI: https://doi.org/10.1016/S0166-1280(96)80048-0

(38) Küchle, W.; Dolg, M.; Stoll, H.; Preuss, H. Energy-adjusted pseudopotentials for the actinides. Parameter sets and test calculations for thorium and thorium monoxide. *J. Chem. Phys.* **1994**, *100* (10), 7535-7542. DOI: https://doi.org/10.1063/1.466847

(39) Cao, X.; Dolg, M.; Stoll, H. Valence basis sets for relativistic energy-consistent small-core actinide pseudopotentials. *J. Chem. Phys.* **2003**, *118* (2), 487-496. DOI: https://doi.org/10.1063/1.1521431

(40) Cao, X.; Dolg, M. Segmented contraction scheme for small-core actinide pseudopotential basis sets. *J. Mol. Struct. THEOCHEM* **2004**, *673* (1-3), 203-209. DOI: https://doi.org/10.1016/j.theochem.2003.12.015

(41) *AIMAll (Version 19.10.12)*; Todd A. Keith, TK Gristmill Software, Overland Park KS, USA: 2019 (aim.tkgristmill.com).
